# Zinc-Mediated Transformation of 1,3-Diols to Cyclopropanes for Late-Stage Modification of Natural Products and Medicinal Agents

**DOI:** 10.1021/acs.orglett.2c02362

**Published:** 2022-07-22

**Authors:** Tristan M. McGinnis, Taylor A. Thane, Elizabeth R. Jarvo

**Affiliations:** Department of Chemistry, University of California, Irvine, California 92697, United States

## Abstract

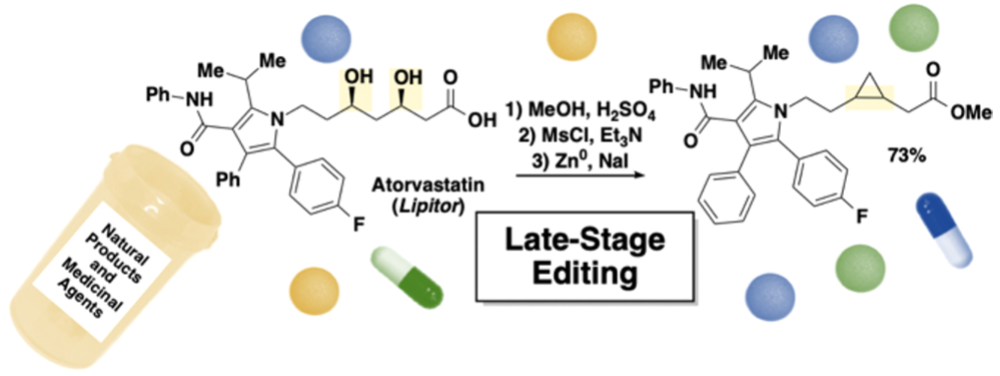

A method for incorporating cyclopropane motifs into complex molecules has been developed. Herein we report a zinc dust-mediated cross-electrophile coupling reaction of 1,3-dimesylates to synthesize cyclopropanes. 1,3-Dimesylates can be readily accessed from 1,3-diols, a functionality prevalent in many natural products and medicinal agents. The reaction conditions are mild, such that functional groups, including amides, esters, heterocycles, and alkenes, are tolerated. Notably, we have demonstrated late-stage cyclopropanation of statin medicinal agents.

Natural products provide the structural frameworks and starting points for the discovery of many medicinal agents; >35% of drugs approved from 1981 to 2019 are natural products and synthetic analogues.^1^ Late-stage modification provides a strategy for remodeling the structures of complex scaffolds and altering activity.^2^ This field has evolved significantly in the past two decades, with exciting developments in chemoselective and site-selective reactions, including alcohol functionalization and C–H activation.^3−6^ Late-stage introduction of cyclopropane moieties would also be desirable, because the cyclopropane motif is important in medicinal chemistry.^7^ Alkene cyclopropanation has been employed to introduce cyclopropanes as epoxide isosteres, for example, in epothilone derivatives,^8^ and as a derivatization and tagging strategy for chemical biology studies.^9^ These strategies require alkenes in the natural product scaffold (Figure 1a).

**Figure 1:**
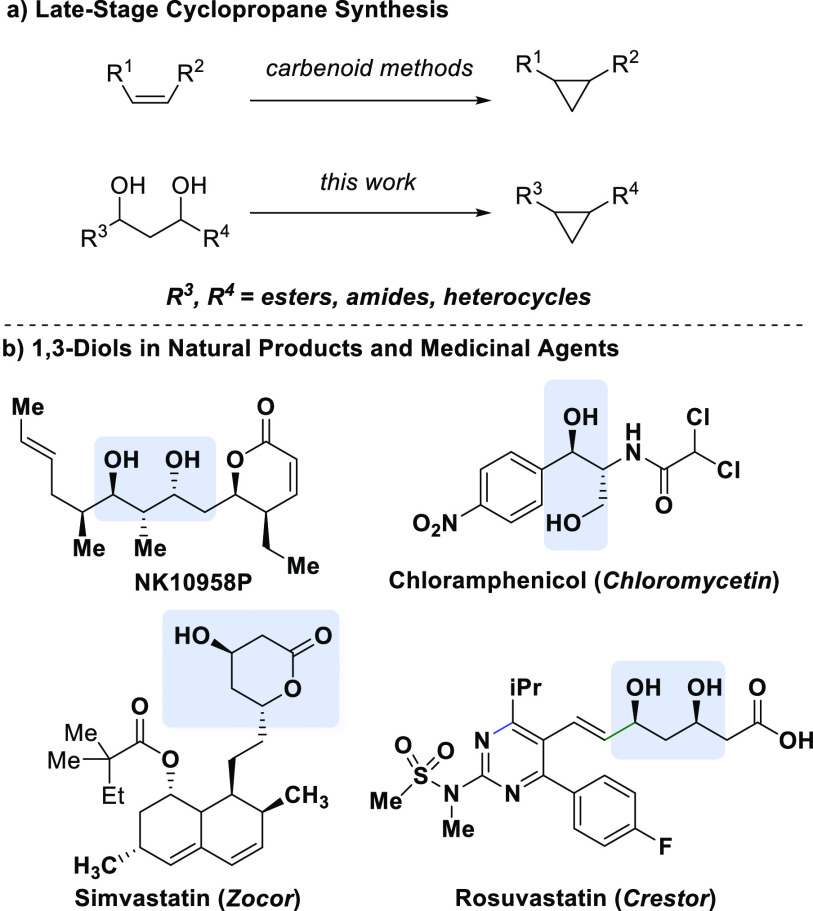


We envisioned conversion of 1,3-diols to cyclopropanes as an orthogonal approach. The appeal of this strategy is the prevalence of the alcohol functional group in natural products and medicinal agents (Figure 1b).^10^ The 1,3-diol motif is central to the backbone of polyketides, secondary metabolites with diverse biological activity ranging from anticancer to antibiotic to cholesterol-lowering activity.^11^ 1,3-Diols are also found in medicinal agents such as rosuvastatin and lumigan. In addition to modifying the pharmacokinetic properties, transformation of a 1,3-diol moiety to a cyclopropane could be employed to alter the overall conformation and relative orientation of functional groups while retaining a C(sp^3^)-rich backbone.^12^ We set out to establish a method that would achieve late-stage synthesis of cyclopropanes from complex 1,3-diols employing mild reagents. On the basis of our prior work in the development of nickel-catalyzed intramolecular cross-electrophile coupling (XEC) reactions of 1,3-dimesylates,^13^ we hypothesized that 1,3-dimesylates would undergo a reducing metal-mediated XEC reaction to form cyclopropanes.^14−17^ In this work, we report a zinc-mediated conversion of 1,3-dimesylates to cyclopropanes and demonstrate this reaction on several natural product and medicinal agent cores, including a series of statins.

To initiate our investigation, we chose 1,3-dimesylate **1** as a suitable test substrate to identify the reaction conditions for an intramolecular XEC reaction to generate cyclopropane **2** (Table 1). Due to the functional group compatibility of reducing metal reagents, zinc dust was chosen as the reductant.^18^ We also included halide salts, including MgX_2_ and NaX, in the reaction based on a working hypothesis that these reactions would proceed through 1,3-dihalide intermediates.^19,20^ In the presence of zinc dust and magnesium bromide in DMA, 1,3-dimesylate **1** was converted to cyclopropane **2** in 69% yield (entry 1). We observed no product formation in the absence of MgBr_2_ (entry 2). Increasing the number of equivalents of MgBr_2_ did not significantly impact the yield (entry 3). Diminished yields were observed with the alternative halide salts MgI_2_, NaBr, and NaI (entries 4–6, respectively). Notably, NaI resulted in 49% recovered starting material. Increasing the amount of NaI from 2 to 8 equiv and changing the solvent from DMA to THF provided a moderate increase in conversion from entry 6 (entry 7). Finally, combining MgBr_2_ and NaI did not afford a higher yield (entry 8).

**Table 1:**
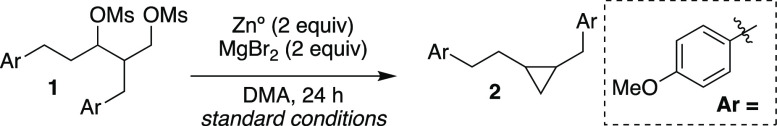
Optimization of Zinc-Mediated XEC Reactions of 1,3-Dimesylates

entry	deviation from standard conditions	dimesylate	cyclopropane (%)^a^	dr (*trans*:*cis*)
1	none	12	69	3.6:1
2	no MgBr_2_	63	<5	NA
3	3.0 equiv of MgBr_2_	21	68	3.9:1
4	Mgl_2_ instead of MgBr_2_	40	19	3.8:1
5	NaBr instead of MgBr_2_	21	52	4.2:1
6	Nal instead of MgBr_2_	49	33	4.5:1
7^b^	8.0 equiv of Nal instead of MgBr_2_	35	47	3.7:1
8	add 2.0 equiv of Nal	11	50	4.1:1

a Yields determined by comparison to PhTMS as the internal standard.

b THF instead of DMA.

With suitable reaction conditions in hand, a variety of mono- and disubstituted cyclopropanes were synthesized to demonstrate the functional group compatibility of this transformation. Monosubstituted cyclopropanes **3–8** were synthesized in good to great yields (Figure 2a). The reaction was tolerant of trifluoromethyl groups (**3**), PMB-protected diols (**4**), β-branched substrates (**3** and **5**), and aryl ethers (**6**). Cyclopropane derivatives of a terpene, (−)-borneol, and a steroid, β-sitosterol, were synthesized (**7** and **8**, respectively). We next sought to evaluate the synthesis of aryl- and alkyl-1,2-disubstituted cyclopropanes (Figure 2b). Aryl ether and alkyl ether substituents were well tolerated (**9**, **11**, and **12**). Notably, cyclopropane **11** containing a pendant ether was synthesized in a 47% yield. Dibenzofuran-substituted cyclopropane **10** and furanyl cyclopropane **12** demonstrate this method’s compatibility with heterocyclic motifs. Additionally, a series of 1,2-disubstituted cyclopropanes from polyketide scaffolds were synthesized (Figure 2c). An aldol reaction followed by sodium borohydride reduction provides rapid access to the desired diol motifs from commercially available β-ketoesters and the corresponding aldehydes or primary alcohols.^21^ To facilitate the XEC reaction toward cyclopropanes **13–15**, we found that Zn^0^ dust with 8 equiv of NaI in THF provided the best yield for this class of substrate. Cyclopropane **13** was formed in 46% yield. The natural product (*S*)-citronellal was derivatized into a 1,3-dimesylate to form cyclopropane **14** in 41% yield. Likewise, (*R*)-nopol was derivatized to form cyclopropane **15** in 43% yield. To determine whether the reaction would be amenable to larger scales, we performed the reaction of 1,3-dimesylate **16** on a 400 mg scale and were pleased to see that the yield improved to 87% (eq 1).
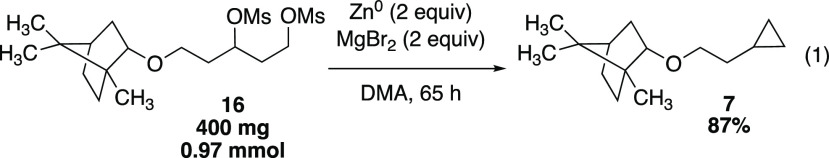
1

**Figure 2:**
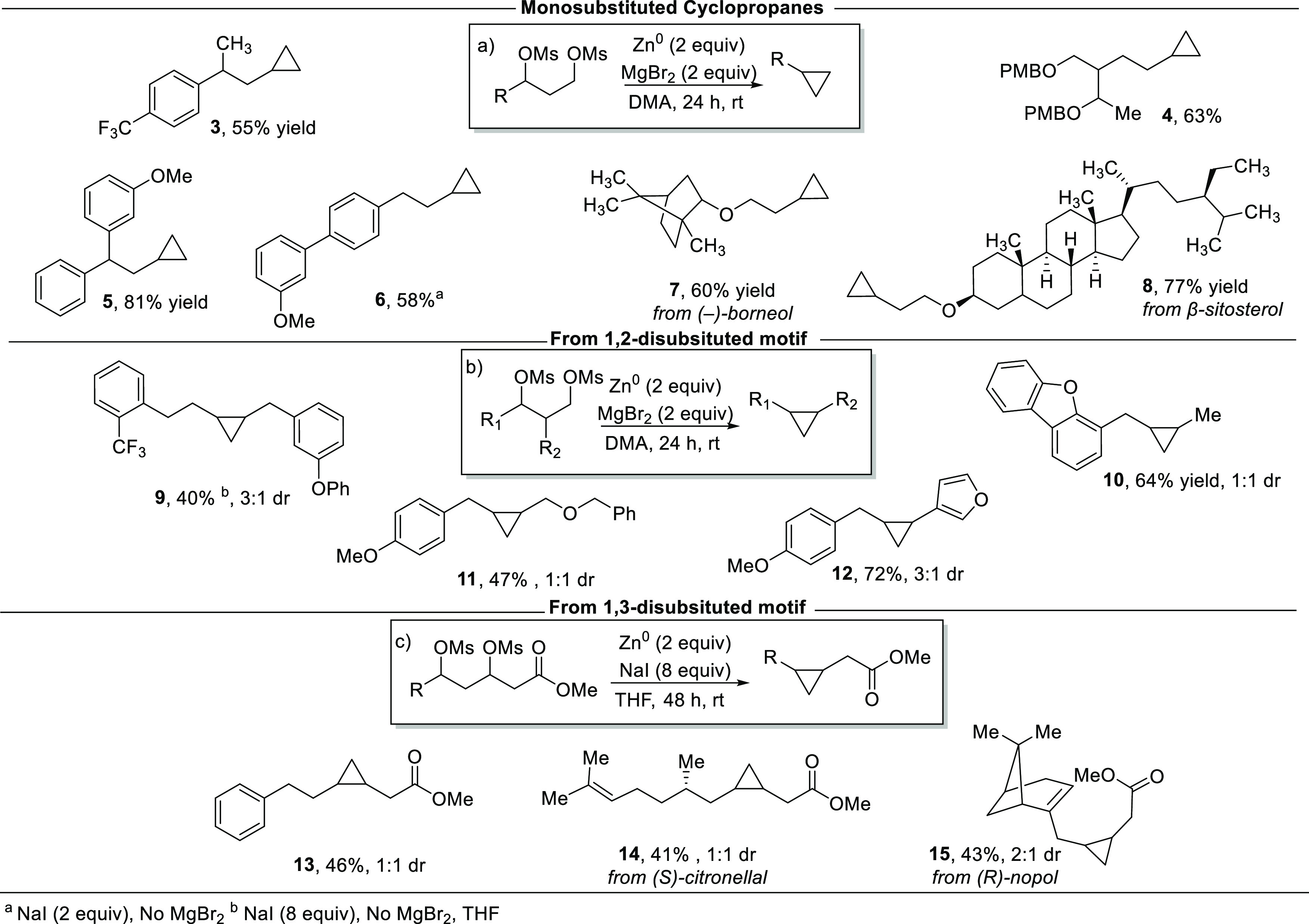
Scope of cyclopropane formation.

Statins contain or can be rapidly converted into 1,3-diol motifs, making them optimal substrates for our XEC reaction. Natural statins, such as mevastatin and lovastatin, are polyketides isolated from fungal sources.^22^ The potent ability of statins to regulate cholesterol metabolism led to the creation of many synthetic variants, such as atorvastatin and rosuvastatin.^23,24^ Many synthetic statins are available as 3,5-dihydroxy carboxylate salts. However, natural statins such as lovastatin and simvastatin are instead available as the β-hydroxy lactone. We aimed to perform a late-stage modification on these medicinal agents utilizing mild reaction conditions to form cyclopropane products (Scheme 1). To cyclize these complex diols, the carboxylic acids were protected as esters and 1,3-diols were converted to 1,3-dimesylates. In some cases, a styrene was reduced or arylated prior to the XEC reaction.^25^ Derivatives of atorvastatin (**17**), rosuvastatin (**19**), fluvastatin (**21**), pitavastatin (**23**), and simvastatin (**25**) provided cyclopropanes **18**, **20**, **22**, **24**, and **26**, respectively. In addition to 1,3-dimesylates, 1-chloro-3-mesylates undergo the reaction. Treatment of pitavastatin methyl ester with mesyl chloride resulted in the formation of allylic chloride **23**; this 1-chloro-3-mesylate underwent the zinc-mediated reaction to afford cyclopropane **24**.^26^

**Scheme 1:**
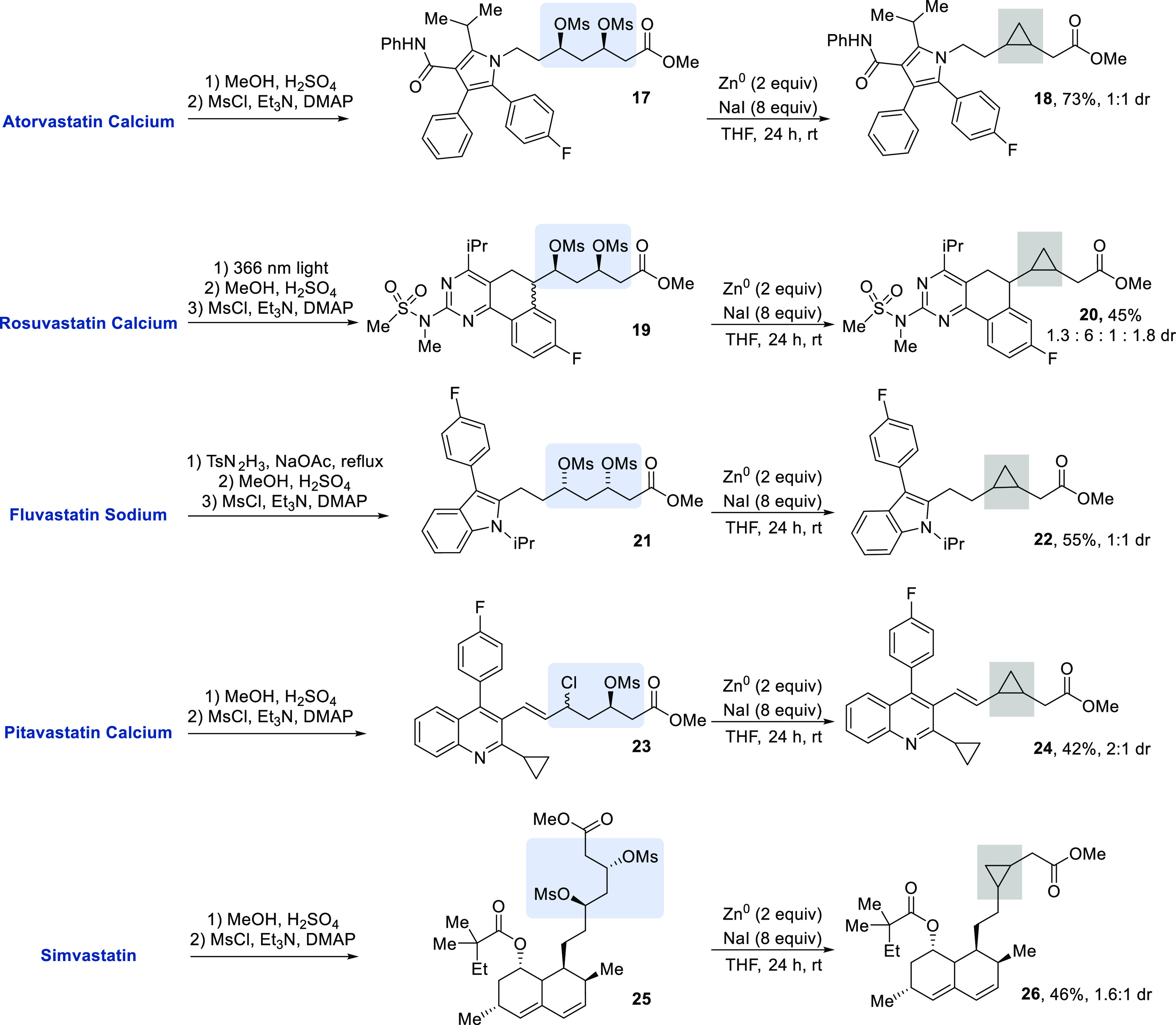
Formation of Cyclopropanes from Statin Derivatives

In summary, we report a zinc-mediated cross-electrophile coupling reaction to afford alkyl and aryl cyclopropanes for late-stage modification of natural products and medicinal agents. This transformation allows for the synthesis of cyclopropanes from monosubstituted 1,3-dimesylates, 1,2-disubstituted 1,3-dimesylates, and polyketide scaffolds. As an application of this method, statin medicinal agents were converted into 1,2-disubstituted cyclopropanes.

## Supporting Information Available

Experimental details and NMR spectra (PDF)
