# Supplementary material for: Zinc-Mediated Transformation of 1,3-Diols to Cyclopropanes for Late-Stage Modification of Natural Products and Medicinal Agents
Source: Org Lett. 2022 Jul 22;24(30):5619–23. doi: 10.1021/acs.orglett.2c02362 (PMC9361355; doi:10.1021/acs.orglett.2c02362)
Supplement: Supplementary file 1 — ol2c02362_si_001.pdf [file ol2c02362_si_001.pdf]

Supporting Information

Zinc-Mediated Transformation of 1,3-Diols to Cyclopropanes for Late-Stage Modification of Natural Products and Medicinal Agents

Tristan M. McGinnis, Taylor A. Thane, Elizabeth R. Jarvo

Department of Chemistry, University of California, Irvine, CA 92697-2025

Table of Contents

**I.**	**General Procedures**	**S–1**
**II.**	**Experimental**	**S–2**
	**A. General Cross-Electrophile Coupling Procedures**	**S–2**
	**a. Method A**	**S–2**
	**b. Method B**	**S–2**
	**B. Characterization for Cyclopropanes**	**S–2**
	**a. Monosubstituted Cyclopropanes**	**S–2**
	**b. Cyclopropanes from 1,2-Disubstituted Motifs**	**S–5**
	**c. Cyclopropanes from Polyketide Scaffolds**	**S–7**
	**C. Characterization of 1,3-Dimesylate Substrates**	**S–12**
**III.**	**References for Supporting Information**	**S–23**
**IV.**	**^{1}H, ^{13}C, and ^{19}F NMR Spectra**	**S–24**

I. GENERAL PROCEDURES

All reactions were carried out under a N_2 atmosphere, unless otherwise stated. All glassware was either oven-dried or flame-dried prior to use. Dimethylacetamide (DMA), diethyl ether (Et_2O), dichloromethane (DCM), hexanes (hex), triethylamine (Et_3N), and tetrahydrofuran (THF) were degassed with argon and then passed through two 4 x 36 inch columns of anhydrous neutral A-2 alumina (8 x 14 mesh; LaRoche Chemicals; activated under a flow of argon at 350 °C for 12 hours) to remove H_2O. Other solvents were purchased "anhydrous" commercially or were purified as described. ^{1}H NMR were recorded on Bruker DRX-400 (400 MHz ^{1}H, 100 MHz ^{13}C), CRYO-500 (500 MHz ^{1}H, 125.7 MHz ^{13}C), GN-500 (500 MHz ^{1}H, 125.7 MHz ^{13}C), or AVANCE-600 (150 MHz ^{13}C, 564.6 MHz ^{19}F) spectrometers. Proton chemical shifts are reported in ppm (δ) relative to internal tetramethylsilane (TMS, δ 0.00) unless otherwise noted. Data are reported as follows: chemical shift (multiplicity [singlet (s), broad singlet (br s), doublet (d), doublet of doublets (dd), doublet of doublet of doublets (ddd), triplet (t), doublet of triplets (dt), triplet of doublets (td), doublet of doublet of triplets (ddt), quartet (q), quintet (quint), quintet of triplets (quintt), quintet of doublets (quintd), sextet (sext), septet (sept), octet (oct), nonuplet (non), multiplet (m), apparent singlet (as), apparent doublet (ad), apparent triplet (at), apparent quartet (aq), apparent quintet (aquint)], coupling constants [Hz], integration). Carbon chemical shifts are reported in ppm (δ) relative to TMS with the solvent resonance as the internal standard ($CDCl_3$, δ 77.16 ppm). NMR data were collected at 25 °C. Analytical thin-layer chromatography (TLC) was performed using Silica Gel 60Å F254 precoated plates (0.25 mm thickness). Visualization was accomplished by irradiation with a UV lamp and/or staining with *p*-anisaldehyde (PAA), cerium ammonium molybdate (CAM), potassium permanganate ($KMnO_4$), or phosphomolybdic acid (PMA) solutions. Flash chromatography was performed using either SiliaFlash F60 (40- 63 µm, 60 Å)

from SiliCycle, or Teledyne Isco Combiflash® Rf+ automated flash chromatography system. High resolution mass spectrometry was performed by the University of California, Irvine Mass Spectrometry Center. For reactions performed at rt, average room temperature was 20 ºC. All chemicals were purchased commercially and used as received, unless otherwise noted.

II. EXPERIMENTAL

A. General Cross-Electrophile Coupling Procedures:

Method A: Cross-Electrophile Coupling for the Synthesis of Monosubstituted Cyclopropanes or Cyclopropanes from 1,2-Disubstituted Motifs

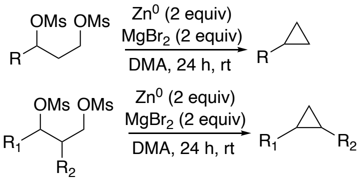

In a glovebox, a flame-dried 7mL vial equipped with a stir bar was charged with substrate (1.0 equiv), Zn^0 (2.0 equiv), $MgBr_2$ (2.0 equiv), and DMA (0.10–0.20 M in substrate). The reaction was stirred vigorously for 24 h before removal from the glovebox. Then the reaction was filtered through a plug of silica gel (eluting with 100% Et_2O), concentrated in vacuo, and purified by flash column chromatography.

Method B: Cross-Electrophile Coupling for the Synthesis of Cyclopropanes from Polyketide Scaffolds

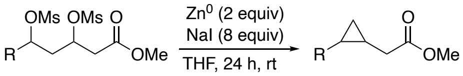

In a glovebox, a flame-dried 7mL vial equipped with a stir bar was charged with substrate (1.0 equiv), Zn^0 (2.0 equiv), NaI (8.0 equiv), and THF (0.10–0.20 M in substrate). The reaction was stirred vigorously for 24–48 h before removal from the glovebox. Then the reaction was filtered through a plug of silica gel (eluting with 100% Et_2O), concentrated in vacuo, and purified by flash column chromatography.

B. Characterization Data for Cyclopropanes:

a) Monosubstituted Cyclopropanes

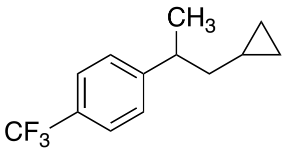

Cyclopropane 3 was prepared according to Method A. The following amounts of reagents were used: substrate **SI-1** (42 mg, 0.10 mmol, 1.0 equiv), $MgBr_2$ (37 mg, 0.20 mmol, 2.0 equiv), Zn^0

(13 mg, 0.20 mmol, 2.0 equiv) and DMA (0.5 mL, 0.2 M in substrate). The desired compound was purified by flash column chromatography (0–10% EtOAc/hexanes) to afford the title compound as a colorless oil (12 mg, 54 µmol, 55% yield). **TLC** $\mathbf{R_f}$ **=** 0.8 (25% EtOAc/hexanes); ^{1}**H NMR** (400 MHz, $CDCl_3$) δ 7.52 (d, *J* = 7.8 Hz, 2H), 7.31 (d, *J* = 7.9 Hz, 2H), 2.89 (sext, *J* = 7.1 Hz, 1H), 1.58 (quint, *J* = 7.1 Hz, 1H), 1.36 (quint, *J* = 7.0 Hz, 1H), 1.29 (d, *J* = 7.0 Hz, 3H), 0.58–0.51 (m, 1H), 0.43–0.37 (m, 1H), 0.36–0.31 (m, 1H), 0.05–0.01 (m, 1H), –0.03 to –0.08 (m, 1H); ^{13}**C NMR** ($CDCl_3$, 125 MHz) *δ* 152.0, 128.1 (q, *J* = 32.1 Hz), 127.4 (2C), 124.5 (q, *J* = 271.4 Hz), 125.2 (q, *J* = 3.7 Hz, 2C), 43.5, 40.5, 21.5, 9.4, 4.7, 4.5; ^{19}**F NMR** (564.6 MHz, $CDCl_3$) δ –62.2; **HRMS** (TOF MS CI^+) m/z: $[M]^+$ calculated for $C_{13}H_{15}F_3$, 228.1126; found, 228.1132.

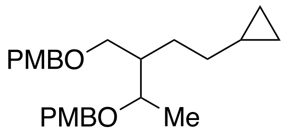

Cyclopropane 4 was prepared using Method A. The following amounts of reagents were used: substrate **SI–2** (56 mg, 0.095 mmol, 1.0 equiv), Zn^0 (12 mg, 0.19 mmol, 2.0 equiv), $MgBr_2$ (35 mg, 0.19 mmol, 2.0 equiv), DMA (1 mL, 0.1 M in substrate). The compound was purified by flash column chromatography (0–20% EtOAc/hexanes) to afford the title compound as a clear, colorless oil (24 mg, 0.060 mmol, 63%). **TLC** $\mathbf{R_f}$ = 0.5 (10% EtOAc/hexanes); The following NMR data was characterized as a 1:1 mixture of 2 diastereomers: ^{1}**H NMR** (400 MHz, $CDCl_3$) δ 7.25 (d, *J* = 8.5 Hz, 4H), 6.91–6.84 (m, 4H), 4.54–4.33 (m, 4H), 3.81 (s, 6H), 3.73–3.62 (m, 1H), 3.56–3.37 (m, 2H), 1.90–1.72 (m, 1H), 1.65–1.54 (m, 1H), 1.53–1.34 (m, 1H), 1.30–1.12 (m, 5H), 0.71–0.60 (m, 1H), 0.41 (d, *J* = 7.2 Hz, 2H), 0.05 to –0.04 (m, 2H). Analytical data is consistent with literature values.[i]

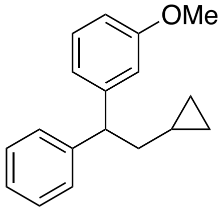

Cyclopropane 5 was prepared according to Method A. The following amounts of reagents were used: substrate **SI-3** (44 mg, 0.10 mmol, 1.0 equiv), $MgBr_2$ (37 mg, 0.20 mmol, 2.0 equiv), Zn^0 (13 mg, 0.20 mmol, 2.0 equiv) and DMA (0.5 mL, 0.2 M in substrate). The desired compound was purified by flash column chromatography (0–10% EtOAc/hexanes) to afford the title compound as a colorless oil (20. mg, 81 µmol, 81% yield). **TLC** $\mathbf{R_f}$ **=** 0.8 (25% EtOAc/hexanes); ^{1}**H NMR** (500 MHz, $CDCl_3$) *δ* 7.26–7.13 (m, 6H), 6.85 (d, *J* = 9.4 Hz, 1H), 6.81 (s, 1H), 6.70 (dd, *J* = 8.3, 2.7 Hz, 1H), 4.00 (t, *J* = 7.6 Hz, 1H), 3.75 (s, 3H), 1.96–1.87 (m, 2H), 0.62–0.57 (m, 1H), 0.39–0.35 (m, 2H), 0.07–0.03 (m, 2H). Analytical data is consistent with literature values.[i]

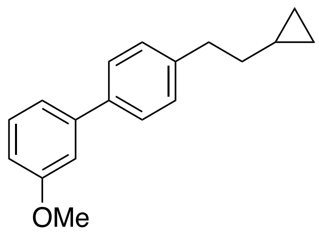

Cyclopropane 6 was prepared according to a Method A using NaI instead of $MgBr_2$. The following amounts of reagents were used: substrate **SI-4** (25 mg, 0.10 mmol, 1.0 equiv), NaI (30. mg, 0.20 mmol, 2.0 equiv), Zn^0 (13 mg, 0.20 mmol, 2.0 equiv) and DMA (0.5 mL, 0.2 M in substrate). The desired compound was purified by flash column chromatography (0–10% EtOAc/hexanes) to afford the title compound as a colorless oil (16 mg, 65 μmol, 58% yield). **TLC** $\mathbf{R_f}$ **=** 0.8 (25% EtOAc/hexanes); ^{1}**H NMR** (400 MHz, $CDCl_3$) δ 7.50 (d, *J* = 8.1 Hz, 2H), 7.33 (t, *J* = 7.8 Hz, 1H), 7.25 (d, *J* = 8.0 Hz, 2H), 7.16 (d, *J* = 7.2 Hz, 1H), 7.11 (s, 1H), 6.87 (dd, *J* = 8.2, 2.6 Hz, 1H), 3.85 (s, 3H), 2.75 (t, *J* = 7.7 Hz, 2H), 1.55 (q, *J* = 7.1 Hz, 2H), 0.78–0.68 (m, 1H), 0.44 (aq, *J* = 5.7 Hz, 2H), 0.06 (q, *J* = 5.1 Hz, 2H). Analytical data is consistent with literature values.[i]

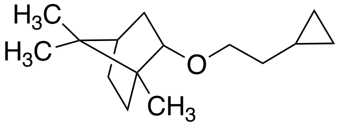

Cyclopropane 7 was prepared according to Method A. The following amounts of reagents were used: substrate **16** (41 mg, 0.10 mmol, 1.0 equiv), $MgBr_2$ (37 mg, 0.20 mmol, 2.0 equiv), Zn^0 (13 mg, 0.20 mmol, 2.0 equiv) and DMA (0.5 mL, 0.2 M in substrate). The desired compound was purified by flash column chromatography (0–10% EtOAc/hexanes) to afford the title compound as a colorless oil (13 mg, 60 μmol, 60% yield); **400 mg Scale Reaction** The following amounts of reagents were used: substrate **16** (400 mg, 0.97 mmol, 1.0 equiv), $MgBr_2$ (360 mg, 1.9 mmol, 2.0 equiv), Zn^0 (130 mg, 1.9 mmol, 2.0 equiv) and DMA (5.0 mL, 0.19 M in substrate). The desired compound was purified by flash column chromatography (0–10% EtOAc/hexanes) to afford the title compound as a colorless oil (190 mg, 0.84 mmol, 87% yield); **TLC** $\mathbf{R_f}$ = 0.9 (25% EtOAc/hexanes); ^{1}**H NMR** (400 MHz, $CDCl_3$) δ 3.58–3.54 (m, 1H), 3.53–3.38 (m, 2H), 2.15–2.07 (m, 1H), 2.03–1.95 (m, 1H), 1.73–1.64 (m, 1H), 1.61 (t, *J* = 1.6 Hz, 1H), 1.44 (q, *J* = 6.7 Hz, 2H), 1.25–1.14 (m, 2H), 1.01 (dd, *J* = 13.0, 3.4 Hz, 1H), 0.87 (s, 3H), 0.85 (s, 3H), 0.84 (s, 3H), 0.78–0.68 (m, 1H), 0.43–0.38 (m, 2H), 0.06–0.02 (m, 2H). Analytical data is consistent with literature values.[i]

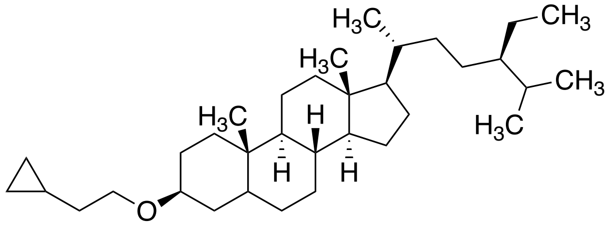

Cyclopropane 8 was prepared according to Method A. The following amounts of reagents were used: substrate **SI-5** (68 mg, 0.10 mmol, 1.0 equiv), $MgBr_2$ (37 mg, 0.20 mmol, 2.0 equiv), Zn^0 (13 mg, 0.20 mmol, 2.0 equiv) and DMA (0.5 mL, 0.2 M in substrate). The desired compound was

purified by flash column chromatography (0–10% EtOAc/hexanes) to afford the title compound as a colorless oil (37 mg, 77 μmol, 77% yield). **TLC R_f** = 0.9 (25% EtOAc/hexanes); **^{1}H NMR** (400 MHz, $CDCl_3$) δ 3.54–3.48 (m, 2H), 3.21 (quint, *J* = 15.8, 11.1, 4.5 Hz, 1H), 1.96 (dt, *J* = 12.6, 3.4 Hz, 1H), 1.88–0.68 (m, 49H), 0.65 (s, 3H), 0.43–0.39 (m, 2H), 0.06–0.02 (m, 2H). Analytical data is consistent with literature values.[i]

b) Cyclopropanes from 1,2-Disubstituted Motifs

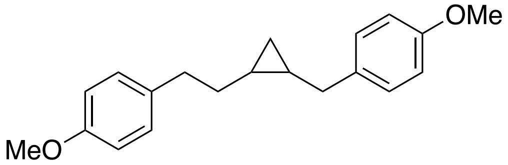

Cyclopropane 2 was prepared according to Method A. The following amounts of reagents were used: substrate **1** (0.34 ml, 0.10 mmol, 1.0 equiv, 0.29 M stock soln. of substrate in Et_2O), $MgBr_2$ (37 mg, 0.20 mmol, 2.0 equiv), Zn^0 (13 mg, 0.20 mmol, 2.0 equiv) and DMA (0.5 mL, 0.2 M in substrate). The desired compound was purified by flash column chromatography (0–10% EtOAc/hexanes) to afford the title compound in a 3.6:1 (trans:cis) mixture of diastereomers as a colorless oil (22 mg, 74 μmol, 74% yield). **TLC R_f =** 0.8 (25% EtOAc/hexanes). Analytical data is consistent with literature values.[i]

Major Diastereomer: ^{1}H NMR (400 MHz, $CDCl_3$) δ 7.12 (d, *J* = 8.7 Hz, 2H), 7.05 (d, *J* = 8.4 Hz, 2H), 6.84–6.79 (m, 4H), 3.78 (s, 3H), 3.77 (s, 3H), 2.58 (at, *J* = 9.8 Hz, 2H), 2.47 (t, *J* = 7.3 Hz, 2H), 1.54–1.47 (m, 2H), 0.74–0.66 (m, 1H), 0.63–0.55 (m, 1H), 0.36–0.32 (m, 1H), 0.31–0.26 (m, 1H).

Minor Diastereomer: ^{1}H NMR (400 MHz, $CDCl_3$) δ 7.17 (d, *J* = 8.4 Hz, 2H), 7.10 (d, *J* = 8.4 Hz, 2H), 6.84–6.79 (m, 4H), 3.78 (s, 3H), 3.77 (s, 3H), 2.67–2.61 (m, 2H), 2.52–2.41 (m, 2H), 1.80–1.71 (m, 2H), 1.04–0.95 (m, 1H), 0.88–0.79 (m, 2H), –0.07 (q, *J* = 5.2 Hz, 1H).

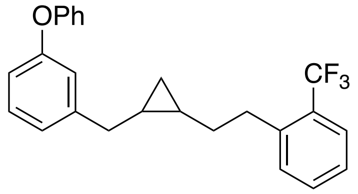

Cyclopropane 9 was prepared using Method B. The following amounts of reagents were used: substrate **SI–6** (30. mg, 0.051 mmol, 1.0 equiv), Zn^0 (7.0 mg, 0.10 mmol, 2.0 equiv), NaI (61 mg, 0.41 mmol, 8.0 equiv), THF (0.5 mL, 0.10 M in substrate). The compound was purified by flash column chromatography (0–20% EtOAc/hexanes) to afford the title compound as a clear, colorless oil as a 3:1 (trans:cis) mixture of diastereomers (8.0 mg, 0.020 mmol, 40%); **TLC R_f** = 0.3 (100% hexanes). Analytical data is consistent with literature values.[i]

Major Diastereomer: ^{1}H NMR (500 MHz, $CDCl_3$) δ 7.59 (t, *J* = 8.0 Hz, 1H), 7.42 (t, *J* = 8.1 Hz, 1H), 7.35–7.20 (m, 5H), 7.11–7.04 (m, 1H), 7.04–6.93 (m, 3H), 6.90 (s, 1H), 6.83 (d, *J* = 7.6 Hz,

1H), 2.96–2.65 (m, 2H), 2.52 (d, J = 6.6 Hz, 2H), 1.59–1.47 (m, 2H), 0.80–0.69 (m, 1H), 0.69–0.60 (m, 1H), 0.41–0.36 (m, 1H), 0.36–0.29 (m, 1H).

Minor Diastereomer: ^{1}H NMR (500 MHz, $CDCl_3$) δ 7.59 (t, J = 8.0 Hz, 1H), 7.42 (t, J = 8.1 Hz, 1H), 7.35–7.20 (m, 5H), 7.11–7.04 (m, 1H), 7.04–6.93 (m, 3H), 6.90 (s, 1H), 6.83 (d, J = 7.6 Hz, 1H), 2.96–2.65 (m, 2H), 2.52 (d, J = 6.6 Hz, 2H), 1.83–1.73 (m, 1H), 1.59–1.47 (m, 2H), 1.11–1.02 (m, 1H), 0.96–0.80 (m, 1H), –0.02 to –0.07 (m, 1H).

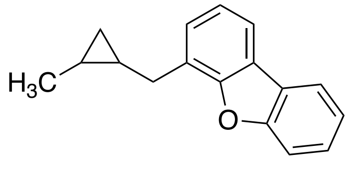

Cyclopropane 10 was prepared according to Method A. The following amounts of reagents were used: substrate **SI-7** (43 mg, 0.10 mmol, 1.0 equiv), $MgBr_2$ (37 mg, 0.20 mmol, 2.0 equiv), Zn^0 (13 mg, 0.20 mmol, 2.0 equiv) and DMA (0.5 mL, 0.2 M in substrate). The desired compound was purified by flash column chromatography (0–10% EtOAc/hexanes) to afford the title compound in a 1:1 mixture of diastereomers as a colorless oil (15 mg, 64 μmol, 64% yield). **TLC R_f =** 0.8 (25% EtOAc/hexanes); **^{1}H NMR** (400 MHz, $CDCl_3$) δ 7.94 (d, J = 7.8 Hz, 2H, both diastereomers), 7.80 (d, J = 7.6 Hz, 2H, both diastereomers), 7.58 (d, J = 8.2 Hz, 2H, both diastereomers), 7.45–7.42 (m, 2H, both diastereomers), 7.38–7.27 (m, 6H both diastereomers), 3.05–2.95 (m, 2H, one diastereomer), 2.92–2.90 (m, 2H, other diastereomer), 1.23–1.19 (m, 1H, one diastereomer), 1.17 (d, J = 6.4 Hz, 3H, other diastereomer), 1.06 (d, J = 6.0 Hz, 3H, one diastereomer), 0.97–0.85 (m, 2H, other diastereomer), 0.78–0.70 (m, 2H, one diastereomer), 0.48–0.45 (m, 1H, other diastereomer), 0.30–0.27 (m, 1H, one diastereomer), 0.03–0.01 (m, 1H, other diastereomer). Analytical data is consistent with literature values.[i]

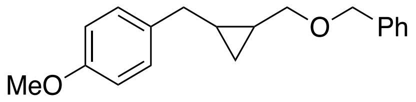

Cyclopropane 11 was prepared according to Method A. The following amounts of reagents were used: substrate **SI-8** (43 mg, 0.10 mmol, 1.0 equiv), $MgBr_2$ (37 mg, 0.20 mmol, 2.0 equiv), Zn^0 (13 mg, 0.20 mmol, 2.0 equiv) and DMA (0.5 mL, 0.2 M in substrate). The desired compound was purified by flash column chromatography (0–10% EtOAc/hexanes) to afford the title compound in a 1:1 mixture of diastereomers as a colorless oil (12 mg, 47 μmol, 47% yield). **TLC R_f =** 0.9 (25% EtOAc/hexanes); **^{1}H NMR** (400 MHz, $CDCl_3$) δ 7.34–7.23 (m, 10H, both diastereomers), 7.20–7.16 (m, 4H, both diastereomers), 6.84–6.80 (m, 4H, both diastereomers), 4.53 (aq, J = 11.8 Hz, 2H, one diastereomer), 4.48 (s, 2H, other diastereomer), 3.78 (s, 3H, one diastereomer), 3.77 (s, 3H, other diastereomer), 3.63 (dd, J = 10.2, 6.5 Hz, 1H, one diastereomer), 3.46 (dd, J = 10.2, 8.1 Hz, 1H, other diastereomer), 3.40–3.31 (m, 2H, both diastereomers), 2.78 (dd, J = 15.1, 6.1 Hz, 1H, one diastereomer), 2.60–2.50 (m, 2H, both diastereomers), 2.43 (dd, J = 15.0, 8.2 Hz, 1H, other diastereomer), 1.29–1.21 (m, 1H, one diastereomer), 1.19–1.11 (m, 1H, other diastereomer), 1.05–0.97 (m, 1H, one diastereomer), 0.92–0.86 (m, 1H, other diastereomer), 0.85–0.80 (m, 1H, one diastereomer), 0.50–0.43 (m, 2H, both diastereomers), 0.16 (q, J = 5.5 Hz, 1H, other diastereomer); **^{13}C NMR** ($CDCl_3$, 125.7 MHz) δ 158.01 (one diastereomer), 157.95 (other

diastereomer), 138.8 (one diastereomer), 138.7 (other diastereomer), 134.4 (one diastereomer), 133.9 (other diastereomer), 129.5 (2C, one diastereomer), 129.3 (2C, other diastereomer), 128.49 (2C, one diastereomer), 128.45 (2C, other diastereomer), 127.9 (2C, one diastereomer), 127.73 (2C, other diastereomer), 127.69 (one diastereomer), 127.6 (other diastereomer), 113.87 (2C, one diastereomer), 113.85 (2C, other diastereomer), 74.2 (one diastereomer), 72.9 (other diastereomer), 72.4 (one diastereomer), 70.62 (other diastereomer), 55.40 (one diastereomer), 55.39 (other diastereomer), 38.5 (one diastereomer), 33.7 (other diastereomer), 18.6 (one diastereomer), 18.5 (other diastereomer), 17.2 (one diastereomer), 15.9 (other diastereomer), 10.3 (one diastereomer), 10.0 (other diastereomer); **HRMS** (TOF MS ES+) m/z: $[M+Na]^+$ calculated for $C_{19}H_{22}O_2Na$, 305.1518; found, 305.1525.

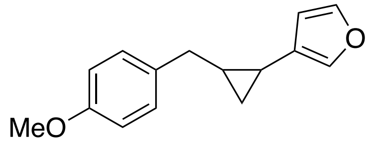

Cyclopropane 12 was prepared according to Method A. The following amounts of reagents were used: substrate **SI-9** (36 mg, 0.10 mmol, 1.0 equiv), $MgBr_2$ (37 mg, 0.20 mmol, 2.0 equiv), Zn^0 (13 mg, 0.20 mmol, 2.0 equiv) and DMA (0.5 mL, 0.2 M in substrate). The desired compound was purified by flash column chromatography (0–10% EtOAc/hexanes) to afford the title compound as a colorless oil in a 3:1 (trans:cis) mixture of diastereomers (16 mg, 72 μmol, 72% yield). **TLC** $\mathbf{R_f}$ **=** 0.8 (25% EtOAc/hexanes). Analytical data is consistent with literature values.[i]

Major Diastereomer: ^{1}**H NMR** (400 MHz, $CDCl_3$) δ 7.28 (t, *J* = 1.7 Hz, 1H), 7.21–7.20 (m, 1H), 7.17–7.15 (m, 2H), 6.85–6.81 (m, 2H), 6.11–6.10 (m, 1H), 3.78 (s, 3H), 2.67 (dd, *J* = 14.8, 6.9 Hz, 1H), 2.59 (dd, *J* = 14.9, 6.8 Hz, 1H), 1.58–1.54 (m, 1H), 1.19–1.11 (m, 1H), 0.79–0.75 (m, 2H).

Minor Diastereomer: ^{1}**H NMR** (400 MHz, $CDCl_3$) δ 7.34 (t, *J* = 1.7 Hz, 1H), 7.24 (s, 1H), 7.07–7.04 (m, 2H), 6.80–6.78 (m, 2H), 6.26–6.25 (m, 1H), 3.77 (s, 3H), 2.48 (dd, *J* = 15.1, 7.1 Hz, 1H), 2.33 (dd, *J* = 15.1, 7.2 Hz, 1H), 1.90–1.84 (m, 1H), 1.30–1.22 (m, 1H), 1.08–1.01 (dt, *J* = 8.5, 4.8 Hz, 1H), 0.51 (q, *J* = 5.7 Hz, 1H).

c) Cyclopropanes From Polyketide Scaffolds

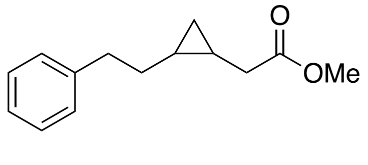

Cyclopropane 13 was prepared according to Method B and allowed to stir for 48 hours. The following amounts of reagents were used: substrate **SI-10** (48 mg, 0.12 mmol, 1.0 equiv), Zn^0 (15 mg, 0.24 mmol, 2.0 equiv), NaI (140 mg, 0.94 mmol, 8.0 equiv), THF (1.1 mL, 0.11 M in substrate). The compound was purified by flash column chromatography (0–20% EtOAc/hexanes) to afford the title compound in a 1:1 mixture of diastereomers as a clear, colorless oil (12 mg, 0.054 mmol, 46%). **TLC** $\mathbf{R_f}$ = 0.7 (20% EtOAc/hexanes); **HRMS** (TOF MS ES+) *m/z*: $[M+Na]^+$ calculated for $C_{14}H_{18}O_2Na$, 241.1205; found, 241.1205.

Major Diastereomer: ^{1}**H NMR** (600 MHz, $CDCl_3$) δ 7.37–7.30 (m, 2H), 7.27–7.20 (m, 3H), 3.75 (s, 3H), 2.81–2.73 (m, 2H), 2.38–2.33 (m, 1H), 2.28 (d, J = 7.2 Hz, 1H), 1.77–1.64 (m, 1H), 1.61–1.54 (m, 1H), 1.22–1.15 (m, 1H), 0.67–0.61 (m, 1H), 0.44–0.37 (m, 2H); ^{13}**C NMR** (150.9 MHz, $CDCl_3$) δ 174.1, 142.5, 128.5 (2C), 128.3 (2C), 125.7, 51.6, 38.8, 35.8, 33.7, 18.4, 14.5, 11.7.

Minor Diastereomer: ^{1}**H NMR** (600 MHz, $CDCl_3$) δ 7.37–7.30 (m, 2H), 7.27–7.20 (m, 3H), 3.75 (s, 3H), 2.81–2.73 (m, 2H), 2.38–2.33 (m, 1H), 2.28 (d, J = 7.2 Hz, 1H), 1.77–1.64 (m, 1H), 1.61–1.54 (m, 1H), 0.97–0.84 (m, 2H), 0.83–0.79 (m, 1H), –0.05 (q, J = 5.3 Hz, 1H); ^{13}**C NMR** (150.9 MHz, $CDCl_3$) δ 173.7, 142.4, 128.5 (2C), 128.3 (2C), 125.8, 51.6, 36.2, 35.9, 31.0, 15.3, 11.7, 10.8.

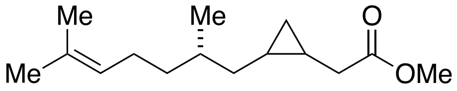

Cyclopropane 14 was prepared according to Method B and allowed to stir for 48 hours. The following amounts of reagents were used: substrate **SI-11** (26 mg, 0.061 mmol, 1.0 equiv), Zn^0 (8.0 mg, 0.12 mmol, 2.0 equiv), NaI (74 mg, 0.49 mmol, 8.0 equiv), THF (1.0 mL, 0.060 M in substrate). The compound was purified by flash chromatography (0–20% EtOAc/hexanes) to afford the title compound in a 1:1 mixture of diastereomers as a clear, colorless oil (6.0 mg, 0.025 mmol, 41%). **TLC R$_f$** = 0.7 (20% EtOAc/hexanes); **HRMS** (TOF MS CI+) *m/z*: $[M]^+$ calculated for $C_{15}H_{26}O_2$, 238.1933; found, 238.1931.

Major Diastereomer: ^{1}**H NMR** (600 MHz, $CDCl_3$) δ 5.18–5.12 (m, 1H), 3.73 (s, 3H), 2.37–2.21 (m, 2H), 2.10–1.94 (m, 3H), 1.74 (s, 3H), 1.66 (s, 3H), 1.43–1.31 (m, 2H), 1.25–1.17 (m, 2H), 1.01–0.95 (m, 3H), 0.85–0.77 (m, 1H), 0.66–0.58 (m, 1H), 0.43–0.32 (m, 2H); ^{13}**C NMR** (150.9 MHz, $CDCl_3$) δ 131.1, 125.0, 41.3, 39.0, 37.1, 36.0, 33.9, 33.1, 25.8, 19.6, 17.7, 16.8, 14.8, 13.6, 12.2.

Minor Diastereomer: ^{1}**H NMR** (600 MHz, $CDCl_3$) δ 5.18–5.12 (m, 1H), 3.75 (s, 3H), 2.37–2.21 (m, 2H), 2.10–1.94 (m, 3H), 1.74 (s, 3H), 1.66 (s, 3H), 1.43–1.31 (m, 2H), 1.25–1.17 (m, 2H), 1.16–1.10 (m, 1H), 1.01–0.95 (m, 3H), 0.93–0.86 (m, 2H), –0.03 to –0.11 (m, 1H); ^{13}**C NMR** (150.9 MHz, $CDCl_3$) δ 131.1, 125.0, 41.3, 39.0, 37.0, 35.8, 34.0, 33.1, 25.8, 19.6, 17.7, 16.7, 14.3, 13.6, 12.2.

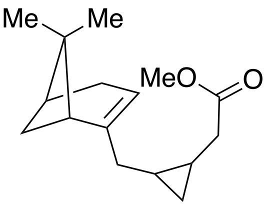

Cyclopropane 15 was prepared according to Method B and allowed to stir for 48 hours. The following amounts of reagents were used: substrate **SI-12** (75 mg, 0.17 mmol, 1.0 equiv), Zn^0 (22 mg, 0.34 mmol, 2.0 equiv), NaI (0.20 g, 1.4 mmol, 8.0 equiv), THF (1.7 mL, 0.10 M in substrate). The compound was purified by flash chromatography (0–20% EtOAc/hexanes) to afford the title compound in a 1.5:1 mixture of diastereomers as a clear, colorless oil (18 mg, 0.73 mmol, 43%).

TLC R_f = 0.8 (20% EtOAc/hexanes); **HRMS** (TOF MS CI+) *m/z*: $[M]^+$ calculated for $C_{16}H_{24}O_2$, 248.1776; found, 248.1765.

Major Diastereomer: ^{1}**H NMR** (500 MHz, $CDCl_3$) δ 5.22 (bs, 1H), 3.68 (s, 3H), 2.45–2.31 (m, 2H), 2.30–1.99 (m, 6H), 1.95–1.81 (m, 2H), 1.27 (s, 3H), 1.22–1.09 (m, 1H), 0.84 (s, 3H), 0.65–0.56 (m, 1H), 0.38–0.30 (m, 2H); ^{13}**C NMR** (125.8 MHz, $CDCl_3$) δ 173.7, 147.9, 115.9, 51.7, 46.0, 41.0, 38.9, 35.9, 33.8, 31.4, 26.5, 21.3, 16.8, 14.5, 13.4, 11.7.

Minor Diastereomer: ^{1}**H NMR** (500 MHz, $CDCl_3$) δ 5.28 (s, 1H), 3.68 (s, 3H), 2.45–2.31 (m, 2H), 2.30–1.99 (m, 6H), 1.95–1.81 (m, 2H), 1.27 (s, 3H), 1.22–1.09 (m, 1H), 0.94–0.87 (m, 1H), 0.84 (s, 3H), 0.79–0.72 (m, 1H), –0.06 to –0.12 (m, 1H); ^{13}**C NMR** (125.8 MHz, $CDCl_3$) δ 174.2, 147.9, 115.9, 51.7, 46.1, 41.0, 38.9, 35.9, 33.8, 31.7, 26.5, 21.3, 16.8, 14.2, 13.2, 11.7.

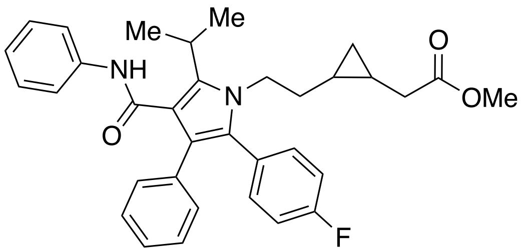

Cyclopropane 18 was prepared according to Method B and allowed to stir for 24 h. The following amounts of reagents were used: substrate **17** (77 mg, 0.11 mmol, 1.0 equiv), Zn^0 (14 mg, 0.21 mmol, 2.0 equiv), NaI (130 mg, 0.84 mmol, 8.0 equiv), THF (1.0 mL, 0.11 M in substrate). The compound was purified by flash chromatography (0–20% EtOAc/hexanes) to afford the title compound in a 1.4:1 mixture of diastereomers as a clear, colorless oil (42 mg, 0.080 mmol, 73%). **TLC R_f** = 0.2 (10% EtOAc/hexanes); **HRMS** (TOF MS ES+) *m/z*: $[M+H]^+$ calculated for $C_{34}H_{35}FO_3N_2H$, 539.2710; found, 539.2712.

Major Diastereomer: ^{1}**H NMR** (600 MHz, $CDCl_3$) δ 7.23–7.13 (m, 9H), 7.92 (d, J = 7.9 Hz, 2H), 7.03–6.95 (m, 3H), 6.87 (s, 1H), 4.04–3.77 (m, 2H), 3.66 (s, 3H), 3.60–3.52 (m, 1H), 2.30–2.21 (m, 1H), 2.09–1.95 (m, 2H), 1.56–1.51 (m, 6H), 1.45–1.36 (m, 1H), 0.69–0.54 (m, 2H), 0.28–0.22 (m, 1H), 0.17–0.12 (m, 1H); ^{13}**C NMR** (150.9 MHz, $CDCl_3$) δ 173.4, 164.8, 162.3 (d, J = 247.7 Hz), 141.5, 138.5, 134.8, 133.3 (d, J = 7.7 Hz, 2C), 130.6 (2C), 128.9, 128.8 (2C), 128.5 (2C), 126.7, 123.6, 121.8, 119.7, 115.4 (d, J = 21.6 Hz, 2C), 51.7, 44.5, 38.5, 36.0, 33.3, 31.1, 26.3, 21.9, 15.9, 14.2, 12.8, 11.3, 10.4; ^{19}**F NMR** (564.7 MHz, $CDCl_3$) δ –113.5.

Minor Diastereomer: ^{1}**H NMR** (600 MHz, $CDCl_3$) δ 7.23–7.13 (m, 9H), 7.92 (d, J = 7.9 Hz, 2H), 7.03–6.95 (m, 3H), 6.87 (s, 1H), 4.04–3.77 (m, 2H), 3.66 (s, 3H), 3.60–3.52 (m, 1H), 2.30–2.21 (m, 1H), 2.09–1.95 (m, 2H), 1.56–1.51 (m, 6H), 1.45–1.36 (m, 1H), 1.07–0.99 (m, 1H), 0.91–0.81 (m, 1H), 0.40–0.32 (m, 1H), –0.33 to –0.39 (m, 1H); ^{13}**C NMR** (150.9 MHz, $CDCl_3$) δ 173.6, 164.9, 162.4 (d, J = 247.7 Hz), 141.5, 138.5, 134.7, 133.3 (d, J = 7.7 Hz, 2C), 130.6 (2C), 128.9, 128.8 (2C), 128.5 (2C), 126.7, 123.6, 121.9, 119.7, 115.5 (d, J = 21.6 Hz, 2C), 51.8, 44.8, 38.5, 36.0, 33.3, 31.1, 26.3, 21.8, 15.9, 14.2, 12.8, 11.5, 10.4; ^{19}**F NMR** (564.7 MHz, $CDCl_3$) δ –113.4.

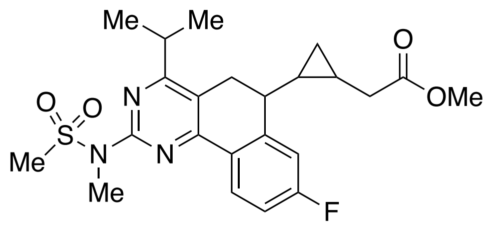

Cyclopropane 20 was prepared according to Method B and allowed to stir for 24 h. The following amounts of reagents were used: substrate **19** (54 mg, 0.083 mmol, 1.0 equiv), Zn^0 (11 mg, 0.17 mmol, 2.0 equiv), NaI (0.10 g, 0.66 mmol, 8.0 equiv), THF (1.0 mL, 0.083 M in substrate). The compound was purified by flash chromatography (0–20% EtOAc/hexanes) to afford the title compound as a 1.3 : 6.0 : 1.0 :1.8 mixture of diastereomers as a clear, colorless oil (17 mg, 0.037 mmol, 45%). **TLC** $\mathbf{R_f}$ = 0.2 (10% EtOAc/hexanes); **HRMS** (TOF MS ES+) *m/z*: $[M+H]^+$ calculated for $C_{23}H_{28}FO_4N_3SH$, 462.1863; found, 462.1843.

Major Diastereomer: ^{1}**H NMR** (500 MHz, $CDCl_3$) δ 8.39–8.28 (m, 1H), 7.28–7.05 (m, 2H), 3.85–3.51 (m, 9H), 3.42–2.95 (m, 3H), 2.65–2.14 (m, 3H), 1.41–1.27 (m, 6H), 1.23–1.14 (m, 1H), 0.85–0.76 (m, 1H), 0.71–0.60 (m, 1H), 0.58–0.46 (m, 1H); ^{13}**C NMR** (125.8 MHz, $CDCl_3$) δ 173.5, 163.5, 157.6, 144.8, 128.6, 117.3, 114.6, 114.2, 114.1, 51.6, 42.2, 38.5, 33.3, 31.3, 29.8, 27.9, 23.1, 21.6, 20.9, 19.9, 15.1, 11.8, 10.9; ^{19}**F NMR** (564.7 MHz, $CDCl_3$) δ –109.1.

Minor Diastereomer: ^{1}**H NMR** (500 MHz, $CDCl_3$) δ 8.39–8.28 (m, 1H), 7.28–7.05 (m, 2H), 3.85–3.51 (m, 9H), 3.42–2.95 (m, 3H), 2.65–2.14 (m, 3H), 1.41–1.27 (m, 6H), 1.23–1.14 (m, 1H), 1.05–0.97 (m, 2H), 0.31–0.17 (m, 1H); ^{13}**C NMR** (125.8 MHz, $CDCl_3$) δ 172.9, 165.5, 158.3, 144.8, 128.2, 117.7, 114.5, 114.2, 113.8, 51.8, 42.4, 38.2, 34.0, 31.1, 29.0, 28.2, 23.1, 21.3, 21.1, 19.8, 14.2, 12.0, 9.5; ^{19}**F NMR** (564.7 MHz, $CDCl_3$) δ –109.1.

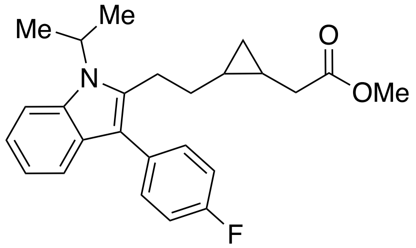

Cyclopropane 22 was prepared according to Method B and allowed to stir for 24 h. The following amounts of reagents were used: substrate **21** (0.11 g, 0.18 mmol, 1.0 equiv), Zn^0 (23 mg, 0.36 mmol, 2.0 equiv), NaI (0.22 g, 1.5 mmol, 8.0 equiv), THF (2.0 mL, 0.090 M in substrate). The compound was purified by flash chromatography (0–20% EtOAc/hexanes) to afford the title compound in a 1:1 mixture of diastereomers as a clear, colorless oil (39 mg, 0.10 mmol, 55%). **TLC** $\mathbf{R_f}$ = 0.2 (10% EtOAc/hexanes); **HRMS** (TOF MS ES+) *m/z*: $[M+H]^+$ calculated for $C_{25}H_{28}FO_2NH$, 394.2182; found, 394.2192.

Major Diastereomer: ^{1}**H NMR** (600 MHz, $CDCl_3$) δ 7.56 (d, J = 8.4 Hz, 1H), 7.50 (t, J = 7.5 Hz, 1H), 7.39–7.34 (m, 2H), 7.17–7.10 (m, 3H), 7.06 (t, J = 7.5 Hz, 1H), 4.69–4.60 (m, 1H), 3.64 (s, 3H), 2.93–2.74 (m, 2H), 2.28–2.11 (m, 2H), 1.79–1.76 (m, 6H), 1.56–1.42 (m, 2H), 0.71–0.67 (m, 1H), 0.59–0.53 (m, 1H), 0.34–0.27 (m, 2H); ^{13}**C NMR** (150.9 MHz, $CDCl_3$) δ 173.5, 161.5 (d, J

= 250.7 Hz), 136.7, 134.1, 132.0 (d, J = 3.3 Hz), 131.5 (d, J = 7.7 Hz), 128.5, 120.7, 119.2, 119.0, 115.3 (d, J = 21.0 Hz, 2C), 111.8, 51.6, 47.3, 38.6, 35.2, 33.6, 30.1, 25.1, 21.6, 18.6, 15.4, 11.8, 10.9; **^{19}F NMR** (564.7 MHz, $CDCl_3$) δ –117.0.

Minor Diastereomer: ^{1}H NMR (600 MHz, $CDCl_3$) δ 7.56 (d, J = 8.4 Hz, 1H), 7.50 (t, J = 7.5 Hz, 1H), 7.39–7.34 (m, 2H), 7.17–7.10 (m, 3H), 7.06 (t, J = 7.5 Hz, 1H), 4.69–4.60 (m, 1H), 3.65 (s, 3H), 2.93–2.74 (m, 2H), 2.28–2.11 (m, 2H), 1.79–1.76 (m, 6H), 1.56–1.42 (m, 2H), 1.10–1.03 (m, 1H), 0.83–0.76 (m, 2H), –0.17 (q, J = 5.6 Hz, 1H); **^{13}C NMR** (150.9 MHz, $CDCl_3$) δ 173.5, 161.4 (d, J = 250.0 Hz), 136.7, 134.1, 131.9 (d, J = 3.3 Hz), 131.6 (d, J = 7.7 Hz), 128.6, 120.8, 119.3, 119.0, 115.4 (d, J = 21.0 Hz, 2C), 111.8, 51.7, 47.3, 38.6, 35.2, 33.6, 30.4, 25.4, 21.6, 18.6, 14.6, 11.7, 10.9; **^{19}F NMR** (564.7 MHz, $CDCl_3$) δ –117.0.

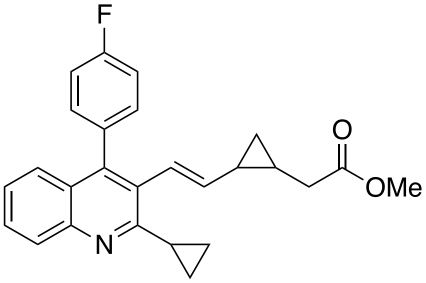

Cyclopropane 24 was prepared according to Method B and allowed to stir for 24 h. The following amounts of reagents were used: substrate **23** (33 mg, 0.061 mmol, 1.0 equiv), Zn^0 (8.0 mg, 0.12 mmol, 2.0 equiv), NaI (74 mg, 0.49 mmol, 8.0 equiv), THF (0.60 mL, 0.10 M in substrate). The compound was purified by flash chromatography (0–20% EtOAc/hexanes) to afford the title compound in a 2:1 mixture of diastereomers as a clear, yellow oil (10. mg, 0.026 mmol, 42%). **TLC R_f** = 0.2 (10% EtOAc/hexanes); **HRMS** (TOF MS ES+) *m/z*: $[M+Na]^+$ calculated for $C_{26}H_{24}FO_2NNa$, 424.1689; found, 424.1671.

Major Diastereomer: ^{1}H NMR (600 MHz, $CDCl_3$) δ 7.99 (d, J = 8.2 Hz, 1H), 7.65–7.59 (m, 1H), 7.37–7.33 (m, 2H), 7.29–7.20 (m, 4H), 6.52 (d, J = 15.3 Hz, 1H), 5.48 (dd, J = 16.0, 8.4 Hz, 1H), 3.73 (s, 3H), 2.18–2.01 (m, 2H), 1.72–1.57 (m, 1H), 1.48–1.42 (m, 1H), 1.41–1.33 (m, 2H), 1.32–1.29 (m, 1H), 1.11–1.05 (m, 2H), 1.05–1.00 (m, 1H), 0.31–0.25 (m, 1H); **^{13}C NMR** (150.9 MHz, $CDCl_3$) δ 173.5, 160.7, 146.5, 143.7, 140.8, 137.0, 131.9 (d, J = 7.7 Hz), 131.7 (d, J = 7.7 Hz), 129.9, 128.9, 128.5, 126.8, 126.3, 126.0, 125.4, 123.4, 115.5 (d, J = 4.4 Hz) , 115.3 (d, J = 4.4 Hz), 51.7, 33.8, 19.3, 16.1, 14.5, 12.4, 10.5, 10.1; **^{19}F NMR** (564.7 MHz, $CDCl_3$) δ –114.5.

Minor Diastereomer: ^{1}H NMR (600 MHz, $CDCl_3$) δ 7.99 (d, J = 8.2 Hz, 1H), 7.65–7.59 (m, 1H), 7.37–7.33 (m, 2H), 7.29–7.20 (m, 4H), 6.42 (d, J = 15.9 Hz, 1H), 5.48 (dd, J = 16.1, 8.7 Hz, 1H), 3.74 (s, 3H), 2.18–2.01 (m, 2H), 1.41–1.33 (m, 2H), 1.32–1.29 (m, 1H), 1.11–1.05 (m, 2H), 1.05–1.00 (m, 1H), 0.97–0.91 (m, 1H), 0.69–0.64 (m, 1H), 0.61–0.56 (m, 1H); **^{13}C NMR** (150.9 MHz, $CDCl_3$) δ 173.5, 160.7, 146.5, 143.7, 140.8, 137.0, 134.0, 132.0 (d, J = 7.7 Hz), 131.9 (d, J = 7.7 Hz), 129.9, 128.9, 128.5, 126.8, 126.3, 126.0, 125.4, 123.4, 115.3 (d, J = 4.4 Hz), 115.2 (d, J = 4.4 Hz), 38.2, 22.0, 16.6, 13.7, 12.4, 10.5, 10.1; **^{19}F NMR** (564.7 MHz, $CDCl_3$) δ –114.5.

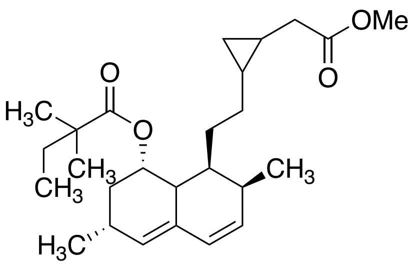

Cyclopropane 26 was prepared according to Method B and allowed to stir for 24 h. The following amounts of reagents were used: substrate **25** (59 mg, 0.098 mmol, 1.0 equiv), Zn^0 (13 mg, 0.20 mmol, 2.0 equiv), NaI (0.12 g, 0.78 mmol, 8.0 equiv), THF (1.0 mL, 0.10 M in substrate). The compound was purified by flash chromatography (0–20% EtOAc/hexanes) to afford the title compound in a 1.6:1 mixture of diastereomers as a clear, colorless oil (19 mg, 0.045 mmol, 46%). **TLC R_f** = 0.7 (20% EtOAc/hexanes); **HRMS** (TOF MS ES+) *m/z*: $[M+Na]^+$ calculated for $C_{26}H_{40}O_4Na$, 439.2824; found, 439.2814.

Major Diastereomer: ^{1}H NMR (600 MHz, $CDCl_3$) δ 6.06–5.99 (m, 1H), 5.86–5.80 (m, 1H), 5.57–5.53 (m, 1H), 5.41–5.34 (m, 1H), 3.74 (s, 3H), 2.53–1.92 (m, 10H), 1.80–1.73 (m, 2H), 1.63–1.58 (m, 2H), 1.21–1.16 (m, 6H), 1.15–1.10 (m, 3H), 0.93–0.86 (m, 6H), 0.85–0.74 (m, 1H), 0.58–0.49 (m, 1H), 0.39–0.31 (m, 2H); **^{13}C NMR** (150.9 MHz, $CDCl_3$) δ 177.8, 174.1, 133.2, 131.9, 129.5, 128.4, 68.1, 51.6, 43.0, 38.9, 37.6, 36.6, 36.4, 34.0, 33.1, 30.6, 28.7, 27.4, 25.7, 24.7, 23.0, 18.8, 15.8, 13.8, 11.7, 9.3.

Minor Diastereomer: ^{1}H NMR (600 MHz, $CDCl_3$) δ 6.06–5.99 (m, 1H), 5.86–5.80 (m, 1H), 5.57–5.53 (m, 1H), 5.41–5.34 (m, 1H), 3.74 (s, 3H), 2.53–1.92 (m, 10H), 1.80–1.73 (m, 2H), 1.63–1.58 (m, 2H), 1.21–1.16 (m, 6H), 1.15–1.10 (m, 3H), 1.08–1.03 (m, 1H), 0.93–0.86 (m, 6H), 0.85–0.74 (m, 2H), –0.09 to –0.16 (m, 1H); **^{13}C NMR** (150.9 MHz, $CDCl_3$) δ 177.8, 174.1, 133.2, 131.9, 129.5, 128.4, 68.1, 51.6, 43.0, 38.9, 37.6, 36.6, 36.4, 34.0, 33.1, 30.6, 28.7, 27.4, 25.7, 24.7, 23.0, 18.8, 15.8, 13.8, 11.7, 9.3.

C. Characterization of 1,3-Dimesylate Substrates

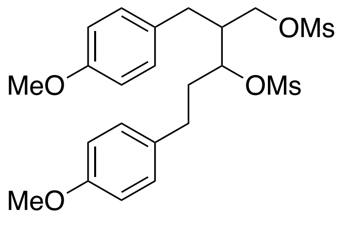

16

1,3-Dimesylate 1 was prepared by was charging a flame-dried flask with $LiAlH_4$ (2.2 equiv) in a glovebox. The flask was capped with a stopper and removed from glovebox. An N_2 inlet and anhydrous Et_2O (25 mL, 0.4 M in substrate) were added. The reaction flask was cooled to 0 ºC and the prerequisite beta-keto ester (11 mmol, 1.0 equiv) was added as a solution in Et_2O (11 mL, 1.0 M). The reaction was warmed to rt and stirred for 2 h. To quench, saturated NH_4Cl was added and reaction was extracted with EtOAc (x3). The combined organic layers were washed with brine, dried over Na_2SO_4, and concentrated in vacuo. The diol was carried into the next step without

further purification. The unpurified diol (1.1 g, 3.4 mmol, 1.0 equiv) and DMAP (83 mg, 0.68 mmol, 0.20 equiv) in DCM (10. mL, 0.34 M substrate) under Schlenk conditions. Et_3N (1.4 mL, 10. mmol, 3.0 equiv) and MsCl (0.58 mL, 7.5 mL, 2.2 equiv) were added sequentially to the flask and allowed to stir overnight. The resulting solution was quenched with $NaHCO_3$, extracted with DCM (x3), washed with brine, dried with $MgSO_4$, filtered, and concentrated in vacuo. The compound was purified by flash column chromatography (0–60% EtOAc/hexanes) to afford the title compound as a clear, light-yellow oil (1.5 g, 3.1 mmol, 92% over two steps). The compound was characterized as a 3:1 mixture of diastereomers. **TLC R_f =** 0.6 (50% EtOAc/hexanes). Analytical data is consistent with literature values.[i]

Major Diastereomer: ^{1}H NMR (400 MHz, $CDCl_3$) δ 7.11–7.06 (m, 2H), 7.00 (d, *J* = 8.6 Hz, 2H), 6.83 (t, *J* = 8.5 Hz, 4H), 4.95–4.88 (m, 1H), 4.21–4.14 (m, 2H), 3.79–3.88 (m, 6H), 2.97 (s, 3H), 2.85–2.52 (m, 4H), 2.50–2.41 (m, 4H), 2.18–1.97 (m, 2H);

Minor Diastereomer: ^{1}H NMR (400 MHz, $CDCl_3$) δ 7.11–7.06 (m, 2H), 7.00 (d, *J* = 8.6 Hz, 2H), 6.83 (t, *J* = 8.5 Hz, 4H), 4.95–4.88 (m, 1H), 4.21–4.14 (m, 2H), 3.79–3.88 (m, 6H), 3.04 (s, 3H), 2.95 (s, 3H), 2.85–2.52 (m, 4H), 2.50–2.41 (m, 1H), 2.18–1.97 (m, 2H).

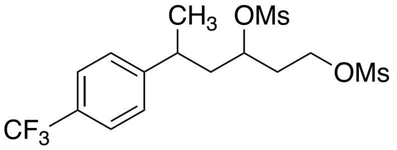

1,3-Dimesylate SI-1 was prepared by dissolving the prerequisite diol (1.17 g, 4.48 mmol, 1.00 equiv) and DMAP (109 mg, 0.896 mmol, 0.200 equiv) in DCM (9 mL, 0.5 M in substrate) under Schlenk conditions. Et_3N (1.87 mL, 13.4 mmol, 3.00 equiv) and MsCl (0.86 mL, 11 mmol, 2.5 equiv) were added sequentially to the flask and allowed to stir overnight. The resulting solution was quenched with $NaHCO_3$, extracted with DCM (x3), washed with brine, dried with $MgSO_4$, filtered, and concentrated in vacuo. The compound was purified by flash column chromatography (50% EtOAc/hexanes) to afford the title compound in a 1:1 mixture of diastereomers as a viscous oil (0.92 mg, 2.2 mmol, 49% yield). **TLC R_f =** 0.6 (50% EtOAc/hexanes); **^{1}H NMR** (500 MHz, $CDCl_3$) *δ* 7.58 (d, *J* = 7.9 Hz, 4H, both diastereomers), 7.35 (t, *J* = 8.8 Hz, 4H, both diastereomers), 4.77–4.74 (m, 1H, one diastereomer), 4.71–4.67 (m, 1H, other diastereomer), 4.34–4.26 (m, 4H, both diastereomers), 3.03–3.00 (m, 2H, both diastereomers), 2.99–2.95 (m, 12H, both diastereomers), 2.23–1.92 (m, 8H, both diastereomers), 1.33 (d, *J* = 6.9 Hz, 3H, one diastereomer), 1.30 (d, *J* = 7.0 Hz, 3H, other diastereomer); **^{13}C NMR** (500 MHz, $CDCl_3$) *δ* 149.7 (one diastereomer), 149.6 (other diastereomer), 129.3–128.5 (q, *J* = 32.3 Hz, 2C, both diastereomers), 127.4–120.6 (q, *J* = 271.8 Hz, 2C, both diastereomers), 127.6 (2C, one diastereomer), 127.3 (2C, other diastereomer), 125.8–125.6 (quint, *J* = 3.7 Hz, 4C, both diastereomers), 77.1 (one diastereomer), 76.8 (other diastereomer), 65.4 (one diastereomer), 65.3 (other diastereomer), 42.9 (one diastereomer), 42.6 (other diastereomer), 38.7 (one diastereomer), 38.5 (other diastereomer), 37.43 (one diastereomer), 37.40 (other diastereomer), 36.2 (one diastereomer), 36.0 (other diastereomer), 34.5 (one diastereomer), 34.2 (other diastereomer), 22.9 (one diastereomer), 22.4 (other diastereomer); **^{19}F NMR** (564.6 MHz, $CDCl_3$) δ –62.3; **HRMS** (TOF MS ES+) m/z: $[M+Na]^+$ calculated for $C_{15}H_{21}F_3O_6S_2Na$, 441.0629; found, 441.0621.

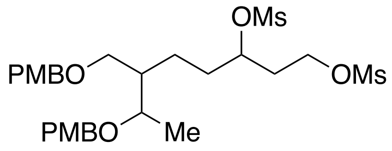

1,3-Dimesylate SI-2 was prepared by dissolving the prerequisite diol (1.0 g, 2.4 mmol, 1.0 equiv) and DMAP (0.59 g, 0.48 mmol, 0.20 equiv) in a solution of DCM (10. mL, 0.24 M in substrate) under Schlenk conditions. Et_3N (0.74 mL, 5.3 mmol, 2.2 equiv) followed by MsCl (0.41 mL, 5.3 mmol, 2.2 equiv) were added sequentially to the flask and allowed to stir overnight. The resulting solution was quenched with $NaHCO_3$, extracted with DCM (x3), washed with brine, dried with $MgSO_4$, filtered and concentrated in vacuo. The compound was purified by flash chromatography (0–60% EtOAc/hexanes) to afford the title compound in a 1:1:1:1 mixture of 4 diastereomers as a clear, colorless oil (1.2 g, 2.0 mmol, 84%). **TLC** $\mathbf{R_f}$ = 0.6 (60% EtOAc/hexanes); ^{1}**H NMR** (400 MHz, $CDCl_3$) δ 7.23 (d, J = 8.3 Hz, 4H), 6.91–6.83 (m, 4H), 4.88–4.78 (m, 1H), 4.54–4.26 (m, 6H), 3.81 (s, 6H), 3.67–3.56 (m, 1H), 3.53–3.35 (m, 2H), 3.02 (s, 3H), 2.94 (s, 3H), 2.15–1.97 (m, 2H), 1.87–1.65 (m, 3H), 1.64–1.35 (m, 2H), 1.16 (at, J = 7.2 Hz, 3H). Analytical data is consistent with literature values.[i]

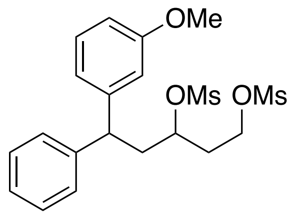

1,3-Dimesylate SI-3 was prepared by dissolving the prerequisite diol (0.65 g, 2.4 mmol, 1.0 equiv) and DMAP (59 mg, 0.50 mmol, 0.20 equiv) in DCM (5 mL, 0.5 M in substrate) under Schlenk conditions. Et_3N (0.80 mL, 5.8 mmol, 2.4 equiv) and MsCl (0.45 mL, 5.8 mmol, 2.4 equiv) were added sequentially to the flask and allowed to stir overnight. The resulting solution was quenched with $NaHCO_3$, extracted with DCM (x3), washed with brine, dried with $MgSO_4$, filtered, and concentrated in vacuo. The compound was purified by column chromatography (0–50% EtOAc/hexanes) to afford a clear oil (0.54 g, 1.2 mmol, 48% yield). **TLC** $\mathbf{R_f}$ = 0.4 (50% EtOAc/hexanes); ^{1}**H NMR** (500 MHz, $CDCl_3$) δ 7.32–7.18 (m, 6H), 6.85–6.72 (m, 3H), 4.78–4.75 (m, 1H), 4.35– 4.29 (m, 2H), 4.10–4.05 (m, 1H), 3.77 (s, 3H), 2.93 (s, 3H), 2.89 (s, 3H), 2.62–2.56 (m, 1H), 2.45–2.39 (m, 1H), 2.19–2.11 (m, 2H). Analytical data is consistent with literature values.[i]

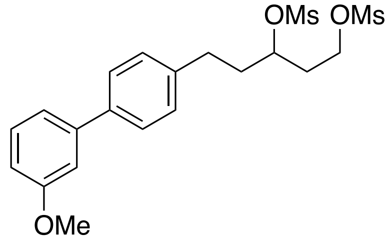

1,3-Dimesylate SI-4 was prepared by dissolving the prerequisite diol (0.70 g, 2.4 mmol, 1.0 equiv) and DMAP (60. mg, 0.49 mmol, 0.20 equiv) in DCM (10. mL, 0.24 M in substrate) under Schlenk conditions. Et_3N (1.0 mL, 7.3 mmol, 3.0 equiv) and MsCl (0.42 mL, 5.4 mmol, 2.2 equiv) were

added sequentially to the flask and allowed to stir overnight. The resulting solution was quenched with $NaHCO_3$, extracted with DCM (x3), washed with brine, dried with $MgSO_4$, filtered, and concentrated in vacuo. The compound was purified by flash column chromatography (0–50% EtOAc/hexanes) to afford the title compound as a white solid (0.93 g, 2.1 mmol, 86%). **TLC R_f =** 0.7 (50% EtOAc/hexanes); **^{1}H NMR** (500 MHz, $CDCl_3$) δ 7.53 (d, *J* = 7.8 Hz, 2H), 7.34 (t, *J* = 8.0 Hz, 1H), 7.26 (d, *J* = 8.2 Hz, 2H), 7.16 (d, *J* = 7.5 Hz, 1H), 7.10 (s, 1H), 6.88 (d, *J* = 8.7 Hz, 1H), 4.97–4.93 (m, 1H), 4.40–4.33 (m, 2H), 3.85 (s, 3H), 3.04 (s, 3H), 3.03 (s, 3H), 2.82–2.74 (m, 2H), 2.24–2.05 (m, 4H). Analytical data is consistent with literature values.[i]

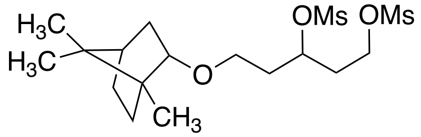

1,3-Dimesylate 16 was prepared by dissolving the prerequisite diol (1.4 g, 5.6 mmol, 1.0 equiv) and DMAP (130 mg, 1.1 mmol, 0.20 equiv) in DCM (20 mL, 0.3 M in substrate). Et_3N (1.9 mL, 13 mmol, 2.4 equiv) and MsCl (1.0 mL, 13 mmol, 2.4 equiv) were added sequentially to the flask and allowed to stir overnight. The resulting solution was quenched with $NaHCO_3$, extracted with DCM (x3), washed with brine, dried with $MgSO_4$, filtered, and concentrated in vacuo. The compound was purified by flash column chromatography (0–50% EtOAc/hexanes) to afford the title compound in 1:1 mixture of diastereomers as a clear yellow oil (1.3 g, 3.3 mmol, 59% yield). **TLC R_f** = 0.7 (50% EtOAc/hexanes); **^{1}H NMR** (400 MHz, $CDCl_3$) δ 5.06–4.99 (m, 2H, both diastereomers), 4.39–4.35 (m, 4H, both diastereomers), 3.62–3.53 (m, 4H, both diastereomers), 3.46–3.41 (m, 2H, both diastereomers), 3.07 (s, 3H, one diastereomer), 3.06 (s, 3H, other diastereomer), 3.05 (s, 6H, both diastereomers), 2.31–1.86 (m, 12H, both diastereomers), 1.75–1.62 (m, 4H, both diastereomers), 1.25–1.15 (m, 4H, both diastereomers), 0.99 (td, *J* = 1.0, 0.9 Hz, 2H, both diastereomers), 0.86 (s, 6H, both diastereomers), 0.84 (s, 12H, both diastereomers). Analytical data is consistent with literature values.[i]

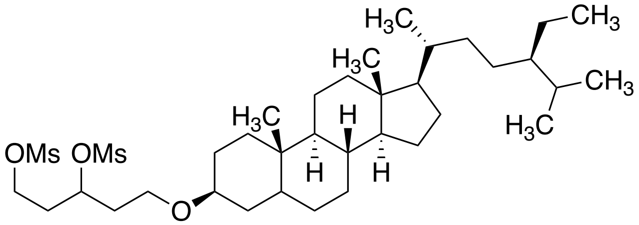

1,3-Dimesylate SI-5 was prepared by dissolving the prerequisite diol (0.77 g, 1.5 mmol, 1.0 equiv) and DMAP (37 mg, 0.30 mmol, 0.20 equiv) in DCM (8 mL, 0.2 M in substrate). Et_3N (0.51 mL, 3.6 mmol, 2.4 equiv) and MsCl (0.28 mL, 3.6 mmol, 2.4 equiv) were added sequentially to the flask and allowed to stir overnight. The resulting solution was quenched with $NaHCO_3$, extracted with DCM (x3), washed with brine, dried with $MgSO_4$, filtered, and concentrated in vacuo. The compound was purified by flash column chromatography (50% EtOAc/hexanes) to afford the title compound as a white waxy solid (0.44 g, 0.65 mmol, 43% yield). The compound was characterized as a 1:1 mixture of diastereomers. **TLC R_f** = 0.8 (10% EtOAc/hexanes); **^{1}H NMR** (400 MHz, $CDCl_3$) δ 5.09–5.05 (m, 1H), 4.38–4.35 (m, 2H), 3.58–3.55 (m, 2H), 3.31–3.24 (asept, *J* = 5.0 Hz,

1H), 3.06 (s, 3H), 3.04 (s, 3H), 2.15–0.64 (m, 54H). Analytical data is consistent with literature values.[i]

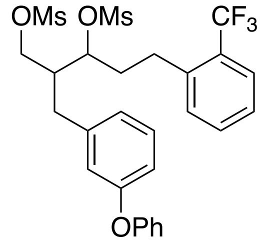

1,3-Dimesylate SI-6 was prepared by dissolving substrate (0.47 g, 1.1 mmol, 1.0 equiv) in a solution of DMAP (26 mg, 0.21 mmol, 0.20 equiv) in DCM (6.0 mL, 0.18 M in substrate) under Schlenk conditions. Et_3N (0.45 mL, 3.3 mmol, 3.0 equiv) followed by MsCl (0.19 mL, 2.4 mmol, 2.2 equiv) were added sequentially to the flask and allowed to stir overnight. The resulting solution was quenched with $NaHCO_3$, extracted with DCM (x3), washed with brine, dried with $MgSO_4$, filtered and concentrated in vacuo. The compound was purified by flash chromatography (0–50% EtOAc/hexanes) to afford the title compound in a 1.1:1 mixture of diastereomers as a clear, colorless oil (0.41 g, 0.73 mmol, 66%). **TLC R_f** = 0.6 (40% EtOAc/hexanes). Analytical data is consistent with literature values.[i]

Major Diastereomer: ^{1}H NMR (500 MHz, $CDCl_3$) δ 7.64 (t, *J* = 7.7 Hz, 1H), 7.49 (t, *J* = 7.6 Hz, 1H), 7.37–7.24 (m, 5H), 7.11 (t, *J* = 7.4 Hz, 1H), 7.04–6.95 (m, 2H), 6.92–6.84 (m, 2H), 6.82 (s, 1H), 5.05–4.95 (m, 1H), 4.26–4.08 (m, 2H), 3.09 (s, 2H), 3.03–2.78 (m, 6H), 2.66–2.56 (m, 1H), 2.54–2.45 (m, 1H), 2.18–1.98 (m, 3H).

Minor Diastereomer: ^{1}H NMR (500 MHz, $CDCl_3$) δ 7.64 (t, *J* = 7.7 Hz, 1H), 7.49 (t, *J* = 7.6 Hz, 1H), 7.37–7.24 (m, 5H), 7.11 (t, *J* = 7.4 Hz, 1H), 7.04–6.95 (m, 2H), 6.92–6.84 (m, 2H), 6.82 (s, 1H), 5.05–4.95 (m, 1H),4.26–4.08 (m, 2H), 3.09 (s, 2H), 3.03–2.78 (m, 6H), 2.66–2.56 (m, 1H), 2.54–2.45 (m, 1H), 2.18–1.98 (m, 3H).

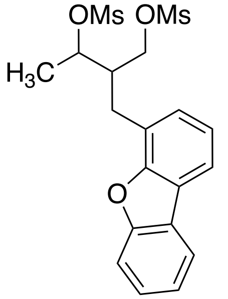

1,3-Dimesylate SI-7 was prepared by dissolving the prerequisite diol (0.306 g, 1.13 mmol, 1.00 equiv) and DMAP (28 mg, 0.23 mmol, 0.20 equiv) in DCM (6 mL, 0.2 M in substrate). Et_3N (0.38 mL, 2.7 mmol, 2.4 equiv) and MsCl (0.21 mL, 2.7 mmol, 2.4 equiv) were added sequentially to the flask and allowed to stir overnight. The resulting solution was quenched with $NaHCO_3$, extracted with DCM (x3), washed with brine, dried with $MgSO_4$, filtered, and concentrated in vacuo. The oil was purified by column chromatography (0–50% EtOAc/hexanes) to afford the title

compound in a 1:1 mixture of diastereomers as a clear oil (0.37 g, 0.86 mmol, 76% yield). **TLC R_f =** 0.6 (50% EtOAc/hexanes); **^{1}H NMR** (400 MHz, $CDCl_3$) δ 7.95 (d, *J* = 7.7 Hz, 2H, both diastereomers), 7.86 (d, *J* = 6.4 Hz, 2H, both diastereomers), 7.57 (d, J = 8.4 Hz, 2H, both diastereomers), 7.49–7.45 (m, 2H, both diastereomers), 7.39–7.28 (m, 5H, both diastereomers), 7.19–7.16 (m, 1H, one diastereomer), 5.17–5.12 (m, 1H, one diastereomer), 5.05 (quint, *J* = 6.3 Hz, 1H, other diastereomer), 4.29 (dd, *J* = 10.4, 4.1 Hz, 1H, one diastereomer), 4.25–4.21 (m, 2H, other diastereomer), 4.17 (dd, *J* = 10.2, 5.0 Hz, 1H, other diastereomer), 3.35 (dd, *J* = 14.5, 4.9 Hz, 1H, one diastereomer), 3.25 (dd, *J* = 13.9, 5.5 Hz, 1H, one diastereomer), 3.09 (s, 3H, one diastereomer), 3.08 (s, 3H, other diastereomer), 3.06–2.98 (m, 1H, other diastereomer), 2.97 (s, 3H, one diastereomer), 2.96 (s, 3H, other diastereomer), 2.95–2.92 (m, 1H, other diastereomer), 2.73–2.65 (m, 2H, one diastereomer), 1.66 (d, *J* = 6.5 Hz, 6H, both diastereomers). Analytical data is consistent with literature values.[i]

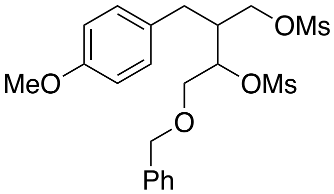

1,3-Dimesylate SI-8 was prepared by dissolving the prerequisite diol (240 mg, 0.77 mmol, 1.0 equiv) and DMAP (18 mg, 0.15 mmol, 0.20 equiv) in DCM (4 mL, 0.4 M in substrate) under Schlenk conditions. Et_3N (0.26 mL, 1.9 mmol, 2.4 equiv) and MsCl (0.14 mL, 1.9 mmol, 2.4 equiv) were added sequentially to the flask and allowed to stir overnight. The resulting solution was quenched with $NaHCO_3$, extracted with DCM (x3), washed with brine, dried with $MgSO_4$, filtered, and concentrated in vacuo. The compound was purified by flash column chromatography (50% EtOAc/hexanes) to afford the title compound in a 3:1 mixture of diastereomers as a viscous oil (0.30 g, 0.63 mmol, 82% yield). **TLC R_f =** 0.5 (50% EtOAc/hexanes); **HRMS** (TOF MS ES+) m/z: $[M+Na]^+$ calculated for $C_{21}H_{28}S_2O_8Na$, 495.1123; found, 495.1112.

Major Diastereomer: ^{1}H NMR (400 MHz, $CDCl_3$) δ 7.39–7.36 (m, 5H), 7.12 (d, *J* = 8.6 Hz, 2H), 6.91–6.88 (m, 2H), 5.09–5.06 (m, 1H), 4.65–4.56 (m, 2H), 4.26–4.15 (m, 2H), 3.84 (s, 3H), 3.82–3.77 (m, 2H), 3.10 (s, 3H), 3.02 (s, 3H), 2.88 (dd, *J* = 14.2, 5.5 Hz, 1H), 2.69–2.61 (m, 1H), 2.55–2.46 (m, 1H); **^{13}C NMR** (125 MHz, $CDCl_3$) *δ* 158.5, 137.2, 129.9 (2C), 129.6, 128.6 (2C), 128.2, 128.1 (2C), 114.3 (2C), 80.7, 73.6, 69.7, 67.9, 55.3, 42.2, 38.7, 37.2, 31.2.

Minor Diastereomer: ^{1}H NMR (400 MHz, $CDCl_3$) δ 7.44–7.41 (m, 5H), 7.17 (d, *J* = 8.6 Hz, 2H), 6.91–6.88 (m, 2H), 5.00–4.97 (m, 1H), 4.65–4.56 (m, 2H), 4.26–4.15 (m, 2H), 3.84 (s, 3H), 3.71–3.68 (m, 2H), 3.10 (s, 3H), 3.01 (s, 3H), 2.82 (dd, *J* = 13.8, 5.8 Hz, 1H), 2.69–2.61 (m, 1H), 2.55–2.46 (m, 1H); **^{13}C NMR** (125 MHz, $CDCl_3$) *δ* 158.5, 137.1, 130.2 (2C), 129.6, 128.6 (2C), 128.2, 128.0 (2C), 114.3 (2C), 80.8, 73.5, 69.9, 67.4, 55.3, 42.1, 38.9, 37.2, 32.3.

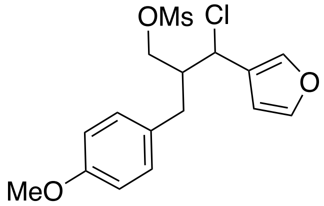

1,3-Dimesylate SI-9 was prepared by dissolving the prerequisite diol (0.58 g, 2.2 mmol, 1.0 equiv) and DMAP (54 mg, 0.44 mmol, 0.20 equiv) in DCM (10 mL, 0.2 M in substrate). Et_3N (0.74 mL, 5.3 mmol, 2.4 equiv) and MsCl (0.41 mL, 5.3 mmol, 2.4 equiv) were added sequentially to the flask and allowed to stir overnight. The resulting solution was quenched with $NaHCO_3$, extracted with DCM (x3), washed with brine, dried with $MgSO_4$, filtered, and concentrated in vacuo. The oil was purified by column chromatography (0–50% EtOAc/hexanes) to afford the title compound as a 1:1 mixture of diastereomers as a clear oil (0.32 g, 0.89 mmol, 40% yield). **TLC R_f =** 0.7 (50% EtOAc/hexanes); **^{1}H NMR** (400 MHz, $CDCl_3$) δ 7.50–7.49 (m, 2H, both diastereomers), 7.46–7.44 (m, 2H, both diastereomers), 7.08 (d, *J* = 8.8 Hz, 2H, both diastereomers), 7.06 (d, *J* = 8.6 Hz, 2H, both diastereomers), 6.85–6.83 (m, 4H, both diastereomers), 6.44 (s, 2H, both diastereomers), 5.15 (d, *J* = 5.1 Hz, 1H, one diastereomer), 4.99 (d, *J* = 6.9 Hz, 1H, other diastereomer), 4.39 (dd, *J* = 9.8, 4.6 Hz, 1H, one diastereomer), 4.22–4.18 (m, 2H, both diastereomers), 4.03 (dd, *J* = 10.0, 4.5 Hz, 1H, other diastereomer), 3.78 (s, 6H, both diastereomers), 3.01–2.98 (m, 1H, one diastereomer), 2.97 (s, 3H, both diastereomers), 2.94 (s, 3H, both diastereomers), 2.76 (dd, *J* = 13.9, 5.9 Hz, 1H, other diastereomer), 2.64–2.57 (m, 2H, both diastereomers), 2.56–2.45 (m, 2H, both diastereomers). Analytical data is consistent with literature values.[i]

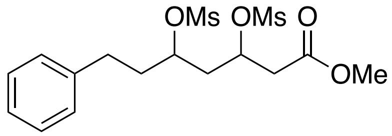

1,3-Dimesylate SI-10 was prepared by dissolving the prerequisite diol (0.49 g, 2.0 mmol, 1.0 equiv) in a solution of DMAP (48 mg, 0.39 mmol, 0.20 equiv) in DCM (10. mL, 0.20 M in substrate) under Schlenk conditions. Et_3N (0.81 mL, 5.9 mmol, 3.0 equiv) followed by MsCl (0.33 mL, 4.3 mmol, 2.2 equiv) were added sequentially to the flask and allowed to stir for 1h. The resulting solution was quenched with $NaHCO_3$, extracted with DCM (x3), washed with brine, dried with $MgSO_4$, filtered and concentrated in vacuo. The compound was purified as a by flash chromatography (0–60% EtOAc/hexanes) to afford the title compound in a 3:1 mixture of diastereomers as a clear, yellow oil (0.33 g, 0.80 mmol, 41%). **TLC R_f** = 0.3 (50% EtOAc/hexanes); **^{1}H NMR** (600 MHz, $CDCl_3$) δ 7.32–7.28 (m, 2H), 7.24–7.18 (m, 3H), 5.19–5.13 (m, 1H), 4.88–4.82 (m, 1H), 3.71 (s, 3H), 3.15–3.02 (m, 6H), 3.08–3.02 (m, 1H), 2.85–2.72 (m, 3H), 2.42–2.36 (m, 1H), 2.22–2.08 (m, 3H); **^{13}C NMR** (150.9 MHz, $CDCl_3$) δ 170.0 (2C, both), 140.4 (2C, both), 128.7 (4C, both), 128.5 (4C, both), 126.4 (2C, both), 78.0 (2C, both), 75.0 (2C, both), 60.4 (2C, both), 52.2 (2C, both), 39.8 (one diastereomer), 38.7 (other diastereomer), 38.8 (one diastereomer), 38.5 (other diastereomer), 36.8 (one diastereomer), 36.1 (other diastereomer), 31.6 (one diastereomer), 31.1 (other diastereomer), 21.1 (one diastereomer), 14.2 (other diastereomer); **HRMS** (TOF MS ES+) *m/z*: $[M+Na]^+$ calculated for $C_{16}H_{24}O_8S_2Na$: 431.0810; found, 431.0821.

Major Diastereomer: ^{1}H NMR (600 MHz, $CDCl_3$) δ 7.32–7.28 (m, 2H), 7.24–7.18 (m, 3H), 5.19–5.13 (m, 1H), 4.88–4.82 (m, 1H), 3.71 (s, 3H), 3.15–3.02 (m, 6H), 3.08–3.02 (m, 1H), 2.85–2.72 (m, 3H), 2.42–2.36 (m, 1H), 2.22–2.08 (m, 3H); **^{13}C NMR** (150.9 MHz, $CDCl_3$) δ 170.0, 140.4, 128.7 (2C), 128.5 (2C), 126.4, 78.0, 75.0, 60.4, 52.2, 38.7, 38.5, 36.1, 31.1, 14.2.

Minor Diastereomer: **^{1}H NMR** (600 MHz, $CDCl_3$) δ 7.32–7.28 (m, 2H), 7.24–7.18 (m, 3H), 5.19–5.13 (m, 1H), 4.88–4.82 (m, 1H), 3.71 (s, 3H), 3.15–3.02 (m, 6H), 3.08–3.02 (m, 1H), 2.85–2.72 (m, 3H), 2.42–2.36 (m, 1H), 2.22–2.08 (m, 3H); **^{13}C NMR** (150.9 MHz, $CDCl_3$) δ 170.0, 140.4, 128.7 (2C), 128.5 (2C), 126.4, 78.0, 75.0, 60.4, 52.2, 39.8, 38.8, 36.8, 31.6, 21.1.

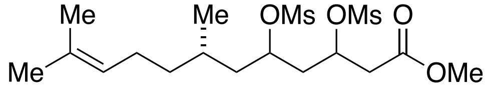

1,3-Dimesylate SI-11 was prepared by dissolving the prerequisite diol (0.23 g, 0.83 mmol, 1.0 equiv) in a solution of DMAP (21 mg, 0.17 mmol, 0.20 equiv) in DCM (6.0 mL, 0.14 M in substrate) under Schlenk conditions. Et_3N (0.34 mL, 2.5 mmol, 3.0 equiv) followed by MsCl (0.16 mL, 2.1 mmol, 2.5 equiv) were added sequentially to the flask and allowed to stir for 1h. The resulting solution was quenched with $NaHCO_3$, extracted with DCM (x3), washed with brine, dried with $MgSO_4$, filtered and concentrated in vacuo. The compound was purified by flash chromatography (0–60% EtOAc/hexanes) to afford the title compound in a 1:1 mixture of diastereomers as a clear, yellow oil (0.11 g, 0.24 mmol, 12%). **TLC R$_f$** = 0.6 (60% EtOAc/hexanes); **^{1}H NMR** (600 MHz, $CDCl_3$) δ 5.26–5.20 (m, 1H), 5.17–5.11 (m, 1H), 4.98–4.91 (m, 1H), 3.78 (s, 3H), 3.21–3.08 (m, 6H), 2.90–2.87 (m, 2H), 2.46–2.34 (m, 1H), 2.26–2.16 (m, 1H), 2.09–1.98 (m, 2H), 1.90–1.84 (m, 1H), 1.74 (s, 3H), 1.66 (s, 3H), 1.56–1.33 (m, 3H), 1.30–1.18 (m, 1H), 1.04–0.99 (m, 3H); **^{13}C NMR** (150.9 MHz, $CDCl_3$) δ 170.0 (2C, both), 131.7 (2C, both), 124.3 (2C, both), 77.2 (2C, both), 75.2 (2C, both), 52.2 (2C, both), 42.1 (2C, both), 40.8 (2C, both), 40.0 (2C, both), 38.8 (2C, both), 38.6 (2C, both), 37.2 (one diastereomer), 36.5 (other diastereomer), 28.8 (2C, both), 25.8 (2C, both), 25.3 (2C, both), 19.6 (other diastereomer), 19.1 (one diastereomer), 17.7 (2C, both); **HRMS** (TOF MS ES+) *m/z*: $[M+NH_4]^+$ calculated for $C_{17}H_{32}O_8S_2NH_4$, 446.1882; found, 446.1874.

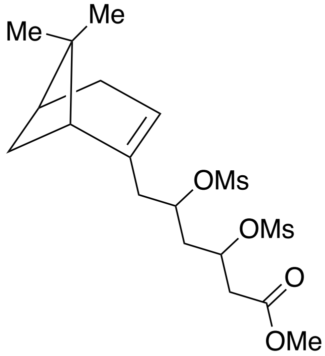

1,3-Dimesylate SI-12 was prepared by dissolving the prerequisite diol (0.35 g, 1.2 mmol, 1.0 equiv) in a solution of DMAP (31 mg, 0.25 mmol, 0.20 equiv) in DCM (10. mL, 0.12 M in substrate) under Schlenk conditions. Et_3N (0.51 mL, 3.7 mmol, 3.0 equiv) followed by MsCl (0.21 mL, 2.7 mmol, 2.2 equiv) were added sequentially to the flask and allowed to stir for 1h. The resulting solution was quenched with $NaHCO_3$, extracted with DCM (x3), washed with brine, dried with $MgSO_4$, filtered and concentrated in vacuo. The compound was purified by flash chromatography (0–60% EtOAc/hexanes) to afford the title compound in a 1:1 mixture of diastereomers as a clear, yellow oil (0.18 g, 0.41 mmol, 33%). **TLC R$_f$** = 0.6 (60% EtOAc/hexanes); **^{1}H NMR** (600 MHz, $CDCl_3$) δ 5.41–5.37 (m, 1H), 5.19–5.13 (m, 1H), 4.84–4.74 (m, 1H), 3.72 (s, 3H), 3.10–3.04 (m, 6H), 2.83 (ddd, *J* = 16.7, 8.8, 4.4 Hz, 1H), 2.76 (ddd, *J* = 16.4, 8.1, 3.1 Hz, 1H), 2.58–2.48 (m, 1H), 2.43–2.19 (m, 6H), 2.12–2.07 (m, 2H), 1.29 (s, 3H),

1.13 (dd, J =33.9, 8.8 Hz, 1H), 0.84 (s, 3H); **^{13}C NMR** (150.9 MHz, $CDCl_3$) δ 170.1 (2C, both), 142.2 (2C, both), 121.9 (2C, both), 76.6 (2C, both), 75.6 (2C, both), 52.2 (2C, both), 45.9 (one diastereomer), 45.7 (other diastereomer), 42.9 (one diastereomer), 42.6 (other diastereomer), 40.5 (2C, both), 39.6 (one diastereomer), 39.0 (other diastereomer), 38.9 (2C, both), 38.6 (2C, both), 38.4 (2C, both), 38.1 (2C, both), 31.7 (2C, both), 31.5 (2C, both), 26.2 (2C, both), 21.4 (one diastereomer), 21.1 (other diastereomer); **HRMS** (TOF MS ES+) *m/z*: $[M+NH_4]^+$ calculated for $C_{18}H_{30}O_8S_2NH_4$, 456.1726; found, 456.1713.

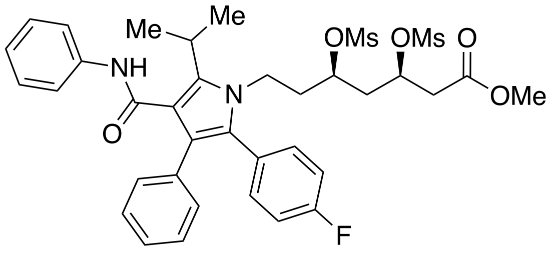

1,3-Dimesylate 17 was prepared by dissolving the prerequisite diol (0.95 g, 1.7 mmol, 1.0 equiv) in a solution of DMAP (40. mg, 0.33 mmol, 0.20 equiv) in DCM (10. mL, 0.17 M in substrate) under Schlenk conditions. Et_3N (0.70 mL, 5.0 mmol, 3.0 equiv) followed by MsCl (0.29 mL, 3.7 mmol, 2.2 equiv) were added sequentially to the flask and allowed to stir for 1h. The resulting solution was quenched with $NaHCO_3$, extracted with DCM (x3), washed with brine, dried with $MgSO_4$, filtered and concentrated in vacuo. The compound was purified by flash chromatography (0–60% EtOAc/hexanes) to afford the title compound as a white foam (0.86 g, 1.2 mmol, 71%). **TLC R_f** = 0.6 (60% EtOAc/hexanes); **^{1}H NMR** (600 MHz, $CDCl_3$) δ 7.30–7.18 (m, 9H), 7.13 (d, J = 8.1 Hz, 2H), 7.08 (t, J = 8.5 Hz, 2H), 7.03 (t, J = 7.4 Hz, 1H), 6.94 (s, 1H), 5.03–4.97 (m, 1H), 4.79–4.73 (m, 1H), 4.19–4.01 (m, 2H), 3.75 (s, 3H), 3.65–3.57 (m, 1H), 3.09 (s, 3H), 2.99 (s, 3H), 2.77 (ddd, J = 58.3, 16.9, 7.8 Hz, 2H), 2.28–2.21 (m, 1H), 2.16–2.09 (m, 1H), 2.02–1.94 (m, 2H), 1.60 (t, J = 6.0 Hz, 6H); **^{13}C NMR** (150.9 MHz, $CDCl_3$) δ 169.8, 164.7, 162.2 (d, J = 248.2 Hz), 141.4, 138.4, 134.4, 133.4 (d, J = 8.3 Hz, 2C), 130.5 (2C), 128.7 (2C), 128.7 (2C), 128.4, 128.0 (d, J = 3.9 Hz), 126.7, 123.6, 122.1, 119.6, 115.7 (d, J = 21.6 Hz, 2C), 75.0, 74.2, 52.2, 40.5, 39.7, 38.6, 38.5 (4C), 35.9, 26.2, 21.8 (2C); **^{19}F NMR** (564.7 MHz, $CDCl_3$) δ –113.2; **HRMS** (TOF MS ES+) *m/z*: $[M+H]^+$ calculated for $C_{36}H_{41}FO_9N_2S_2H$: 729.2316; found, 729.2314.

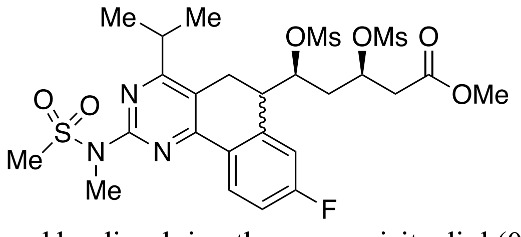

1,3-Dimesylate 19 was prepared by dissolving the prerequisite diol (0.11 g, 0.23 mmol, 1.0 equiv) in a solution of DMAP (6.0 mg, 0.046 mmol, 0.20 equiv) in DCM (2.0 mL, 0.11 M in substrate) under Schlenk conditions. Et_3N (0.10 mL, 0.69 mmol, 3.0 equiv) followed by MsCl (40. µL, 0.51 mmol, 2.2 equiv) were added sequentially to the flask and allowed to stir for 1h. The resulting solution was quenched with $NaHCO_3$, extracted with DCM (x3), washed with brine, dried with $MgSO_4$, filtered and concentrated in vacuo. The compound was purified by flash chromatography (0–60% EtOAc/hexanes) to afford the title compound in a 1:1 mixture of diastereomers as a clear,

yellow oil (86 mg, 0.13 mmol, 57%). **TLC R$_f$** = 0.6 (60% EtOAc/hexanes); **^{1}H NMR** (600 MHz, $CDCl_3$) δ 8.45–8.36 (m, 2H, both), 7.25–7.11 (m, 4H, both), 5.29–5.23 (m, 1H, one diastereomer), 5.15–5.09 (m, 1H, other diastereomer), 4.90–4.85 (m, 1H, other diastereomer), 4.84–4.79 (m, 1H, one diastereomer), 3.77 (s, 3H, one diastereomer), 3.75 (s, 3H, other diastereomer), 3.66 (s, 3H, one diastereomer), 3.65 (s, 3H, other diastereomer), 3.61 (s, 6H, both), 3.57–3.53 (m, 2H, both), 3.48–3.35 (m, 4H, both), 3.21–3.04 (m, 4H, both), 3.12 (s, 6H, both), 2.84–2.78 (m, 4H, both), 2.73–2.67 (m, 2H, both), 2.60 (s, 2H, both), 2.32–2.18 (m, 4H, both), 1.43–1.38 (m, 6H, both), 1.29–1.24 (m, 6H, both); **^{13}C NMR** (150.9 MHz, $CDCl_3$) δ 174.1 (one diastereomer), 173.8 (other diastereomer), 169.9 (2C, both), 164.3 (d, J = 253.2 Hz, one diastereomer), 164.2 (d, J = 253.2 Hz, other diastereomer), 157.9 (2C, both), 157.7 (2C, both), 139.3 (2C, both), 138.7 (2C, both), 129.7 (d, J = 2.8 Hz, one diastereomer), 129.4 (d, J = 3.3 Hz, other diastereomer), 129.0 (d, J = 8.8 Hz, 2C, one diastereomer), 128.8 (d, J = 8.8 Hz, 2C, other diastereomer), 116.2 (d, J = 21.6 Hz, 2C, one diastereomer), 116.1 (d, J = 21.6 Hz, 2C, other diastereomer), 79.8 (2C, both), 74.3 (2C, both), 52.2 (2C, both), 42.2 (2C, both), 40.2 (one diastereomer), 39.8 (other diastereomer), 38.9 (one diastereomer), 38.6 (other diastereomer), 38.5 (other diastereomer), 38.3 (one diastereomer), 37.9 (one diastereomer), 37.0 (other diastereomer), 33.4 (2C, both), 31.1 (2C, both), 23.9 (other diastereomer), 22.6 (one diastereomer), 21.5 (2C, both), 20.8 (2C, both); **^{19}F NMR** (564.7 MHz, $CDCl_3$) δ –107.5 (one diastereomer), –108.2 (other diastereomer); **HRMS** (TOF MS ES+) *m/z*: $[M+H]^+$ calculated for $C_{25}H_{34}FO_{10}N_3S_3H$, 652.1469; found, 652.1469.

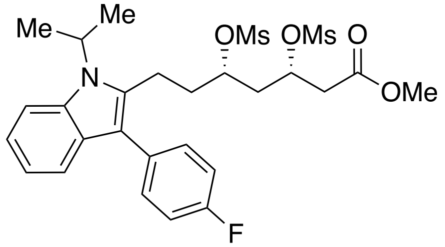

1,3-Dimesylate 21 was prepared by dissolving the prerequisite diol (0.44 g, 1.0 mmol, 1.0 equiv) in a solution of DMAP (26 mg, 0.21 mmol, 0.20 equiv) in DCM (5.0 mL, 0.20 M in substrate) under Schlenk conditions. Et_3N (0.43 mL, 3.1 mmol, 3.0 equiv) followed by MsCl (0.18 mL, 2.3 mmol, 2.2 equiv) were added sequentially to the flask and allowed to stir for 1h. The resulting solution was quenched with $NaHCO_3$, extracted with DCM (x3), washed with brine, dried with $MgSO_4$, filtered and concentrated in vacuo. The compound was purified by flash chromatography (0–60% EtOAc/hexanes) to afford the title compound as a clear, yellow oil (0.27 g, 0.46 mmol, 44%). **TLC R$_f$** = 0.6 (60% EtOAc/hexanes); **^{1}H NMR** (600 MHz, $CDCl_3$) δ 7.64 (d, J = 8.4 Hz, 1H), 7.57 (d, J = 7.9 Hz, 1H), 7.49–7.42 (m, 2H), 7.26–7.20 (m, 3H), 7.14 (t, J = 7.5 Hz, 1H), 5.20–5.14 (m, 1H), 4.97–4.91 (m, 1H), 4.78–4.71 (m, 1H), 3.77 (s, 3H), 3.13 (s, 3H), 3.01 (s, 3H), 3.10–3.02 (m, 1H), 3.00–2.93 (m, 1H), 2.83 (ddd, J = 53.9, 16.9, 7.5 Hz, 2H), 2.41–2.35 (m, 1H), 2.26–2.12 (m, 2H), 2.09–2.00 (m, 1H), 1.79 (d, J = 7.0 Hz, 3H), 1.77 (d, J = 7.0 Hz, 3H); **^{13}C NMR** (150.9 MHz, $CDCl_3$) δ 169.9, 161.5 (d, J = 245.0 Hz, 2C), 134.9, 134.3, 131.6 (d, J = 7.7 Hz, 2C), 128.4, 121.1, 119.4, 119.1, 115.5 (d, J = 21.0 Hz, 2C), 113.7, 112.0, 77.5, 74.5, 52.2, 47.4, 39.5, 38.9, 38.6 (2C), 35.1, 21.6 (2C), 20.6; **^{19}F NMR** (564.7 MHz, $CDCl_3$) δ –116.5; **HRMS** (TOF MS ES+) *m/z*: $[M]^+$ calculated for $C_{27}H_{34}FO_8NS_2$, 583.1710; found, 583.1701.

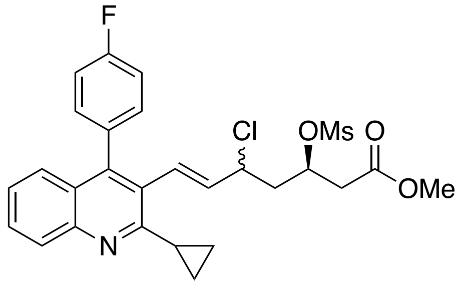

1,3-Dimesylate 23 was prepared by dissolving the prerequisite diol (0.23 g, 0.54 mmol, 1.0 equiv) in a solution of DMAP (12 mg, 0.10 mmol, 0.20 equiv) in DCM (3.0 mL, 0.18 M in substrate) under Schlenk conditions. Et_3N (0.22 mL, 1.6 mmol, 3.0 equiv) followed by MsCl (0.090 mL, 1.1 mmol, 2.2 equiv) were added sequentially to the flask and allowed to stir for 1h. The resulting solution was quenched with $NaHCO_3$, extracted with DCM (x3), washed with brine, dried with $MgSO_4$, filtered and concentrated in vacuo. The compound was purified by flash chromatography (0–60% EtOAc/hexanes) to afford the title compound in a 1:1 mixture of diastereomers as a clear, yellow oil (33 mg, 0.062 mmol, 12%). **TLC R_f** = 0.5 (60% EtOAc/hexanes); **^{1}H NMR** (400 MHz, $CDCl_3$) δ 7.97 (d, *J* = 8.3 Hz, 2H, both), 7.61 (bs, 2H, both), 7.42–7.29 (m, 4H, both), 7.29–7.15 (m, 8H, both), 6.74 (dd, *J* = 39.3, 16.2 Hz, 2H, both), 5.71–5.55 (m, 2H, both), 5.23–5.15 (m, 1H, one diastereomer), 4.92–4.83 (m, 1H, other diastereomer), 4.61–4.48 (m, 2H, both), 3.74 (s, 6H, both), 3.10 (s, 3H, one diastereomer), 3.04 (s, 3H, other diastereomer), 2.93–2.64 (m, 4H, both), 2.49–2.27 (m, 4H, both), 2.11–2.00 (m, 2H, both), 1.41–1.30 (m, 4H, both), 1.12–1.02 (m, 4H, both); **^{13}C NMR** (100.6 MHz, $CDCl_3$) δ 169.8 (2C, both), 160.7 (d, *J* = 21.8 Hz, 2C, both), 147.3 (2C, both), 145.0 (2C, both), 136.4 (2C, both), 132.1 (d, *J* = 8.5 Hz, 2C, both), 132.0 (2C, both), 130.3 (2C, both), 129.3 (2C, both), 129.1 (d, *J* = 20.0 Hz, one diastereomer), 128.4 (d, *J* = 20.0 Hz, other diastereomer), 126.3 (2C, both), 125.8 (d, *J* = 8.1 Hz, 4C, both), 115.9 (2C, both), 115.7 (2C, both), 76.0 (2C, both), 58.0 (2C, both), 53.6 (2C, both), 52.3 (2C, both), 43.1 (2C, both), 39.9 (2C, both), 39.3 (2C, both), 38.9 (2C, both), 38.6 (2C, both), 16.3 (2C, both), 10.5 (4C, both); **^{19}F NMR** (564.7 MHz, $CDCl_3$) δ –114.3; **HRMS** (TOF MS ES+) *m/z*: $[M+H]^+$ calculated for $C_{27}H_{27}FClO_5NSH$, 532.1360; found, 532.1360.

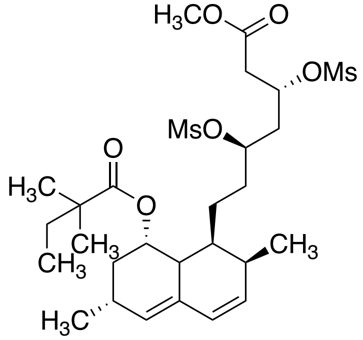

1,3-Dimesylate 25 was prepared by dissolving the prerequisite diol (0.27 g, 0.60 mmol, 1.0 equiv) in a solution of DMAP (17 mg, 0.12 mmol, 0.20 equiv) in DCM (5.0 mL, 0.12 M in substrate) under Schlenk conditions. Et_3N (0.25 mL, 1.8 mmol, 3.0 equiv) followed by MsCl (0.10 mL, 1.3 mmol, 2.2 equiv) were added sequentially to the flask and allowed to stir for 1h. The resulting solution was quenched with $NaHCO_3$, extracted with DCM (x3), washed with brine, dried with $MgSO_4$, filtered and concentrated in vacuo. The compound was purified by flash chromatography (0–60% EtOAc/hexanes) to afford the title compound as a clear, yellow oil (0.16 g, 0.26 mmol,

43%). **TLC R_f** = 0.4 (60% EtOAc/hexanes); **^{1}H NMR** (500 MHz, $CDCl_3$) δ 6.00 (d, *J* = 9.7 Hz, 1H), 5.82–5.76 (m, 1H), 5.52 (s, 1H), 5.41 (d, *J* = 2.9 Hz, 1H), 5.21–5.13 (m, 1H), 4.81–4.72 (m, 1H), 3.72 (s, 3H), 3.12 (s, 3H), 3.10 (s, 3H), 2.86 (d, *J* = 6.2 Hz, 2H), 2.50–2.43 (m, 1H), 2.40–2.24 (m, 3H), 2.20–2.11 (m, 1H), 2.03–1.86 (m, 3H), 1.71–1.50 (m, 6H), 1.15 (d, *J* = 2.9 Hz, 6H), 1.11 (d, *J* = 7.5 Hz, 3H), 0.89 (d, *J* = 7.0 Hz, 3H), 0.85 (t, *J* = 7.6 Hz, 3H); **^{13}C NMR** (125.8 MHz, $CDCl_3$) δ 177.8, 170.0, 132.7, 131.3, 129.9, 128.4, 78.5, 75.3, 67.6, 52.1, 43.0, 39.8, 38.5 (3C), 37.6, 36.4, 33.1, 32.9, 32.1, 30.5, 27.3, 24.8, 24.6, 23.7, 23.1, 13.9, 9.4; **HRMS** (TOF MS ES+) *m/z*: $[M+Na]^+$ calculated for $C_{28}H_{46}O_{10}S_2Na$: 629.2430; found 629.2432.

---

[i] A. B. Sanford, T. A. Thane, T. M. McGinnis, P.–P. Chen, X. Hong, E. R. Jarvo, *J. Am. Chem. Soc.* **2020**, *142*, 5017–5023

1H spectrum

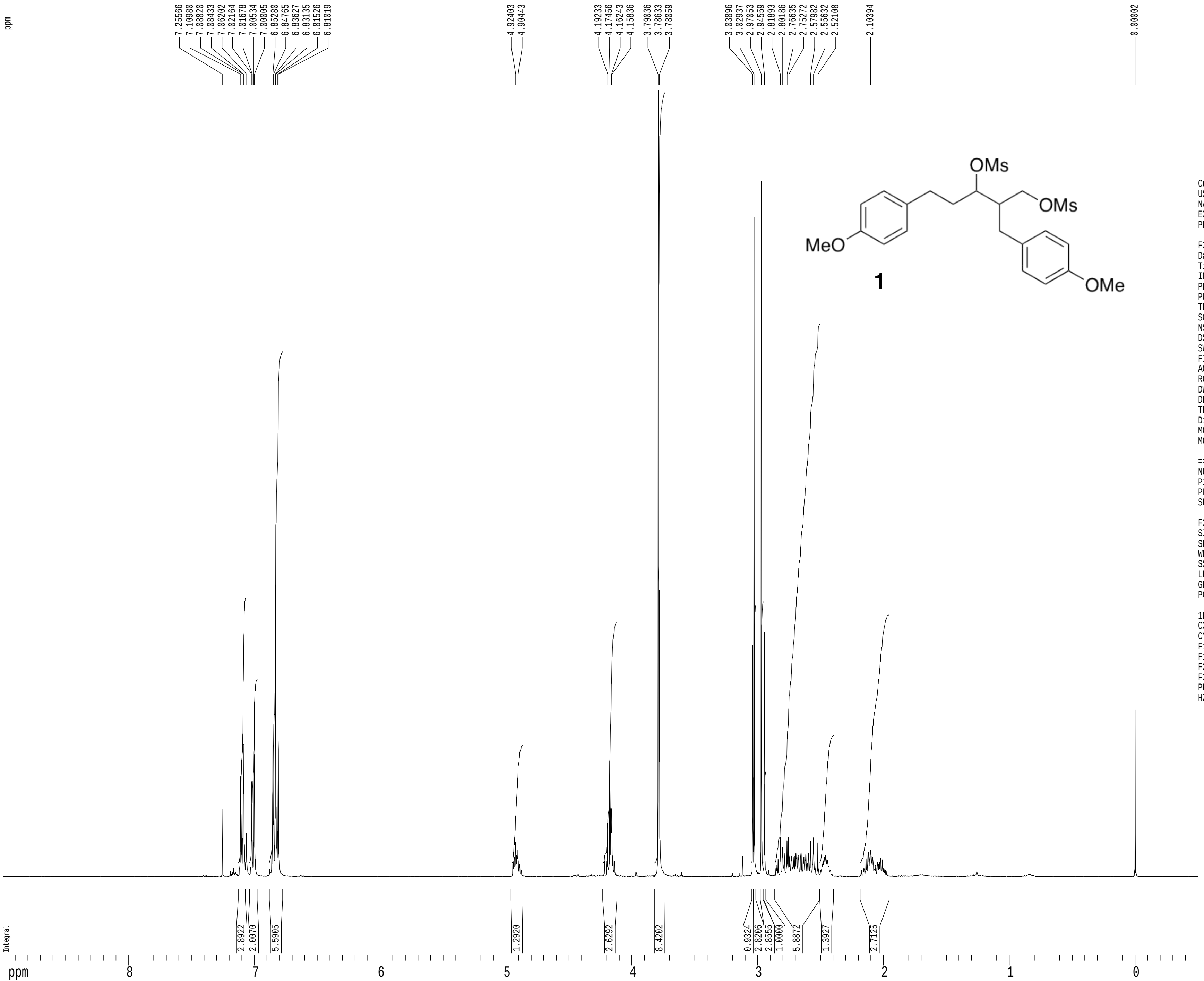

1

Current Data Parameters
USER sanforda
NAME ABS-2-076-proton
EXPNO 1
PROCNO 1

F2 - Acquisition Parameters
Date_ 20191031
Time 18.01
INSTRUM drx400
PROBHD 5 mm QNP H/F/P
PULPROG zg30
TD 65536
SOLVENT CDCl3
NS 8
DS 2
SWH 6410.256 Hz
FIDRES 0.097813 Hz
AQ 5.1118579 sec
RG 161.3
DW 78.000 usec
DE 4.50 usec
TE 298.0 K
D1 0.10000000 sec
MCREST 0.00000000 sec
MCWRK 0.01500000 sec

======== CHANNEL f1 ========
NUC1 1H
P1 12.00 usec
PL1 -1.10 dB
SFO1 400.1328009 MHz

F2 - Processing parameters
SI 65536
SF 400.1300228 MHz
WDW no
SSB 0
LB 0.00 Hz
GB 0
PC 2.00

1D NMR plot parameters
CX 22.80 cm
CY 15.00 cm
F1P 9.000 ppm
F1 3601.17 Hz
F2P -0.500 ppm
F2 -200.06 Hz
PPMCM 0.41667 ppm/cm
HZCM 166.72086 Hz/cm

1H spectrum

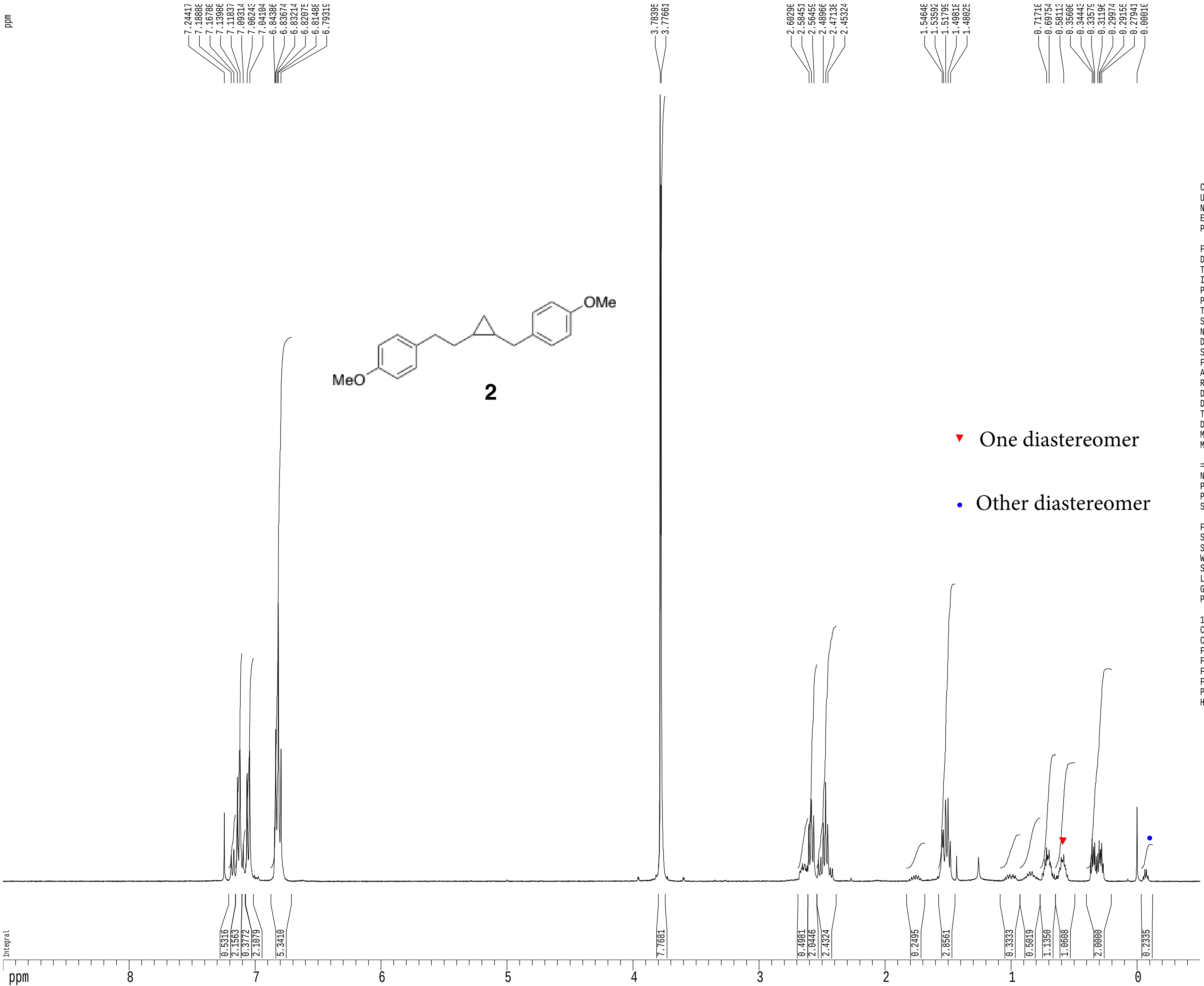

Current Data Parameters
USER tthane
NAME TATv185c-pure
EXPNO 1
PROCNO 1

F2 - Acquisition Parameters
Date_ 20210910
Time 16.52
INSTRUM drx400
PROBHD 5 mm Multinucl
PULPROG zg30
TD 65536
SOLVENT CDCl3T
NS 8
DS 2
SWH 6410.256 Hz
FIDRES 0.097813 Hz
AQ 5.1118579 sec
RG 287.4
DW 78.000 usec
DE 4.50 usec
TE 298.1 K
D1 0.10000000 sec
MCREST 0.00000000 sec
MCWRK 0.01500000 sec

======== CHANNEL f1 ========
NUC1 1H
P1 12.00 usec
PL1 -1.10 dB
SFO1 400.1328009 MHz

F2 - Processing parameters
SI 65536
SF 400.1300282 MHz
WDW no
SSB 0
LB 0.00 Hz
GB 0
PC 2.00

1D NMR plot parameters
CX 22.80 cm
CY 15.00 cm
F1P 9.000 ppm
F1 3601.17 Hz
F2P -0.500 ppm
F2 -200.06 Hz
PPMCM 0.41667 ppm/cm
HZCM 166.72086 Hz/cm

1H spectrum

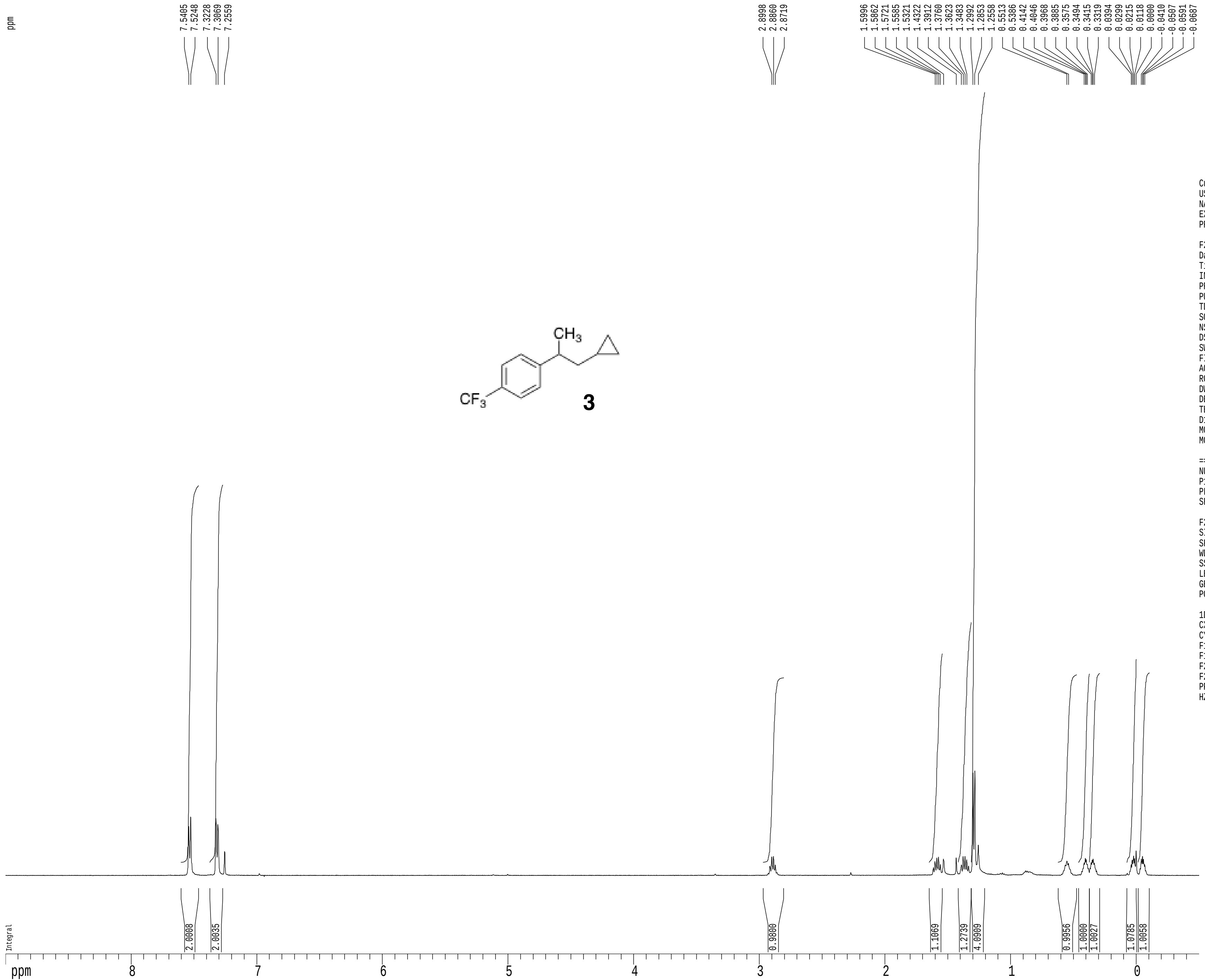

Current Data Parameters
USER tthane
NAME TATv252
EXPNO 1
PROCNO 1

F2 - Acquisition Parameters
Date_ 20211203
Time 16.28
INSTRUM gn500
PROBHD 5 mm broadband
PULPROG zg30
TD 81728
SOLVENT CDCl3T
NS 8
DS 2
SWH 8012.820 Hz
FIDRES 0.098043 Hz
AQ 5.0998774 sec
RG 812.7
DW 62.400 usec
DE 6.00 usec
TE 298.0 K
D1 0.10000000 sec
MCREST 0.00000000 sec
MCWRK 0.01500000 sec

======== CHANNEL f1 ========
NUC1 1H
P1 12.00 usec
PL1 -6.00 dB
SFO1 498.6534906 MHz

F2 - Processing parameters
SI 65536
SF 498.6500313 MHz
WDW no
SSB 0
LB 0.00 Hz
GB 0
PC 1.00

1D NMR plot parameters
CX 22.80 cm
CY 2.00 cm
F1P 9.000 ppm
F1 4487.85 Hz
F2P -0.500 ppm
F2 -249.32 Hz
PPMCM 0.41667 ppm/cm
HZCM 207.77084 Hz/cm

Z-restored spin-echo 13C spectrum with 1H decoupling

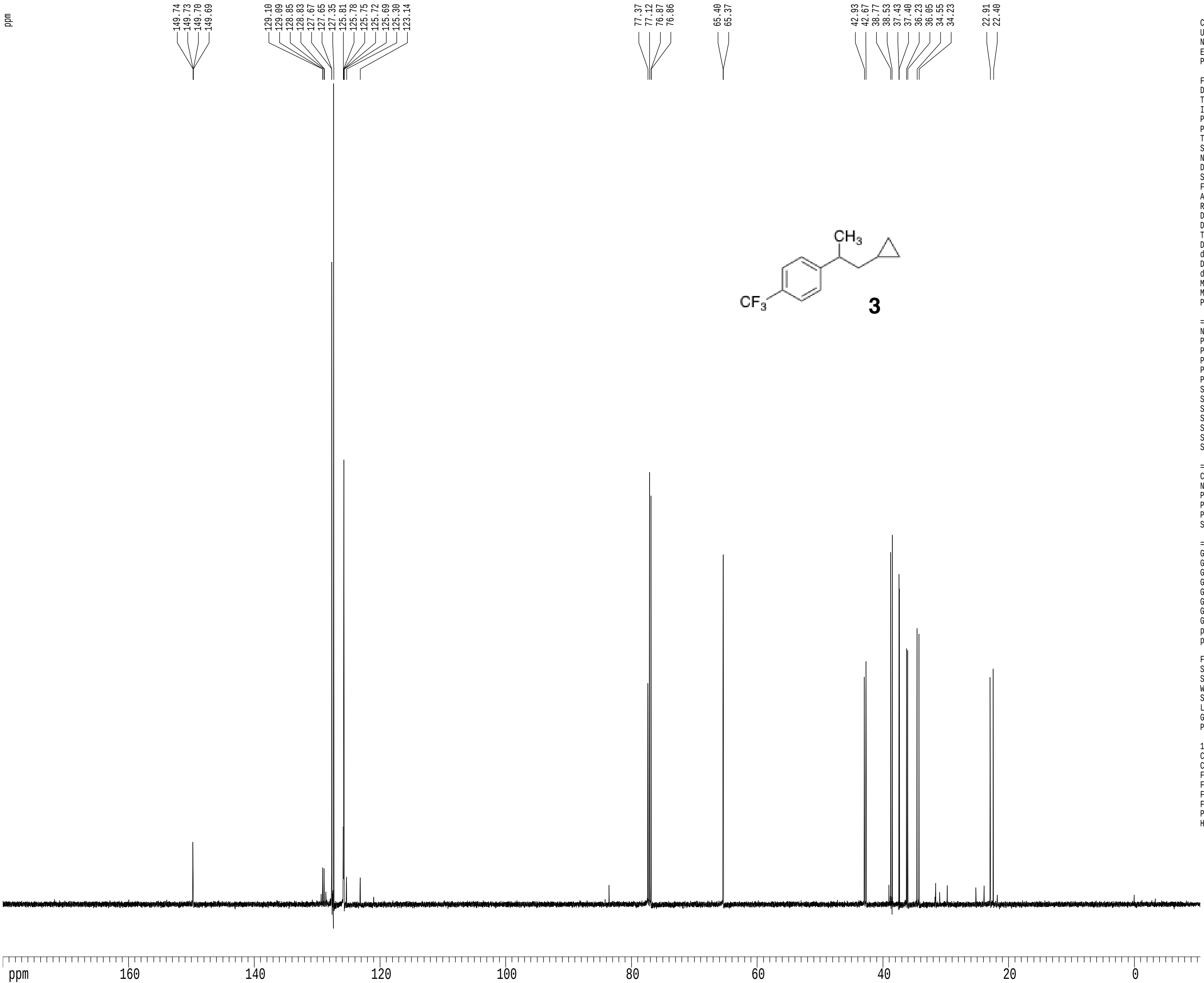

Current Data Parameters
USER tthane
NAME TATi215c
EXPNO 4
PROCNO 1

F2 - Acquisition Parameters
Date_ 20181016
Time 9.46
INSTRUM cryo500
PROBHD 5 mm CPTCI 1H-
PULPROG SpinEchopg30gp2.prd
TD 65536
SOLVENT CDCl3
NS 1024
DS 16
SWH 30303.031 Hz
FIDRES 0.462388 Hz
AQ 1.0813940 sec
RG 7298.2
DW 16.500 usec
DE 6.00 usec
TE 298.0 K
D1 0.25000000 sec
d11 0.03000000 sec
D16 0.00020000 sec
d17 0.00019600 sec
MCREST 0.00000000 sec
MCWRK 0.01500000 sec
P2 33.10 usec

======== CHANNEL f1 ========
NUC1 13C
P1 16.55 usec
P12 2000.00 usec
P20 500.00 usec
PL0 120.00 dB
PL1 -1.00 dB
SFO1 125.7942548 MHz
SP2 2.70 dB
SP4 2.70 dB
SPNAM2 Crp60comp.4
SPNAM4 Crp60,0.5,20.1
SPOFF2 0.00 Hz
SPOFF4 0.00 Hz

======== CHANNEL f2 ========
CPDPRG2 waltz16
NUC2 1H
PCPD2 100.00 usec
PL2 1.60 dB
PL12 23.54 dB
SFO2 500.2225011 MHz

====== GRADIENT CHANNEL =====
GPNAM1 SINE.100
GPNAM2 SINE.100
GPX1 0.00 %
GPX2 0.00 %
GPY1 0.00 %
GPY2 0.00 %
GPZ1 30.00 %
GPZ2 50.00 %
p15 500.00 usec
p16 1000.00 usec

F2 - Processing parameters
SI 65536
SF 125.7804190 MHz
WDW no
SSB 0
LB 0.00 Hz
GB 0
PC 2.00

1D NMR plot parameters
CX 22.80 cm
CY 15.65 cm
F1P 180.000 ppm
F1 22640.47 Hz
F2P -10.460 ppm
F2 -1315.66 Hz
PPMCM 8.35351 ppm/cm
HZCM 1050.70776 Hz/cm

19F spectrum

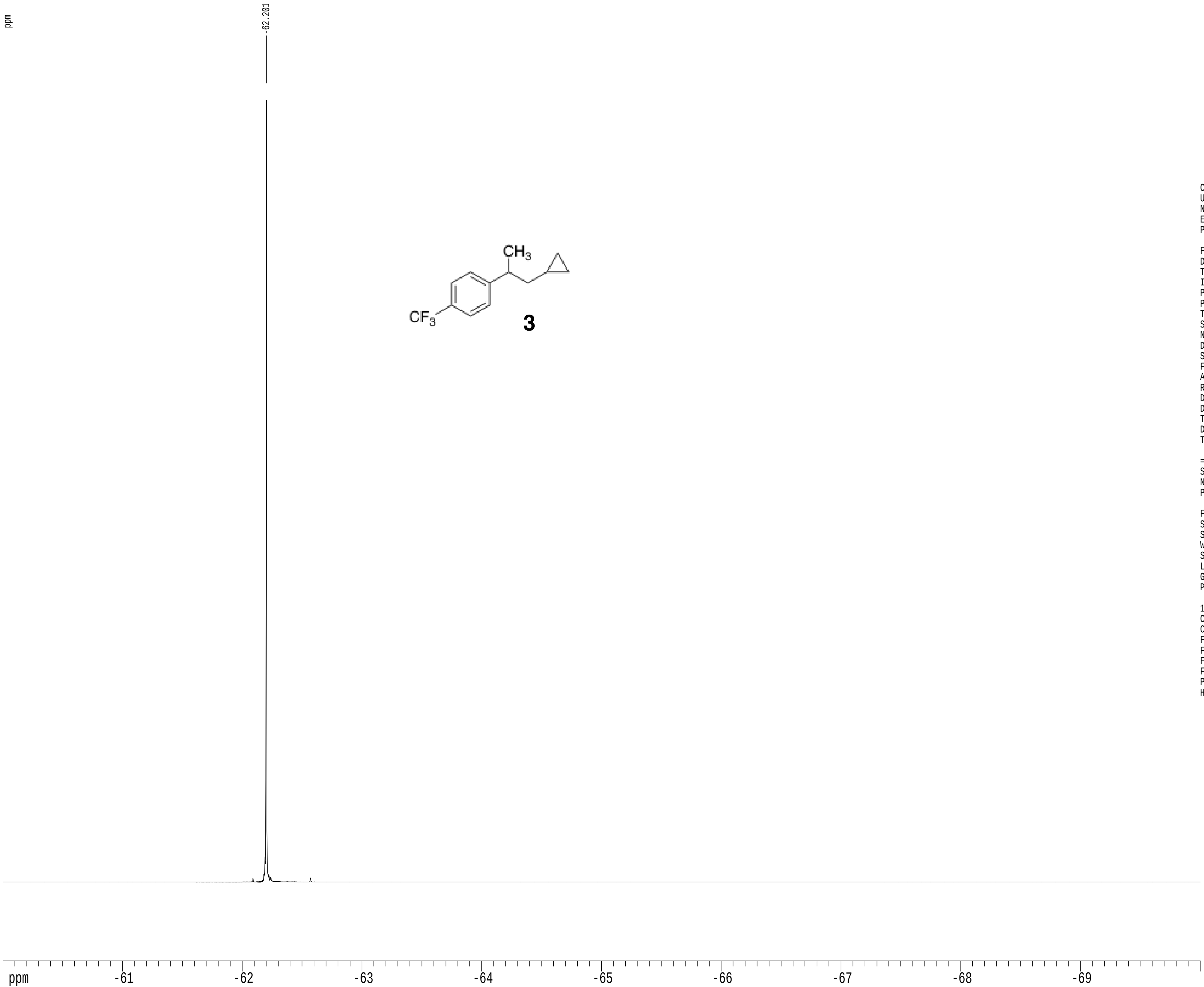


```
Current Data Parameters
USER              tthane
NAME      TATv252fluorine
EXPNO                  1
PROCNO                 1

F2 - Acquisition Parameters
Date_           20211217
Time               15.12
INSTRUM            av600
PROBHD   5 mm CPBBO BB-
PULPROG         zgflqn30
TD                131072
SOLVENT            CDCl3T
NS                    16
DS                     2
SWH           178571.422 Hz
FIDRES          1.362392 Hz
AQ             0.3670516 sec
RG                   575
DW                 2.800 usec
DE                 18.00 usec
TE                 298.0 K
D1            3.00000000 sec
TD0                    1

======== CHANNEL f1 ========
SFO1         564.6299196 MHz
NUC1                 19F
P1                 18.25 usec

F2 - Processing parameters
SI                131072
SF           564.6863858 MHz
WDW                   no
SSB                    0
LB                  0.00 Hz
GB                     0
PC                  1.00

1D NMR plot parameters
CX                 22.80 cm
CY                 15.00 cm
F1P              -60.000 ppm
F1             -33881.19 Hz
F2P              -70.000 ppm
F2             -39528.05 Hz
PPMCM            0.43860 ppm/cm
HZCM           247.66939 Hz/cm
```

1H spectrum

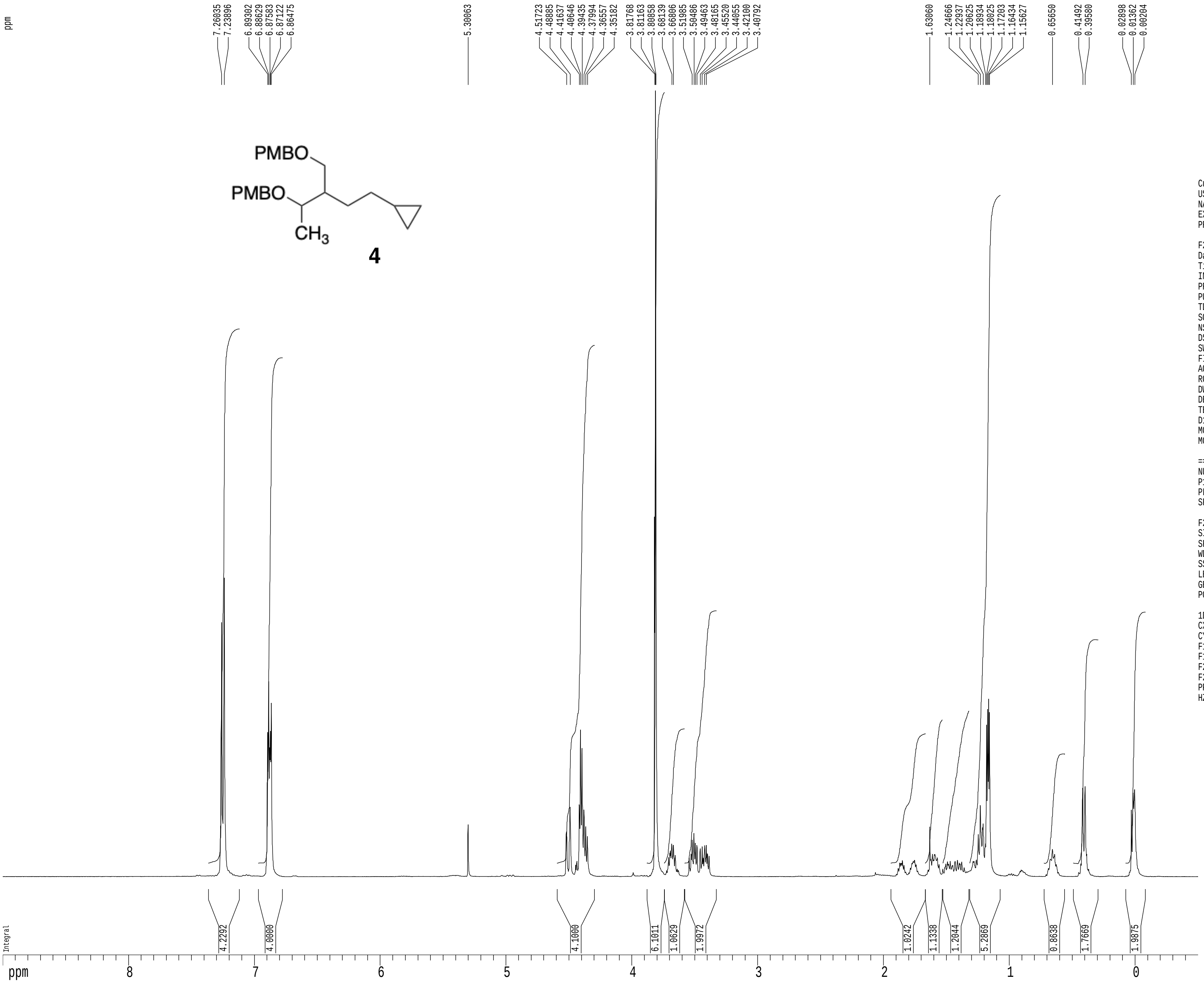

Current Data Parameters
USER mcginnit
NAME tmm-1-181
EXPNO 2
PROCNO 1

F2 - Acquisition Parameters
Date_ 20190821
Time 9.52
INSTRUM drx400
PROBHD 5 mm QNP H/F/P
PULPROG zg30
TD 65536
SOLVENT CDCl3
NS 8
DS 2
SWH 6410.256 Hz
FIDRES 0.097813 Hz
AQ 5.1118579 sec
RG 128
DW 78.000 usec
DE 4.50 usec
TE 298.0 K
D1 0.10000000 sec
MCREST 0.00000000 sec
MCWRK 0.01500000 sec

======== CHANNEL f1 ========
NUC1 1H
P1 12.00 usec
PL1 -1.10 dB
SFO1 400.1328009 MHz

F2 - Processing parameters
SI 65536
SF 400.1300175 MHz
WDW EM
SSB 0
LB 0.30 Hz
GB 0
PC 2.00

1D NMR plot parameters
CX 22.80 cm
CY 15.00 cm
F1P 9.000 ppm
F1 3601.17 Hz
F2P -0.500 ppm
F2 -200.06 Hz
PPMCM 0.41667 ppm/cm
HZCM 166.72084 Hz/cm

1H spectrum

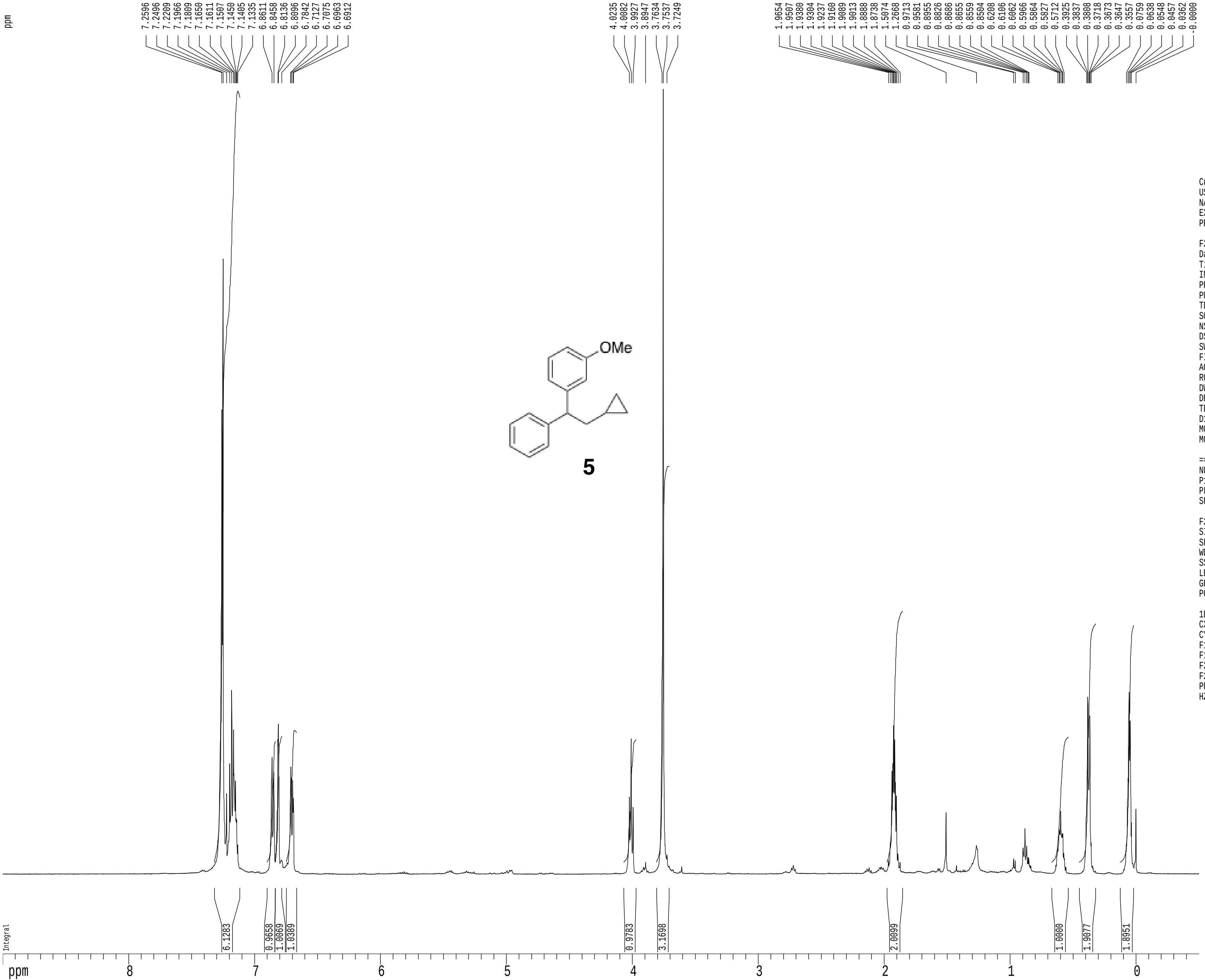


```
Current Data Parameters
USER              tthane
NAME            TATii58c
EXPNO                  1
PROCNO                 1

F2 - Acquisition Parameters
Date_           20190110
Time               18.01
INSTRUM          cryo500
PROBHD   5 mm CPTCI 1H-
PULPROG             zg30
TD                 81728
SOLVENT            CDCl3T
NS                     8
DS                     2
SWH             8012.820 Hz
FIDRES          0.098043 Hz
AQ             5.0998774 sec
RG                   3.2
DW                62.400 usec
DE                  6.00 usec
TE                 298.0 K
D1            0.10000000 sec
MCREST        0.00000000 sec
MCWRK         0.01500000 sec

======== CHANNEL f1 ========
NUC1                  1H
P1                  7.50 usec
PL1                 1.60 dB
SFO1         500.2235015 MHz

F2 - Processing parameters
SI                 65536
SF           500.2200513 MHz
WDW                   no
SSB                    0
LB                  0.00 Hz
GB                     0
PC                  1.00

1D NMR plot parameters
CX                 22.80 cm
CY                 15.00 cm
F1P                9.000 ppm
F1               4501.98 Hz
F2P               -0.500 ppm
F2               -250.11 Hz
PPMCM            0.41667 ppm/cm
HZCM           208.42503 Hz/cm
```

1H spectrum

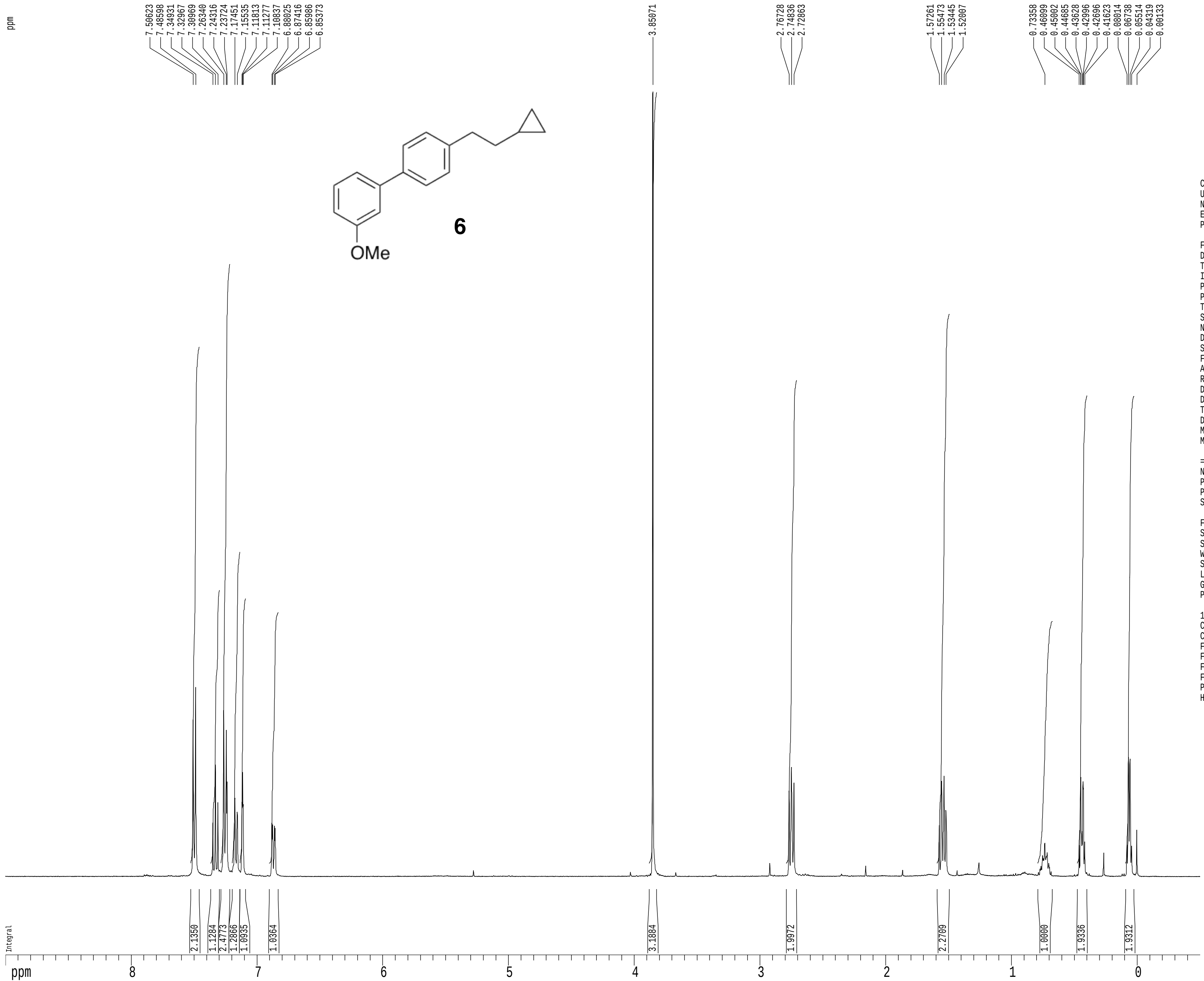


```
Current Data Parameters
USER            sanforda
NAME     ABS-2-065-pure
EXPNO                  1
PROCNO                 1

F2 - Acquisition Parameters
Date_           20181025
Time               19.24
INSTRUM           drx400
PROBHD   5 mm QNP H/F/P
PULPROG             zg30
TD                 65536
SOLVENT            CDCl3
NS                     8
DS                     2
SWH             6410.256 Hz
FIDRES          0.097813 Hz
AQ             5.1118579 sec
RG                 161.3
DW                78.000 usec
DE                  4.50 usec
TE                 298.0 K
D1            0.10000000 sec
MCREST        0.00000000 sec
MCWRK         0.01500000 sec

======== CHANNEL f1 ========
NUC1                  1H
P1                 12.00 usec
PL1                -1.10 dB
SFO1         400.1328009 MHz

F2 - Processing parameters
SI                 65536
SF           400.1300304 MHz
WDW                   no
SSB                    0
LB                  0.00 Hz
GB                     0
PC                  2.00

1D NMR plot parameters
CX                 22.80 cm
CY                 15.00 cm
F1P                9.000 ppm
F1               3601.17 Hz
F2P               -0.500 ppm
F2               -200.06 Hz
PPMCM            0.41667 ppm/cm
HZCM           166.72086 Hz/cm
```

1H spectrum

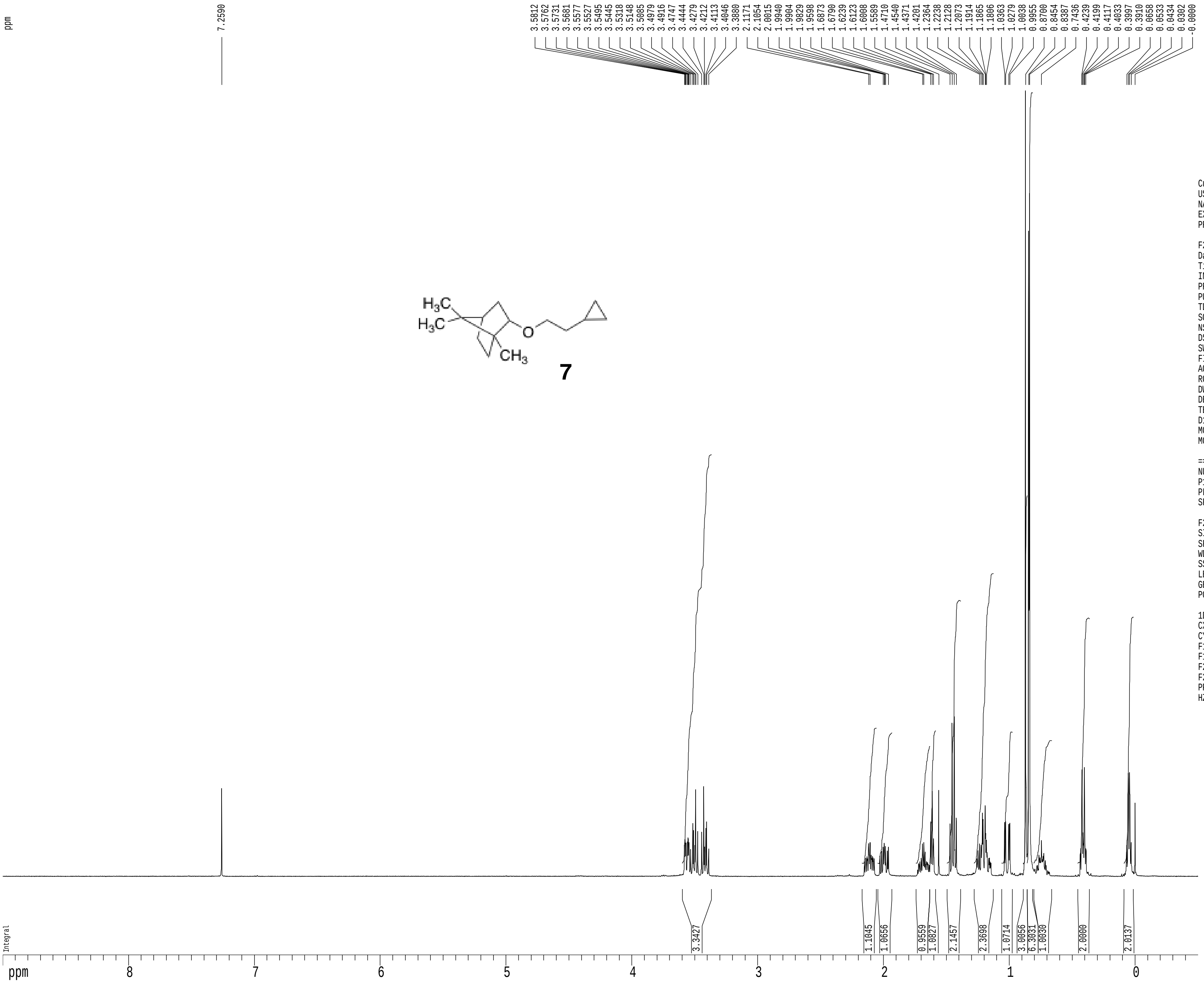

7

Current Data Parameters
USER tthane
NAME TATv255purereal
EXPNO 1
PROCNO 1

F2 - Acquisition Parameters
Date_ 20211206
Time 15.24
INSTRUM drx400
PROBHD 5 mm QNP H/F/P
PULPROG zg30
TD 65536
SOLVENT CDCl3T
NS 8
DS 2
SWH 6410.256 Hz
FIDRES 0.097813 Hz
AQ 5.1118579 sec
RG 143.7
DW 78.000 usec
DE 4.50 usec
TE 298.0 K
D1 0.10000000 sec
MCREST 0.00000000 sec
MCWRK 0.01500000 sec

======== CHANNEL f1 ========
NUC1 1H
P1 12.00 usec
PL1 -0.90 dB
SFO1 400.1328009 MHz

F2 - Processing parameters
SI 65536
SF 400.1300217 MHz
WDW no
SSB 0
LB 0.00 Hz
GB 0
PC 2.00

1D NMR plot parameters
CX 22.80 cm
CY 15.00 cm
F1P 9.000 ppm
F1 3601.17 Hz
F2P -0.500 ppm
F2 -200.06 Hz
PPMCM 0.41667 ppm/cm
HZCM 166.72086 Hz/cm

1H spectrum

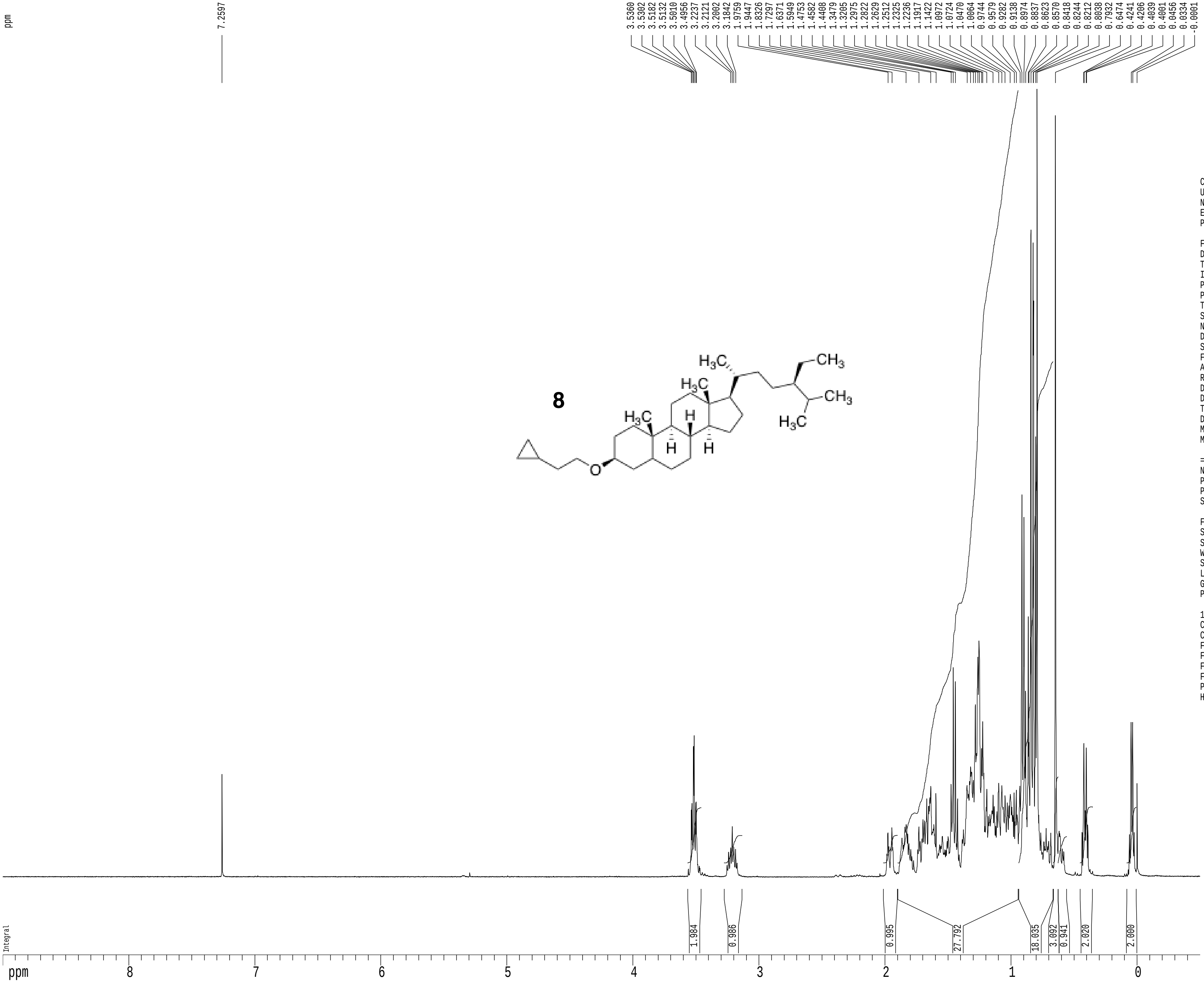

Current Data Parameters
USER tthane
NAME TATv256pure
EXPNO 1
PROCNO 1

F2 - Acquisition Parameters
Date_ 20211204
Time 13.31
INSTRUM drx400
PROBHD 5 mm QNP H/F/P
PULPROG zg30
TD 65536
SOLVENT CDCl3T
NS 8
DS 2
SWH 6410.256 Hz
FIDRES 0.097813 Hz
AQ 5.1118579 sec
RG 57
DW 78.000 usec
DE 4.50 usec
TE 298.0 K
D1 0.10000000 sec
MCREST 0.00000000 sec
MCWRK 0.01500000 sec

======== CHANNEL f1 ========
NUC1 1H
P1 12.00 usec
PL1 -0.90 dB
SFO1 400.1328009 MHz

F2 - Processing parameters
SI 65536
SF 400.1300217 MHz
WDW no
SSB 0
LB 0.00 Hz
GB 0
PC 2.00

1D NMR plot parameters
CX 22.80 cm
CY 15.00 cm
F1P 9.000 ppm
F1 3601.17 Hz
F2P -0.500 ppm
F2 -200.06 Hz
PPMCM 0.41667 ppm/cm
HZCM 166.72086 Hz/cm

1H spectrum

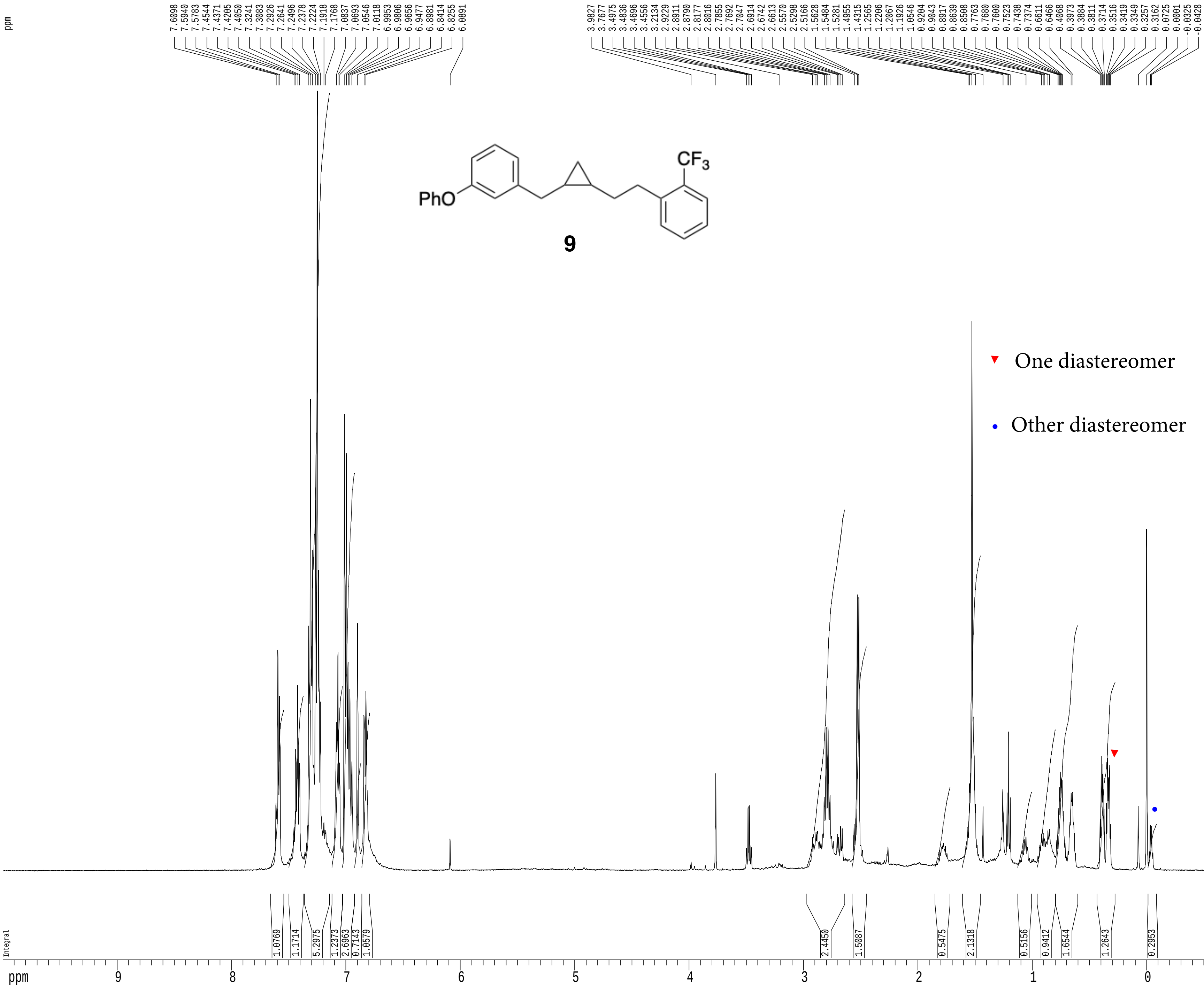


```
Current Data Parameters
USER              mcginnit
NAME              tmm-1-292
EXPNO                    1
PROCNO                   1

F2 - Acquisition Parameters
Date_             20191204
Time                 17.38
INSTRUM            cryo500
PROBHD   5 mm CPTCI 1H-
PULPROG               zg30
TD                   81728
SOLVENT              CDCl3
NS                       8
DS                       2
SWH              8012.820 Hz
FIDRES           0.098043 Hz
AQ              5.0998774 sec
RG                     6.3
DW                  62.400 usec
DE                    6.00 usec
TE                   298.0 K
D1             0.10000000 sec
MCREST         0.00000000 sec
MCWRK          0.01500000 sec

======== CHANNEL f1 ========
NUC1                    1H
P1                    7.50 usec
PL1                   1.60 dB
SFO1           500.2235015 MHz

F2 - Processing parameters
SI                   65536
SF             500.2200366 MHz
WDW                     EM
SSB                      0
LB                    0.30 Hz
GB                       0
PC                    1.00

1D NMR plot parameters
CX                   22.80 cm
CY                   15.00 cm
F1P                 10.000 ppm
F1                 5002.20 Hz
F2P                 -0.500 ppm
F2                 -250.11 Hz
PPMCM              0.46053 ppm/cm
HZCM             230.36450 Hz/cm
```

1H spectrum

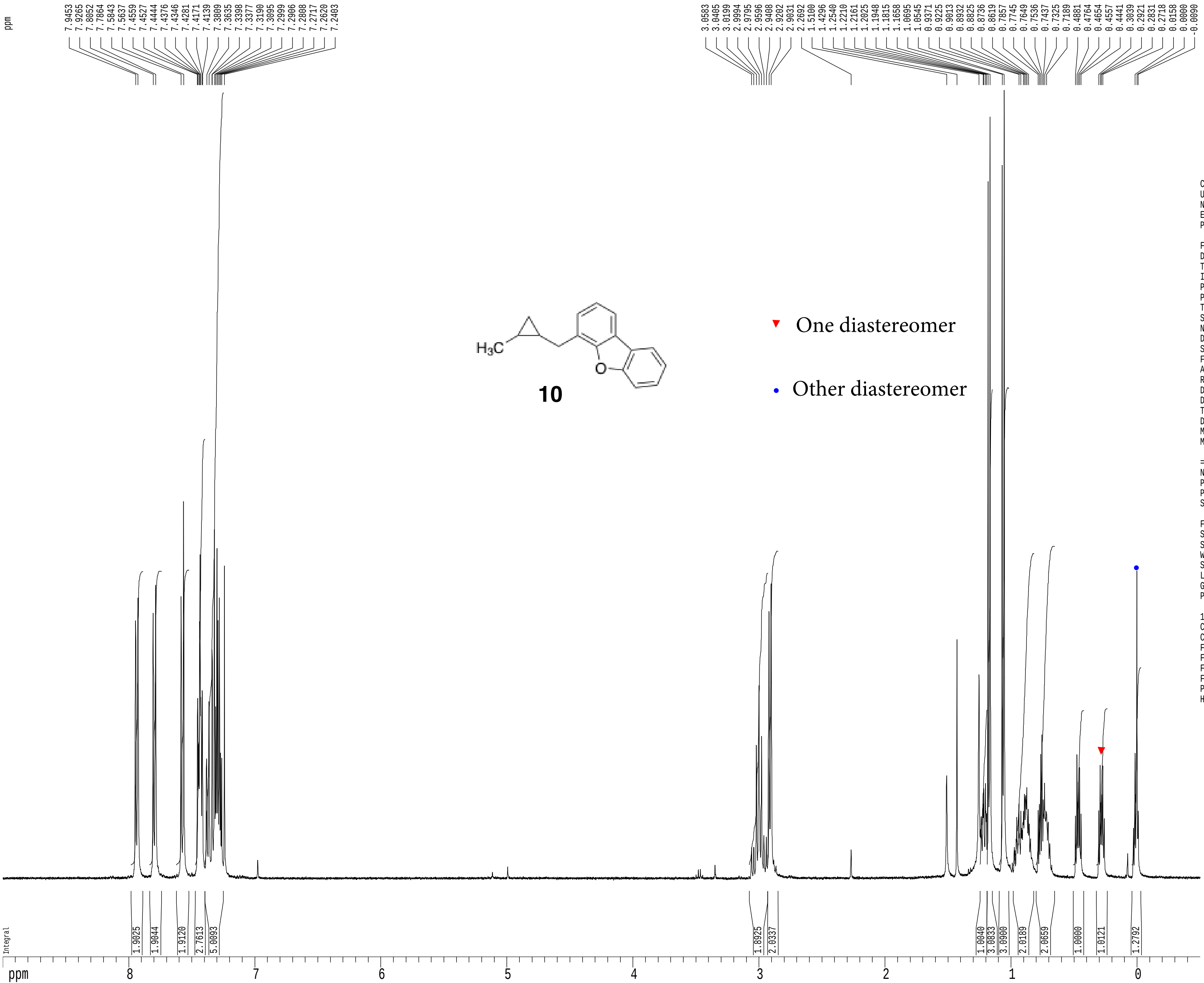


```
Current Data Parameters
USER            tthane
NAME        TATv251pure
EXPNO                1
PROCNO               1

F2 - Acquisition Parameters
Date_          20211202
Time              12.53
INSTRUM          drx400
PROBHD   5 mm QNP H/F/P
PULPROG            zg30
TD                65536
SOLVENT          CDCl3T
NS                    8
DS                    2
SWH            6410.256 Hz
FIDRES         0.097813 Hz
AQ            5.1118579 sec
RG                203.2
DW               78.000 usec
DE                 4.50 usec
TE                298.0 K
D1           0.10000000 sec
MCREST       0.00000000 sec
MCWRK        0.01500000 sec

======== CHANNEL f1 ========
NUC1                 1H
P1                12.00 usec
PL1               -0.90 dB
SFO1        400.1328009 MHz

F2 - Processing parameters
SI                65536
SF          400.1300293 MHz
WDW                  no
SSB                   0
LB                 0.00 Hz
GB                    0
PC                 2.00

1D NMR plot parameters
CX                22.80 cm
CY                15.00 cm
F1P               9.000 ppm
F1              3601.17 Hz
F2P              -0.500 ppm
F2              -200.06 Hz
PPMCM           0.41667 ppm/cm
HZCM          166.72086 Hz/cm
```

1H spectrum

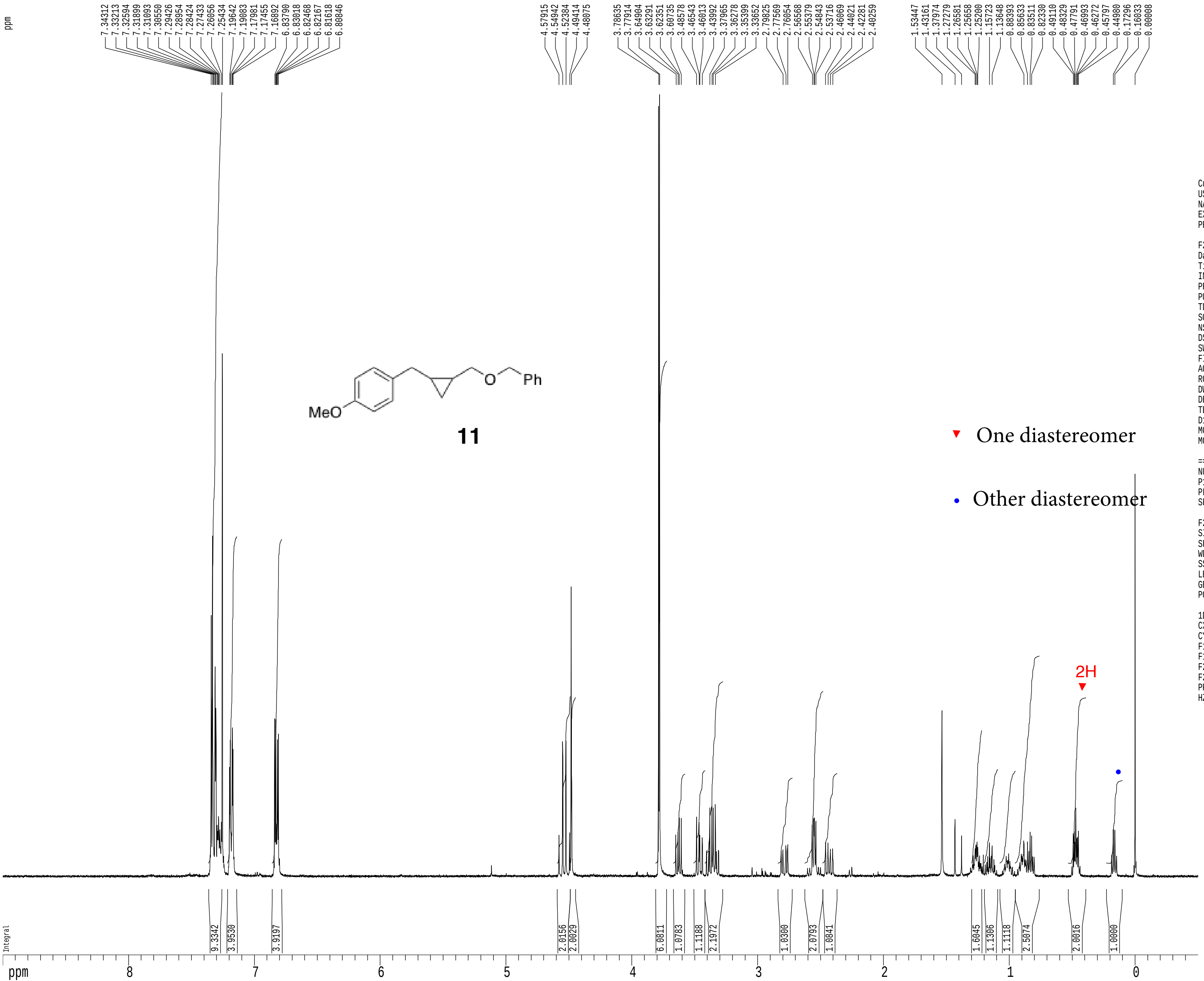

Current Data Parameters
USER tthane
NAME TATv261char
EXPNO 5
PROCNO 1

F2 - Acquisition Parameters
Date_ 20211220
Time 14.14
INSTRUM drx400
PROBHD 5 mm QNP H/F/P
PULPROG zg30
TD 65536
SOLVENT CDCl3T
NS 8
DS 2
SWH 6410.256 Hz
FIDRES 0.097813 Hz
AQ 5.1118579 sec
RG 322.5
DW 78.000 usec
DE 4.50 usec
TE 298.0 K
D1 0.10000000 sec
MCREST 0.00000000 sec
MCWRK 0.01500000 sec

======== CHANNEL f1 ========
NUC1 1H
P1 12.00 usec
PL1 -0.90 dB
SFO1 400.1328009 MHz

F2 - Processing parameters
SI 65536
SF 400.1300235 MHz
WDW no
SSB 0
LB 0.00 Hz
GB 0
PC 2.00

1D NMR plot parameters
CX 22.80 cm
CY 15.00 cm
F1P 9.000 ppm
F1 3601.17 Hz
F2P -0.500 ppm
F2 -200.06 Hz
PPMCM 0.41667 ppm/cm
HZCM 166.72086 Hz/cm

13C spectrum with 1H decoupling

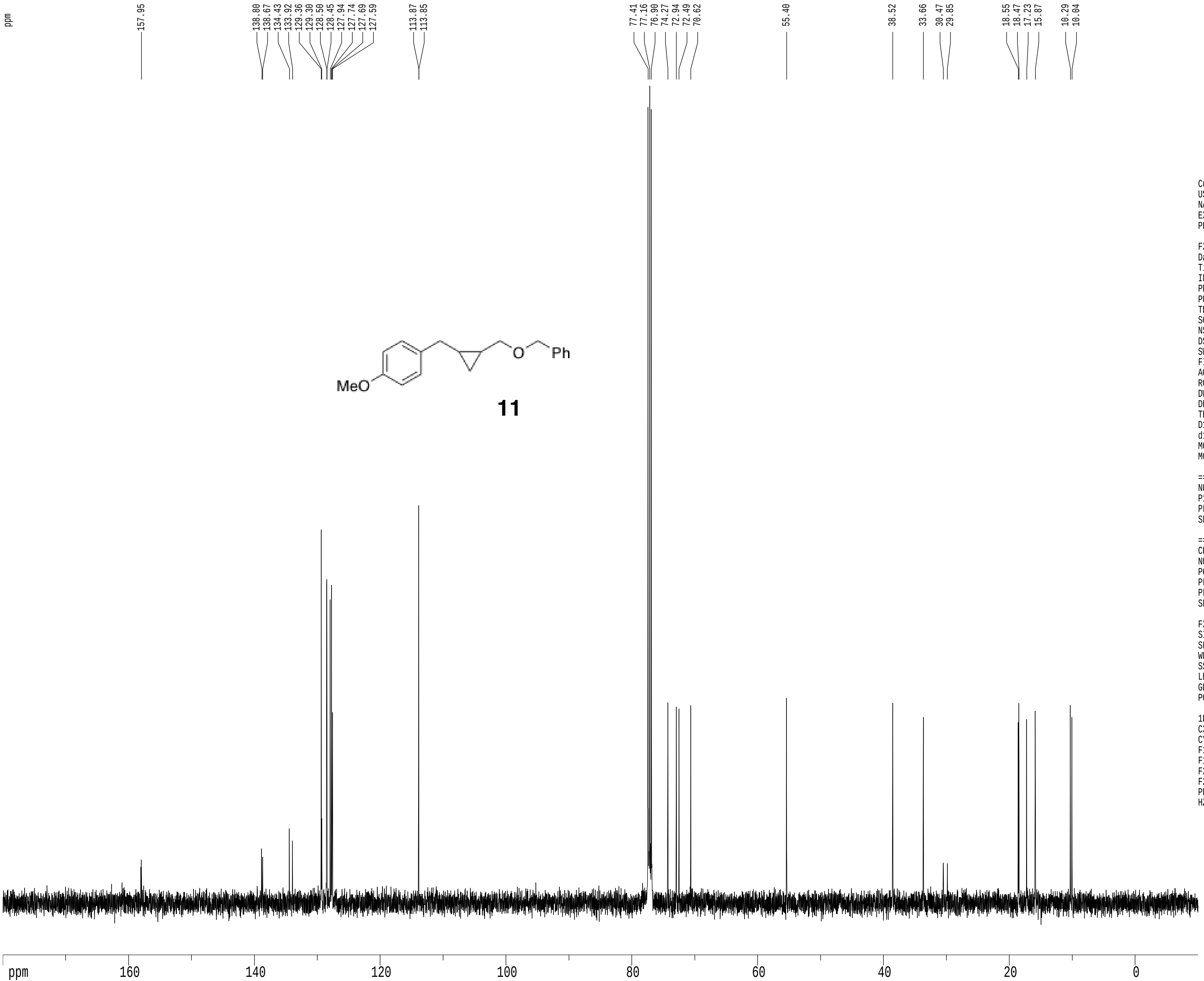

11

```
Current Data Parameters
USER            tthane
NAME        TATv261char
EXPNO                2
PROCNO               1

F2 - Acquisition Parameters
Date_         20211217
Time             13.54
INSTRUM          gn500
PROBHD   5 mm broadband
PULPROG         zgdc30
TD               65536
SOLVENT          CDCl3
NS                 744
DS                   4
SWH          30303.031 Hz
FIDRES        0.462388 Hz
AQ           1.0813940 sec
RG               16384
DW              16.500 usec
DE                6.00 usec
TE               297.9 K
D1          0.25000000 sec
d11         0.03000000 sec
MCREST      0.00000000 sec
MCWRK       0.01500000 sec

======== CHANNEL f1 ========
NUC1               13C
P1               14.20 usec
PL1              -6.00 dB
SFO1       125.3994349 MHz

======== CHANNEL f2 ========
CPDPRG2        waltz16
NUC2                1H
PCPD2           100.00 usec
PL2              -6.00 dB
PL12             12.30 dB
SFO2       498.6524933 MHz

F2 - Processing parameters
SI               65536
SF         125.3856301 MHz
WDW                 EM
SSB                  0
LB                1.00 Hz
GB                   0
PC                2.00

1D NMR plot parameters
CX               22.80 cm
CY               15.65 cm
F1P            180.000 ppm
F1            22569.41 Hz
F2P            -10.000 ppm
F2            -1253.86 Hz
PPMCM          8.33333 ppm/cm
HZCM        1044.88025 Hz/cm
```

1H spectrum

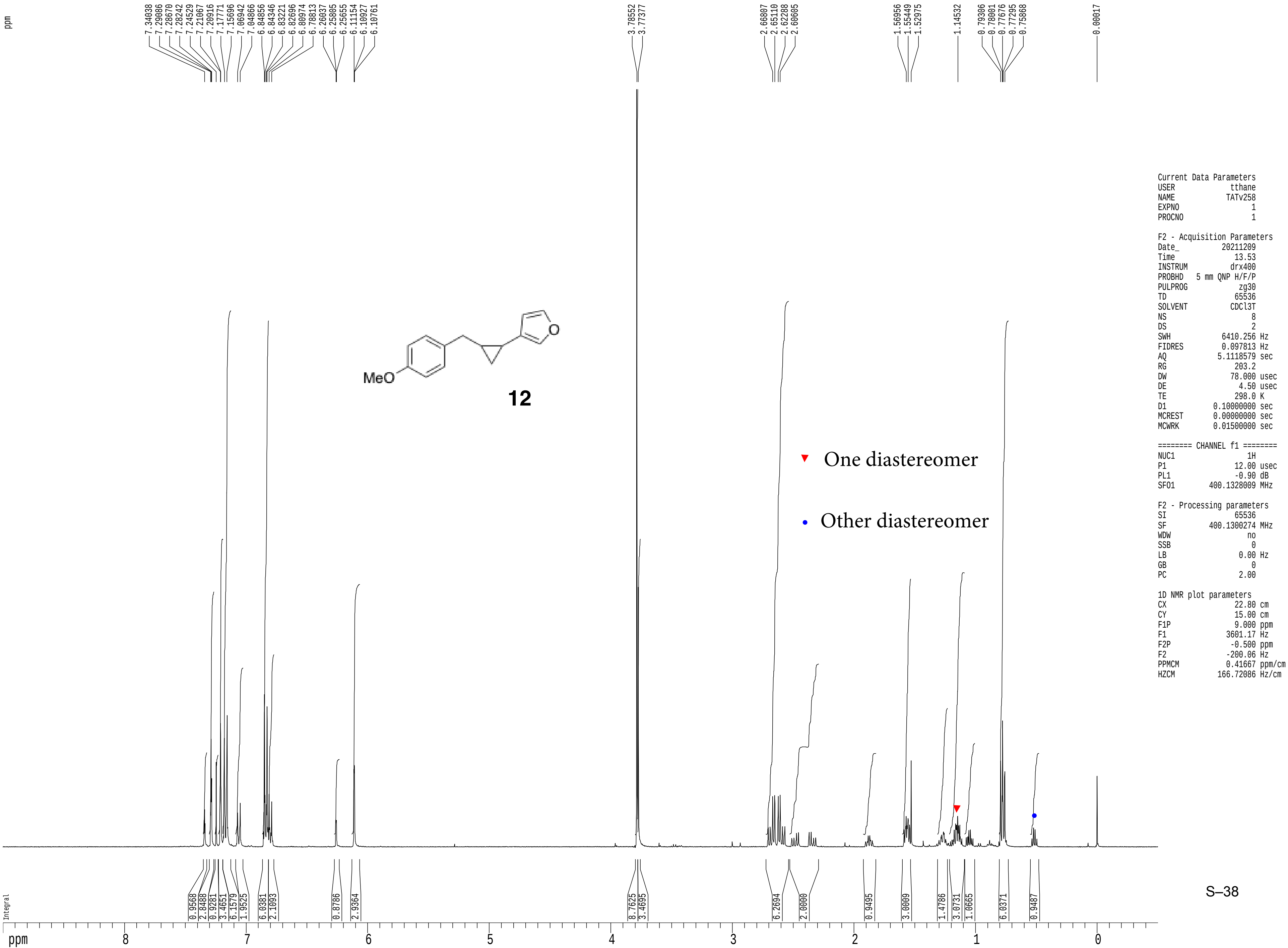
ppm
7.34038 7.29086 7.28670 7.28242 7.24529 7.21067 7.20916 7.17771 7.15696 7.06942 7.04866 6.84856 6.84346 6.83221 6.82696 6.80974 6.78813 6.26037 6.25805 6.25655 6.11154 6.10927 6.10761
3.78552 3.77377
2.66807 2.65110 2.62288 2.60605
1.56956 1.55449 1.52975
1.14532
0.79306 0.78001 0.77676 0.77295 0.75868
0.00017
MeO
O
12
One diastereomer
Other diastereomer
Integral
0.9568 2.8488 0.9281 3.4651 6.1579 1.9525 6.0381 2.1093 0.8786 2.9364 8.7625 3.4695 6.2694 2.0000 0.9495 3.0009 1.4786 3.0731 1.0665 6.0371 0.9487
ppm 8 7 6 5 4 3 2 1 0
Current Data Parameters
USER tthane
NAME TATv258
EXPNO 1
PROCNO 1
F2 - Acquisition Parameters
Date_ 20211209
Time 13.53
INSTRUM drx400
PROBHD 5 mm QNP H/F/P
PULPROG zg30
TD 65536
SOLVENT CDCl3T
NS 8
DS 2
SWH 6410.256 Hz
FIDRES 0.097813 Hz
AQ 5.1118579 sec
RG 203.2
DW 78.000 usec
DE 4.50 usec
TE 298.0 K
D1 0.10000000 sec
MCREST 0.00000000 sec
MCWRK 0.01500000 sec
======== CHANNEL f1 ========
NUC1 1H
P1 12.00 usec
PL1 -0.90 dB
SFO1 400.1328009 MHz
F2 - Processing parameters
SI 65536
SF 400.1300274 MHz
WDW no
SSB 0
LB 0.00 Hz
GB 0
PC 2.00
1D NMR plot parameters
CX 22.80 cm
CY 15.00 cm
F1P 9.000 ppm
F1 3601.17 Hz
F2P -0.500 ppm
F2 -200.06 Hz
PPMCM 0.41667 ppm/cm
HZCM 166.72086 Hz/cm

1H spectrum

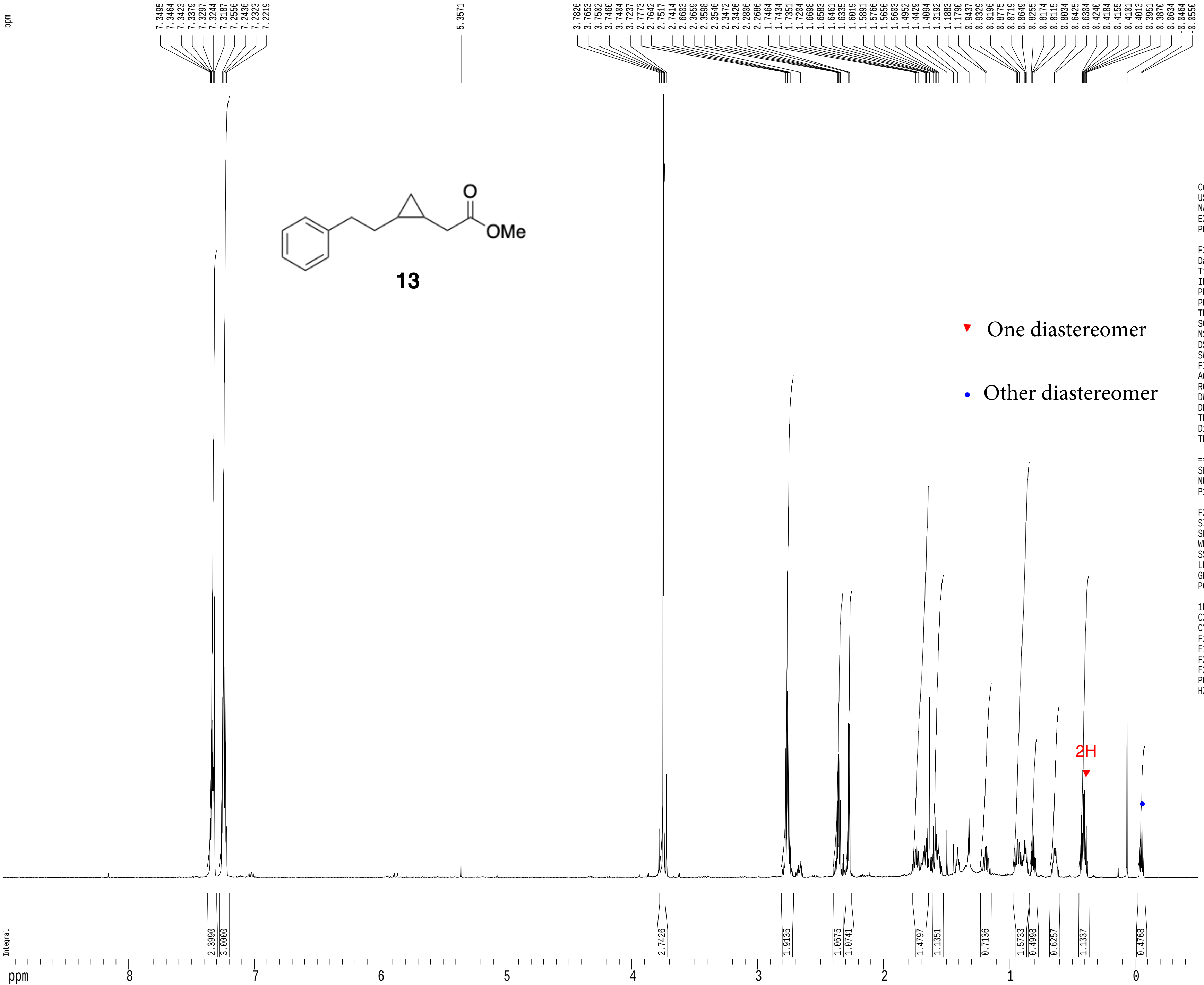

Current Data Parameters
USER mcginnit
NAME tmm-4-169-char
EXPNO 1
PROCNO 1

F2 - Acquisition Parameters
Date_ 20220418
Time 10.15
INSTRUM av600
PROBHD 5 mm CPBBO BB-
PULPROG zg30
TD 98074
SOLVENT CDCl3
NS 8
DS 2
SWH 9615.385 Hz
FIDRES 0.098042 Hz
AQ 5.0998979 sec
RG 10
DW 52.000 usec
DE 14.23 usec
TE 298.0 K
D1 0.10000000 sec
TD0 1

======== CHANNEL f1 ========
SFO1 600.1342009 MHz
NUC1 1H
P1 9.50 usec

F2 - Processing parameters
SI 65536
SF 600.1300000 MHz
WDW no
SSB 0
LB 0.00 Hz
GB 0
PC 1.00

1D NMR plot parameters
CX 22.80 cm
CY 15.00 cm
F1P 9.000 ppm
F1 5401.17 Hz
F2P -0.500 ppm
F2 -300.06 Hz
PPMCM 0.41667 ppm/cm
HZCM 250.05418 Hz/cm

13C spectrum

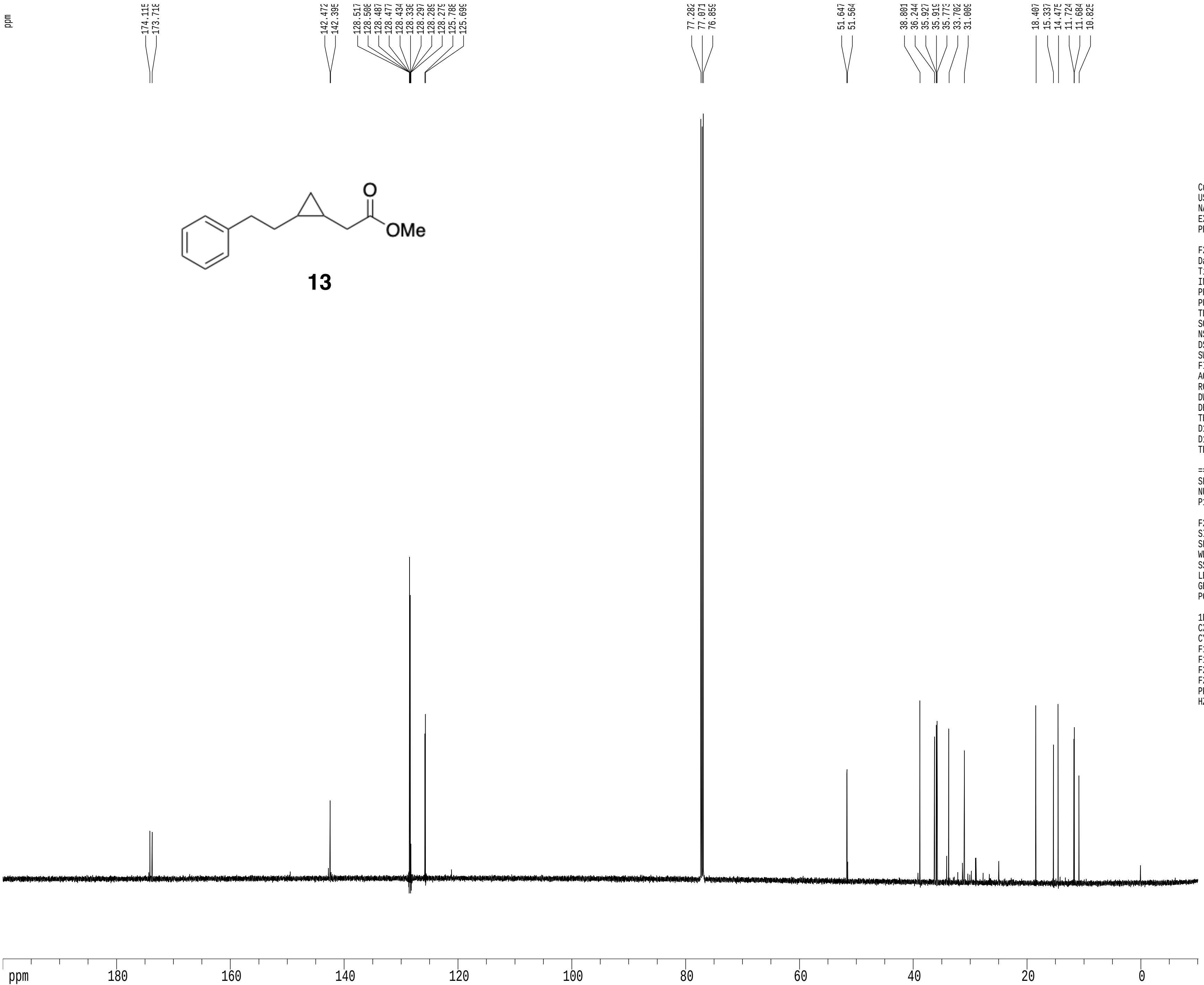


```
Current Data Parameters
USER          mcginnit
NAME     tmm-4-169-char
EXPNO                2
PROCNO               1

F2 - Acquisition Parameters
Date_         20220418
Time             10.20
INSTRUM          av600
PROBHD   5 mm CPBBO BB-
PULPROG         zgdc30
TD               65536
SOLVENT          CDCl3
NS                 209
DS                   4
SWH          36231.883 Hz
FIDRES        0.552855 Hz
AQ           0.9044468 sec
RG                2050
DW              13.800 usec
DE               19.63 usec
TE               298.0 K
D1          0.40000001 sec
D11         0.03000000 sec
TD0                  1

======== CHANNEL f1 ========
SFO1       150.9194080 MHz
NUC1               13C
P1               10.10 usec

F2 - Processing parameters
SI               65536
SF         150.9028085 MHz
WDW                 no
SSB                  0
LB                0.00 Hz
GB                   0
PC                1.00

1D NMR plot parameters
CX               22.80 cm
CY               15.00 cm
F1P            200.000 ppm
F1            30180.56 Hz
F2P            -10.049 ppm
F2            -1516.46 Hz
PPMCM          9.21269 ppm/cm
HZCM        1390.22034 Hz/cm
```

1H spectrum

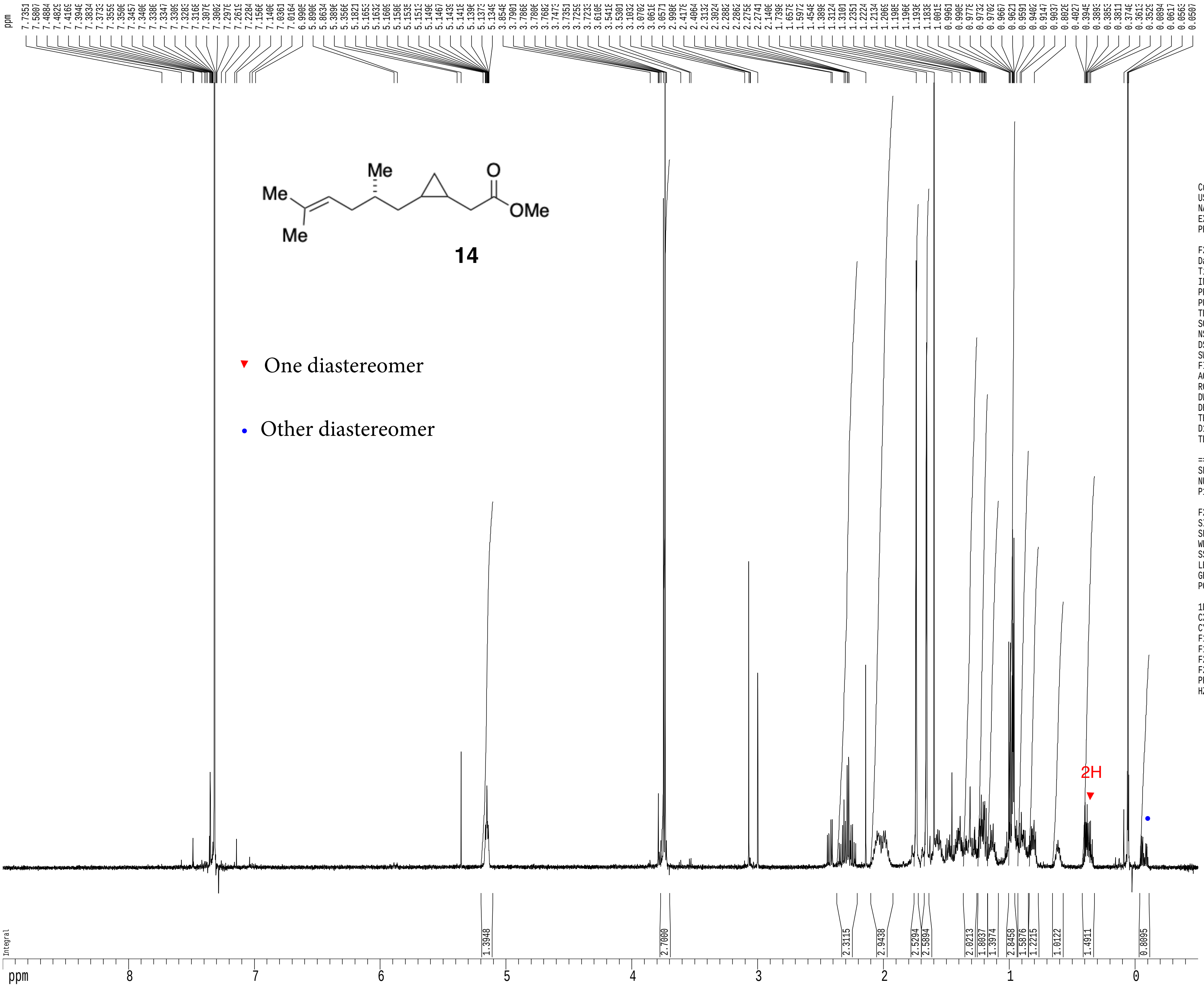

Current Data Parameters
USER mcginnit
NAME tmm-4-087-char
EXPNO 1
PROCNO 1

F2 - Acquisition Parameters
Date_ 20220225
Time 13.54
INSTRUM av600
PROBHD 5 mm CPBBO BB-
PULPROG zg30
TD 98074
SOLVENT CDCl3
NS 16
DS 2
SWH 9615.385 Hz
FIDRES 0.098042 Hz
AQ 5.0998979 sec
RG 16
DW 52.000 usec
DE 14.23 usec
TE 298.0 K
D1 0.10000000 sec
TD0 1

======== CHANNEL f1 ========
SFO1 600.1342009 MHz
NUC1 1H
P1 9.50 usec

F2 - Processing parameters
SI 65536
SF 600.1300000 MHz
WDW no
SSB 0
LB 0.00 Hz
GB 0
PC 1.00

1D NMR plot parameters
CX 22.80 cm
CY 100.00 cm
F1P 9.000 ppm
F1 5401.17 Hz
F2P -0.500 ppm
F2 -300.06 Hz
PPMCM 0.41667 ppm/cm
HZCM 250.05418 Hz/cm

13C spectrum

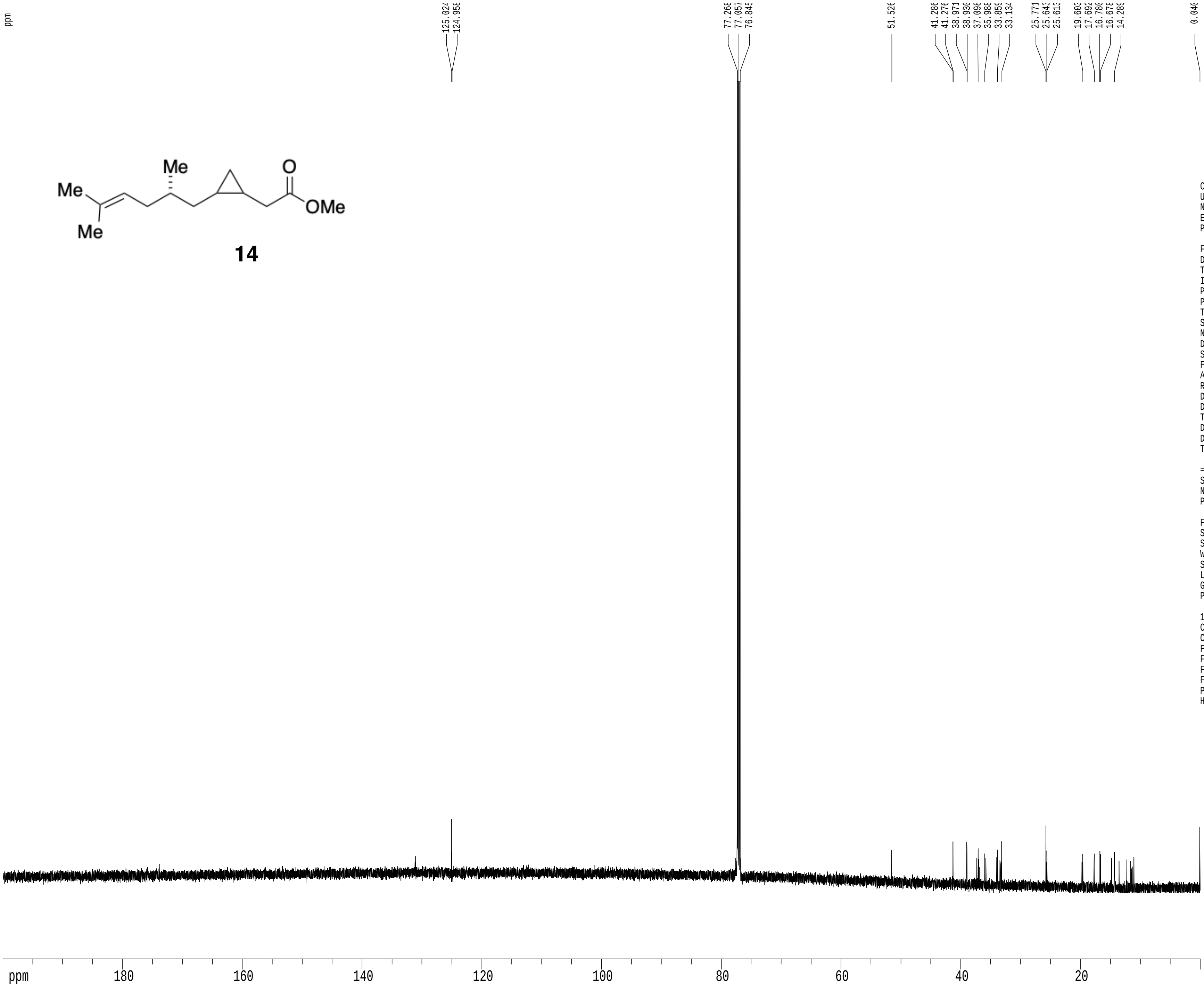

14

Current Data Parameters
USER mcginnit
NAME tmm-4-087-char
EXPNO 2
PROCNO 1

F2 - Acquisition Parameters
Date_ 20220225
Time 14.01
INSTRUM av600
PROBHD 5 mm CPBBO BB-
PULPROG zgdc30
TD 65536
SOLVENT CDCl3
NS 869
DS 4
SWH 36231.883 Hz
FIDRES 0.552855 Hz
AQ 0.9044468 sec
RG 2050
DW 13.800 usec
DE 19.63 usec
TE 298.0 K
D1 0.40000001 sec
D11 0.03000000 sec
TD0 1

======== CHANNEL f1 ========
SFO1 150.9194080 MHz
NUC1 13C
P1 10.10 usec

F2 - Processing parameters
SI 65536
SF 150.9028085 MHz
WDW no
SSB 0
LB 0.00 Hz
GB 0
PC 1.00

1D NMR plot parameters
CX 22.80 cm
CY 40.00 cm
F1P 200.000 ppm
F1 30180.56 Hz
F2P 0.000 ppm
F2 0.00 Hz
PPMCM 8.77193 ppm/cm
HZCM 1323.70886 Hz/cm

1H spectrum

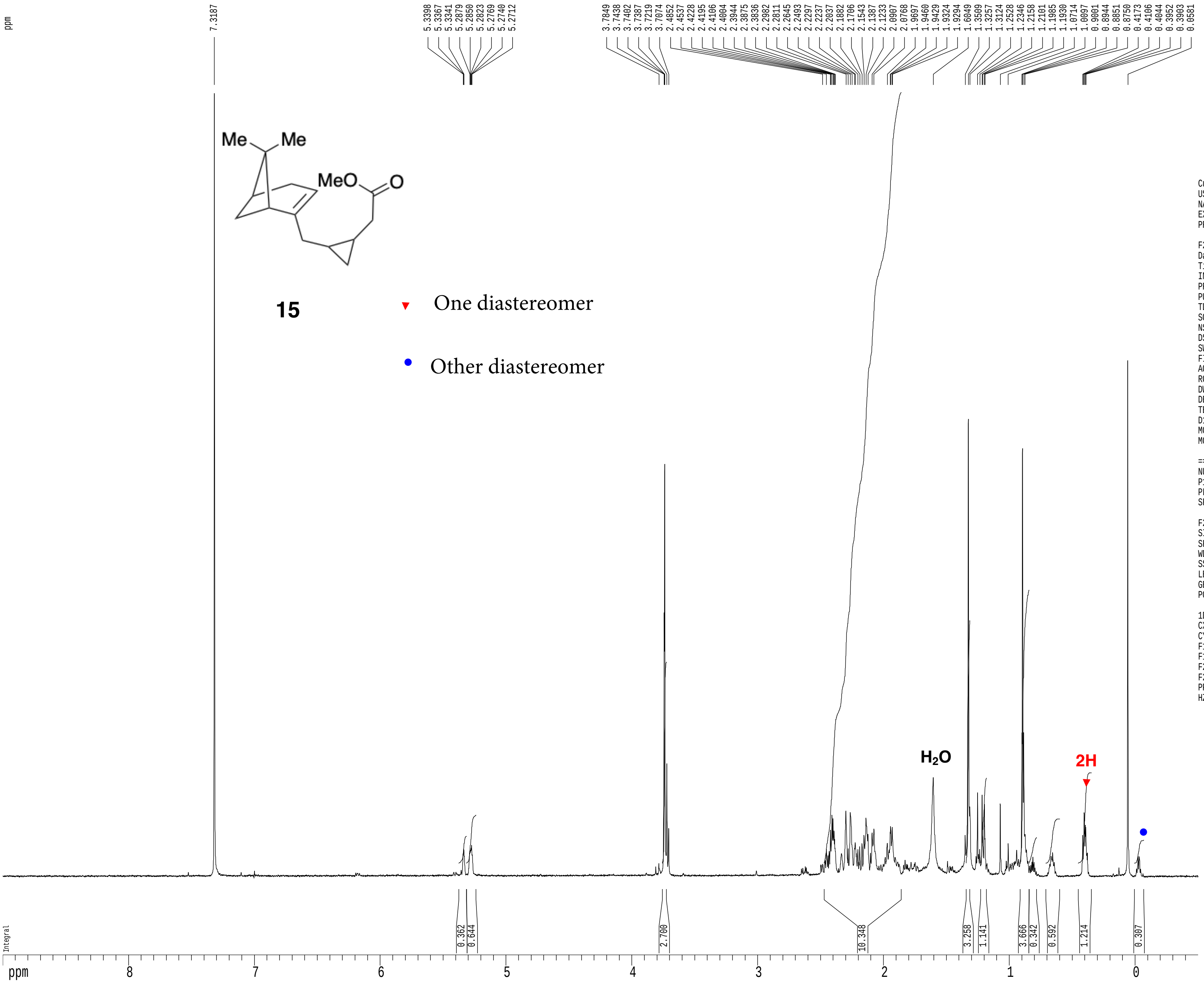

Current Data Parameters
USER mcginnit
NAME tmm-4-nopol
EXPNO 1
PROCNO 1

F2 - Acquisition Parameters
Date_ 20220706
Time 17.14
INSTRUM gn500
PROBHD 5 mm broadband
PULPROG zg30
TD 81728
SOLVENT CDCl3
NS 8
DS 2
SWH 8012.820 Hz
FIDRES 0.098043 Hz
AQ 5.0998774 sec
RG 1824.6
DW 62.400 usec
DE 6.00 usec
TE 298.0 K
D1 0.10000000 sec
MCREST 0.00000000 sec
MCWRK 0.01500000 sec

======== CHANNEL f1 ========
NUC1 1H
P1 12.00 usec
PL1 -6.00 dB
SFO1 498.6534906 MHz

F2 - Processing parameters
SI 65536
SF 498.6500000 MHz
WDW EM
SSB 0
LB 0.30 Hz
GB 0
PC 1.00

1D NMR plot parameters
CX 22.80 cm
CY 15.00 cm
F1P 9.000 ppm
F1 4487.85 Hz
F2P -0.500 ppm
F2 -249.32 Hz
PPMCM 0.41667 ppm/cm
HZCM 207.77084 Hz/cm

Z-restored spin-echo 13C spectrum with 1H decoupling

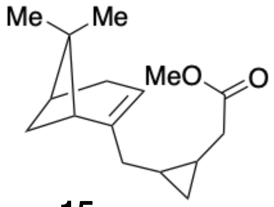

15

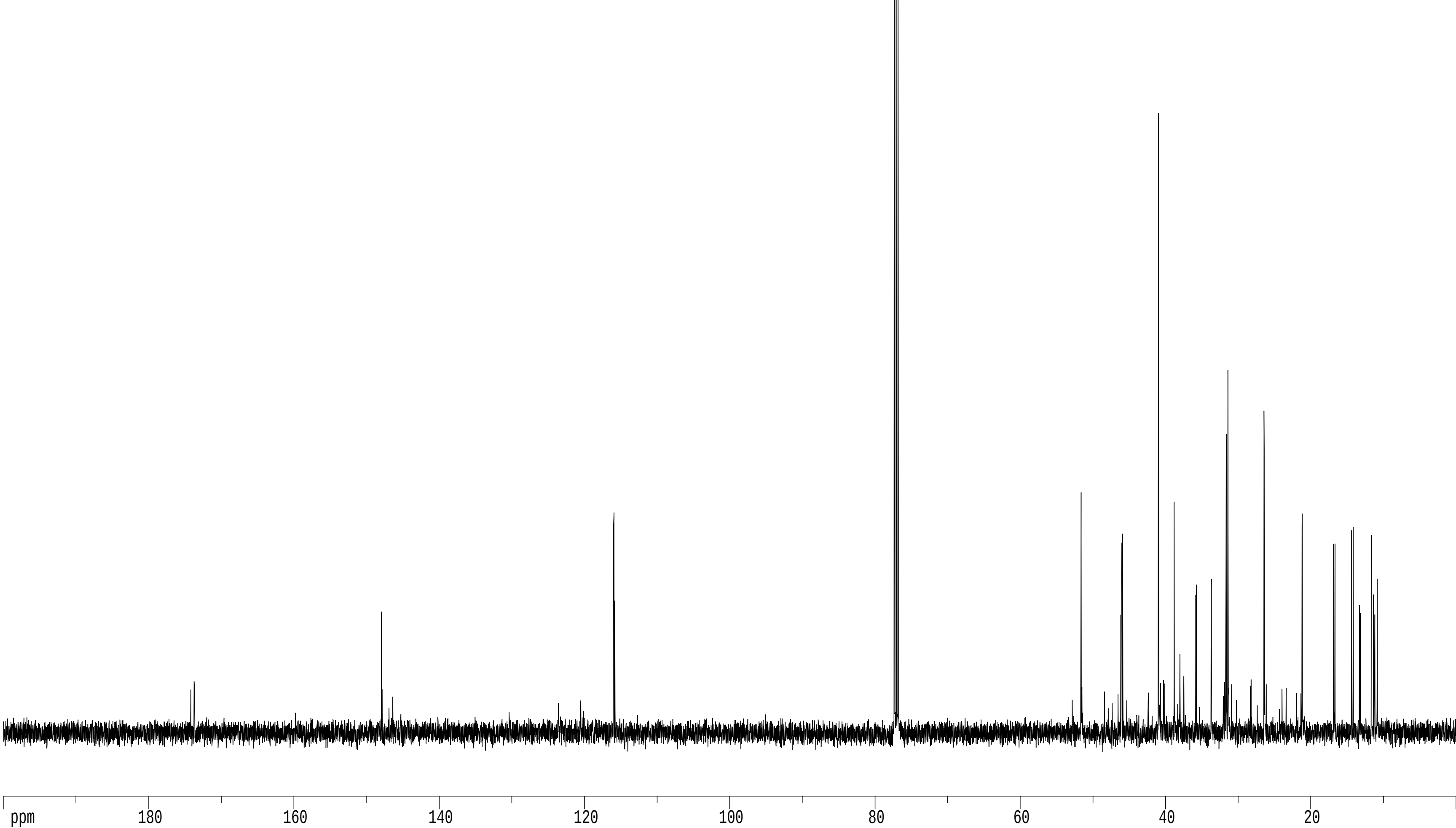

Current Data Parameters
USER mcginnit
NAME tmm-4-116-char
EXPNO 2
PROCNO 1

F2 - Acquisition Parameters
Date_ 20220304
Time 13.49
INSTRUM cryo500
PROBHD 5 mm CPTCI 1H-
PULPROG SpinEchopg30gp2.prd
TD 65536
SOLVENT CDCl3
NS 206
DS 16
SWH 30303.031 Hz
FIDRES 0.462388 Hz
AQ 1.0813940 sec
RG 7298.2
DW 16.500 usec
DE 6.00 usec
TE 298.0 K
D1 0.25000000 sec
d11 0.03000000 sec
D16 0.00020000 sec
d17 0.00019600 sec
MCREST 0.00000000 sec
MCWRK 0.01500000 sec
P2 37.70 usec

======== CHANNEL f1 ========
NUC1 13C
P1 18.85 usec
P12 2000.00 usec
P20 500.00 usec
PL0 120.00 dB
PL1 -1.00 dB
SFO1 125.7942548 MHz
SP2 1.55 dB
SP4 1.55 dB
SPNAM2 Crp60comp.4
SPNAM4 Crp60,0.5,20.1
SPOFF2 0.00 Hz
SPOFF4 0.00 Hz

======== CHANNEL f2 ========
CPDPRG2 waltz16
NUC2 1H
PCPD2 100.00 usec
PL2 1.60 dB
PL12 22.00 dB
SFO2 500.2225011 MHz

====== GRADIENT CHANNEL =====
GPNAM1 SINE.100
GPNAM2 SINE.100
GPX1 0.00 %
GPX2 0.00 %
GPY1 0.00 %
GPY2 0.00 %
GPZ1 30.00 %
GPZ2 50.00 %
p15 500.00 usec
p16 1000.00 usec

F2 - Processing parameters
SI 65536
SF 125.7804190 MHz
WDW EM
SSB 0
LB 1.00 Hz
GB 0
PC 2.00

1D NMR plot parameters
CX 22.80 cm
CY 15.65 cm
F1P 200.000 ppm
F1 25156.08 Hz
F2P 0.000 ppm
F2 0.00 Hz
PPMCM 8.77193 ppm/cm
HZCM 1103.33704 Hz/cm

1H spectrum

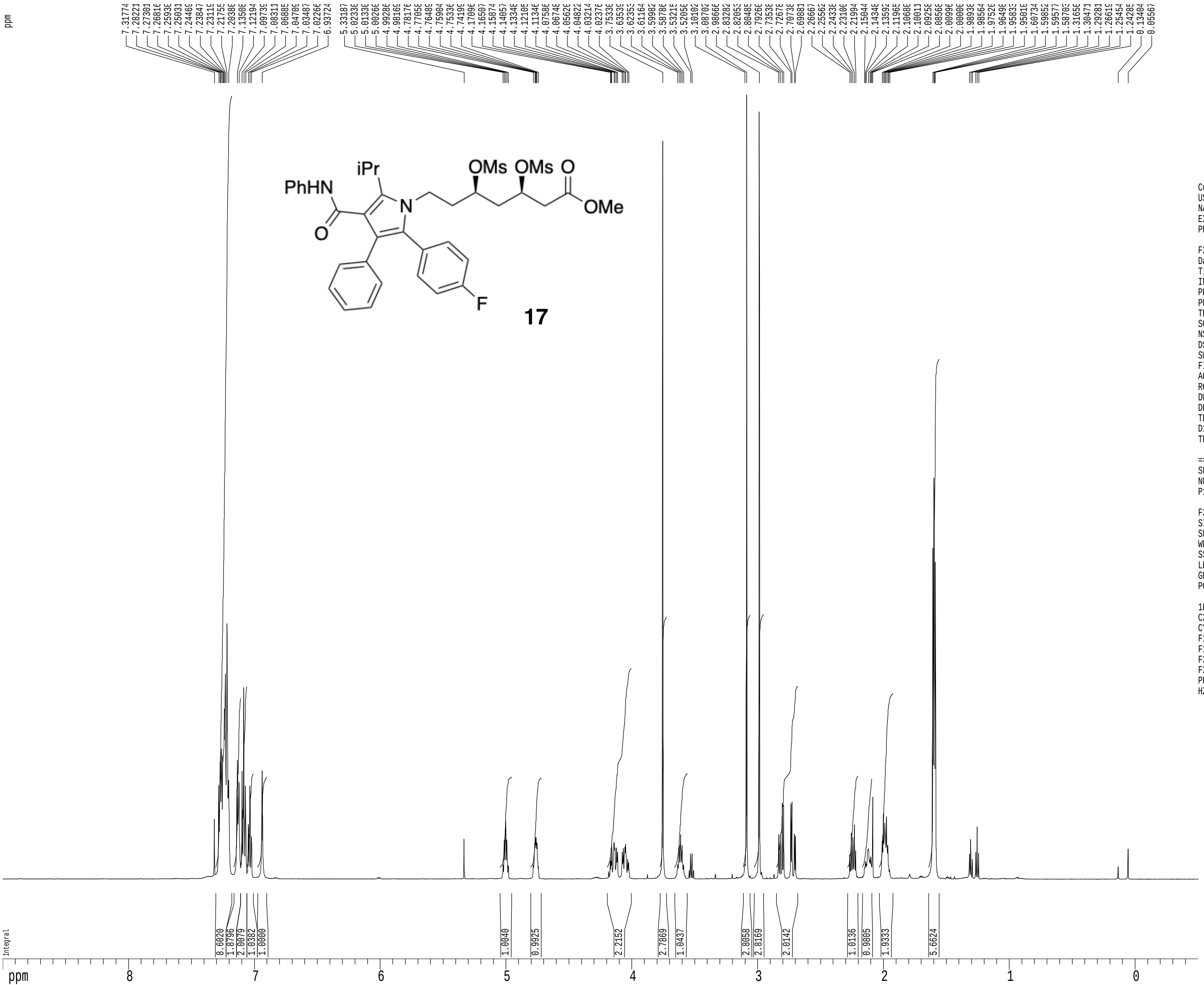


```
Current Data Parameters
USER          mcginnit
NAME     tmm-3-282-char
EXPNO                1
PROCNO               1

F2 - Acquisition Parameters
Date_         20220224
Time             15.48
INSTRUM          av600
PROBHD   5 mm CPBBO BB-
PULPROG           zg30
TD               98074
SOLVENT          CDCl3
NS                   8
DS                   2
SWH           9615.385 Hz
FIDRES        0.098042 Hz
AQ           5.0998979 sec
RG                   8
DW              52.000 usec
DE               14.23 usec
TE               298.0 K
D1          0.10000000 sec
TD0                  1

======== CHANNEL f1 ========
SFO1       600.1342009 MHz
NUC1                1H
P1                9.50 usec

F2 - Processing parameters
SI               65536
SF         600.1300000 MHz
WDW                 no
SSB                  0
LB                0.00 Hz
GB                   0
PC                1.00

1D NMR plot parameters
CX               22.80 cm
CY               15.00 cm
F1P              9.000 ppm
F1             5401.17 Hz
F2P             -0.500 ppm
F2             -300.06 Hz
PPMCM          0.41667 ppm/cm
HZCM          250.05418 Hz/cm
```

13Cspectrum

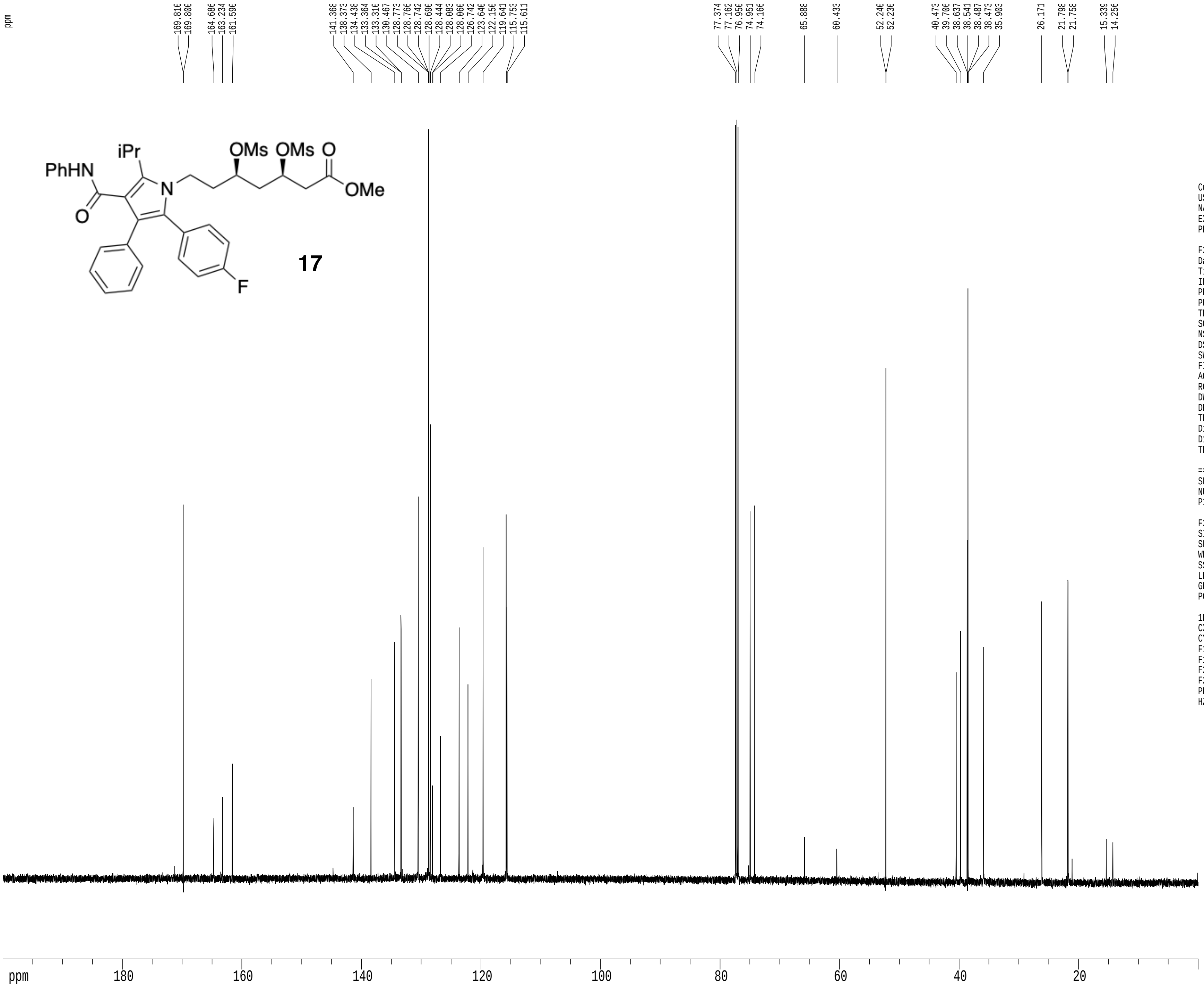


```
Current Data Parameters
USER            mcginnit
NAME      tmm-3-282-char
EXPNO                  2
PROCNO                 1

F2 - Acquisition Parameters
Date_           20220224
Time               15.50
INSTRUM            av600
PROBHD   5 mm CPBBO BB-
PULPROG          zgdc30
TD                 65536
SOLVENT            CDCl3
NS                    80
DS                     4
SWH           36231.883 Hz
FIDRES         0.552855 Hz
AQ            0.9044468 sec
RG                  2050
DW                13.800 usec
DE                 19.63 usec
TE                 297.9 K
D1            0.40000001 sec
D11           0.03000000 sec
TD0                    1

======== CHANNEL f1 ========
SFO1        150.9194080 MHz
NUC1                 13C
P1                 10.10 usec

F2 - Processing parameters
SI                 65536
SF          150.9028085 MHz
WDW                   no
SSB                    0
LB                  0.00 Hz
GB                     0
PC                  1.00

1D NMR plot parameters
CX                 22.80 cm
CY                 15.00 cm
F1P              200.000 ppm
F1              30180.56 Hz
F2P                0.000 ppm
F2                  0.00 Hz
PPMCM            8.77193 ppm/cm
HZCM          1323.70886 Hz/cm
```

19F spectrum

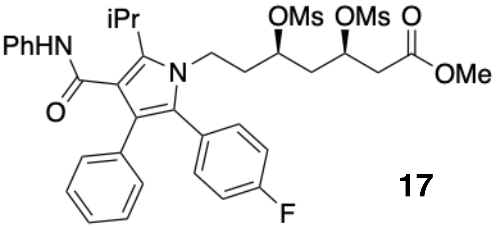

17

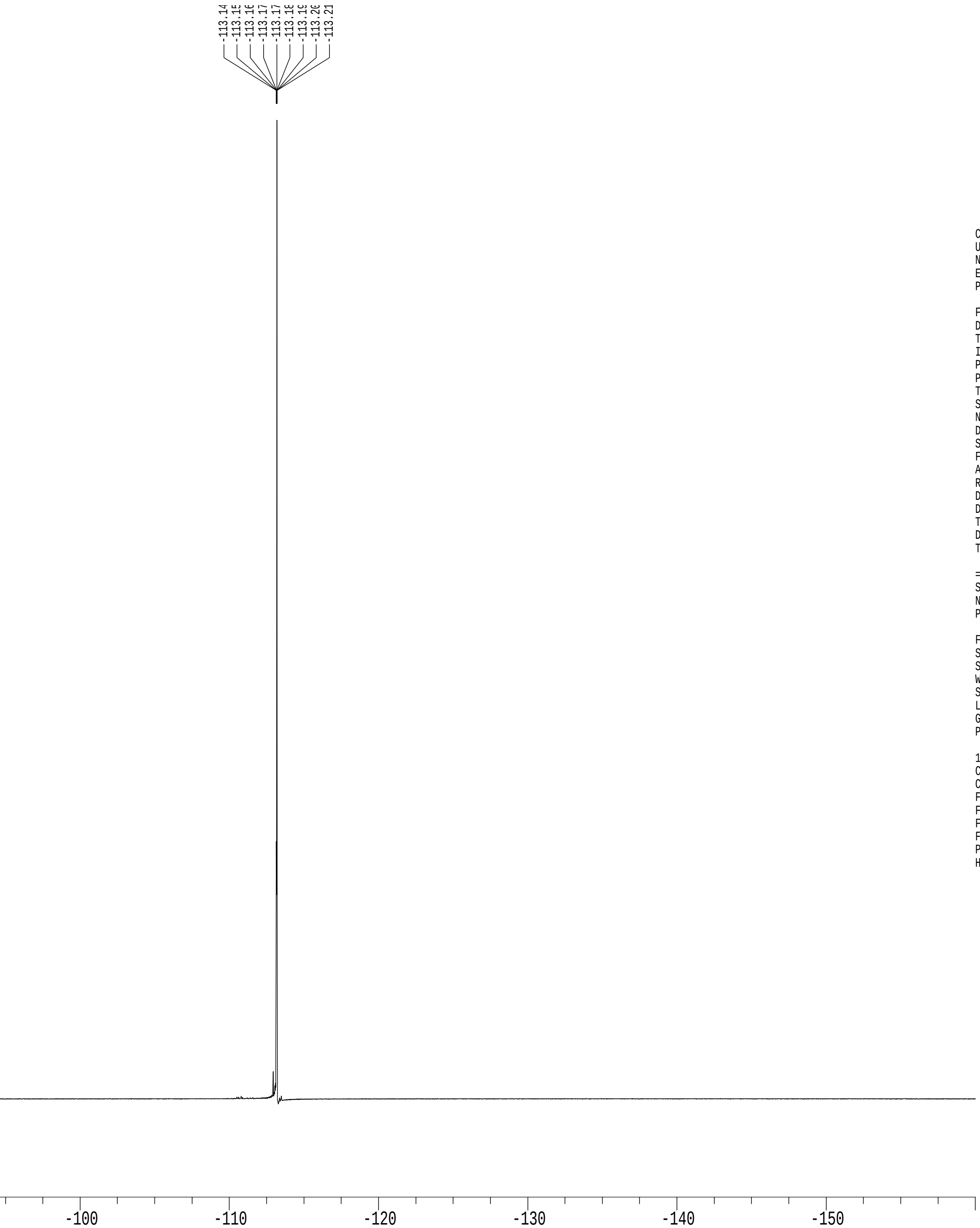


```
Current Data Parameters
USER           mcginnit
NAME     tmm-3-282-char
EXPNO                 3
PROCNO                1

F2 - Acquisition Parameters
Date_          20220224
Time              15.57
INSTRUM           av600
PROBHD   5 mm CPBBO BB-
PULPROG        zgflqn30
TD               131072
SOLVENT           CDCl3
NS                   16
DS                    2
SWH          178571.422 Hz
FIDRES         1.362392 Hz
AQ            0.3670516 sec
RG                  575
DW                2.800 usec
DE                18.00 usec
TE                297.9 K
D1           3.00000000 sec
TD0                   1

======== CHANNEL f1 ========
SFO1        564.6299196 MHz
NUC1                19F
P1                18.25 usec

F2 - Processing parameters
SI               131072
SF          564.6863858 MHz
WDW                  no
SSB                   0
LB                 0.00 Hz
GB                    0
PC                 1.00

1D NMR plot parameters
CX                22.80 cm
CY                15.00 cm
F1P             -60.000 ppm
F1            -33881.19 Hz
F2P            -160.000 ppm
F2            -90349.83 Hz
PPMCM           4.38596 ppm/cm
HZCM         2476.69482 Hz/cm
```

1H spectrum

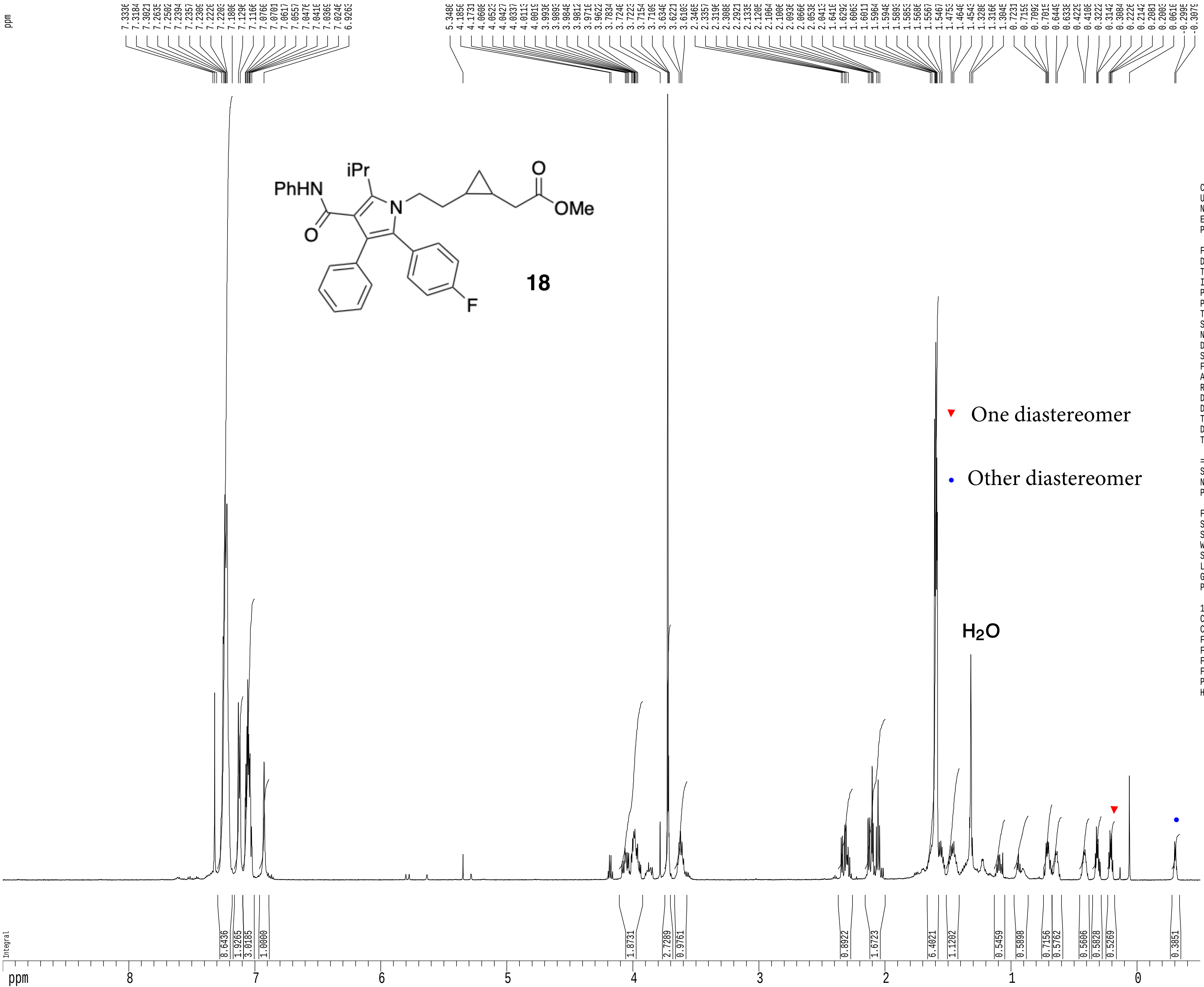

Current Data Parameters
USER mcginnit
NAME tmm-3-285b
EXPNO 11
PROCNO 1

F2 - Acquisition Parameters
Date_ 20211203
Time 17.22
INSTRUM av600
PROBHD 5 mm CPBBO BB-
PULPROG zg30
TD 98074
SOLVENT CDCl3
NS 8
DS 2
SWH 9615.385 Hz
FIDRES 0.098042 Hz
AQ 5.0998979 sec
RG 10
DW 52.000 usec
DE 14.23 usec
TE 298.0 K
D1 0.10000000 sec
TD0 1

======== CHANNEL f1 ========
SFO1 600.1342009 MHz
NUC1 1H
P1 9.50 usec

F2 - Processing parameters
SI 65536
SF 600.1300000 MHz
WDW no
SSB 0
LB 0.00 Hz
GB 0
PC 1.00

1D NMR plot parameters
CX 22.80 cm
CY 15.00 cm
F1P 9.000 ppm
F1 5401.17 Hz
F2P -0.500 ppm
F2 -300.06 Hz
PPMCM 0.41667 ppm/cm
HZCM 250.05418 Hz/cm

13C spectrum

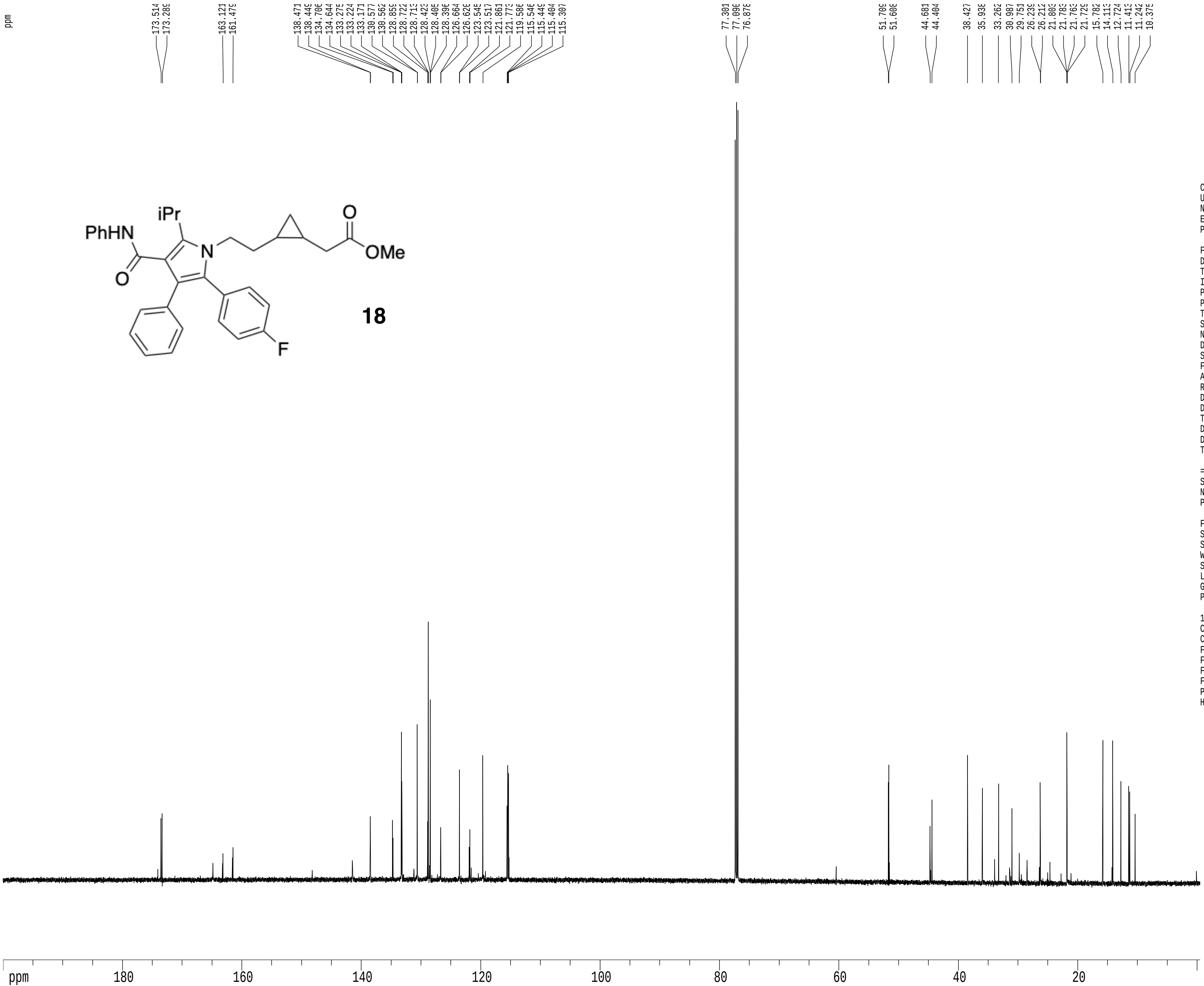


```
Current Data Parameters
USER          mcginnit
NAME         tmm-3-285b
EXPNO               12
PROCNO               1

F2 - Acquisition Parameters
Date_         20211203
Time             17.27
INSTRUM          av600
PROBHD   5 mm CPBBO BB-
PULPROG         zgdc30
TD               65536
SOLVENT          CDCl3
NS                 258
DS                   4
SWH          36231.883 Hz
FIDRES        0.552855 Hz
AQ           0.9044468 sec
RG                2050
DW              13.800 usec
DE               19.63 usec
TE               297.9 K
D1          0.40000001 sec
D11         0.03000000 sec
TD0                  1

======== CHANNEL f1 ========
SFO1       150.9194080 MHz
NUC1               13C
P1               10.10 usec

F2 - Processing parameters
SI               65536
SF         150.9028085 MHz
WDW                 no
SSB                  0
LB                0.00 Hz
GB                   0
PC                1.00

1D NMR plot parameters
CX               22.80 cm
CY               15.00 cm
F1P            200.000 ppm
F1            30180.56 Hz
F2P             -0.500 ppm
F2              -75.45 Hz
PPMCM          8.79386 ppm/cm
HZCM        1327.01807 Hz/cm
```

19F spectrum

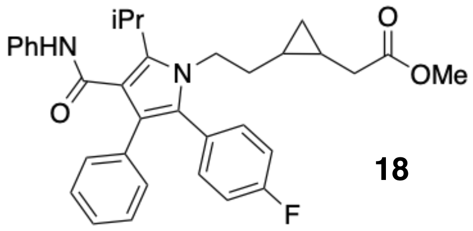

18

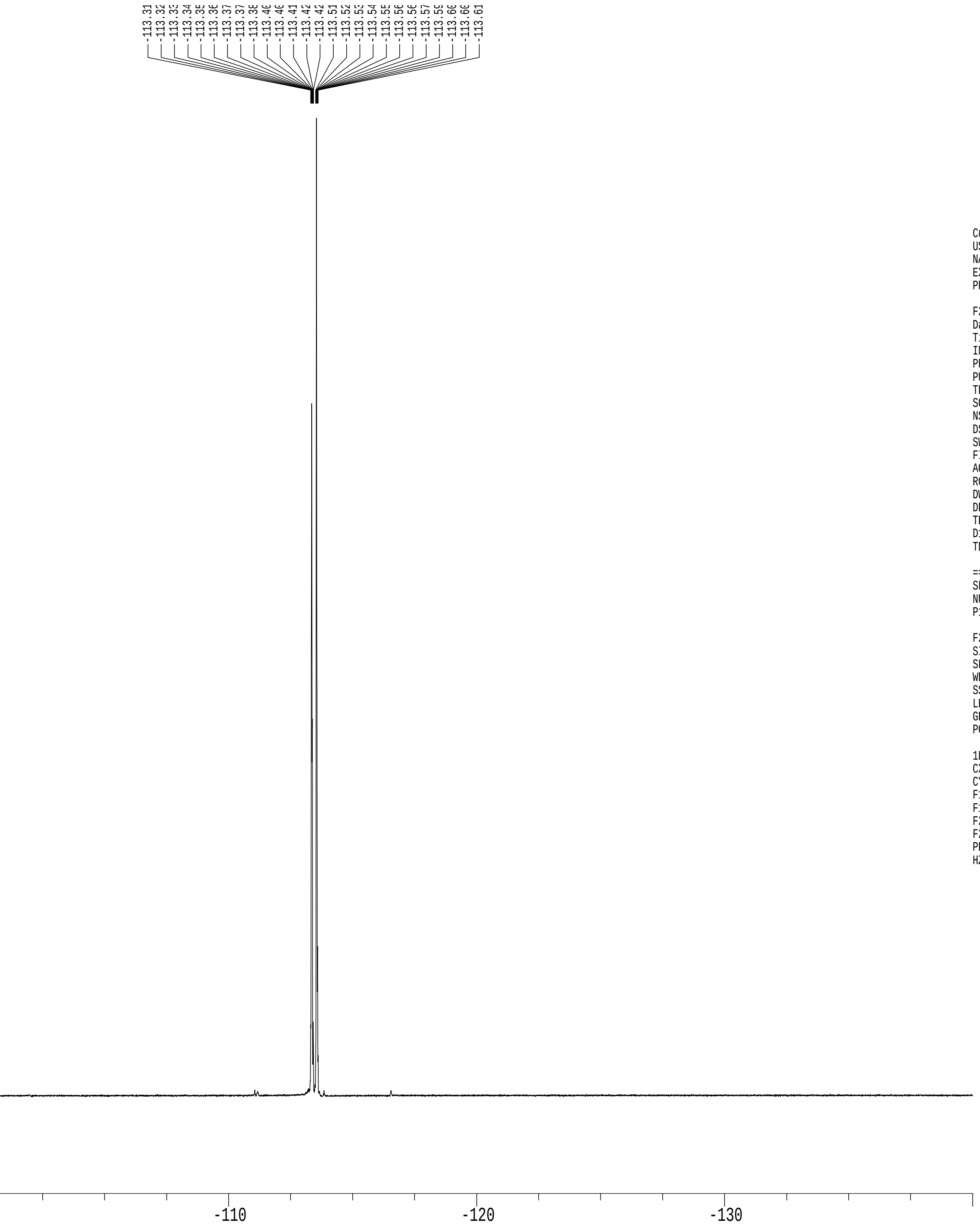


```
Current Data Parameters
USER          mcginnit
NAME        tmm-3-285b
EXPNO               13
PROCNO               1

F2 - Acquisition Parameters
Date_         20211203
Time             17.35
INSTRUM          av600
PROBHD   5 mm CPBBO BB-
PULPROG       zgflqn30
TD              131072
SOLVENT          CDCl3
NS                  16
DS                   2
SWH         178571.422 Hz
FIDRES        1.362392 Hz
AQ           0.3670516 sec
RG                 575
DW               2.800 usec
DE               18.00 usec
TE               297.9 K
D1          3.00000000 sec
TD0                  1

======== CHANNEL f1 ========
SFO1       564.6299196 MHz
NUC1               19F
P1               18.25 usec

F2 - Processing parameters
SI              131072
SF         564.6863858 MHz
WDW                 no
SSB                  0
LB                0.00 Hz
GB                   0
PC                1.00

1D NMR plot parameters
CX               22.80 cm
CY               15.00 cm
F1P            -80.000 ppm
F1           -45174.91 Hz
F2P           -140.000 ppm
F2           -79056.09 Hz
PPMCM          2.63158 ppm/cm
HZCM        1486.01685 Hz/cm
```

gcosy60

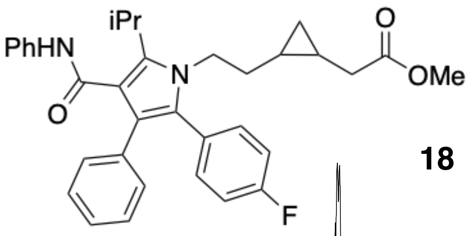

18

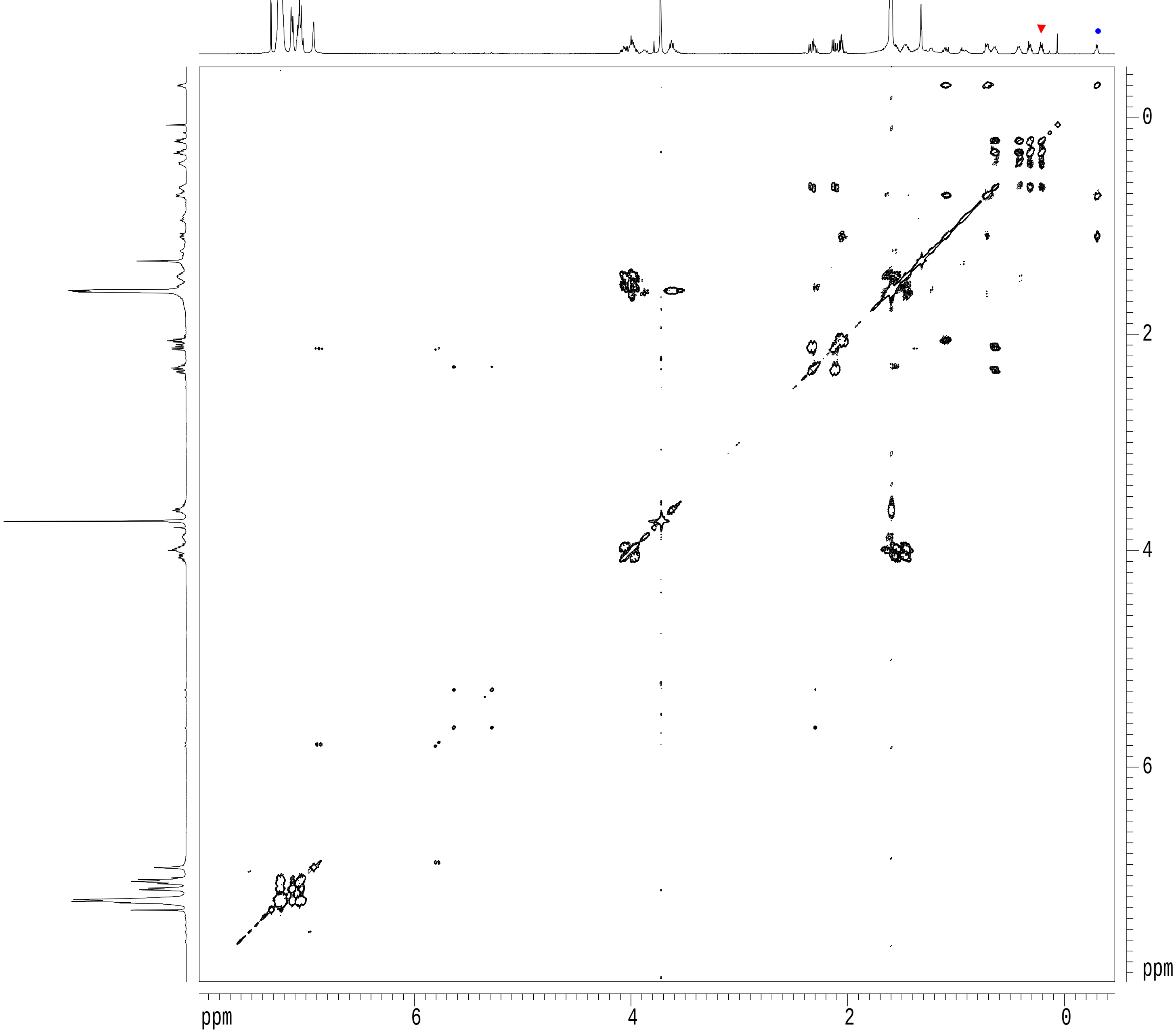

Current Data Parameters
USER mcginnit
NAME tmm-3-285b-cosy
EXPNO 2
PROCNO 1

F2 - Acquisition Parameters
Date_ 20220314
Time 8.22
INSTRUM cryo500
PROBHD 5 mm CPTCI 1H-
PULPROG cosygp60.prd
TD 2048
SOLVENT CDCl3
NS 1
DS 16
SWH 4734.849 Hz
FIDRES 2.311938 Hz
AQ 0.2163188 sec
RG 35.9
DW 105.600 usec
DE 6.00 usec
TE 298.0 K
d0 0.00000300 sec
D1 1.00000000 sec
d13 0.00000300 sec
D16 0.00020000 sec
IN0 0.00021120 sec

======== CHANNEL f1 ========
NUC1 1H
P1 9.75 usec
PL1 1.60 dB
SFO1 500.2221259 MHz

====== GRADIENT CHANNEL =====
GPNAM1 SMSQ10.100
GPNAM2 SMSQ10.100
GPX1 0.00 %
GPX2 0.00 %
GPY1 0.00 %
GPY2 0.00 %
GPZ1 17.00 %
GPZ2 17.00 %
P16 1000.00 usec

F1 - Acquisition parameters
ND0 1
TD 512
SFO1 500.2221 MHz
FIDRES 9.247751 Hz
SW 9.465 ppm
FnMODE QF

F2 - Processing parameters
SI 1024
SF 500.2200000 MHz
WDW SINE
SSB 0
LB 0.00 Hz
GB 0
PC 1.00

F1 - Processing parameters
SI 1024
MC2 QF
SF 500.2200000 MHz
WDW SINE
SSB 0
LB 0.00 Hz
GB 0

2D NMR plot parameters
CX2 15.00 cm
CX1 15.00 cm
F2PLO 7.984 ppm
F2LO 3993.98 Hz
F2PHI -0.464 ppm
F2HI -232.24 Hz
F1PLO 7.984 ppm
F1LO 3993.98 Hz
F1PHI -0.474 ppm
F1HI -23[illegible] Hz
F2PPMCM 0.56325 ppm/cm
F2HZCM 281.74814 Hz/cm
F1PPMCM 0.56386 ppm/cm
F1HZCM 282.05640 Hz/cm

1H spectrum

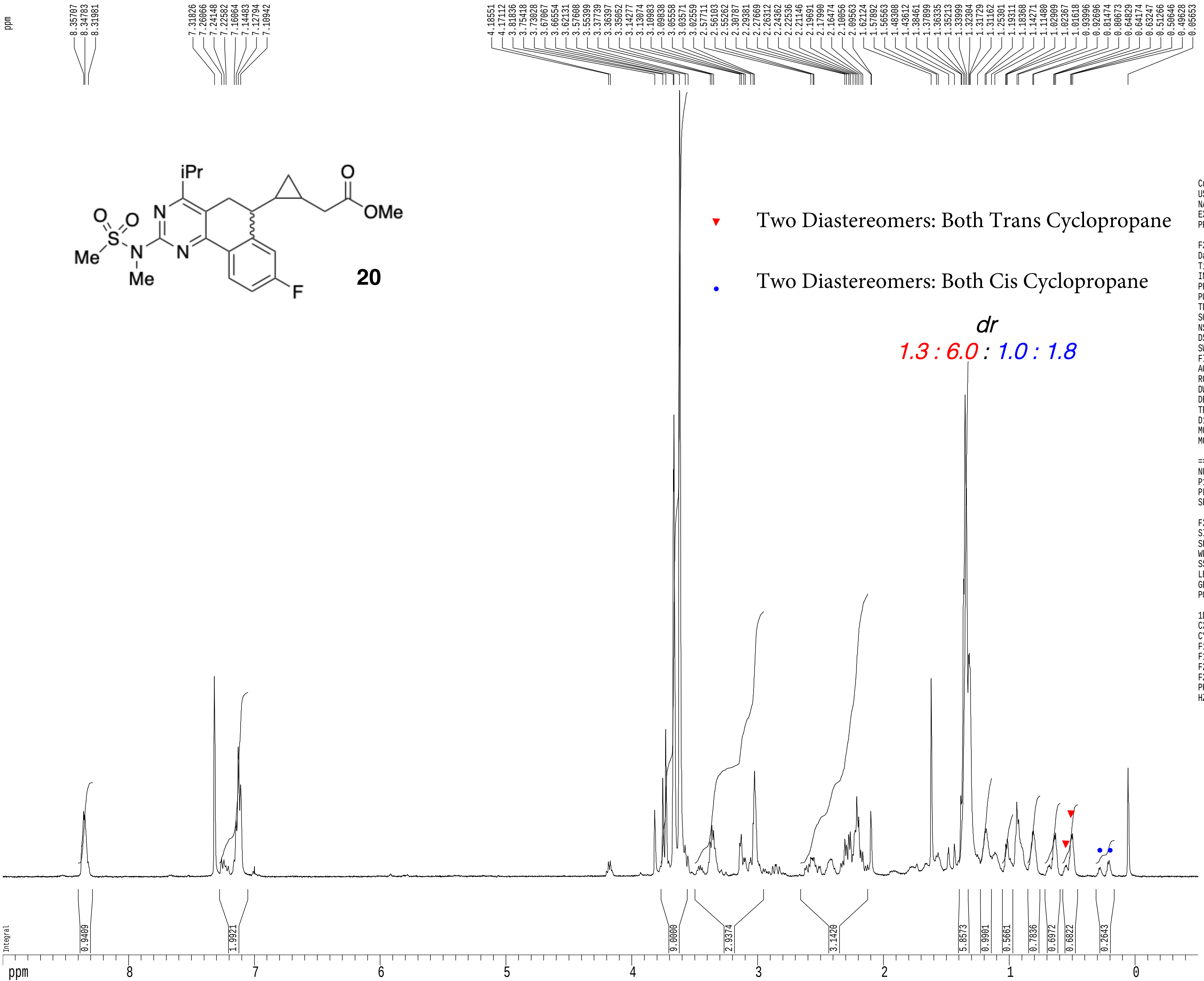


```
Current Data Parameters
USER              mcginnit
NAME              tmm-4-046
EXPNO             11
PROCNO            1

F2 - Acquisition Parameters
Date_             20211209
Time              16.09
INSTRUM           gn500
PROBHD   5 mm broadband
PULPROG           zg30
TD                81728
SOLVENT           CDCl3
NS                8
DS                2
SWH               8012.820 Hz
FIDRES            0.098043 Hz
AQ                5.0998774 sec
RG                912.3
DW                62.400 usec
DE                6.00 usec
TE                298.0 K
D1                0.10000000 sec
MCREST            0.00000000 sec
MCWRK             0.01500000 sec

======== CHANNEL f1 ========
NUC1              1H
P1                12.00 usec
PL1               -6.00 dB
SFO1              498.6534906 MHz

F2 - Processing parameters
SI                65536
SF                498.6500000 MHz
WDW               EM
SSB               0
LB                0.30 Hz
GB                0
PC                1.00

1D NMR plot parameters
CX                22.80 cm
CY                15.00 cm
F1P               9.000 ppm
F1                4487.85 Hz
F2P               -0.500 ppm
F2                -249.32 Hz
PPMCM             0.41667 ppm/cm
HZCM              207.77084 Hz/cm
```

Z-restored spin-echo 13C spectrum with 1H decoupling

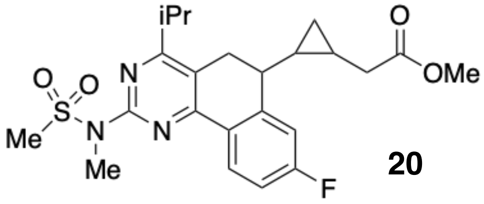

20

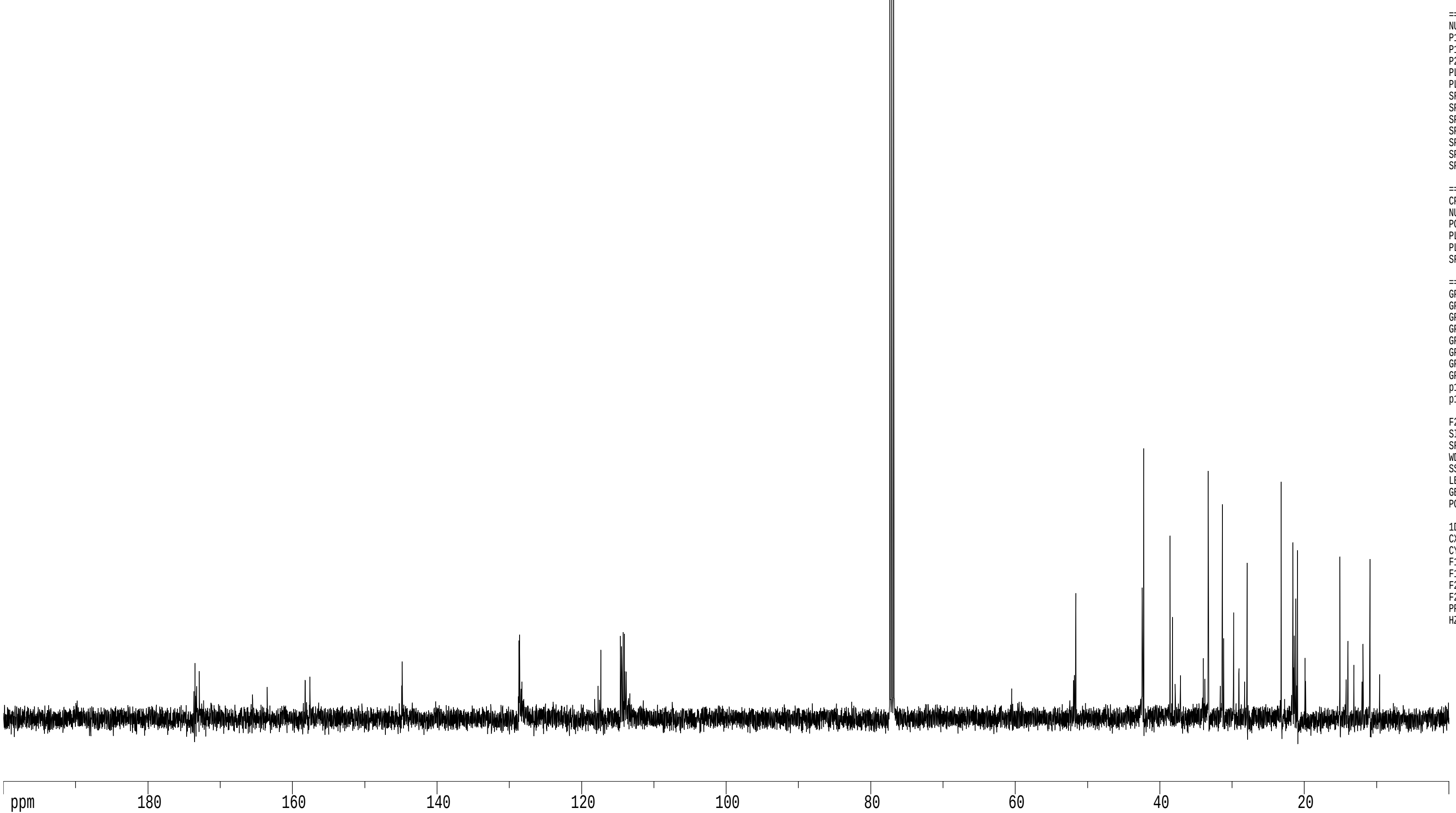


```
Current Data Parameters
USER            mcginnit
NAME       tmm-4-046-char
EXPNO                  2
PROCNO                 1

F2 - Acquisition Parameters
Date_           20220211
Time               13.13
INSTRUM          cryo500
PROBHD   5 mm CPTCI 1H-
PULPROG  SpinEchopg30gp2.prd
TD                 65536
SOLVENT            CDCl3
NS                   496
DS                    16
SWH            30303.031 Hz
FIDRES          0.462388 Hz
AQ             1.0813940 sec
RG                 16384
DW                16.500 usec
DE                  6.00 usec
TE                 298.0 K
D1            0.25000000 sec
d11           0.03000000 sec
D16           0.00020000 sec
d17           0.00019600 sec
MCREST        0.00000000 sec
MCWRK         0.01500000 sec
P2                 37.70 usec

======== CHANNEL f1 ========
NUC1                 13C
P1                 18.85 usec
P12              2000.00 usec
P20               500.00 usec
PL0               120.00 dB
PL1                -1.00 dB
SFO1         125.7942548 MHz
SP2                 1.55 dB
SP4                 1.55 dB
SPNAM2      Crp60comp.4
SPNAM4   Crp60,0.5,20.1
SPOFF2              0.00 Hz
SPOFF4              0.00 Hz

======== CHANNEL f2 ========
CPDPRG2          waltz16
NUC2                  1H
PCPD2             100.00 usec
PL2                 1.60 dB
PL12               22.00 dB
SFO2         500.2225011 MHz

====== GRADIENT CHANNEL =====
GPNAM1          SINE.100
GPNAM2          SINE.100
GPX1                0.00 %
GPX2                0.00 %
GPY1                0.00 %
GPY2                0.00 %
GPZ1               30.00 %
GPZ2               50.00 %
p15               500.00 usec
p16              1000.00 usec

F2 - Processing parameters
SI                 65536
SF           125.7804190 MHz
WDW                   EM
SSB                    0
LB                  1.00 Hz
GB                     0
PC                  2.00

1D NMR plot parameters
CX                 22.80 cm
CY                 15.65 cm
F1P              200.000 ppm
F1              25156.08 Hz
F2P                0.000 ppm
F2                  0.00 Hz
PPMCM            8.77193 ppm/cm
HZCM          1103.33704 Hz/cm
```

19F spectrum

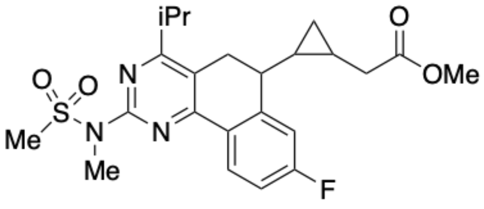

20

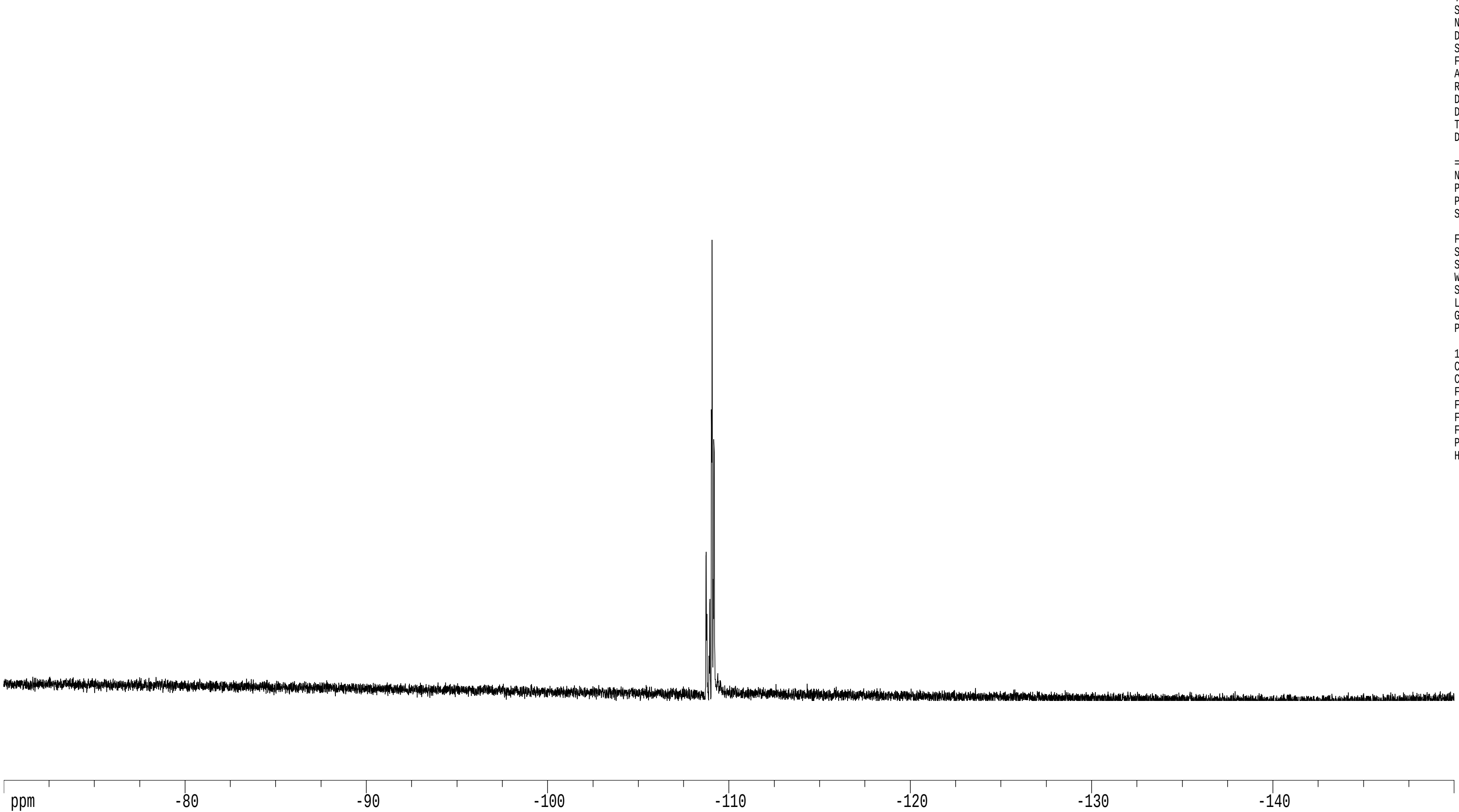


```
Current Data Parameters
USER            mcginnit
NAME     tmm-4-046-char
EXPNO                 3
PROCNO                1

F2 - Acquisition Parameters
Date_          20220210
Time              11.22
INSTRUM          drx400
PROBHD   5 mm QNP H/F/P
PULPROG        zgflqn30
TD                65536
SOLVENT           CDCl3
NS                   40
DS                    2
SWH           75187.969 Hz
FIDRES         1.147277 Hz
AQ            0.4358644 sec
RG                 1024
DW                6.650 usec
DE                 9.46 usec
TE                298.0 K
D1           2.00000000 sec

======== CHANNEL f1 ========
NUC1                19F
P1                21.75 usec
PL1               -6.00 dB
SFO1       376.4646491 MHz

F2 - Processing parameters
SI                65536
SF          376.4984640 MHz
WDW                  EM
SSB                   0
LB                 1.00 Hz
GB                    0
PC                 1.00

1D NMR plot parameters
CX                22.80 cm
CY                 7.00 cm
F1P             -70.000 ppm
F1            -26354.89 Hz
F2P            -150.000 ppm
F2            -56474.77 Hz
PPMCM           3.50877 ppm/cm
HZCM         1321.04736 Hz/cm
```

1H spectrum

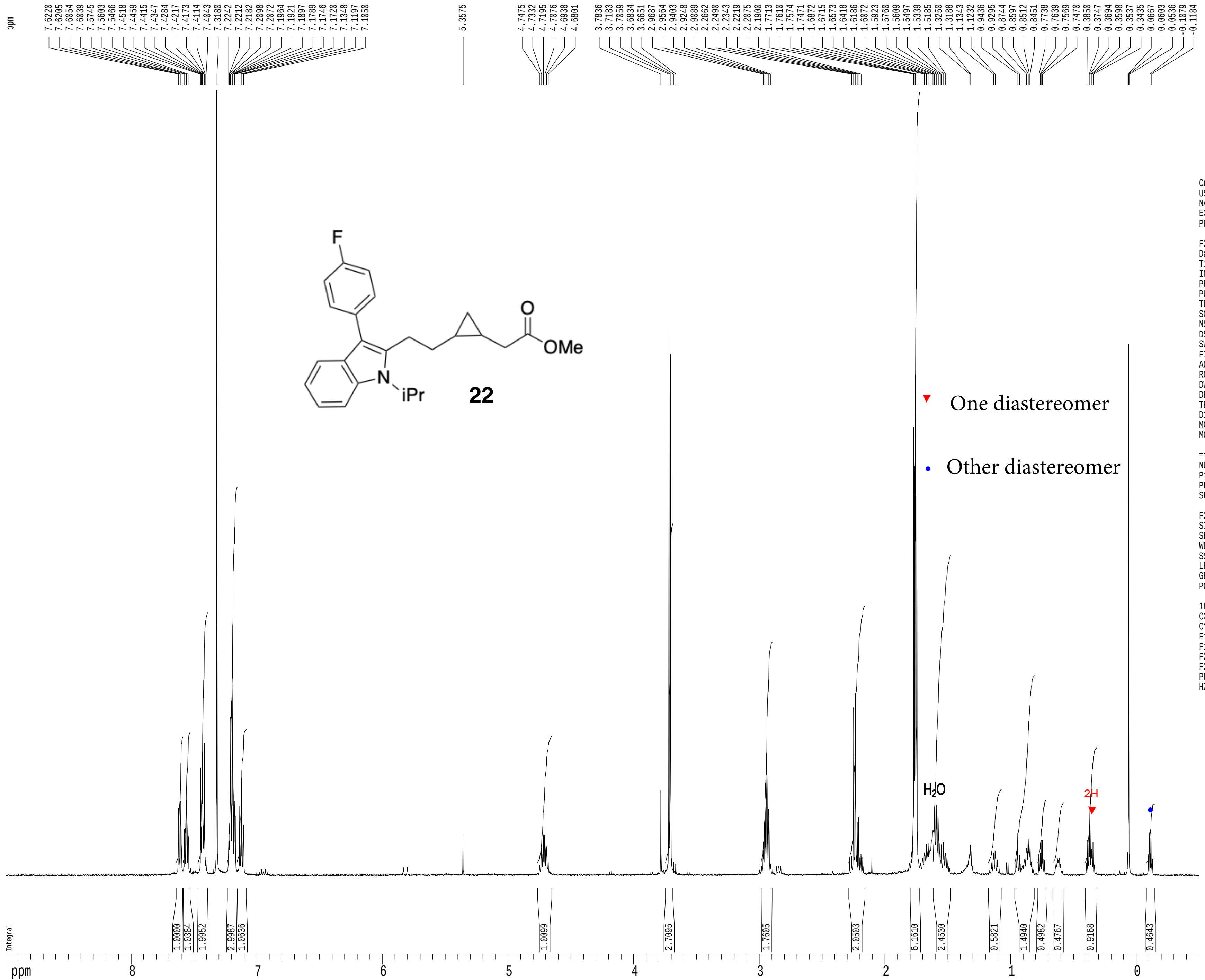


```
Current Data Parameters
USER            mcginnit
NAME      tmm-4-fluvastatin
EXPNO                  1
PROCNO                 1

F2 - Acquisition Parameters
Date_           20220707
Time               13.45
INSTRUM            gn500
PROBHD   5 mm broadband
PULPROG             zg30
TD                 81728
SOLVENT            CDCl3
NS                     8
DS                     2
SWH            8012.820 Hz
FIDRES         0.098043 Hz
AQ            5.0998774 sec
RG                1625.5
DW               62.400 usec
DE                 6.00 usec
TE                298.0 K
D1           0.10000000 sec
MCREST       0.00000000 sec
MCWRK        0.01500000 sec

======== CHANNEL f1 ========
NUC1                  1H
P1                 12.00 usec
PL1                -6.00 dB
SFO1         498.6534906 MHz

F2 - Processing parameters
SI                 65536
SF           498.6500000 MHz
WDW                   EM
SSB                    0
LB                  0.30 Hz
GB                     0
PC                  1.00

1D NMR plot parameters
CX                 22.80 cm
CY                 15.00 cm
F1P                9.000 ppm
F1               4487.85 Hz
F2P               -0.500 ppm
F2               -249.32 Hz
PPMCM            0.41667 ppm/cm
HZCM            207.77084 Hz/cm
```

13C spectrum

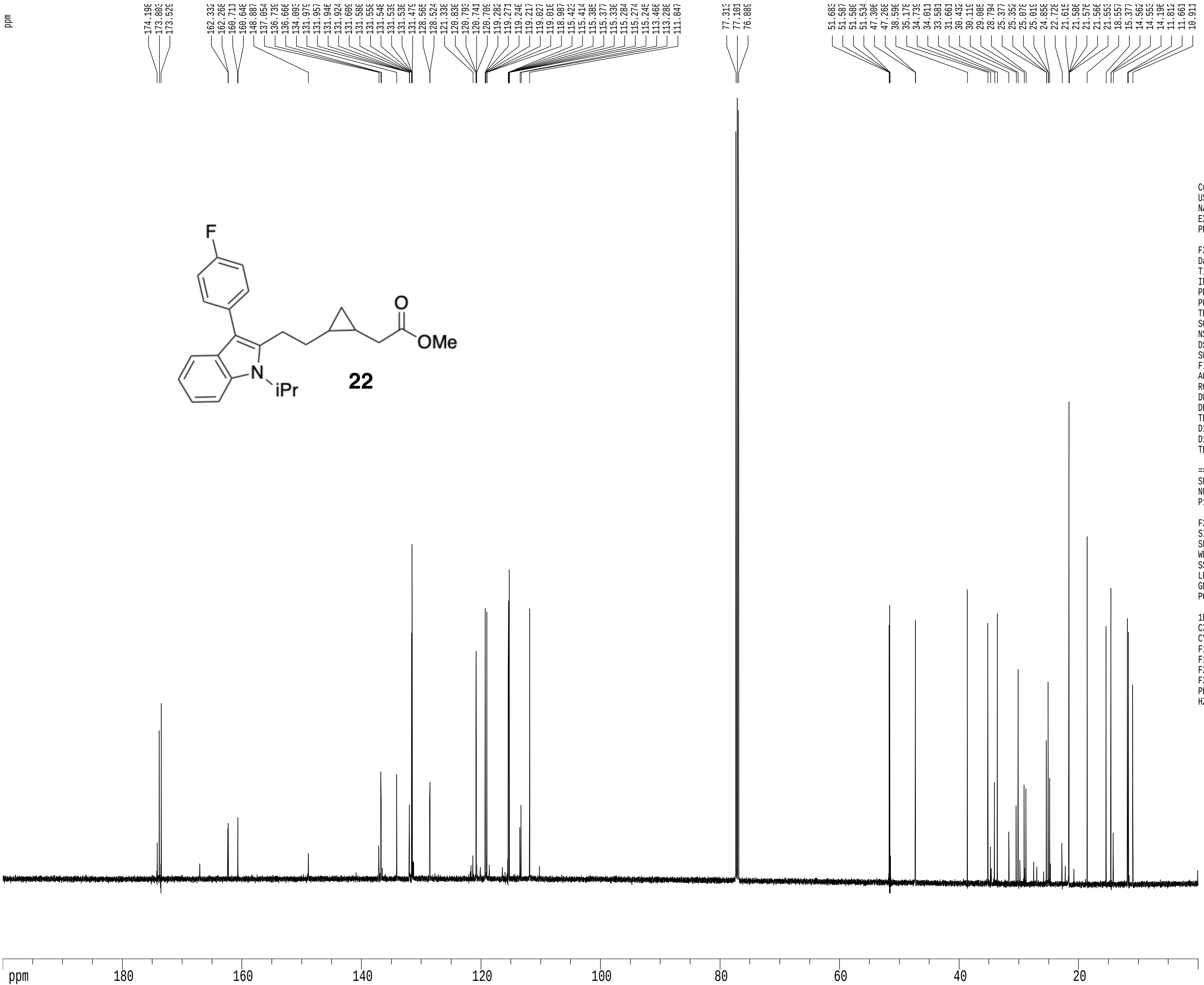

Current Data Parameters
USER mcginnit
NAME tmm-4-037
EXPNO 12
PROCNO 1

F2 - Acquisition Parameters
Date_ 20211203
Time 17.06
INSTRUM av600
PROBHD 5 mm CPBBO BB-
PULPROG zgdc30
TD 65536
SOLVENT CDCl3
NS 268
DS 4
SWH 36231.883 Hz
FIDRES 0.552855 Hz
AQ 0.9044468 sec
RG 2050
DW 13.800 usec
DE 19.63 usec
TE 298.0 K
D1 0.40000001 sec
D11 0.03000000 sec
TD0 1

======== CHANNEL f1 ========
SFO1 150.9194080 MHz
NUC1 13C
P1 10.10 usec

F2 - Processing parameters
SI 65536
SF 150.9028085 MHz
WDW no
SSB 0
LB 0.00 Hz
GB 0
PC 1.00

1D NMR plot parameters
CX 22.80 cm
CY 15.00 cm
F1P 200.000 ppm
F1 30180.56 Hz
F2P 0.000 ppm
F2 0.00 Hz
PPMCM 8.77193 ppm/cm
HZCM 1323.70886 Hz/cm

19F spectrum

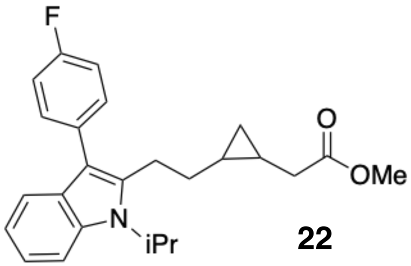

22

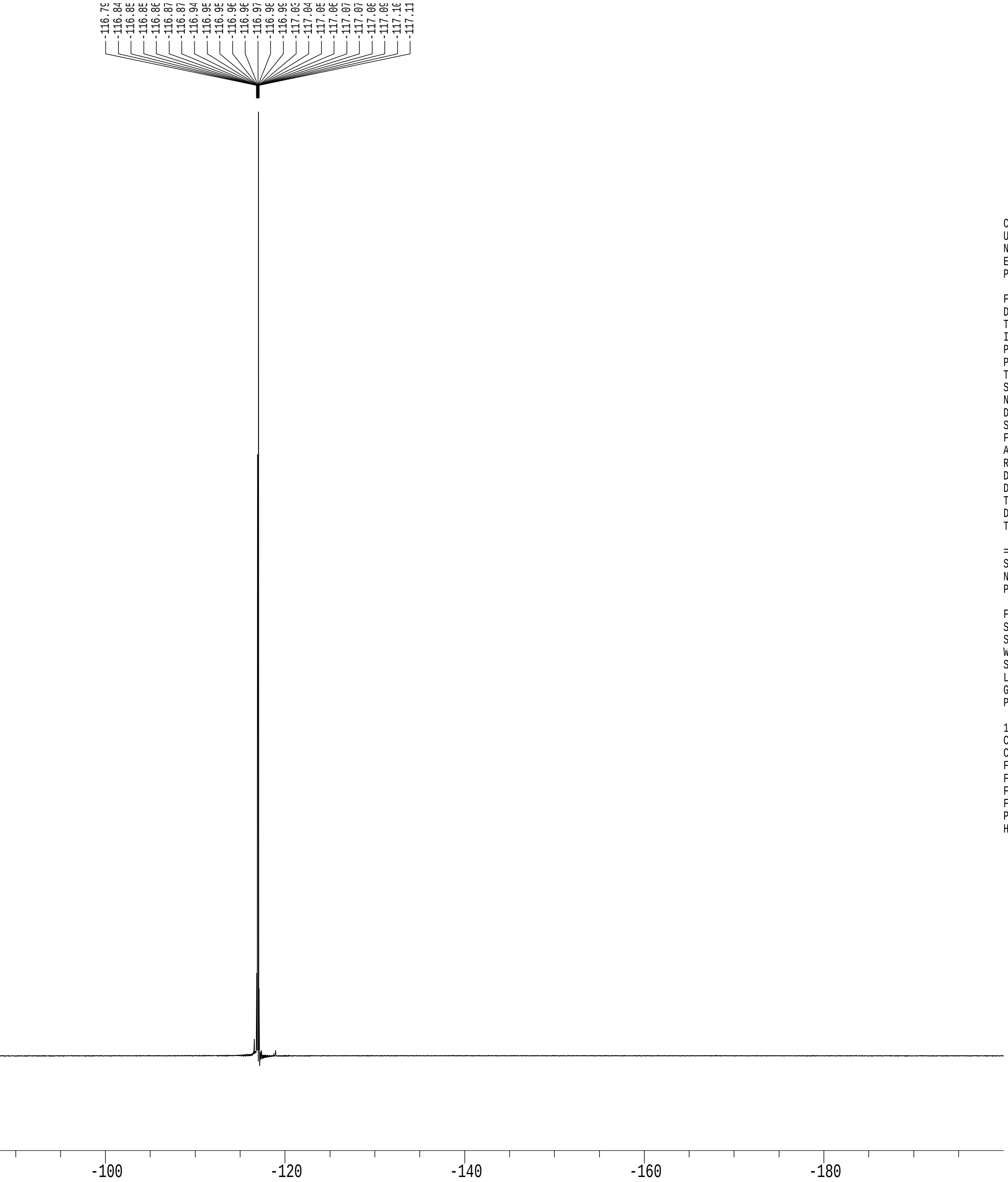


```
Current Data Parameters
USER           mcginnit
NAME          tmm-4-037
EXPNO                13
PROCNO                1

F2 - Acquisition Parameters
Date_          20211203
Time              17.15
INSTRUM           av600
PROBHD   5 mm CPBBO BB-
PULPROG        zgflqn30
TD               131072
SOLVENT           CDCl3
NS                   16
DS                    2
SWH          178571.422 Hz
FIDRES         1.362392 Hz
AQ            0.3670516 sec
RG                  575
DW                2.800 usec
DE                18.00 usec
TE                297.9 K
D1           3.00000000 sec
TD0                   1

======== CHANNEL f1 ========
SFO1       564.6299196 MHz
NUC1                19F
P1                18.25 usec

F2 - Processing parameters
SI               131072
SF         564.6863858 MHz
WDW                  no
SSB                   0
LB                 0.00 Hz
GB                    0
PC                 1.00

1D NMR plot parameters
CX                22.80 cm
CY                15.00 cm
F1P             -40.000 ppm
F1            -22587.46 Hz
F2P            -200.000 ppm
F2           -112937.28 Hz
PPMCM           7.01754 ppm/cm
HZCM         3962.71167 Hz/cm
```

1H spectrum

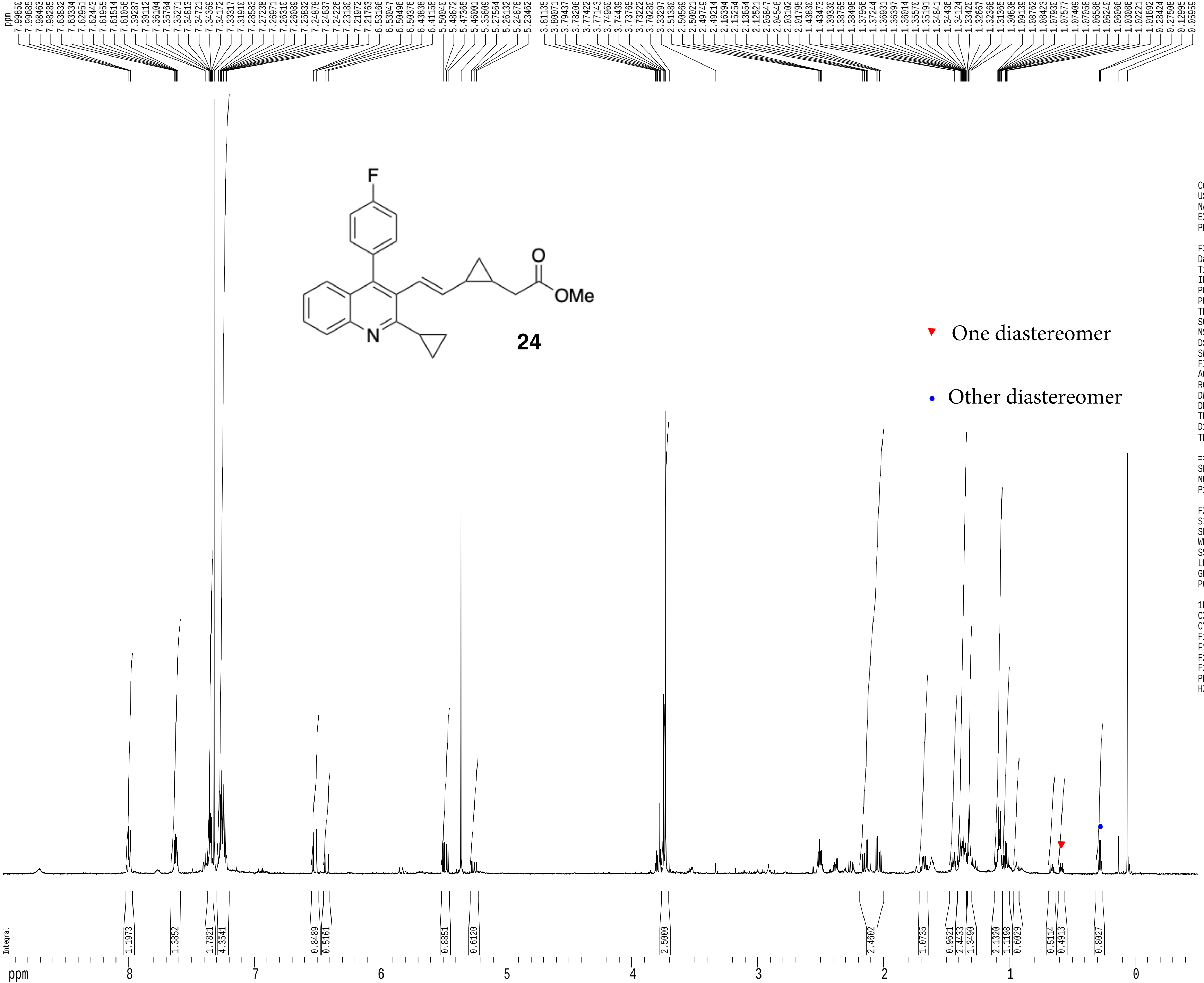


```
Current Data Parameters
USER            mcginnit
NAME      tmm-4-097-char
EXPNO                  1
PROCNO                 1

F2 - Acquisition Parameters
Date_           20220225
Time               13.26
INSTRUM            av600
PROBHD   5 mm CPBBO BB-
PULPROG             zg30
TD                 98074
SOLVENT            CDCl3
NS                     8
DS                     2
SWH             9615.385 Hz
FIDRES          0.098042 Hz
AQ             5.0998979 sec
RG                    10
DW                52.000 usec
DE                 14.23 usec
TE                 297.9 K
D1            0.10000000 sec
TD0                    1

======== CHANNEL f1 ========
SFO1        600.1342009 MHz
NUC1                 1H
P1                  9.50 usec

F2 - Processing parameters
SI                 65536
SF          600.1300000 MHz
WDW                   no
SSB                    0
LB                  0.00 Hz
GB                     0
PC                  1.00

1D NMR plot parameters
CX                 22.80 cm
CY                 15.00 cm
F1P                9.000 ppm
F1               5401.17 Hz
F2P               -0.500 ppm
F2               -300.06 Hz
PPMCM            0.41667 ppm/cm
HZCM            250.05418 Hz/cm
```

13C spectrum

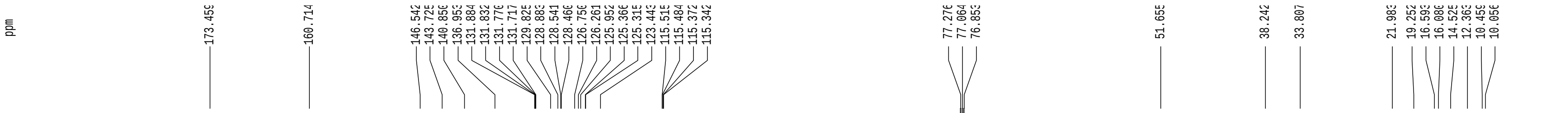

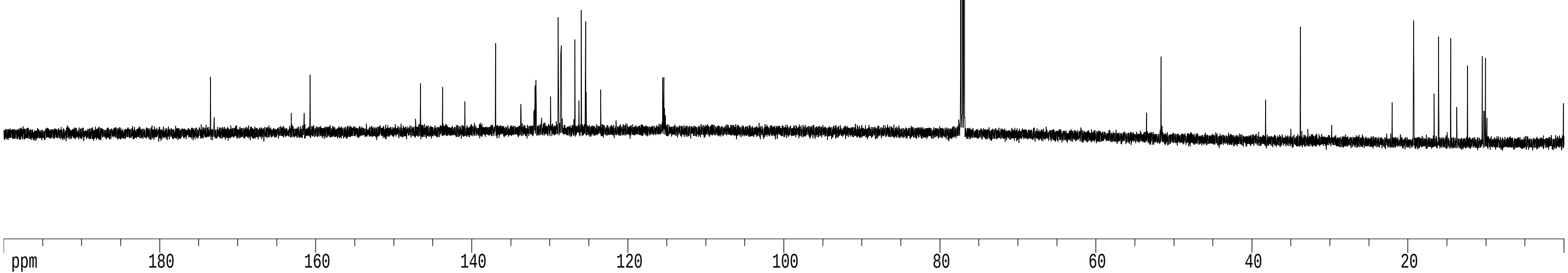

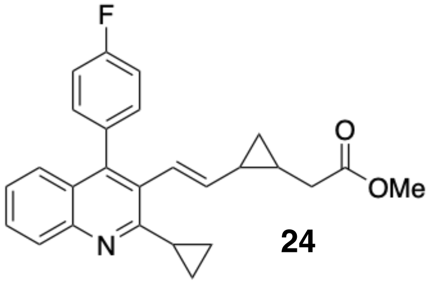

24

Current Data Parameters
USER mcginnit
NAME tmm-4-097-char
EXPNO 2
PROCNO 1

F2 - Acquisition Parameters
Date_ 20220225
Time 13.33
INSTRUM av600
PROBHD 5 mm CPBBO BB-
PULPROG zgdc30
TD 65536
SOLVENT CDCl3
NS 518
DS 4
SWH 36231.883 Hz
FIDRES 0.552855 Hz
AQ 0.9044468 sec
RG 2050
DW 13.800 usec
DE 19.63 usec
TE 298.0 K
D1 0.40000001 sec
D11 0.03000000 sec
TD0 1

======== CHANNEL f1 ========
SFO1 150.9194080 MHz
NUC1 13C
P1 10.10 usec

F2 - Processing parameters
SI 65536
SF 150.9028085 MHz
WDW no
SSB 0
LB 0.00 Hz
GB 0
PC 1.00

1D NMR plot parameters
CX 22.80 cm
CY 25.00 cm
F1P 200.000 ppm
F1 30180.56 Hz
F2P 0.000 ppm
F2 0.00 Hz
PPMCM 8.77193 ppm/cm
HZCM 1323.70886 Hz/cm

19F spectrum

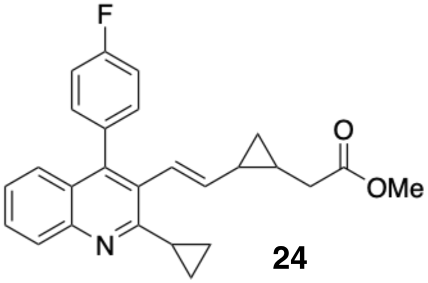

24

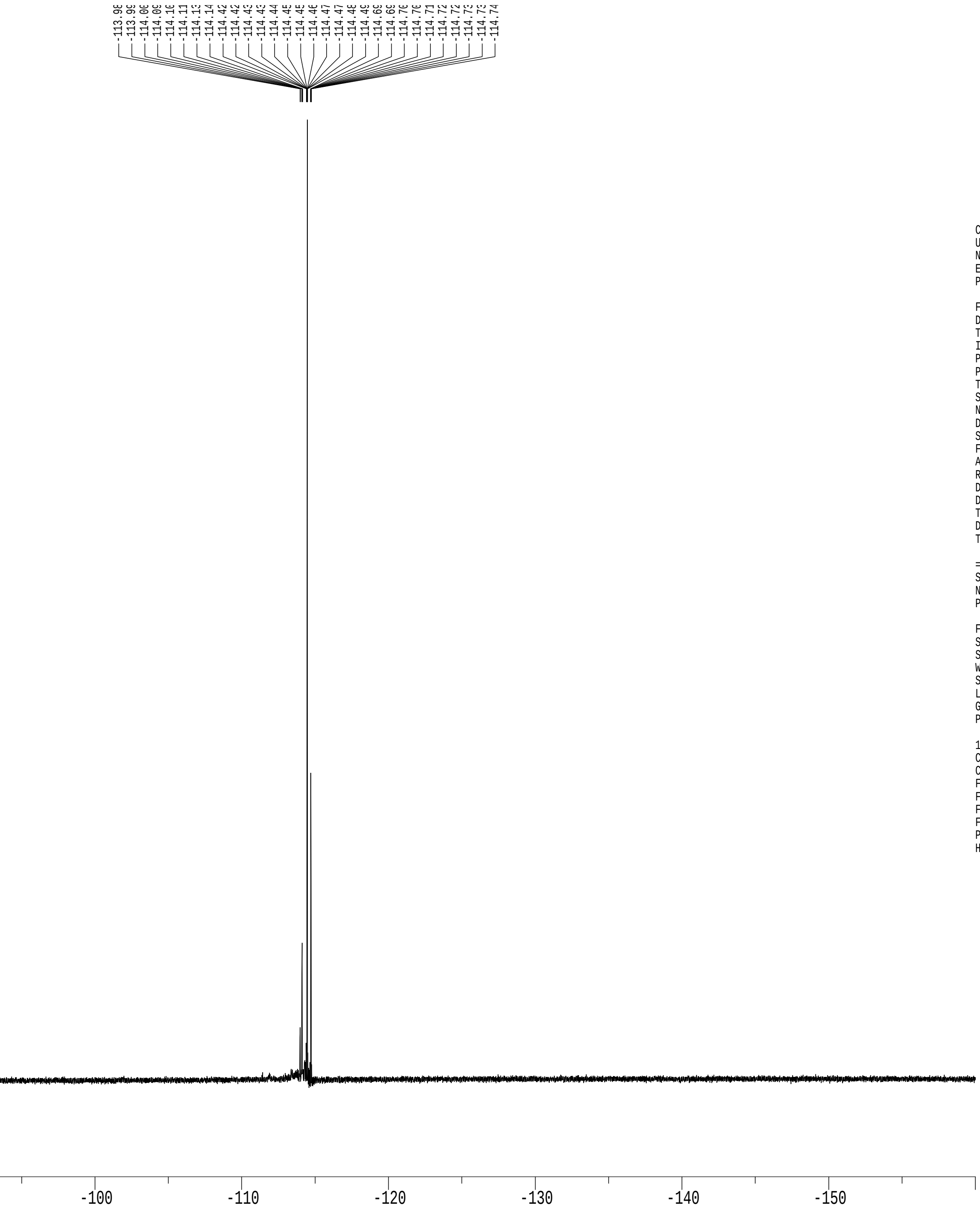


```
Current Data Parameters
USER            mcginnit
NAME      tmm-4-097-char
EXPNO                  3
PROCNO                 1

F2 - Acquisition Parameters
Date_           20220225
Time               13.47
INSTRUM            av600
PROBHD   5 mm CPBBO BB-
PULPROG        zgflqn30
TD                131072
SOLVENT            CDCl3
NS                    16
DS                     2
SWH          178571.422 Hz
FIDRES         1.362392 Hz
AQ            0.3670516 sec
RG                   575
DW                 2.800 usec
DE                 18.00 usec
TE                 298.0 K
D1            3.00000000 sec
TD0                    1

======== CHANNEL f1 ========
SFO1        564.6299196 MHz
NUC1                 19F
P1                 18.25 usec

F2 - Processing parameters
SI                131072
SF          564.6863858 MHz
WDW                   no
SSB                    0
LB                  0.00 Hz
GB                     0
PC                  1.00

1D NMR plot parameters
CX                 22.80 cm
CY                 15.00 cm
F1P              -60.000 ppm
F1              -33881.19 Hz
F2P             -160.000 ppm
F2              -90349.83 Hz
PPMCM            4.38596 ppm/cm
HZCM          2476.69482 Hz/cm
```

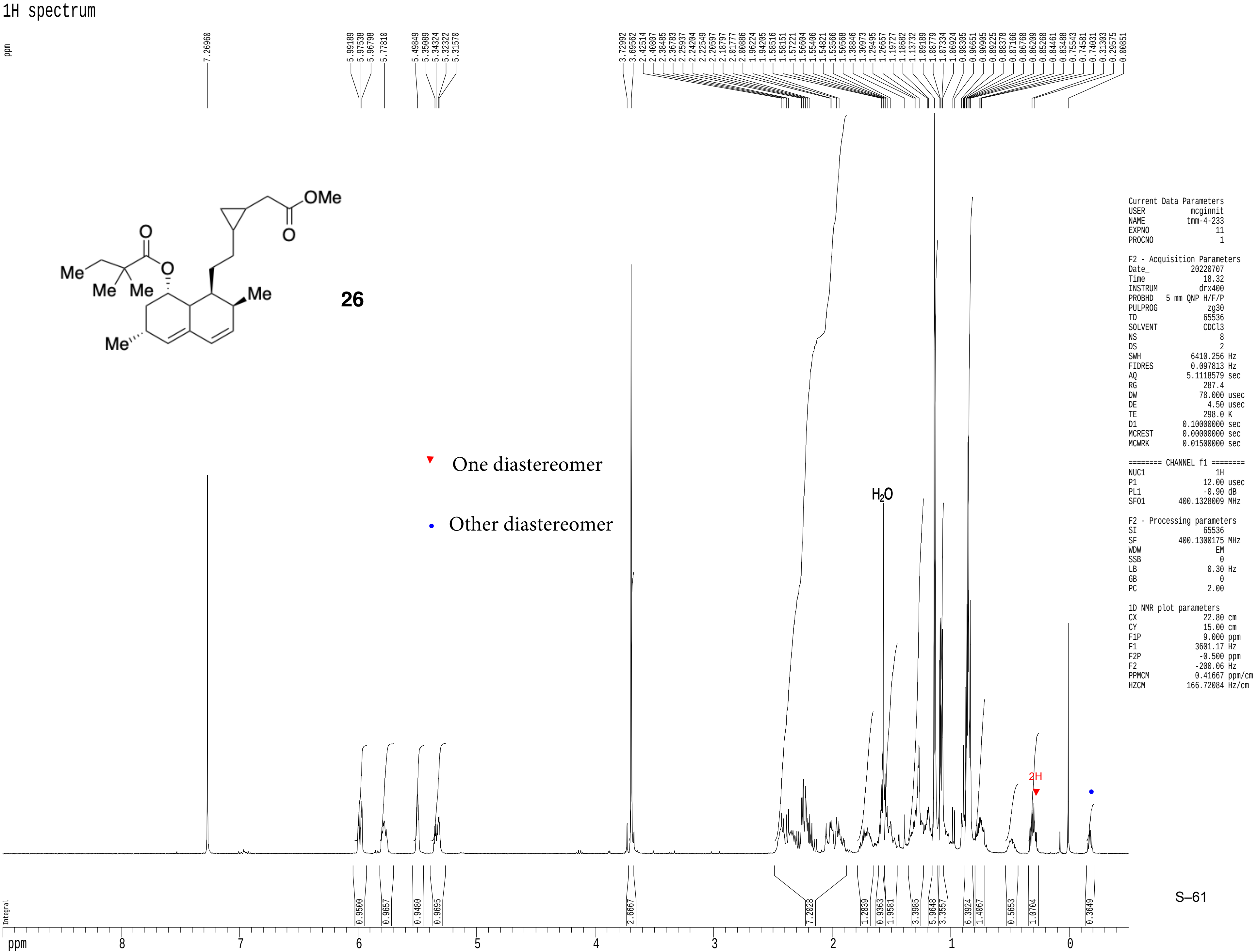
1H spectrum
26
One diastereomer
Other diastereomer
H2O
2H
Current Data Parameters
USER mcginnit
NAME tmm-4-233
EXPNO 11
PROCNO 1
F2 - Acquisition Parameters
Date_ 20220707
Time 18.32
INSTRUM drx400
PROBHD 5 mm QNP H/F/P
PULPROG zg30
TD 65536
SOLVENT CDCl3
NS 8
DS 2
SWH 6410.256 Hz
FIDRES 0.097813 Hz
AQ 5.1118579 sec
RG 287.4
DW 78.000 usec
DE 4.50 usec
TE 298.0 K
D1 0.10000000 sec
MCREST 0.00000000 sec
MCWRK 0.01500000 sec
======== CHANNEL f1 ========
NUC1 1H
P1 12.00 usec
PL1 -0.90 dB
SFO1 400.1328009 MHz
F2 - Processing parameters
SI 65536
SF 400.1300175 MHz
WDW EM
SSB 0
LB 0.30 Hz
GB 0
PC 2.00
1D NMR plot parameters
CX 22.80 cm
CY 15.00 cm
F1P 9.000 ppm
F1 3601.17 Hz
F2P -0.500 ppm
F2 -200.06 Hz
PPMCM 0.41667 ppm/cm
HZCM 166.72084 Hz/cm
ppm

13C Spectrum

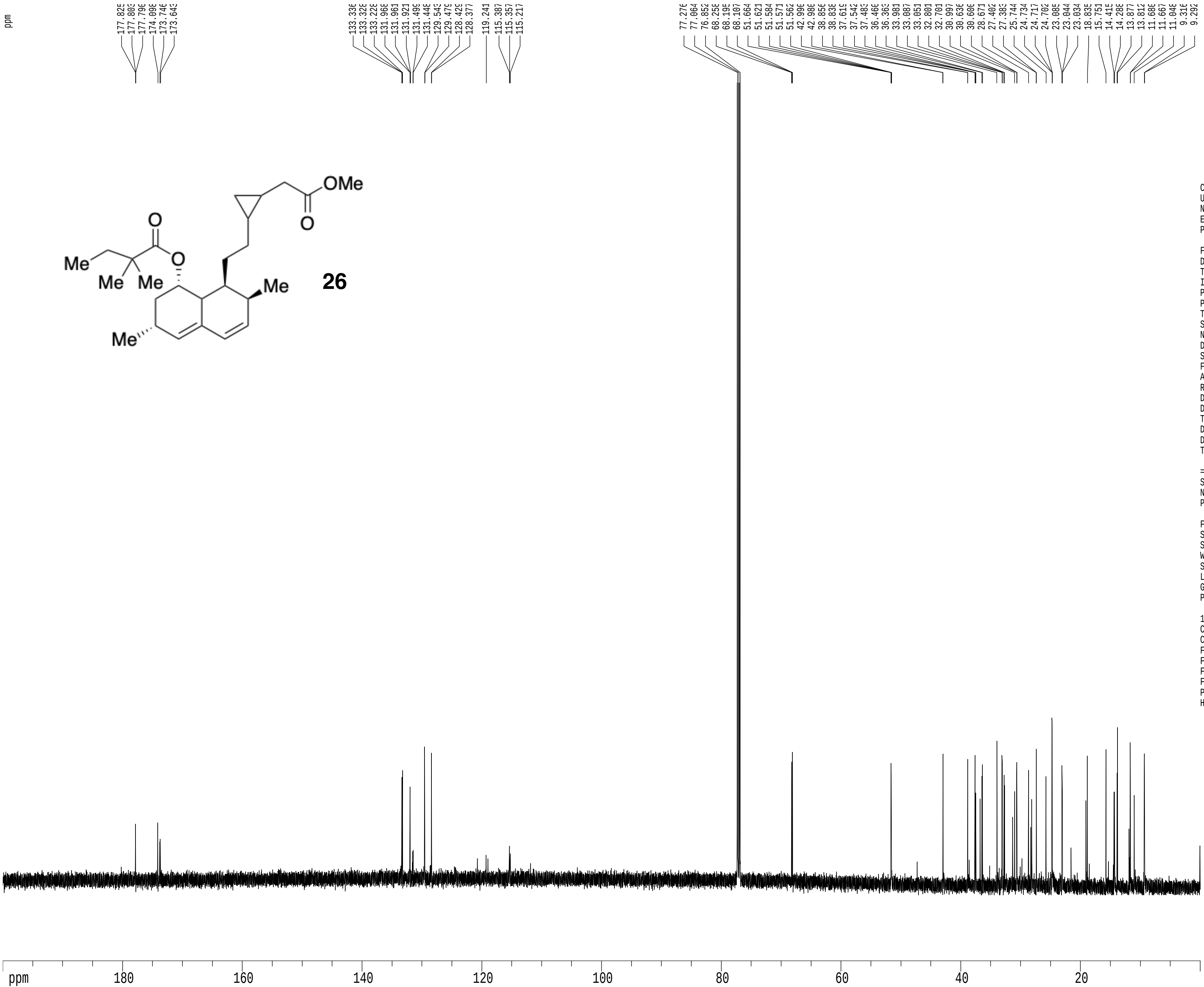

26

Current Data Parameters
USER mcginnit
NAME tmm-4-021-char
EXPNO 2
PROCNO 1

F2 - Acquisition Parameters
Date_ 20220224
Time 15.29
INSTRUM av600
PROBHD 5 mm CPBBO BB-
PULPROG zgdc30
TD 65536
SOLVENT CDCl3
NS 182
DS 4
SWH 36231.883 Hz
FIDRES 0.552855 Hz
AQ 0.9044468 sec
RG 2050
DW 13.800 usec
DE 19.63 usec
TE 297.9 K
D1 0.40000001 sec
D11 0.03000000 sec
TD0 1

======== CHANNEL f1 ========
SFO1 150.9194080 MHz
NUC1 13C
P1 10.10 usec

F2 - Processing parameters
SI 65536
SF 150.9028085 MHz
WDW no
SSB 0
LB 0.00 Hz
GB 0
PC 1.00

1D NMR plot parameters
CX 22.80 cm
CY 40.00 cm
F1P 200.000 ppm
F1 30180.56 Hz
F2P 0.000 ppm
F2 0.00 Hz
PPMCM 8.77193 ppm/cm
HZCM 1323.70886 Hz/cm

1H spectrum

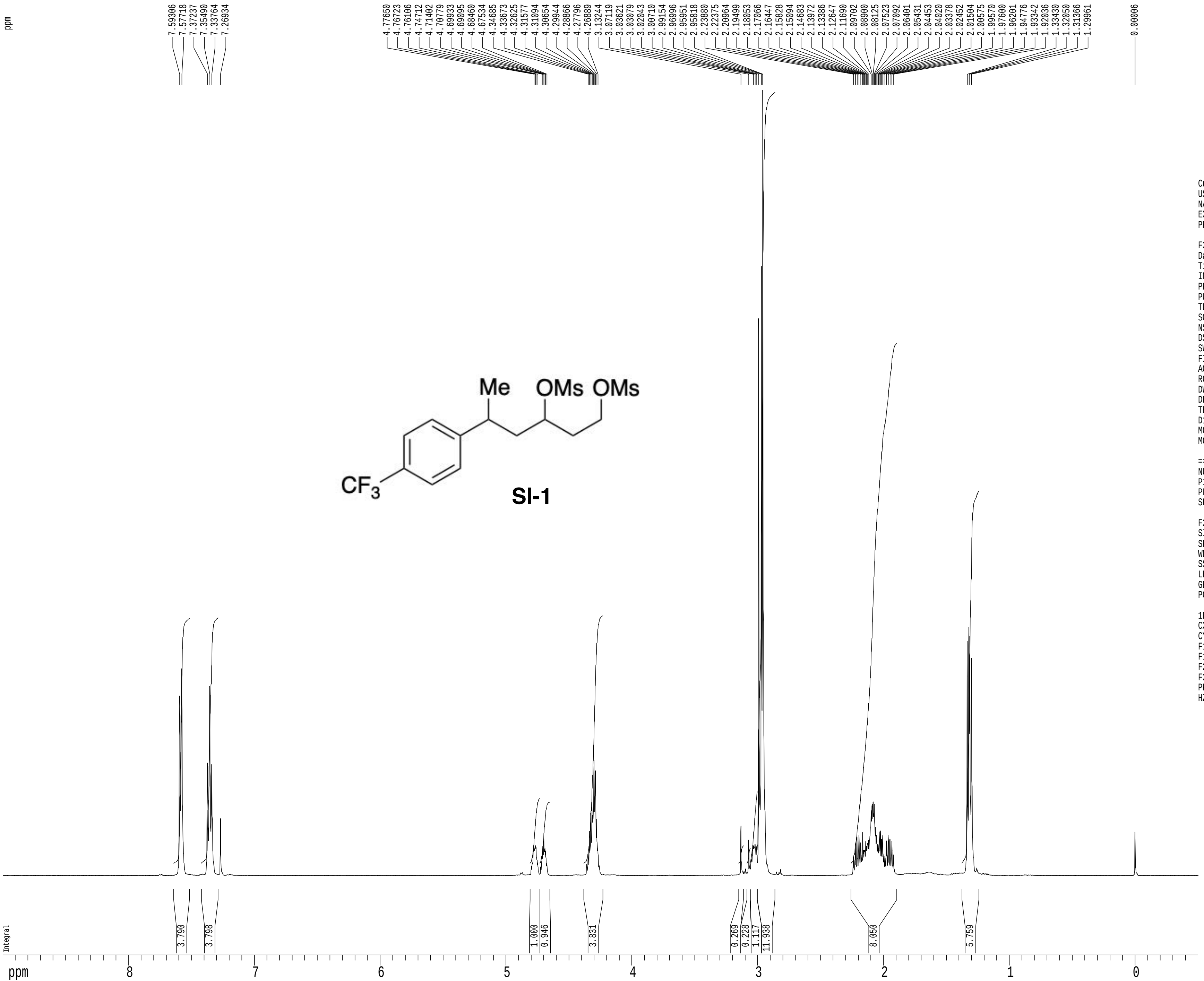

Current Data Parameters
USER tthane
NAME TATi215c
EXPNO 3
PROCNO 1

F2 - Acquisition Parameters
Date_ 20181016
Time 9.43
INSTRUM cryo500
PROBHD 5 mm CPTCI 1H-
PULPROG zg30
TD 81728
SOLVENT CDCl3T
NS 8
DS 2
SWH 8012.820 Hz
FIDRES 0.098043 Hz
AQ 5.0998774 sec
RG 5
DW 62.400 usec
DE 6.00 usec
TE 298.0 K
D1 0.10000000 sec
MCREST 0.00000000 sec
MCWRK 0.01500000 sec

======== CHANNEL f1 ========
NUC1 1H
P1 7.50 usec
PL1 1.60 dB
SFO1 500.2235015 MHz

F2 - Processing parameters
SI 65536
SF 500.2200282 MHz
WDW no
SSB 0
LB 0.00 Hz
GB 0
PC 1.00

1D NMR plot parameters
CX 22.80 cm
CY 15.00 cm
F1P 9.000 ppm
F1 4501.98 Hz
F2P -0.500 ppm
F2 -250.11 Hz
PPMCM 0.41667 ppm/cm
HZCM 208.42502 Hz/cm

19F spectrum

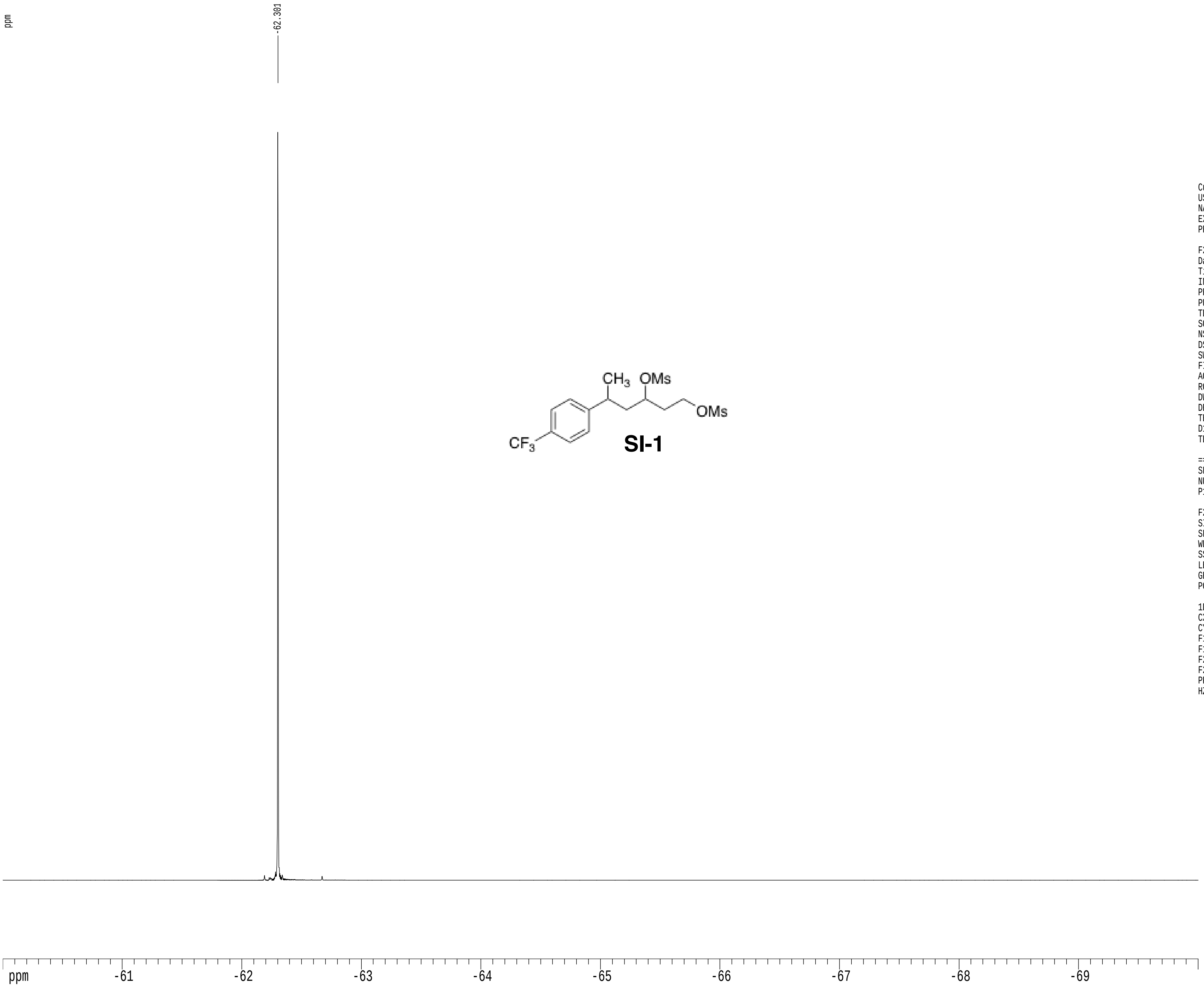

SI-1

```
Current Data Parameters
USER              tthane
NAME     TATi215fluorine
EXPNO                  1
PROCNO                 1

F2 - Acquisition Parameters
Date_          20211217
Time              15.06
INSTRUM           av600
PROBHD   5 mm CPBBO BB-
PULPROG        zgflqn30
TD               131072
SOLVENT          CDCl3T
NS                   16
DS                    2
SWH          178571.422 Hz
FIDRES         1.362392 Hz
AQ            0.3670516 sec
RG                  575
DW                2.800 usec
DE                18.00 usec
TE                298.0 K
D1           3.00000000 sec
TD0                   1

======== CHANNEL f1 ========
SFO1       564.6299196 MHz
NUC1                19F
P1                18.25 usec

F2 - Processing parameters
SI               131072
SF          564.6863858 MHz
WDW                  no
SSB                   0
LB                 0.00 Hz
GB                    0
PC                 1.00

1D NMR plot parameters
CX                22.80 cm
CY                15.00 cm
F1P             -60.000 ppm
F1            -33881.19 Hz
F2P             -70.000 ppm
F2            -39528.05 Hz
PPMCM           0.43860 ppm/cm
HZCM          247.66939 Hz/cm
```

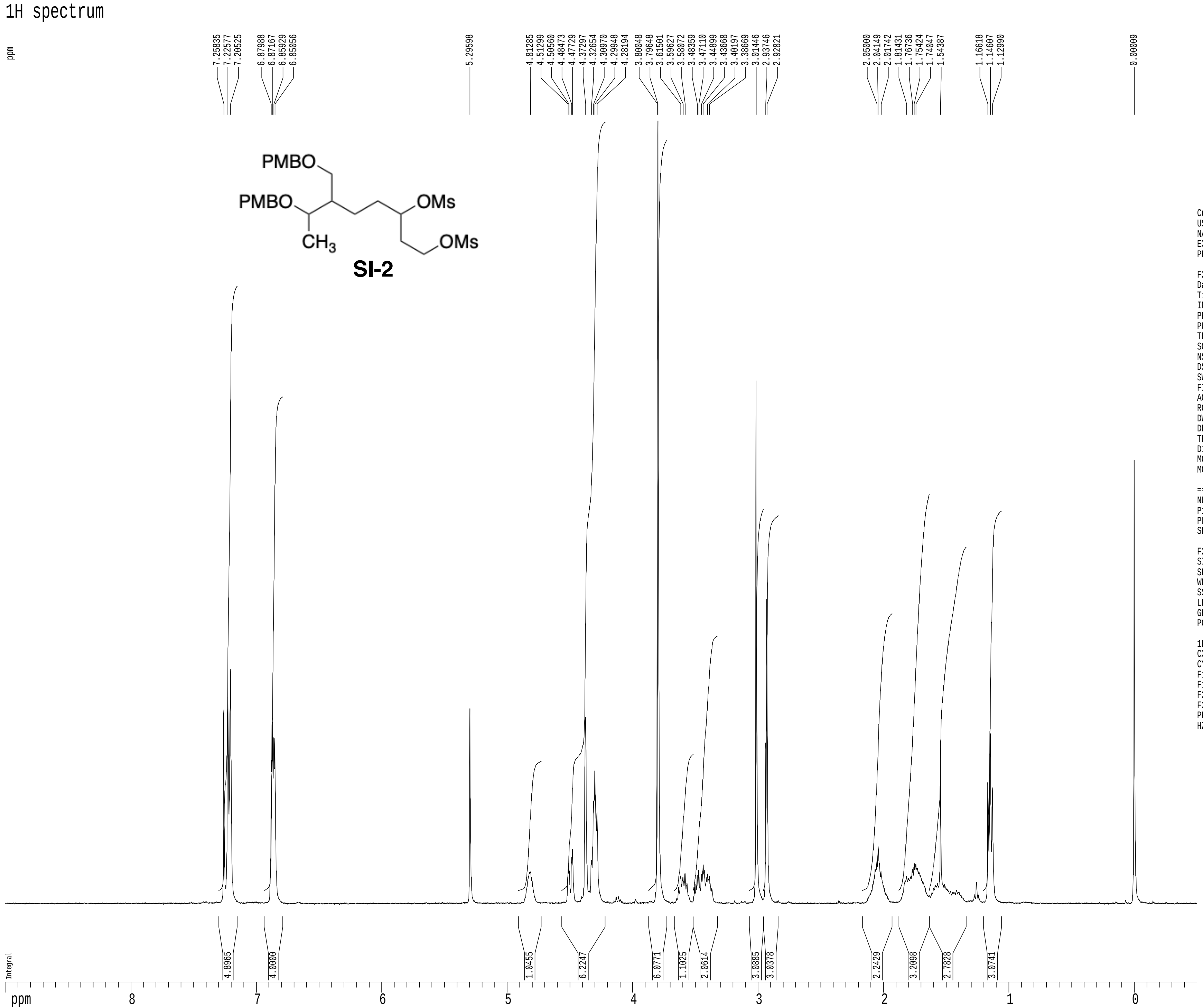


```
Current Data Parameters
USER          mcginnit
NAME          tmm-1-179
EXPNO                2
PROCNO               1

F2 - Acquisition Parameters
Date_         20190830
Time             15.51
INSTRUM         drx400
PROBHD   5 mm QNP H/F/P
PULPROG           zg30
TD               65536
SOLVENT          CDCl3
NS                   8
DS                   2
SWH           6410.256 Hz
FIDRES        0.097813 Hz
AQ           5.1118579 sec
RG               406.4
DW              78.000 usec
DE                4.50 usec
TE               298.1 K
D1          0.10000000 sec
MCREST      0.00000000 sec
MCWRK       0.01500000 sec

======== CHANNEL f1 ========
NUC1                1H
P1               12.00 usec
PL1              -1.10 dB
SFO1       400.1328009 MHz

F2 - Processing parameters
SI               65536
SF         400.1300220 MHz
WDW                 EM
SSB                  0
LB                0.30 Hz
GB                   0
PC                2.00

1D NMR plot parameters
CX               22.80 cm
CY               15.00 cm
F1P              9.000 ppm
F1             3601.17 Hz
F2P             -0.500 ppm
F2             -200.06 Hz
PPMCM          0.41667 ppm/cm
HZCM         166.72086 Hz/cm
```

1H spectrum

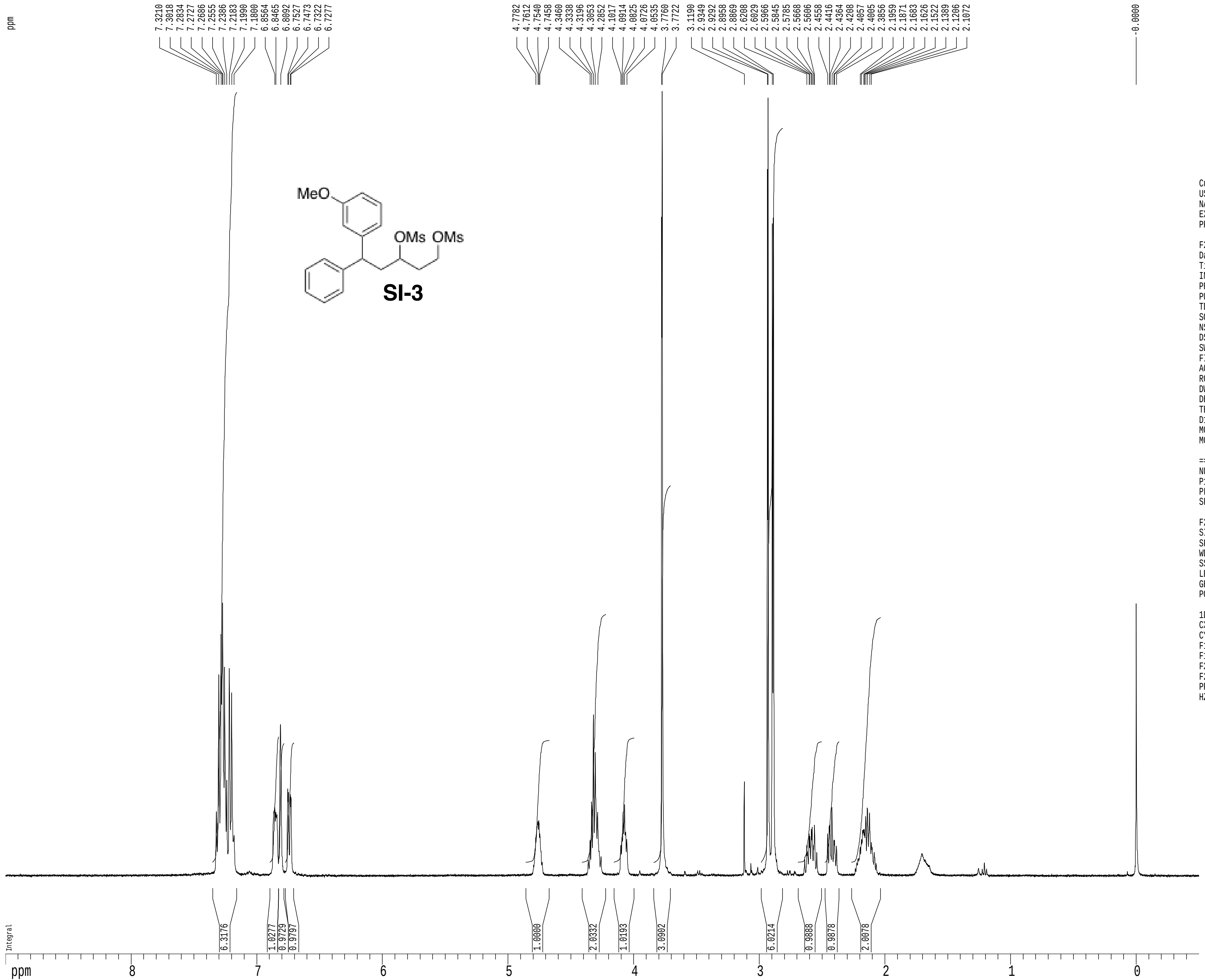

Current Data Parameters
USER tthane
NAME TATii269check
EXPNO 1
PROCNO 1

F2 - Acquisition Parameters
Date_ 20190722
Time 9.52
INSTRUM drx400
PROBHD 5 mm QNP H/F/P
PULPROG zg30
TD 65536
SOLVENT CDCl3T
NS 8
DS 2
SWH 6410.256 Hz
FIDRES 0.097813 Hz
AQ 5.1118579 sec
RG 287.4
DW 78.000 usec
DE 4.50 usec
TE 298.0 K
D1 0.10000000 sec
MCREST 0.00000000 sec
MCWRK 0.01500000 sec

======== CHANNEL f1 ========
NUC1 1H
P1 12.00 usec
PL1 -1.10 dB
SFO1 400.1328009 MHz

F2 - Processing parameters
SI 65536
SF 400.1300231 MHz
WDW no
SSB 0
LB 0.00 Hz
GB 0
PC 2.00

1D NMR plot parameters
CX 22.80 cm
CY 15.00 cm
F1P 9.000 ppm
F1 3601.17 Hz
F2P -0.500 ppm
F2 -200.06 Hz
PPMCM 0.41667 ppm/cm
HZCM 166.72086 Hz/cm

1H spectrum

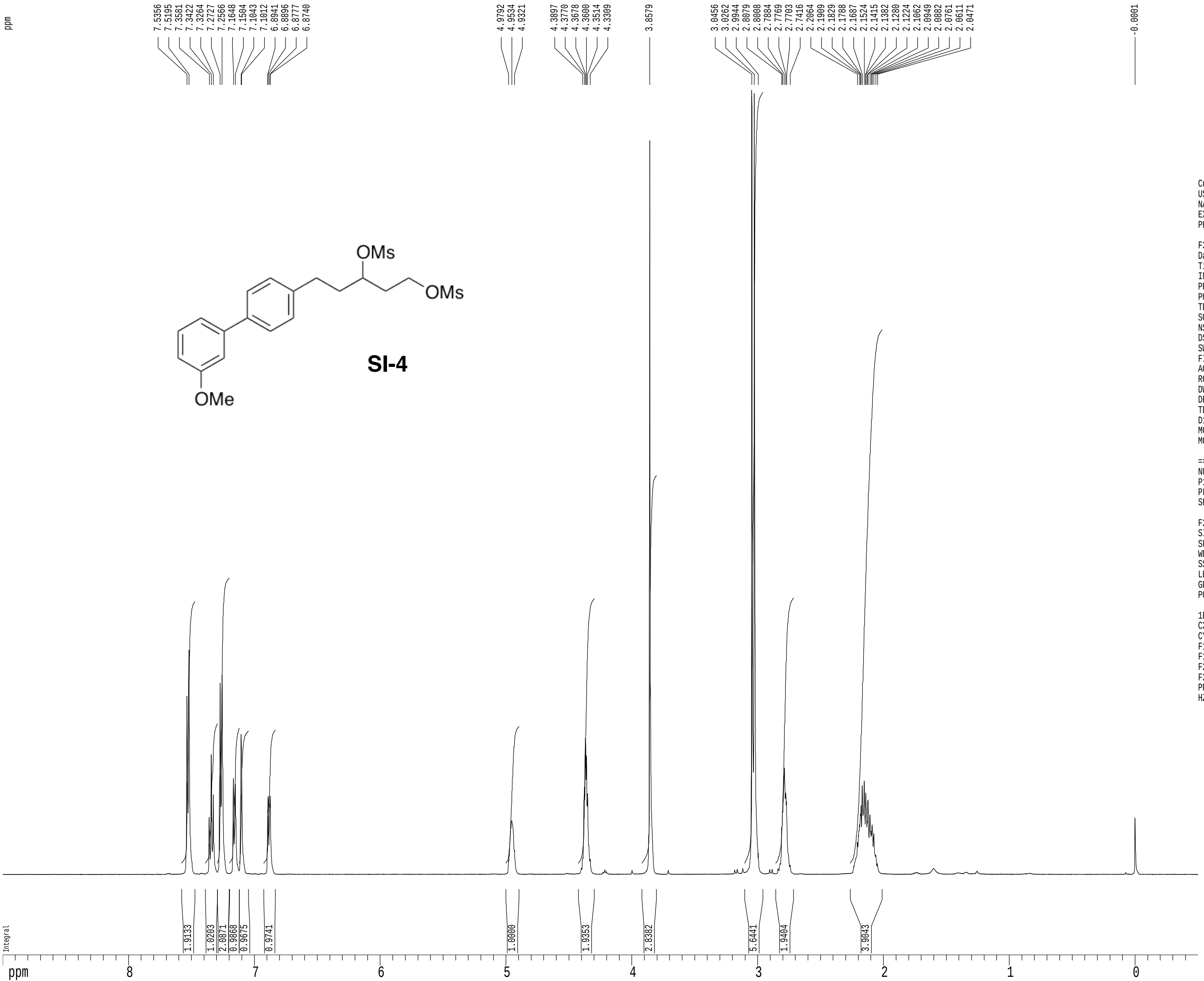


```
Current Data Parameters
USER          sanforda
NAME     ABS-2-025-proton
EXPNO                1
PROCNO               1

F2 - Acquisition Parameters
Date_          20181017
Time              15.15
INSTRUM        cryo500
PROBHD   5 mm CPTCI 1H-
PULPROG            zg30
TD                81728
SOLVENT           CDCl3
NS                    8
DS                    2
SWH           8012.820 Hz
FIDRES        0.098043 Hz
AQ           5.0998774 sec
RG                  6.3
DW               62.400 usec
DE                 6.00 usec
TE                298.0 K
D1           0.10000000 sec
MCREST       0.00000000 sec
MCWRK        0.01500000 sec

======== CHANNEL f1 ========
NUC1                 1H
P1                 7.50 usec
PL1                1.60 dB
SFO1        500.2235015 MHz

F2 - Processing parameters
SI                65536
SF          500.2200371 MHz
WDW                  no
SSB                   0
LB                 0.00 Hz
GB                    0
PC                 1.00

1D NMR plot parameters
CX                22.80 cm
CY                15.00 cm
F1P               9.000 ppm
F1              4501.98 Hz
F2P              -0.500 ppm
F2              -250.11 Hz
PPMCM           0.41667 ppm/cm
HZCM          208.42502 Hz/cm
```

1H spectrum

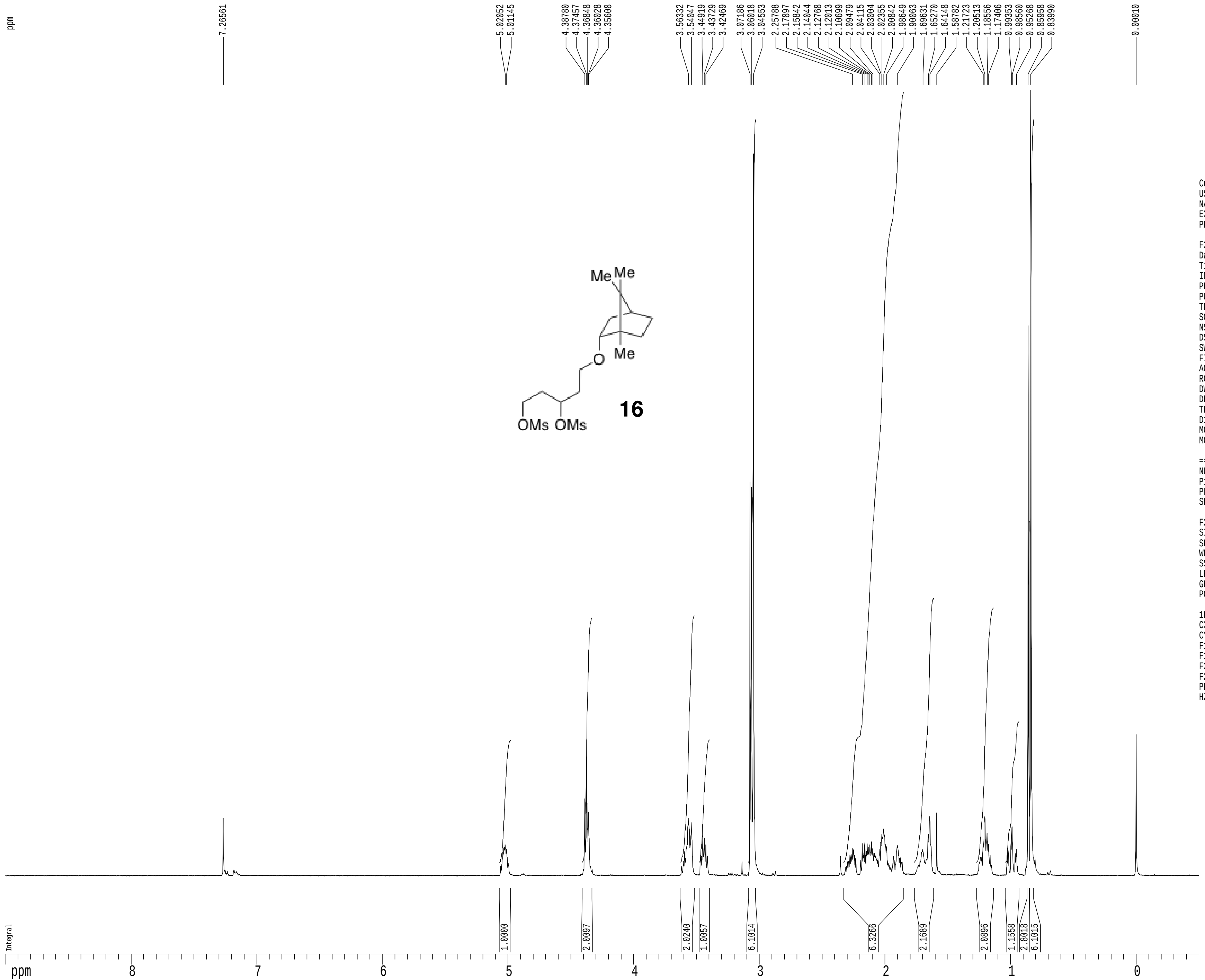


```
Current Data Parameters
USER              tthane
NAME      TATii304check
EXPNO                  1
PROCNO                 1

F2 - Acquisition Parameters
Date_           20190816
Time               16.52
INSTRUM           drx400
PROBHD   5 mm QNP H/F/P
PULPROG             zg30
TD                 65536
SOLVENT           CDCl3T
NS                     8
DS                     2
SWH             6410.256 Hz
FIDRES          0.097813 Hz
AQ             5.1118579 sec
RG                   181
DW                78.000 usec
DE                  4.50 usec
TE                 298.0 K
D1            0.10000000 sec
MCREST        0.00000000 sec
MCWRK         0.01500000 sec

======== CHANNEL f1 ========
NUC1                  1H
P1                 12.00 usec
PL1                -1.10 dB
SFO1         400.1328009 MHz

F2 - Processing parameters
SI                 65536
SF           400.1300190 MHz
WDW                   no
SSB                    0
LB                  0.00 Hz
GB                     0
PC                  2.00

1D NMR plot parameters
CX                 22.80 cm
CY                 15.00 cm
F1P                9.000 ppm
F1               3601.17 Hz
F2P               -0.500 ppm
F2               -200.06 Hz
PPMCM            0.41667 ppm/cm
HZCM           166.72084 Hz/cm
```

1H spectrum

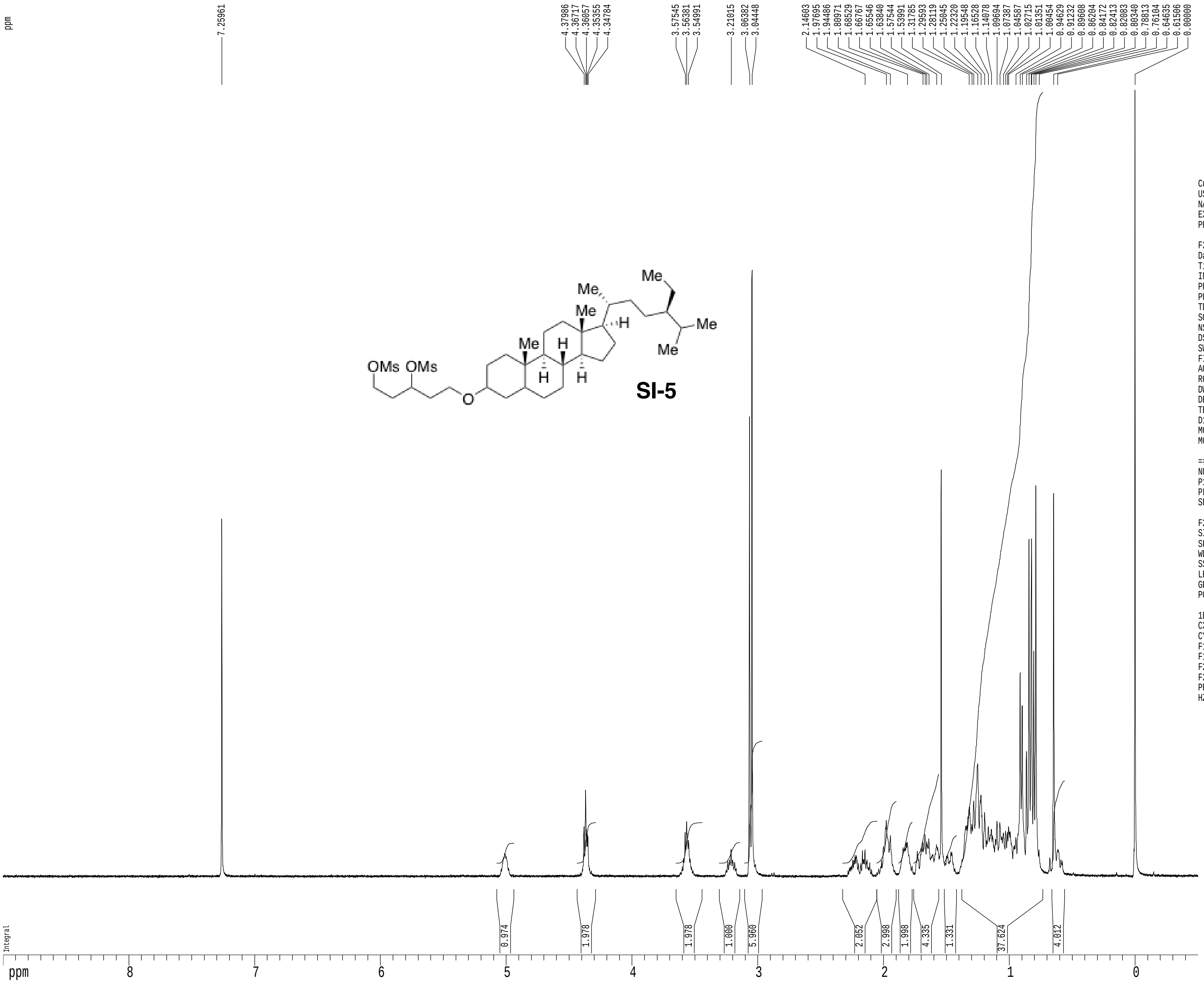

Current Data Parameters
USER tthane
NAME TATii222c
EXPNO 1
PROCNO 1

F2 - Acquisition Parameters
Date_ 20190617
Time 12.03
INSTRUM drx400
PROBHD 5 mm QNP H/F/P
PULPROG zg30
TD 65536
SOLVENT CDCl3
NS 24
DS 2
SWH 6410.256 Hz
FIDRES 0.097813 Hz
AQ 5.1118579 sec
RG 456.1
DW 78.000 usec
DE 4.50 usec
TE 298.1 K
D1 0.10000000 sec
MCREST 0.00000000 sec
MCWRK 0.01500000 sec

======== CHANNEL f1 ========
NUC1 1H
P1 12.00 usec
PL1 -1.10 dB
SFO1 400.1328009 MHz

F2 - Processing parameters
SI 65536
SF 400.1300213 MHz
WDW no
SSB 0
LB 0.00 Hz
GB 0
PC 2.00

1D NMR plot parameters
CX 22.80 cm
CY 15.00 cm
F1P 9.000 ppm
F1 3601.17 Hz
F2P -0.500 ppm
F2 -200.06 Hz
PPMCM 0.41667 ppm/cm
HZCM 166.72086 Hz/cm

1H spectrum

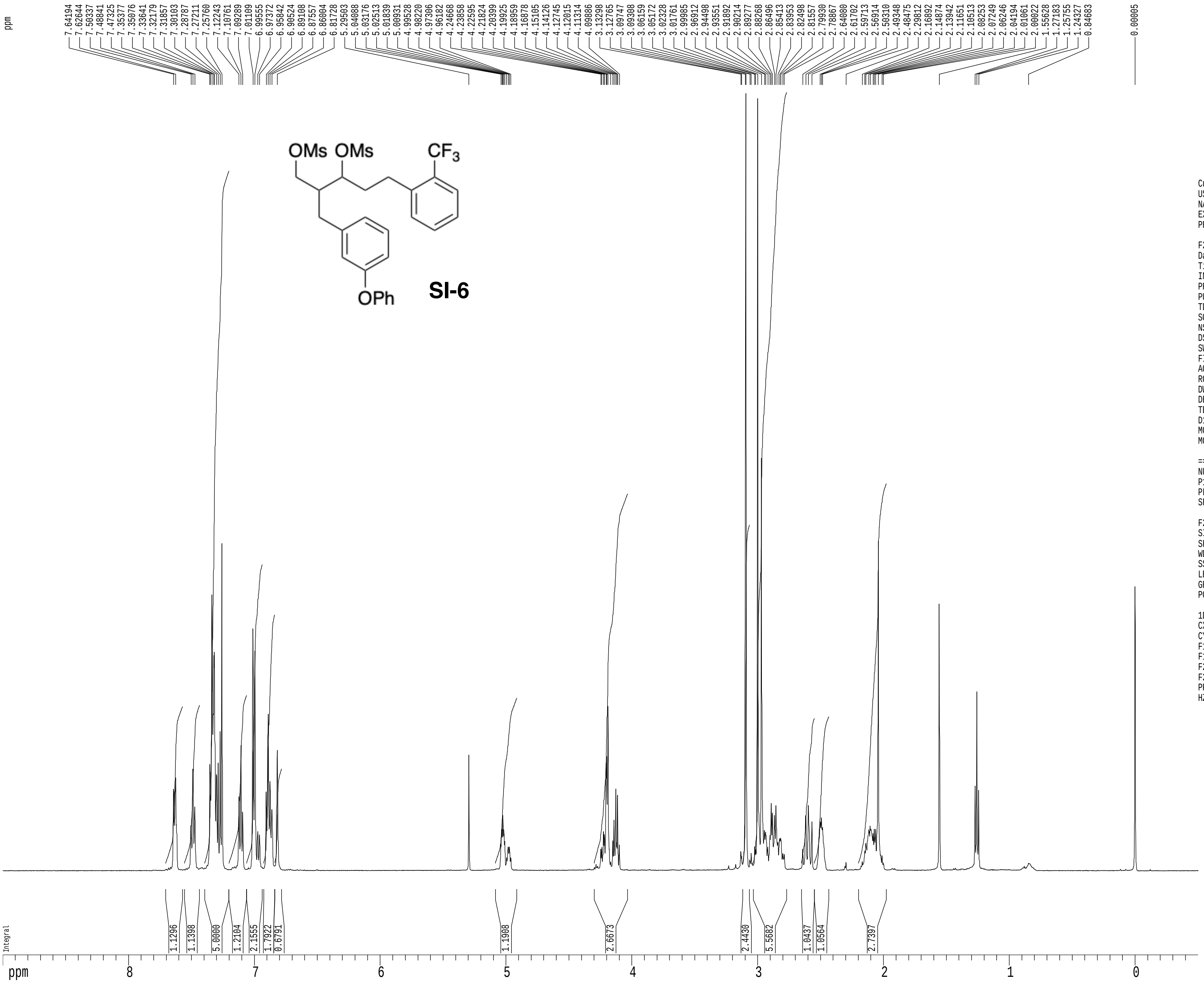

Current Data Parameters
NAME tmm-1-280
USER mcginnit
EXPNO 5
PROCNO 1

F2 - Acquisition Parameters
Date_ 20191123
Time 14.54
INSTRUM cryo500
PROBHD 5 mm CPTCI 1H-
PULPROG zg30
TD 81728
SOLVENT CDCl3
NS 8
DS 2
SWH 8012.820 Hz
FIDRES 0.098043 Hz
AQ 5.0998774 sec
RG 5.7
DW 62.400 usec
DE 6.00 usec
TE 298.0 K
D1 0.10000000 sec
MCREST 0.00000000 sec
MCWRK 0.01500000 sec

======== CHANNEL f1 ========
NUC1 1H
P1 7.50 usec
PL1 1.60 dB
SFO1 500.2235015 MHz

F2 - Processing parameters
SI 65536
SF 500.2200325 MHz
WDW EM
SSB 0
LB 0.30 Hz
GB 0
PC 1.00

1D NMR plot parameters
CX 22.80 cm
CY 15.00 cm
F1P 9.000 ppm
F1 4501.98 Hz
F2P -0.500 ppm
F2 -250.11 Hz
PPMCM 0.41667 ppm/cm
HZCM 208.42502 Hz/cm

1H spectrum

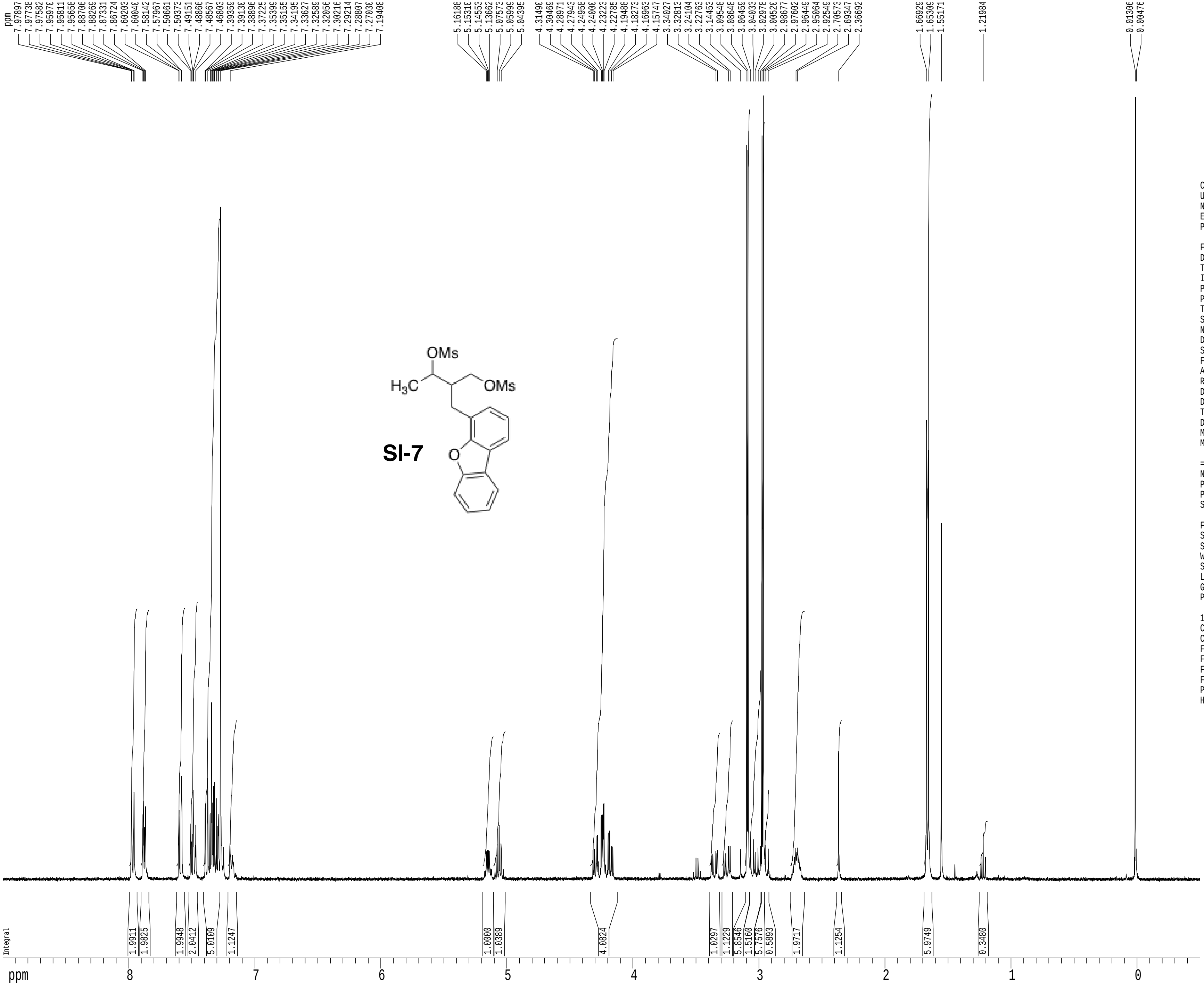


```
Current Data Parameters
USER              tthane
NAME          TATiii138c
EXPNO                  1
PROCNO                 1

F2 - Acquisition Parameters
Date_           20191204
Time               13.48
INSTRUM           drx400
PROBHD   5 mm Multinucl
PULPROG             zg30
TD                 65536
SOLVENT           CDCl3T
NS                     8
DS                     2
SWH             6410.256 Hz
FIDRES          0.097813 Hz
AQ             5.1118579 sec
RG                 812.7
DW                78.000 usec
DE                  4.50 usec
TE                 297.9 K
D1            0.10000000 sec
MCREST        0.00000000 sec
MCWRK         0.01500000 sec

======== CHANNEL f1 ========
NUC1                  1H
P1                 12.00 usec
PL1                -1.10 dB
SFO1         400.1328009 MHz

F2 - Processing parameters
SI                 65536
SF           400.1300175 MHz
WDW                   no
SSB                    0
LB                  0.00 Hz
GB                     0
PC                  2.00

1D NMR plot parameters
CX                 22.80 cm
CY                 15.00 cm
F1P                9.000 ppm
F1               3601.17 Hz
F2P               -0.500 ppm
F2               -200.06 Hz
PPMCM            0.41667 ppm/cm
HZCM           166.72084 Hz/cm
```

1H spectrum

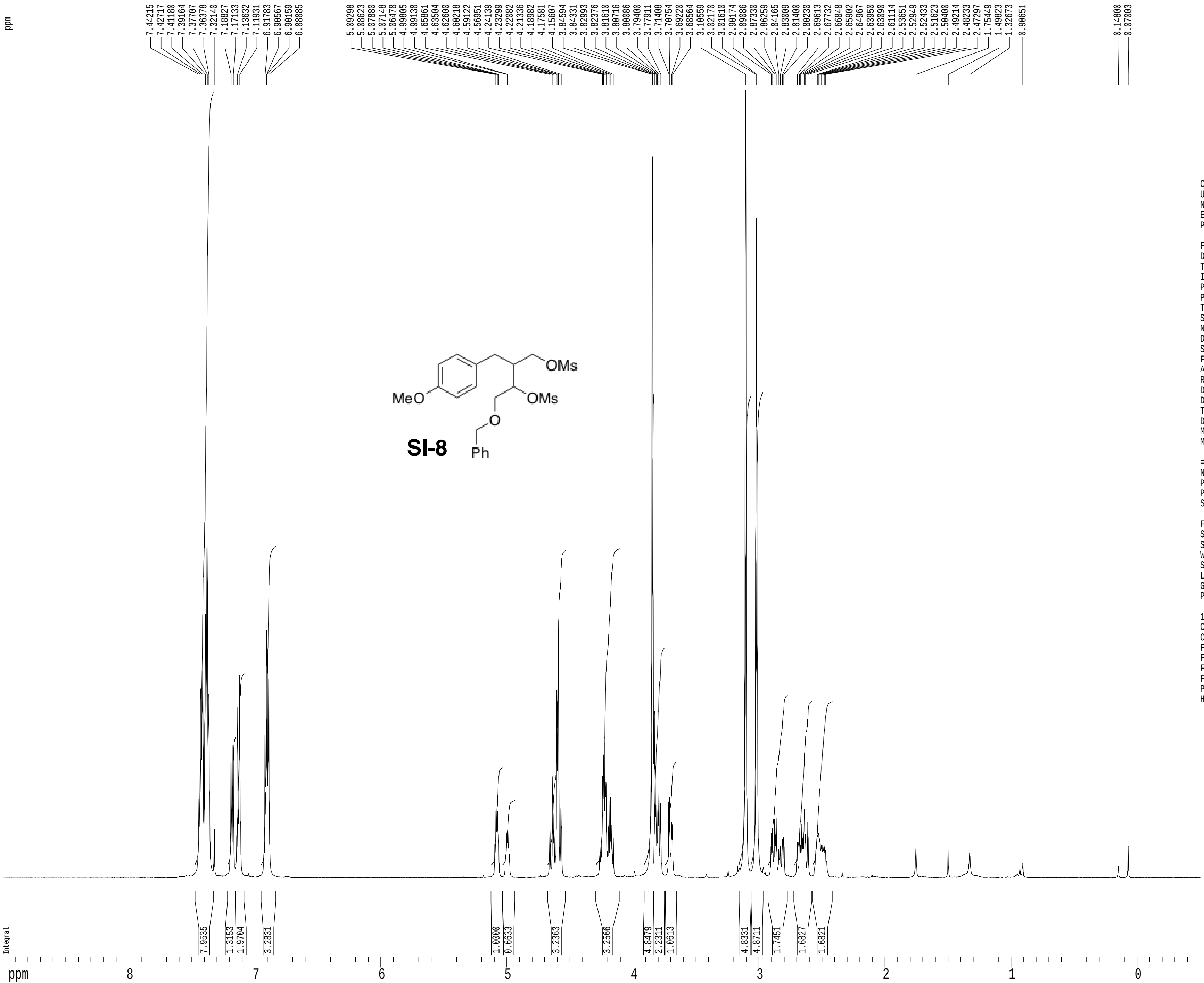


```
Current Data Parameters
USER             tthane
NAME       TATiii128char
EXPNO                 3
PROCNO                1

F2 - Acquisition Parameters
Date_          20211216
Time              12.04
INSTRUM         cryo500
PROBHD   5 mm CPTCI 1H-
PULPROG            zg30
TD                81728
SOLVENT          CDCl3T
NS                    8
DS                    2
SWH            8012.820 Hz
FIDRES         0.098043 Hz
AQ            5.0998774 sec
RG                  4.5
DW               62.400 usec
DE                 6.00 usec
TE                298.0 K
D1           0.10000000 sec
MCREST       0.00000000 sec
MCWRK        0.01500000 sec

======== CHANNEL f1 ========
NUC1                 1H
P1                 9.75 usec
PL1                1.60 dB
SFO1        500.2235015 MHz

F2 - Processing parameters
SI                65536
SF          500.2200000 MHz
WDW                  no
SSB                   0
LB                 0.00 Hz
GB                    0
PC                 1.00

1D NMR plot parameters
CX                22.80 cm
CY                15.00 cm
F1P               9.000 ppm
F1              4501.98 Hz
F2P              -0.500 ppm
F2              -250.11 Hz
PPMCM           0.41667 ppm/cm
HZCM          208.42500 Hz/cm
```

Z-restored spin-echo 13C spectrum with 1H decoupling

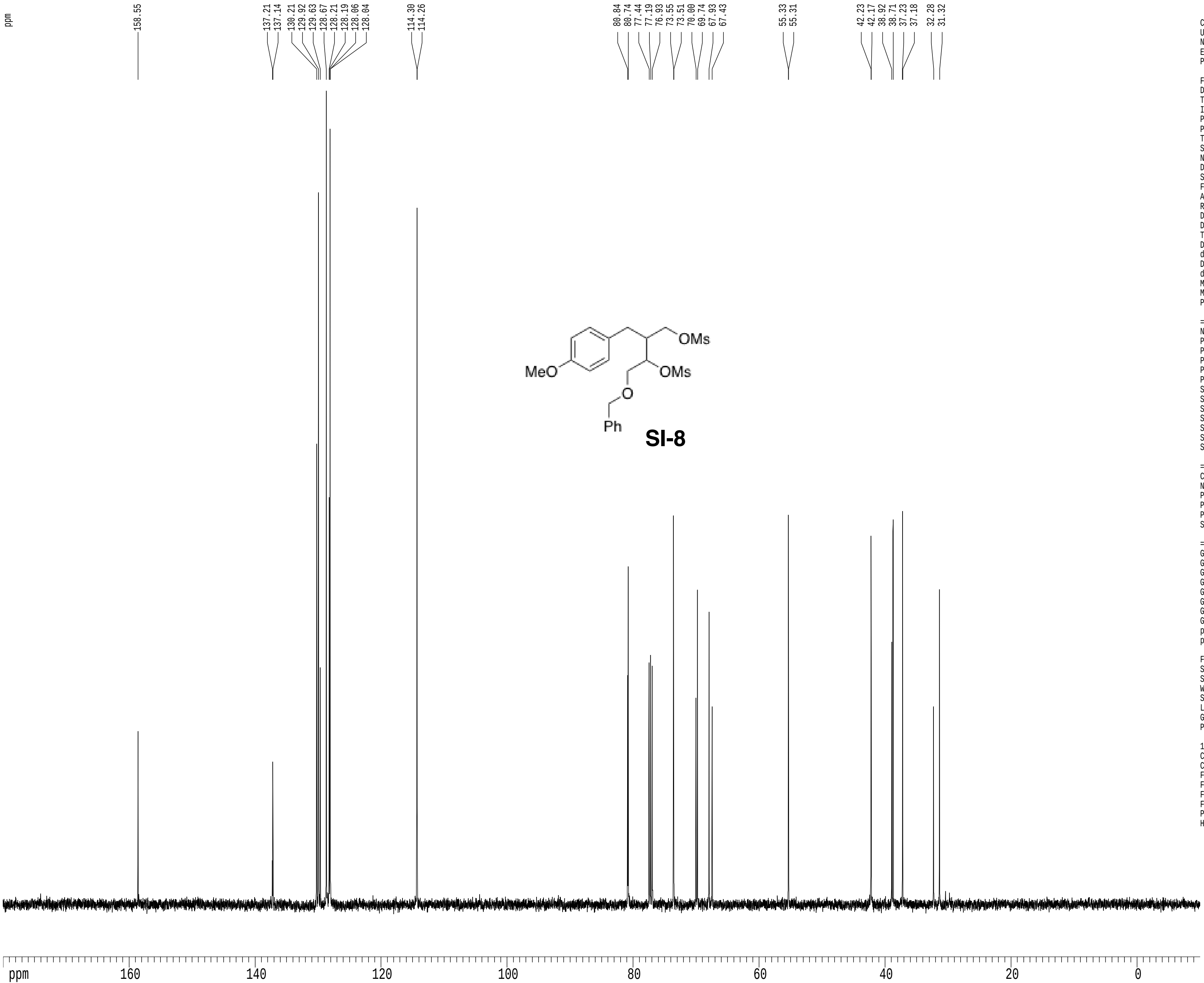

SI-8

Current Data Parameters
USER tthane
NAME TATiii128char
EXPNO 4
PROCNO 1

F2 - Acquisition Parameters
Date_ 20211216
Time 12.06
INSTRUM cryo500
PROBHD 5 mm CPTCI 1H-
PULPROG SpinEchopg30gp2.prd
TD 65536
SOLVENT CDCl3
NS 184
DS 16
SWH 30303.031 Hz
FIDRES 0.462388 Hz
AQ 1.0813940 sec
RG 7298.2
DW 16.500 usec
DE 6.00 usec
TE 298.0 K
D1 0.25000000 sec
d11 0.03000000 sec
D16 0.00020000 sec
d17 0.00019600 sec
MCREST 0.00000000 sec
MCWRK 0.01500000 sec
P2 37.70 usec

======== CHANNEL f1 ========
NUC1 13C
P1 18.85 usec
P12 2000.00 usec
P20 500.00 usec
PL0 120.00 dB
PL1 -1.00 dB
SFO1 125.7942548 MHz
SP2 1.55 dB
SP4 1.55 dB
SPNAM2 Crp60comp.4
SPNAM4 Crp60,0.5,20.1
SPOFF2 0.00 Hz
SPOFF4 0.00 Hz

======== CHANNEL f2 ========
CPDPRG2 waltz16
NUC2 1H
PCPD2 100.00 usec
PL2 1.60 dB
PL12 22.00 dB
SFO2 500.2225011 MHz

====== GRADIENT CHANNEL =====
GPNAM1 SINE.100
GPNAM2 SINE.100
GPX1 0.00 %
GPX2 0.00 %
GPY1 0.00 %
GPY2 0.00 %
GPZ1 30.00 %
GPZ2 50.00 %
p15 500.00 usec
p16 1000.00 usec

F2 - Processing parameters
SI 65536
SF 125.7804190 MHz
WDW EM
SSB 0
LB 1.00 Hz
GB 0
PC 2.00

1D NMR plot parameters
CX 22.80 cm
CY 15.65 cm
F1P 180.000 ppm
F1 22640.47 Hz
F2P -10.000 ppm
F2 -1257.80 Hz
PPMCM 8.33333 ppm/cm
HZCM 1048.17017 Hz/cm

1H spectrum

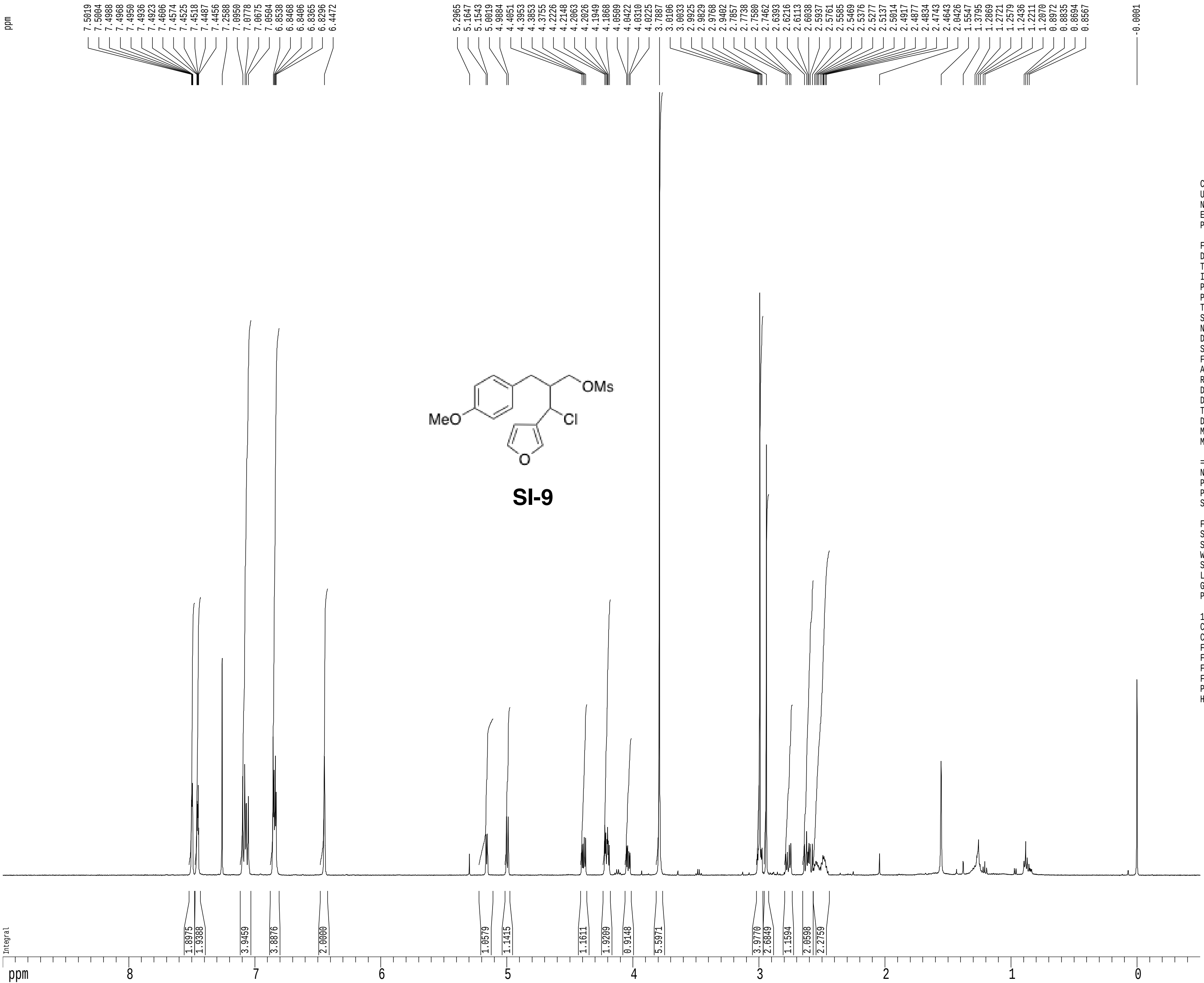

Current Data Parameters
USER tthane
NAME TATiii150c
EXPNO 4
PROCNO 1

F2 - Acquisition Parameters
Date_ 20191205
Time 18.14
INSTRUM cryo500
PROBHD 5 mm CPTCI 1H-
PULPROG zg30
TD 81728
SOLVENT CDCl3
NS 8
DS 2
SWH 8012.820 Hz
FIDRES 0.098043 Hz
AQ 5.0998774 sec
RG 8
DW 62.400 usec
DE 6.00 usec
TE 298.0 K
D1 0.10000000 sec
MCREST 0.00000000 sec
MCWRK 0.01500000 sec

======== CHANNEL f1 ========
NUC1 1H
P1 7.50 usec
PL1 1.60 dB
SFO1 500.2235015 MHz

F2 - Processing parameters
SI 65536
SF 500.2200316 MHz
WDW no
SSB 0
LB 0.00 Hz
GB 0
PC 1.00

1D NMR plot parameters
CX 22.80 cm
CY 15.00 cm
F1P 9.000 ppm
F1 4501.98 Hz
F2P -0.500 ppm
F2 -250.11 Hz
PPMCM 0.41667 ppm/cm
HZCM 208.42502 Hz/cm

1H spectrum

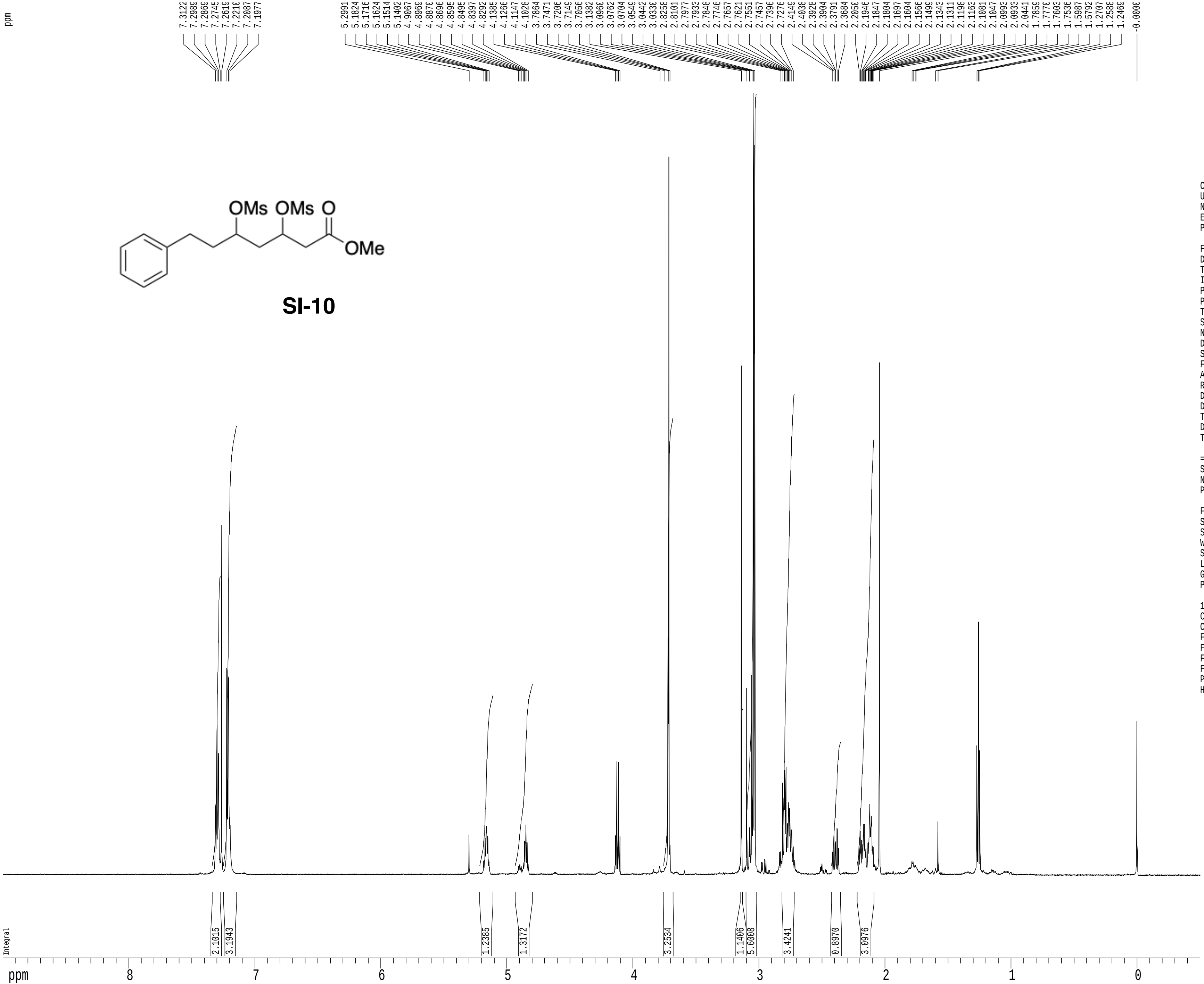


```
Current Data Parameters
USER            mcginnit
NAME     tmm-4-167-char
EXPNO                  1
PROCNO                 1

F2 - Acquisition Parameters
Date_           20220412
Time               14.31
INSTRUM            av600
PROBHD   5 mm CPBBO BB-
PULPROG             zg30
TD                 98074
SOLVENT            CDCl3
NS                     8
DS                     2
SWH            9615.385 Hz
FIDRES         0.098042 Hz
AQ            5.0998979 sec
RG                    10
DW                52.000 usec
DE                 14.23 usec
TE                 298.0 K
D1            0.10000000 sec
TD0                    1

======== CHANNEL f1 ========
SFO1        600.1342009 MHz
NUC1                 1H
P1                  9.50 usec

F2 - Processing parameters
SI                 65536
SF          600.1300339 MHz
WDW                   no
SSB                    0
LB                  0.00 Hz
GB                     0
PC                  1.00

1D NMR plot parameters
CX                 22.80 cm
CY                 15.00 cm
F1P                9.000 ppm
F1               5401.17 Hz
F2P               -0.500 ppm
F2               -300.06 Hz
PPMCM            0.41667 ppm/cm
HZCM           250.05418 Hz/cm
```

13C spectrum

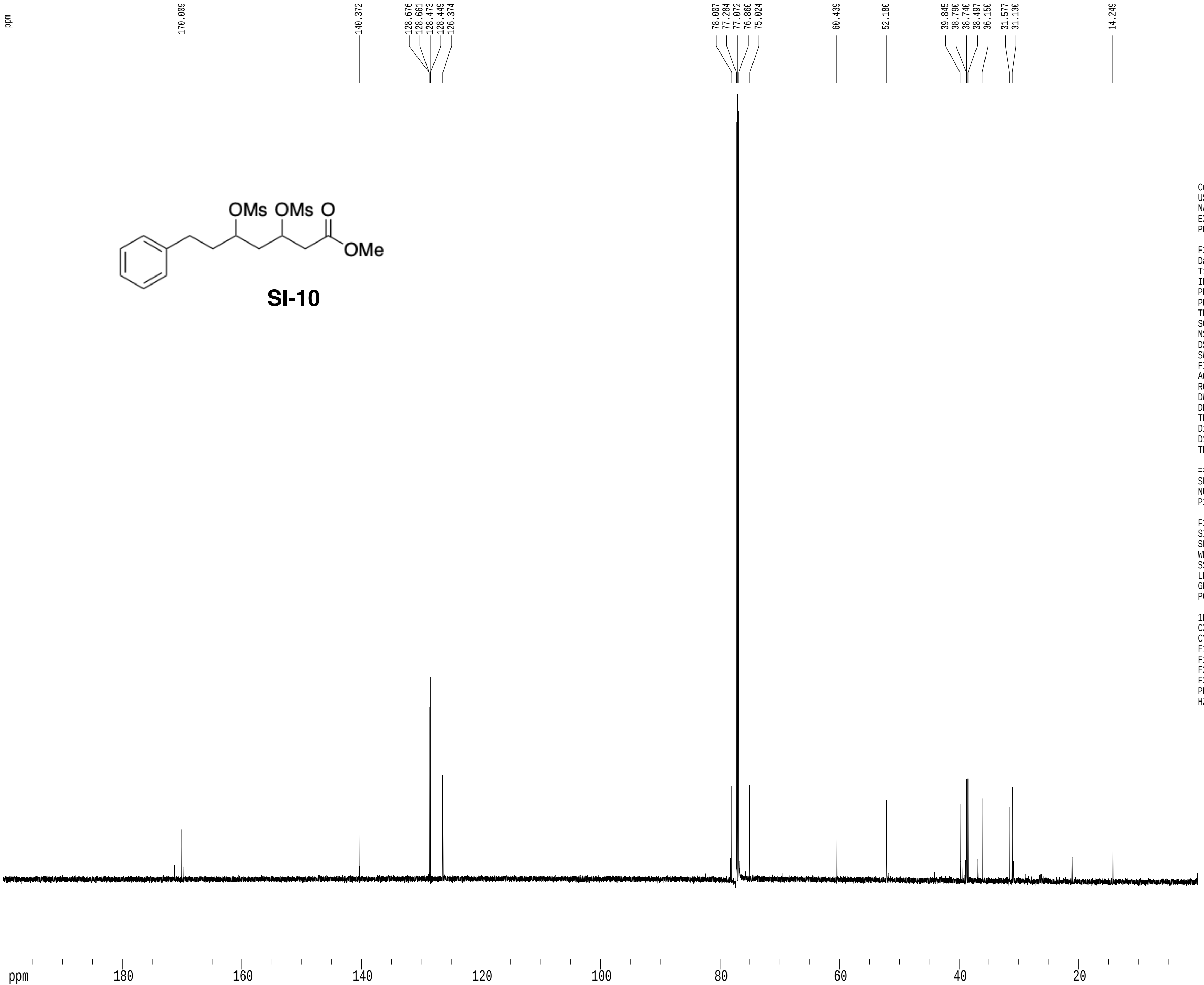

SI-10

```
Current Data Parameters
USER              mcginnit
NAME        tmm-4-167-char
EXPNO                    2
PROCNO                   1

F2 - Acquisition Parameters
Date_             20220412
Time                 14.36
INSTRUM              av600
PROBHD   5 mm CPBBO BB-
PULPROG             zgdc30
TD                   65536
SOLVENT              CDCl3
NS                     196
DS                       4
SWH             36231.883 Hz
FIDRES           0.552855 Hz
AQ              0.9044468 sec
RG                    2050
DW                  13.800 usec
DE                   19.63 usec
TE                   298.0 K
D1              0.40000001 sec
D11             0.03000000 sec
TD0                      1

======== CHANNEL f1 ========
SFO1          150.9194080 MHz
NUC1                   13C
P1                   10.10 usec

F2 - Processing parameters
SI                   65536
SF             150.9028085 MHz
WDW                     no
SSB                      0
LB                    0.00 Hz
GB                       0
PC                    1.00

1D NMR plot parameters
CX                   22.80 cm
CY                   15.00 cm
F1P                200.000 ppm
F1                30180.56 Hz
F2P                  0.000 ppm
F2                    0.00 Hz
PPMCM              8.77193 ppm/cm
HZCM            1323.70886 Hz/cm
```

1H spectrum

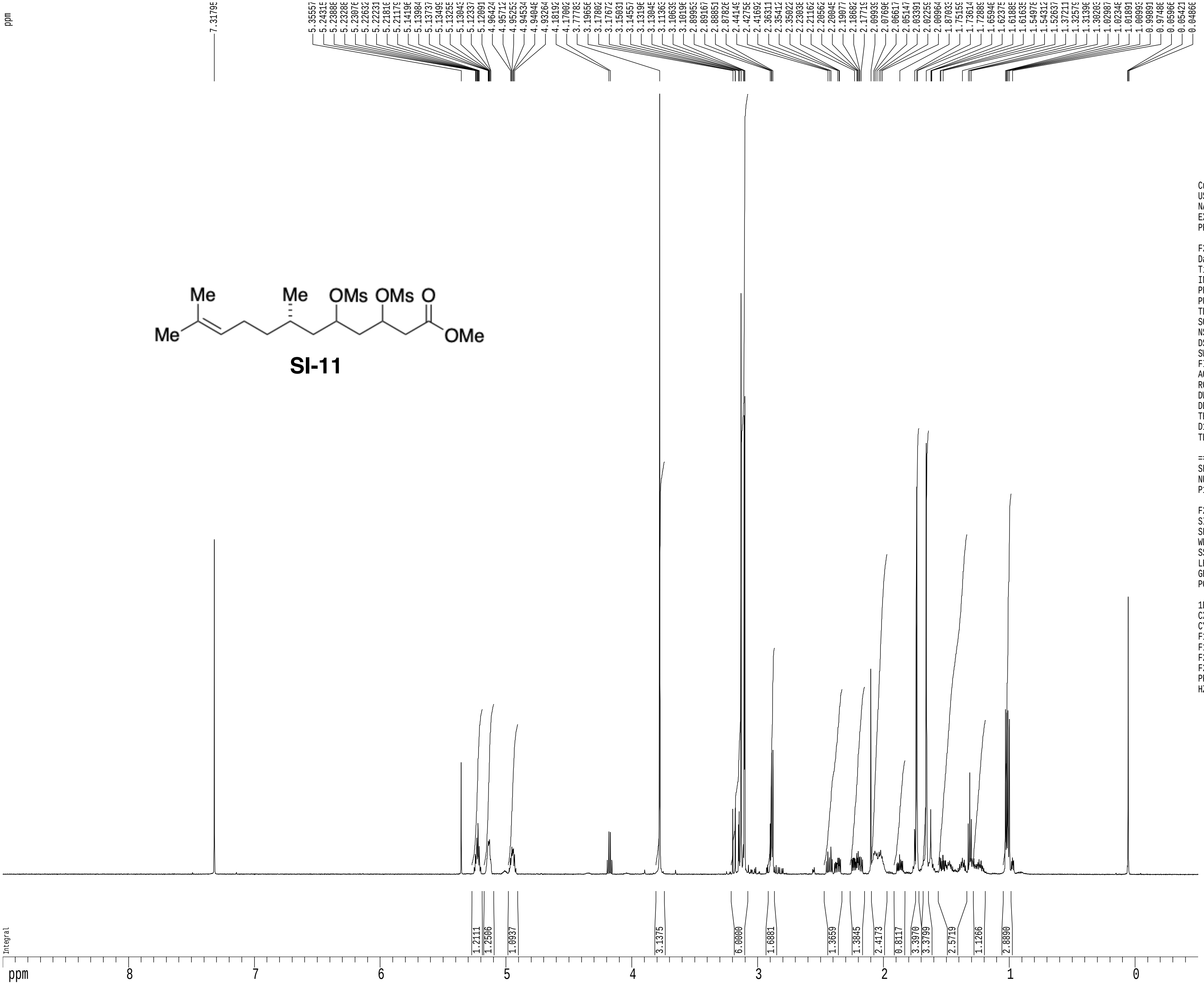

SI-11


```
Current Data Parameters
USER            mcginnit
NAME      tmm-4-173-char
EXPNO                  1
PROCNO                 1

F2 - Acquisition Parameters
Date_           20220418
Time                9.58
INSTRUM            av600
PROBHD   5 mm CPBBO BB-
PULPROG             zg30
TD                 98074
SOLVENT            CDCl3
NS                     8
DS                     2
SWH             9615.385 Hz
FIDRES          0.098042 Hz
AQ             5.0998979 sec
RG                    10
DW                52.000 usec
DE                 14.23 usec
TE                 298.0 K
D1            0.10000000 sec
TD0                    1

======== CHANNEL f1 ========
SFO1         600.1342009 MHz
NUC1                  1H
P1                  9.50 usec

F2 - Processing parameters
SI                 65536
SF           600.1300000 MHz
WDW                   no
SSB                    0
LB                  0.00 Hz
GB                     0
PC                  1.00

1D NMR plot parameters
CX                 22.80 cm
CY                 15.00 cm
F1P                9.000 ppm
F1               5401.17 Hz
F2P               -0.500 ppm
F2               -300.06 Hz
PPMCM            0.41667 ppm/cm
HZCM            250.05418 Hz/cm
```

13C spectrum with 1H decoupling

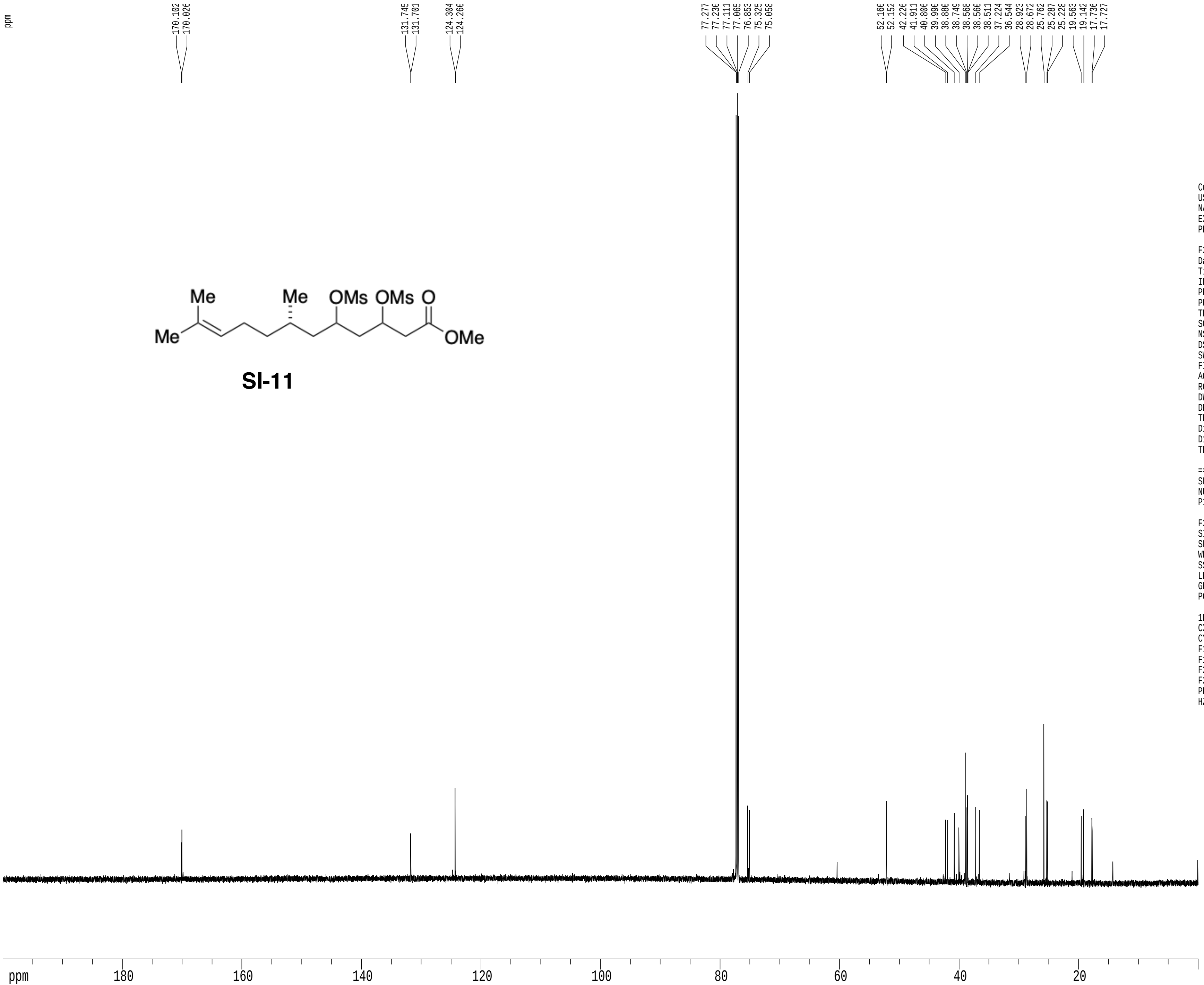

Current Data Parameters
USER mcginnit
NAME tmm-4-173-char
EXPNO 3
PROCNO 1

F2 - Acquisition Parameters
Date_ 20220418
Time 10.05
INSTRUM av600
PROBHD 5 mm CPBBO BB-
PULPROG zgdc30
TD 65536
SOLVENT CDCl3
NS 224
DS 4
SWH 36231.883 Hz
FIDRES 0.552855 Hz
AQ 0.9044468 sec
RG 2050
DW 13.800 usec
DE 19.63 usec
TE 298.0 K
D1 0.40000001 sec
D11 0.03000000 sec
TD0 1

======== CHANNEL f1 ========
SFO1 150.9194080 MHz
NUC1 13C
P1 10.10 usec

F2 - Processing parameters
SI 65536
SF 150.9028085 MHz
WDW no
SSB 0
LB 0.00 Hz
GB 0
PC 1.00

1D NMR plot parameters
CX 22.80 cm
CY 15.00 cm
F1P 200.000 ppm
F1 30180.56 Hz
F2P 0.000 ppm
F2 0.00 Hz
PPMCM 8.77193 ppm/cm
HZCM 1323.70886 Hz/cm

1H Spectrum

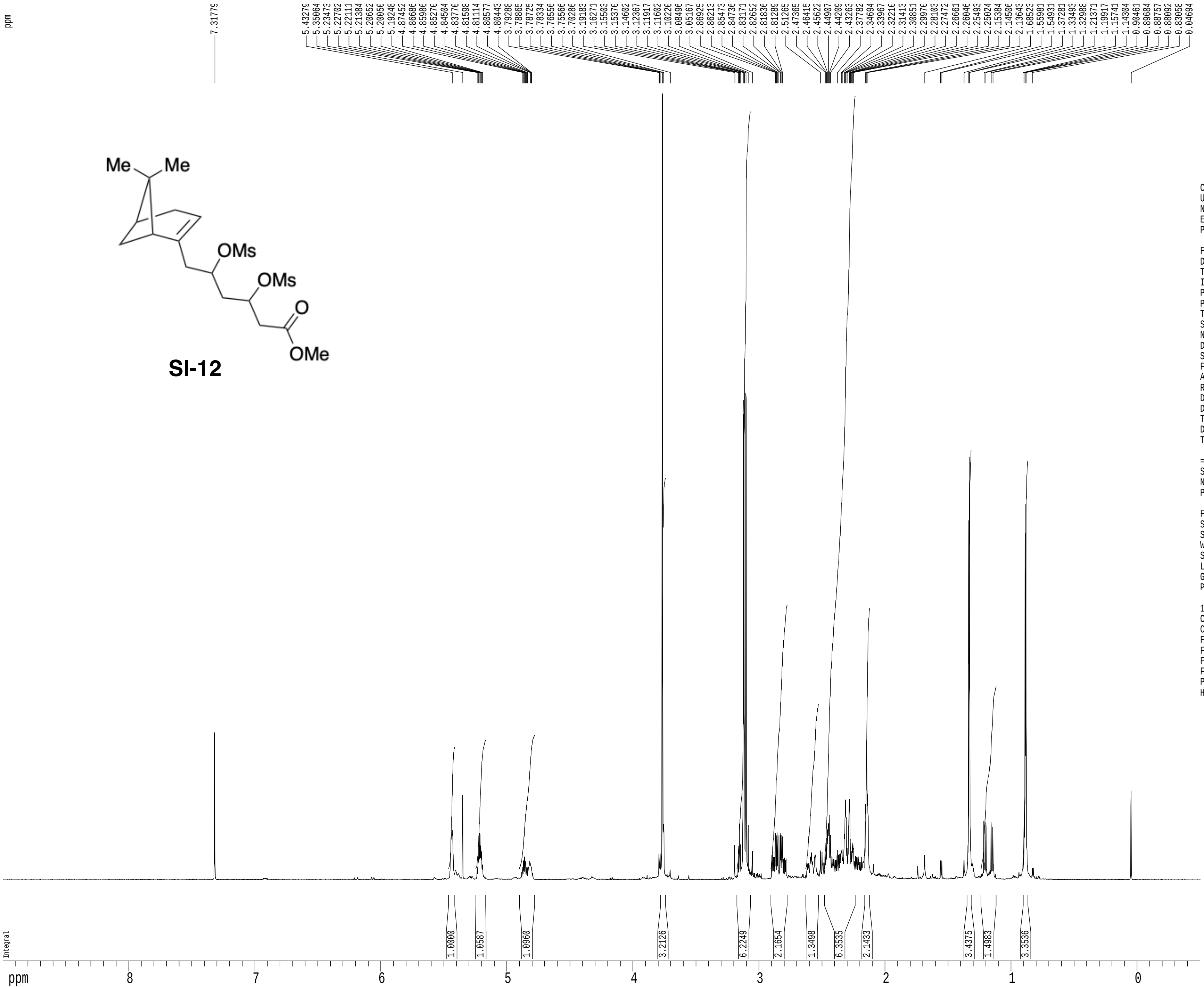

SI-12

```
Current Data Parameters
USER          mcginnit
NAME     tmm-4-114-char
EXPNO                1
PROCNO               1

F2 - Acquisition Parameters
Date_         20220223
Time             12.19
INSTRUM          av600
PROBHD   5 mm CPBBO BB-
PULPROG           zg30
TD               98074
SOLVENT          CDCl3
NS                   8
DS                   2
SWH           9615.385 Hz
FIDRES        0.098042 Hz
AQ           5.0998979 sec
RG                   9
DW              52.000 usec
DE               14.23 usec
TE               298.0 K
D1          0.10000000 sec
TD0                  1

======== CHANNEL f1 ========
SFO1       600.1342009 MHz
NUC1                1H
P1                9.50 usec

F2 - Processing parameters
SI               65536
SF         600.1300000 MHz
WDW                 no
SSB                  0
LB                0.00 Hz
GB                   0
PC                1.00

1D NMR plot parameters
CX               22.80 cm
CY               15.00 cm
F1P              9.000 ppm
F1             5401.17 Hz
F2P             -0.500 ppm
F2             -300.06 Hz
PPMCM          0.41667 ppm/cm
HZCM         250.05418 Hz/cm
```

13C Spectrum

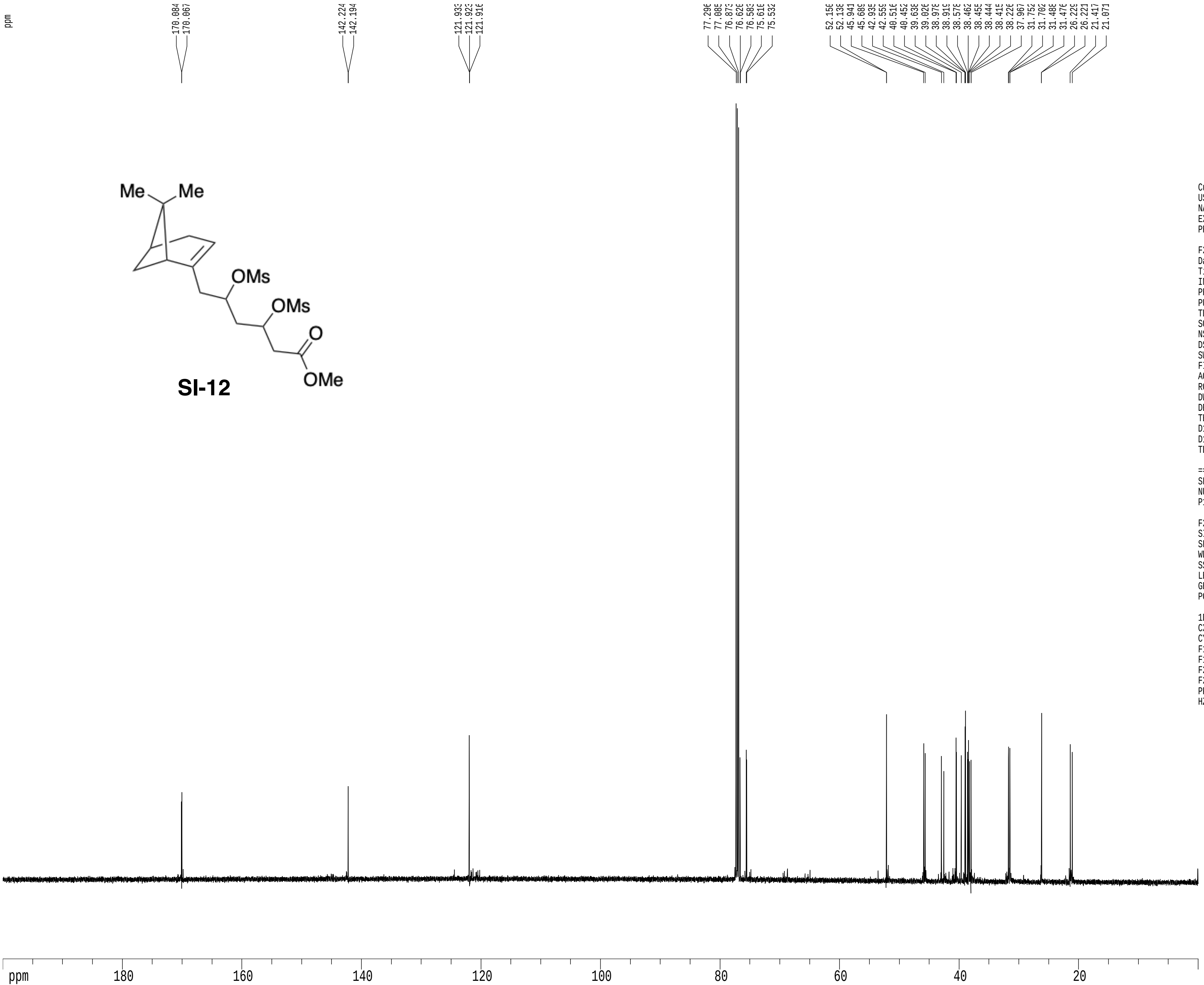

SI-12

```
Current Data Parameters
USER          mcginnit
NAME     tmm-4-114-char
EXPNO                 2
PROCNO                1

F2 - Acquisition Parameters
Date_          20220223
Time              12.23
INSTRUM           av600
PROBHD   5 mm CPBBO BB-
PULPROG          zgdc30
TD                65536
SOLVENT           CDCl3
NS                  208
DS                    4
SWH           36231.883 Hz
FIDRES         0.552855 Hz
AQ            0.9044468 sec
RG                 2050
DW               13.800 usec
DE                19.63 usec
TE                298.0 K
D1           0.40000001 sec
D11          0.03000000 sec
TD0                   1

======== CHANNEL f1 ========
SFO1        150.9194080 MHz
NUC1                13C
P1                10.10 usec

F2 - Processing parameters
SI                65536
SF          150.9028085 MHz
WDW                  no
SSB                   0
LB                 0.00 Hz
GB                    0
PC                 1.00

1D NMR plot parameters
CX                22.80 cm
CY                15.00 cm
F1P             200.000 ppm
F1             30180.56 Hz
F2P               0.000 ppm
F2                 0.00 Hz
PPMCM           8.77193 ppm/cm
HZCM         1323.70886 Hz/cm
```

1H spectrum

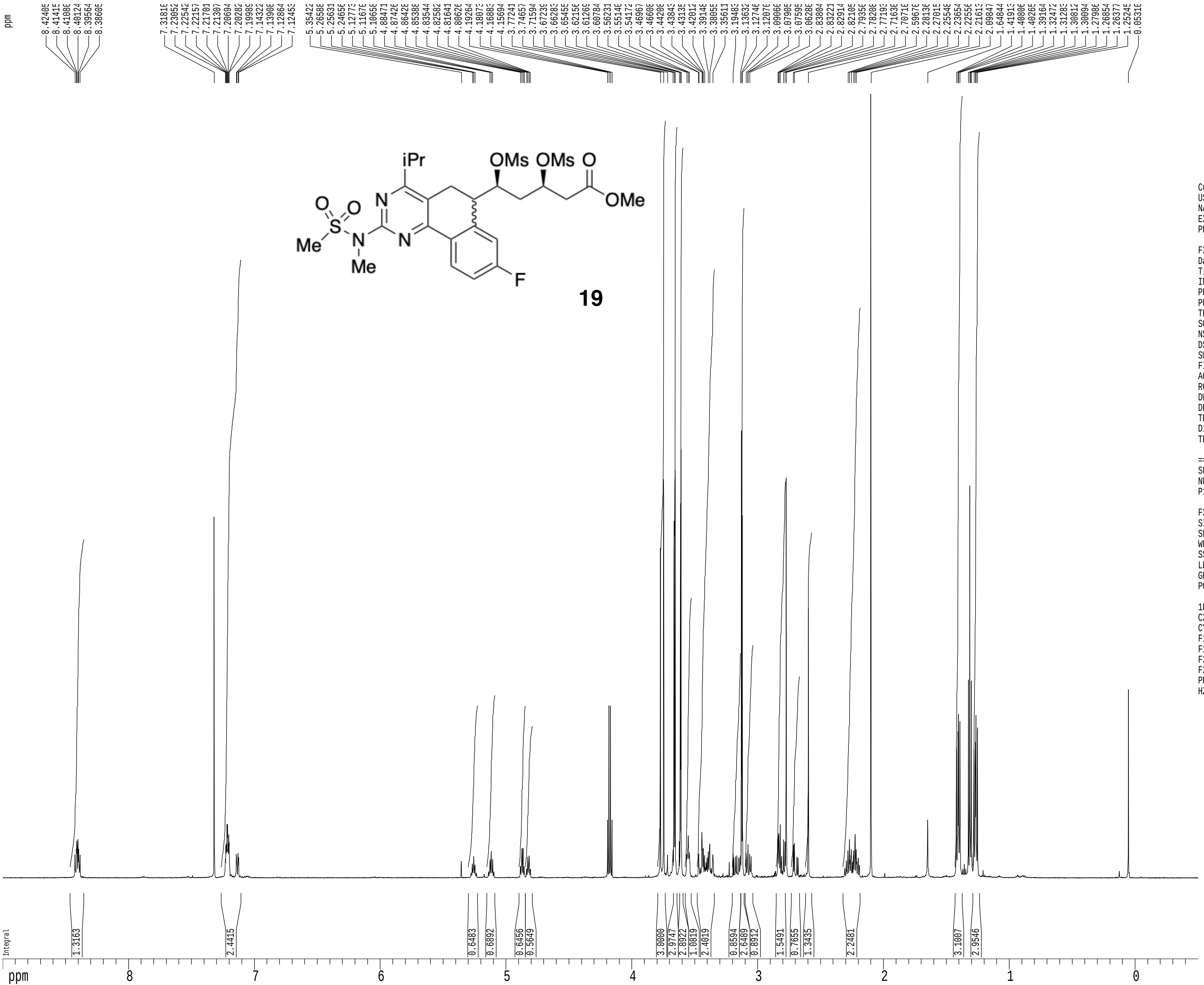


```
Current Data Parameters
USER           mcginnit
NAME      tmm-4-43-char
EXPNO                 1
PROCNO                1

F2 - Acquisition Parameters
Date_          20220224
Time              16.03
INSTRUM           av600
PROBHD   5 mm CPBBO BB-
PULPROG            zg30
TD                98074
SOLVENT           CDCl3
NS                    8
DS                    2
SWH           9615.385 Hz
FIDRES        0.098042 Hz
AQ           5.0998979 sec
RG                   10
DW               52.000 usec
DE                14.23 usec
TE                297.9 K
D1           0.10000000 sec
TD0                   1

======== CHANNEL f1 ========
SFO1       600.1342009 MHz
NUC1                 1H
P1                 9.50 usec

F2 - Processing parameters
SI                65536
SF          600.1300000 MHz
WDW                  no
SSB                   0
LB                 0.00 Hz
GB                    0
PC                 1.00

1D NMR plot parameters
CX                22.80 cm
CY                15.00 cm
F1P               9.000 ppm
F1              5401.17 Hz
F2P              -0.500 ppm
F2              -300.06 Hz
PPMCM           0.41667 ppm/cm
HZCM           250.05418 Hz/cm
```

13C spectrum

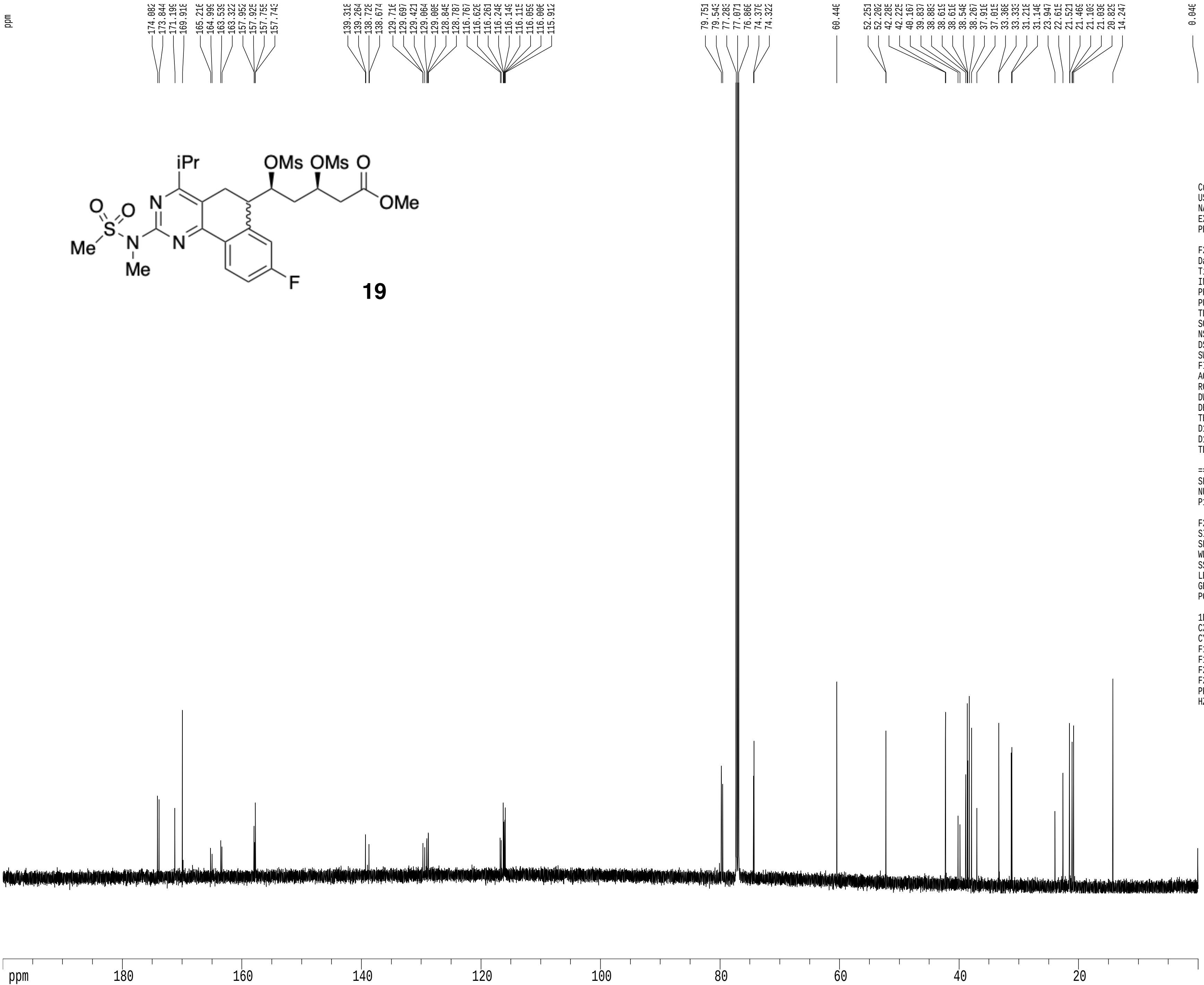

Current Data Parameters
USER mcginnit
NAME tmm-4-43-char
EXPNO 2
PROCNO 1

F2 - Acquisition Parameters
Date_ 20220224
Time 16.08
INSTRUM av600
PROBHD 5 mm CPBBO BB-
PULPROG zgdc30
TD 65536
SOLVENT CDCl3
NS 302
DS 4
SWH 36231.883 Hz
FIDRES 0.552855 Hz
AQ 0.9044468 sec
RG 2050
DW 13.800 usec
DE 19.63 usec
TE 298.0 K
D1 0.40000001 sec
D11 0.03000000 sec
TD0 1

======== CHANNEL f1 ========
SFO1 150.9194080 MHz
NUC1 13C
P1 10.10 usec

F2 - Processing parameters
SI 65536
SF 150.9028085 MHz
WDW no
SSB 0
LB 0.00 Hz
GB 0
PC 1.00

1D NMR plot parameters
CX 22.80 cm
CY 50.00 cm
F1P 200.000 ppm
F1 30180.56 Hz
F2P 0.000 ppm
F2 0.00 Hz
PPMCM 8.77193 ppm/cm
HZCM 1323.70886 Hz/cm

19F spectrum

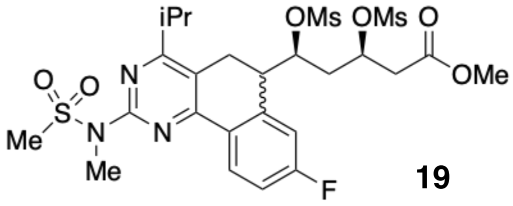

19

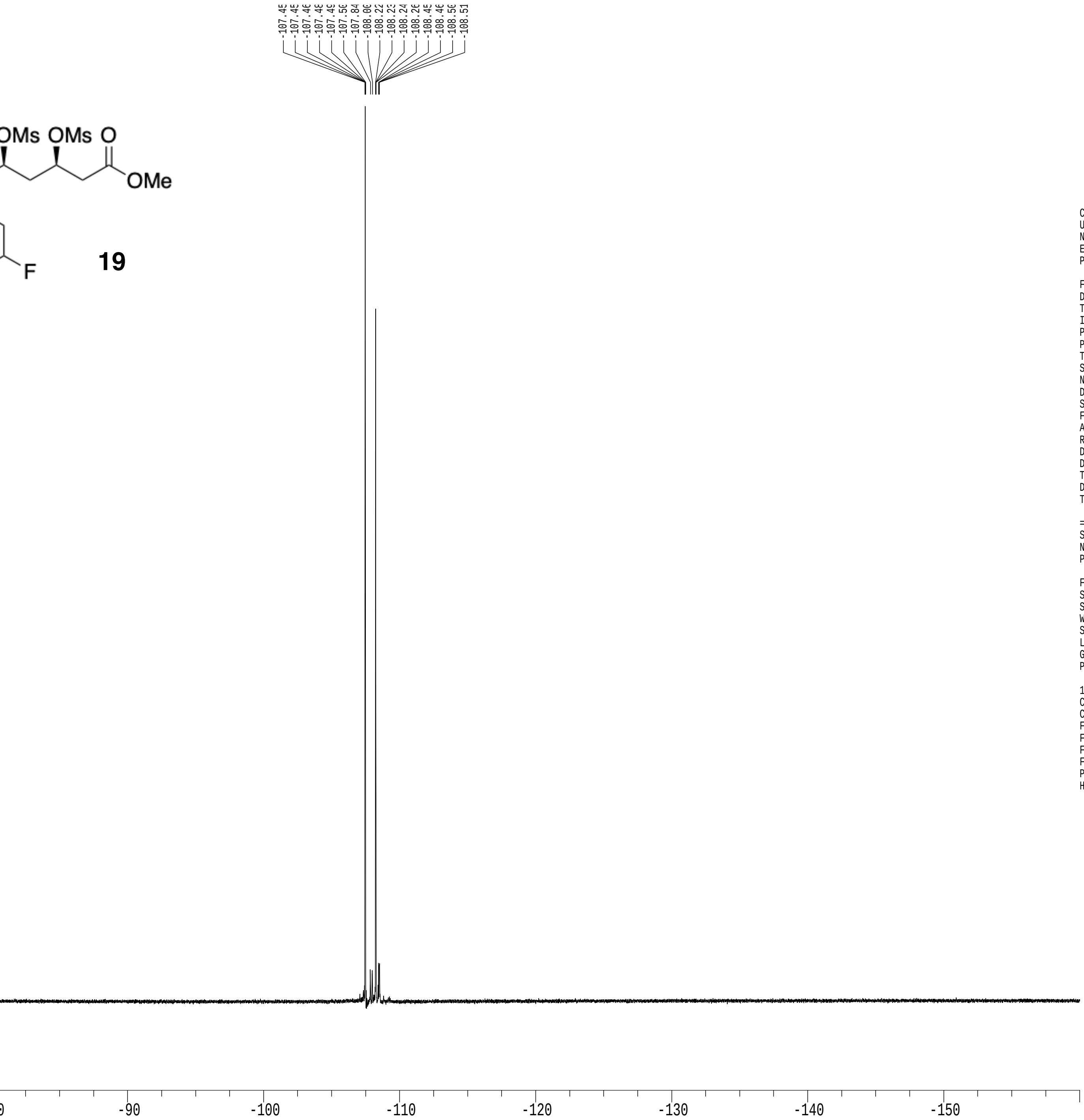

Current Data Parameters
USER mcginnit
NAME tmm-4-43-char
EXPNO 3
PROCNO 1

F2 - Acquisition Parameters
Date_ 20220224
Time 16.18
INSTRUM av600
PROBHD 5 mm CPBBO BB-
PULPROG zgflqn30
TD 131072
SOLVENT CDCl3
NS 16
DS 2
SWH 178571.422 Hz
FIDRES 1.362392 Hz
AQ 0.3670516 sec
RG 575
DW 2.800 usec
DE 18.00 usec
TE 298.0 K
D1 3.00000000 sec
TD0 1

======== CHANNEL f1 ========
SFO1 564.6299196 MHz
NUC1 19F
P1 18.25 usec

F2 - Processing parameters
SI 131072
SF 564.6863858 MHz
WDW no
SSB 0
LB 0.00 Hz
GB 0
PC 1.00

1D NMR plot parameters
CX 22.80 cm
CY 15.00 cm
F1P -60.000 ppm
F1 -33881.19 Hz
F2P -160.000 ppm
F2 -90349.83 Hz
PPMCM 4.38596 ppm/cm
HZCM 2476.69482 Hz/cm

1H spectrum

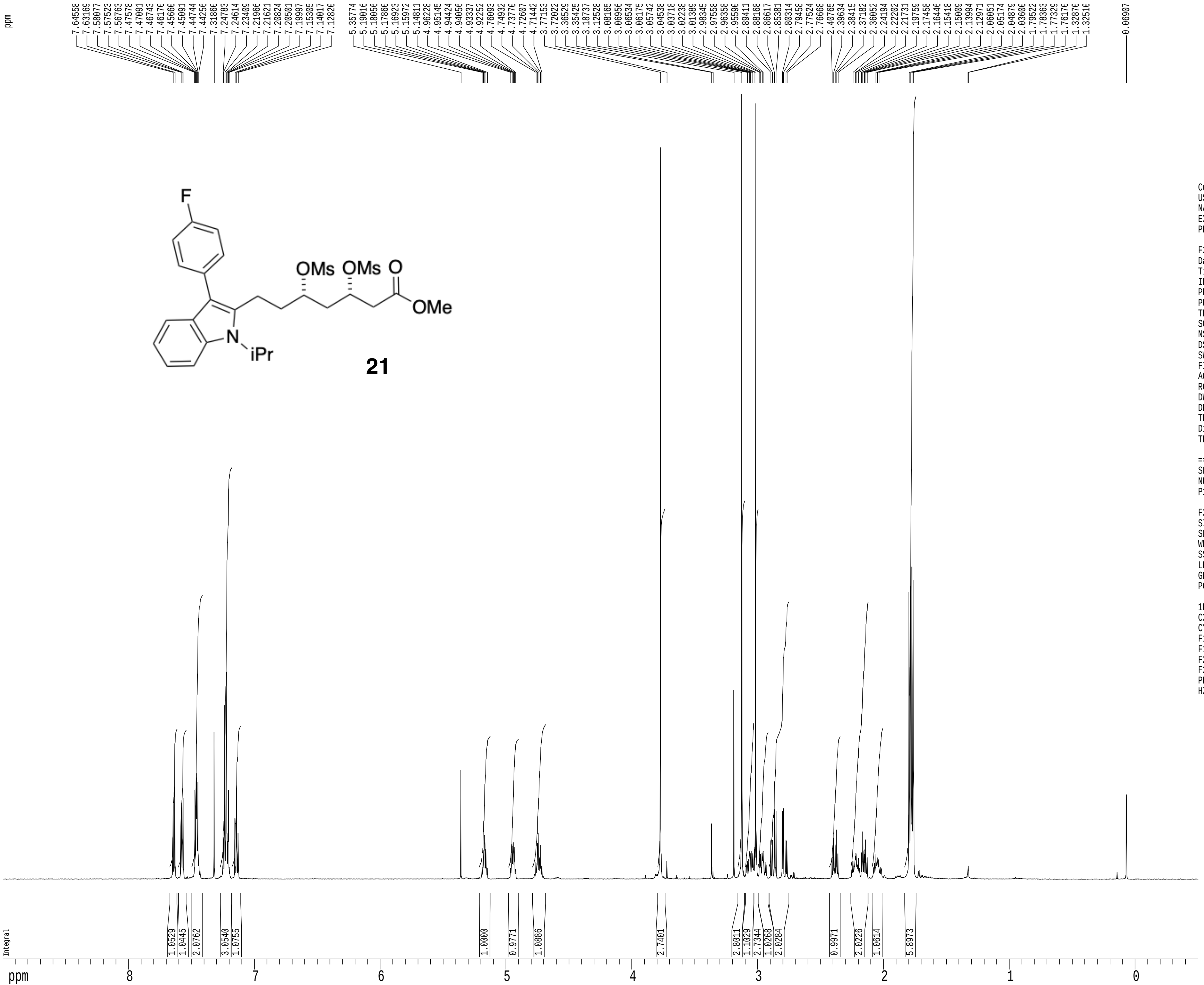

Current Data Parameters
USER mcginnit
NAME tmm-4-32-char
EXPNO 1
PROCNO 1

F2 - Acquisition Parameters
Date_ 20220224
Time 16.25
INSTRUM av600
PROBHD 5 mm CPBBO BB-
PULPROG zg30
TD 98074
SOLVENT CDCl3
NS 8
DS 2
SWH 9615.385 Hz
FIDRES 0.098042 Hz
AQ 5.0998979 sec
RG 10
DW 52.000 usec
DE 14.23 usec
TE 298.0 K
D1 0.10000000 sec
TD0 1

======== CHANNEL f1 ========
SFO1 600.1342009 MHz
NUC1 1H
P1 9.50 usec

F2 - Processing parameters
SI 65536
SF 600.1300000 MHz
WDW no
SSB 0
LB 0.00 Hz
GB 0
PC 1.00

1D NMR plot parameters
CX 22.80 cm
CY 15.00 cm
F1P 9.000 ppm
F1 5401.17 Hz
F2P -0.500 ppm
F2 -300.06 Hz
PPMCM 0.41667 ppm/cm
HZCM 250.05418 Hz/cm

13C spectrum

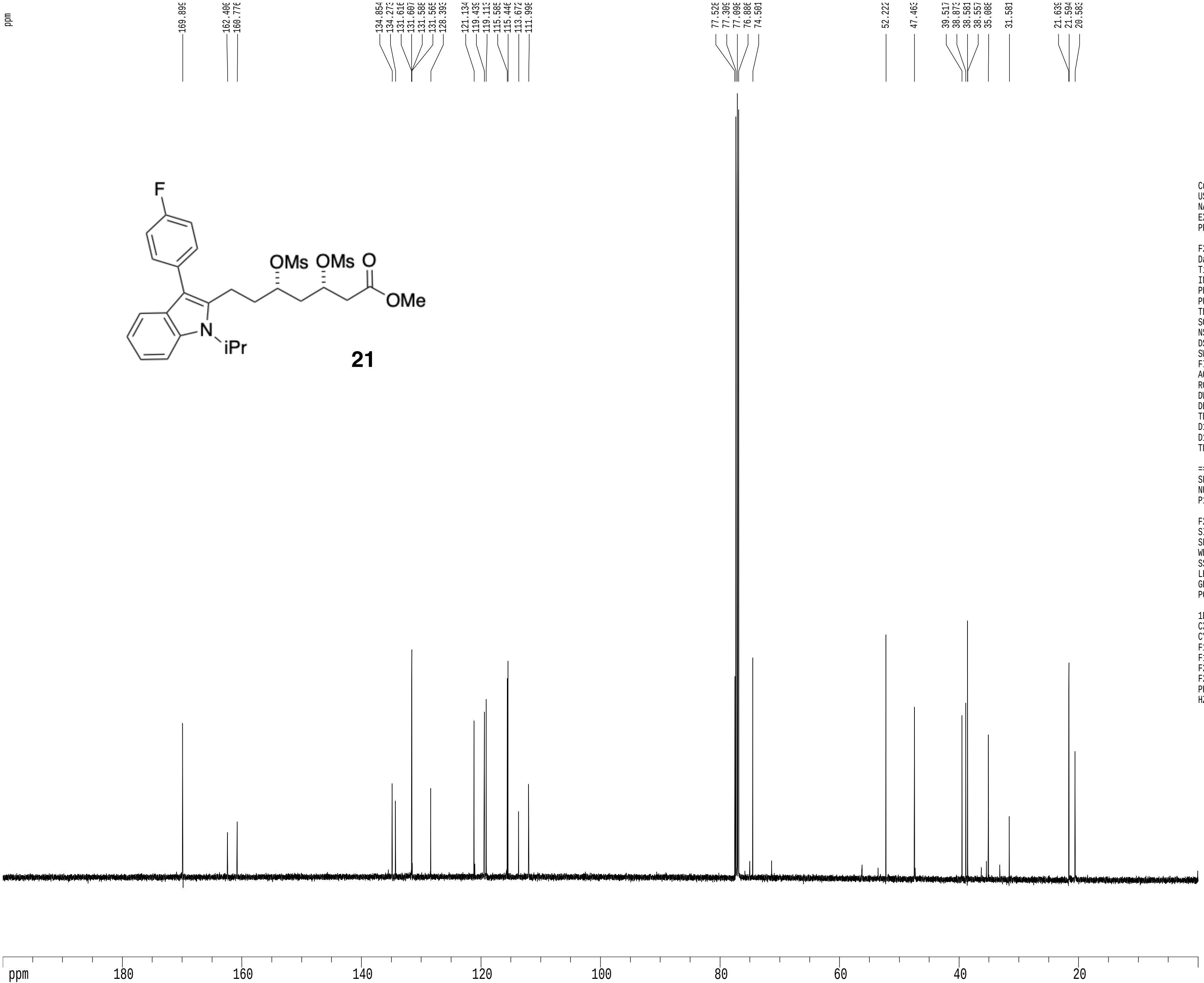

21


```
Current Data Parameters
USER          mcginnit
NAME      tmm-4-32-char
EXPNO                2
PROCNO               1

F2 - Acquisition Parameters
Date_          20220224
Time              16.30
INSTRUM           av600
PROBHD   5 mm CPBBO BB-
PULPROG          zgdc30
TD                65536
SOLVENT           CDCl3
NS                  106
DS                    4
SWH           36231.883 Hz
FIDRES         0.552855 Hz
AQ            0.9044468 sec
RG                 2050
DW               13.800 usec
DE                19.63 usec
TE                298.0 K
D1           0.40000001 sec
D11          0.03000000 sec
TD0                   1

======== CHANNEL f1 ========
SFO1        150.9194080 MHz
NUC1                13C
P1                10.10 usec

F2 - Processing parameters
SI                65536
SF          150.9028085 MHz
WDW                  no
SSB                   0
LB                 0.00 Hz
GB                    0
PC                 1.00

1D NMR plot parameters
CX                22.80 cm
CY                15.00 cm
F1P             200.000 ppm
F1             30180.56 Hz
F2P               0.000 ppm
F2                 0.00 Hz
PPMCM           8.77193 ppm/cm
HZCM         1323.70886 Hz/cm
```

19F spectrum

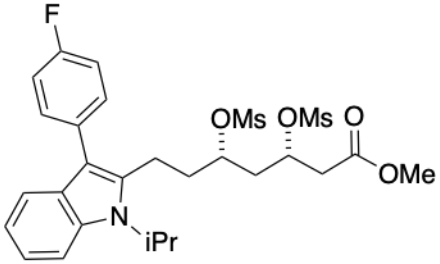

21

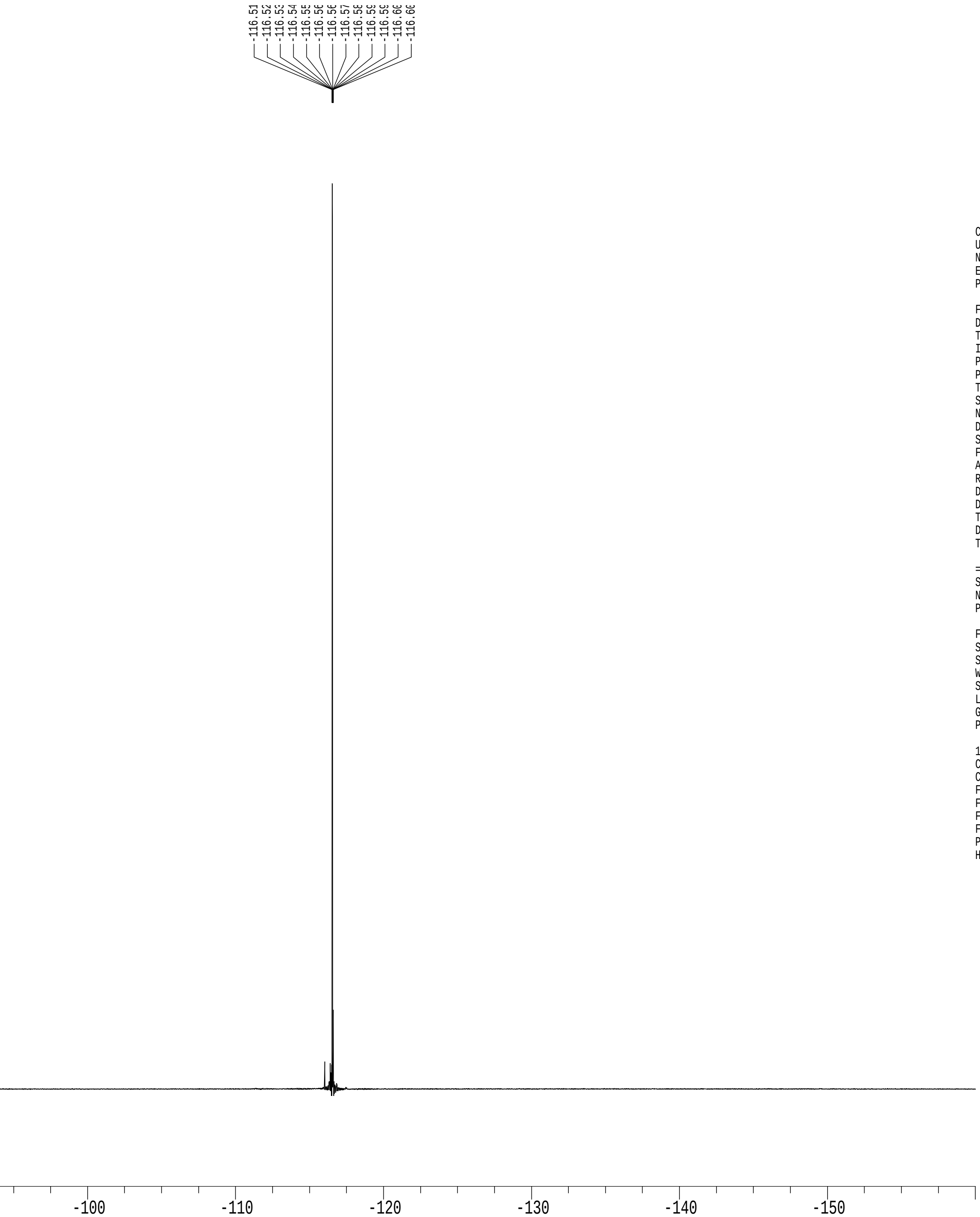

Current Data Parameters
USER mcginnit
NAME tmm-4-32-char
EXPNO 3
PROCNO 1

F2 - Acquisition Parameters
Date_ 20220224
Time 16.35
INSTRUM av600
PROBHD 5 mm CPBBO BB-
PULPROG zgflqn30
TD 131072
SOLVENT CDCl3
NS 16
DS 2
SWH 178571.422 Hz
FIDRES 1.362392 Hz
AQ 0.3670516 sec
RG 575
DW 2.800 usec
DE 18.00 usec
TE 298.0 K
D1 3.00000000 sec
TD0 1

======== CHANNEL f1 ========
SFO1 564.6299196 MHz
NUC1 19F
P1 18.25 usec

F2 - Processing parameters
SI 131072
SF 564.6863858 MHz
WDW no
SSB 0
LB 0.00 Hz
GB 0
PC 1.00

1D NMR plot parameters
CX 22.80 cm
CY 15.00 cm
F1P -60.000 ppm
F1 -33881.19 Hz
F2P -160.000 ppm
F2 -90349.83 Hz
PPMCM 4.38596 ppm/cm
HZCM 2476.69482 Hz/cm

1H spectrum

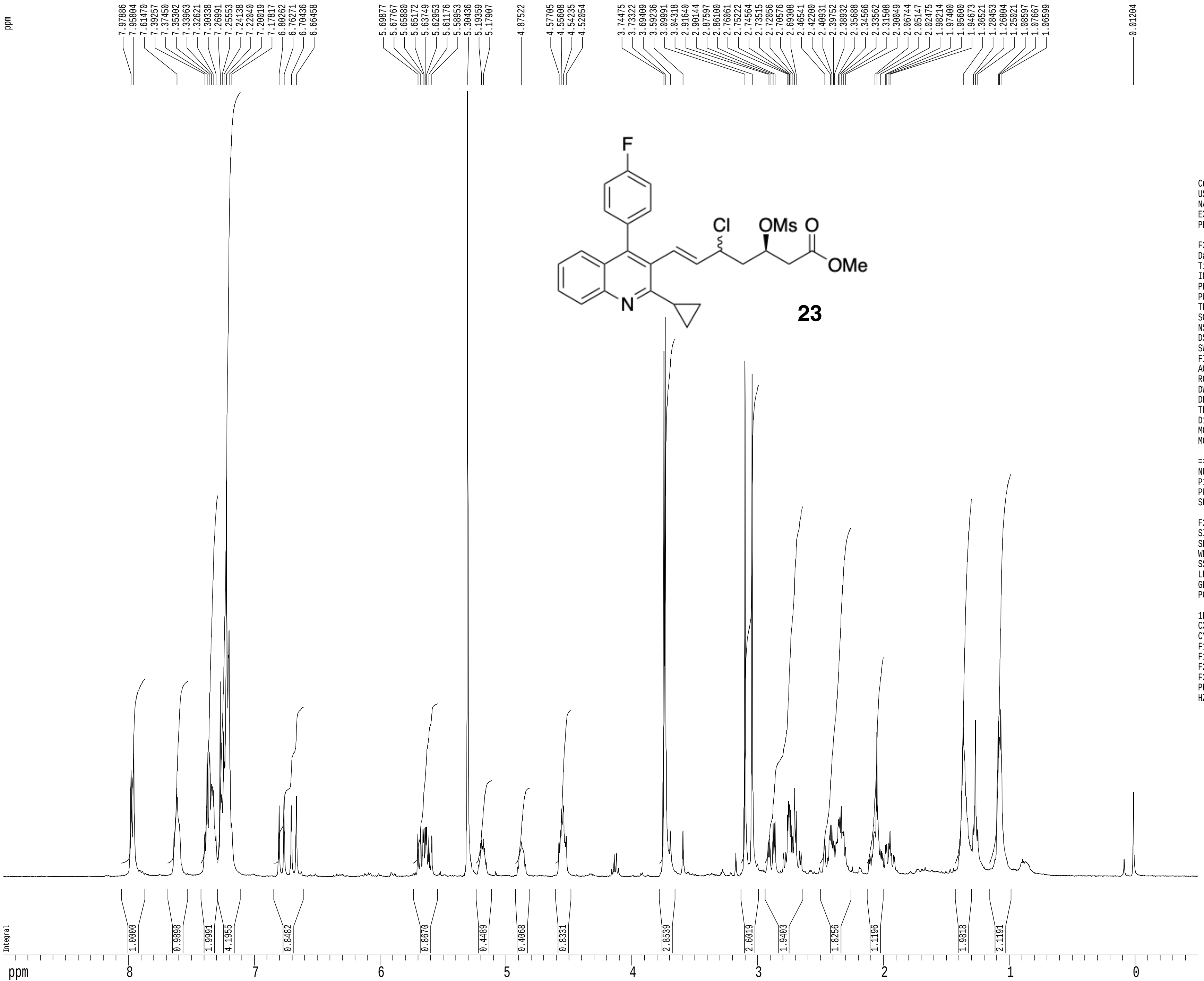

Current Data Parameters
USER mcginnit
NAME tmm-4-095-char
EXPNO 1
PROCNO 1

F2 - Acquisition Parameters
Date_ 20220210
Time 10.53
INSTRUM drx400
PROBHD 5 mm QNP H/F/P
PULPROG zg30
TD 65536
SOLVENT CDCl3
NS 8
DS 2
SWH 6410.256 Hz
FIDRES 0.097813 Hz
AQ 5.1118579 sec
RG 181
DW 78.000 usec
DE 4.50 usec
TE 298.0 K
D1 0.10000000 sec
MCREST 0.00000000 sec
MCWRK 0.01500000 sec

======== CHANNEL f1 ========
NUC1 1H
P1 12.00 usec
PL1 -0.90 dB
SFO1 400.1328009 MHz

F2 - Processing parameters
SI 65536
SF 400.1300175 MHz
WDW EM
SSB 0
LB 0.30 Hz
GB 0
PC 2.00

1D NMR plot parameters
CX 22.80 cm
CY 15.00 cm
F1P 9.000 ppm
F1 3601.17 Hz
F2P -0.500 ppm
F2 -200.06 Hz
PPMCM 0.41667 ppm/cm
HZCM 166.72084 Hz/cm

13C spectrum with 1H decoupling

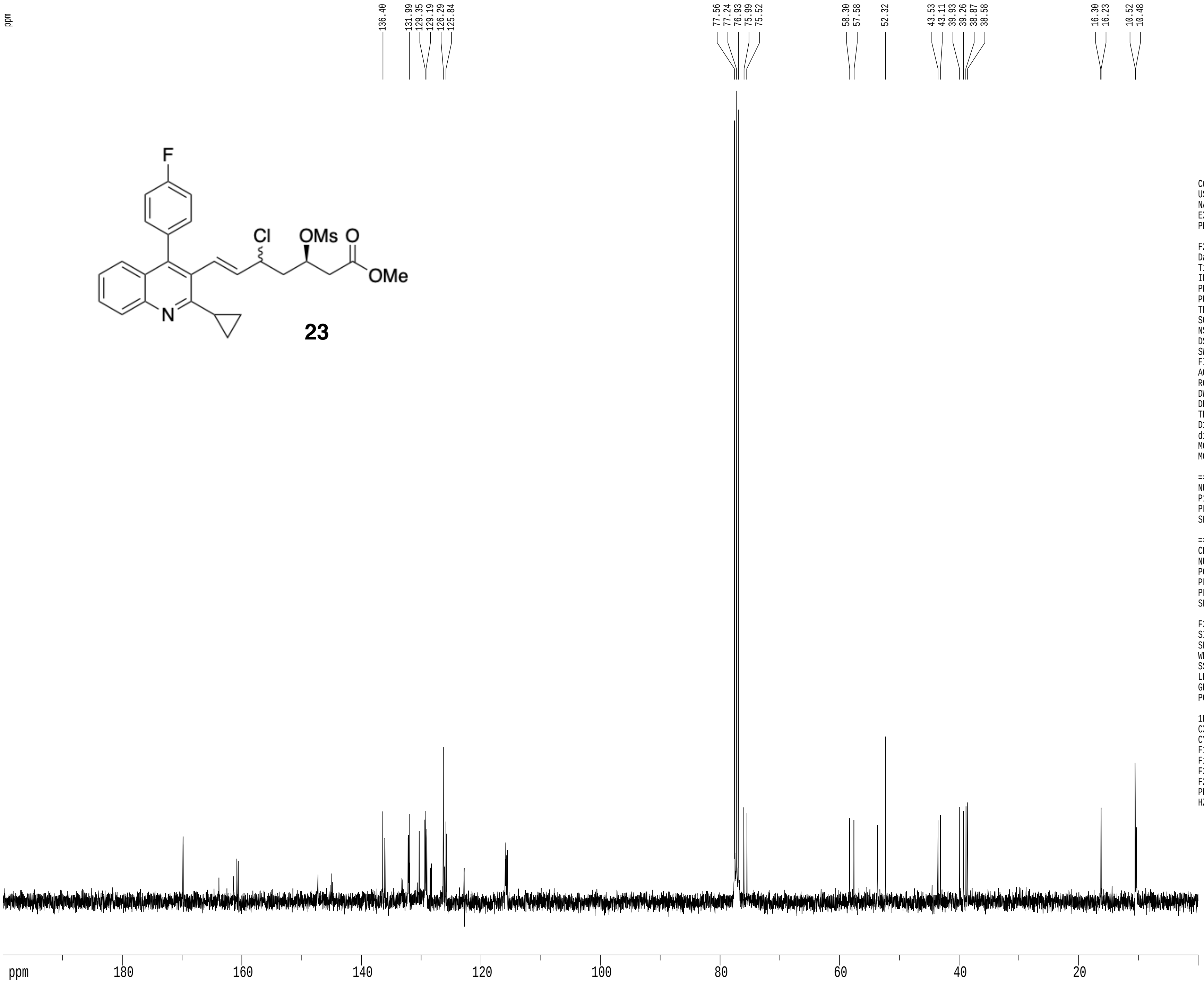


```
Current Data Parameters
USER           mcginnit
NAME     tmm-4-095-char
EXPNO                 2
PROCNO                1

F2 - Acquisition Parameters
Date_          20220210
Time              10.56
INSTRUM          drx400
PROBHD   5 mm QNP H/F/P
PULPROG          zgdc30
TD                65536
SOLVENT           CDCl3
NS                  606
DS                    4
SWH           24154.590 Hz
FIDRES         0.368570 Hz
AQ            1.3566452 sec
RG               9195.2
DW               20.700 usec
DE                20.39 usec
TE                298.0 K
D1           0.10000000 sec
d11          0.03000000 sec
MCREST       0.00000000 sec
MCWRK        0.01500000 sec

======== CHANNEL f1 ========
NUC1                13C
P1                 7.90 usec
PL1               -3.00 dB
SFO1        100.6237964 MHz

======== CHANNEL f2 ========
CPDPRG2         waltz16
NUC2                 1H
PCPD2             90.00 usec
PL2               -0.90 dB
PL12              17.00 dB
SFO2        400.1328009 MHz

F2 - Processing parameters
SI                65536
SF          100.6127500 MHz
WDW                  EM
SSB                   0
LB                 1.00 Hz
GB                    0
PC                 1.00

1D NMR plot parameters
CX                22.80 cm
CY                15.50 cm
F1P             200.000 ppm
F1             20122.55 Hz
F2P               0.000 ppm
F2                 0.00 Hz
PPMCM           8.77193 ppm/cm
HZCM          882.56799 Hz/cm
```

19F spectrum

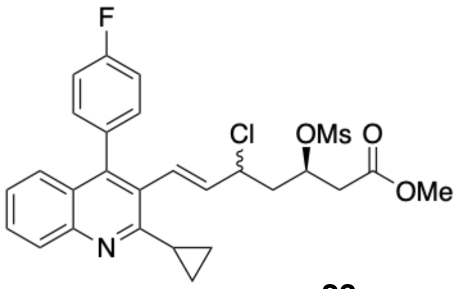

23

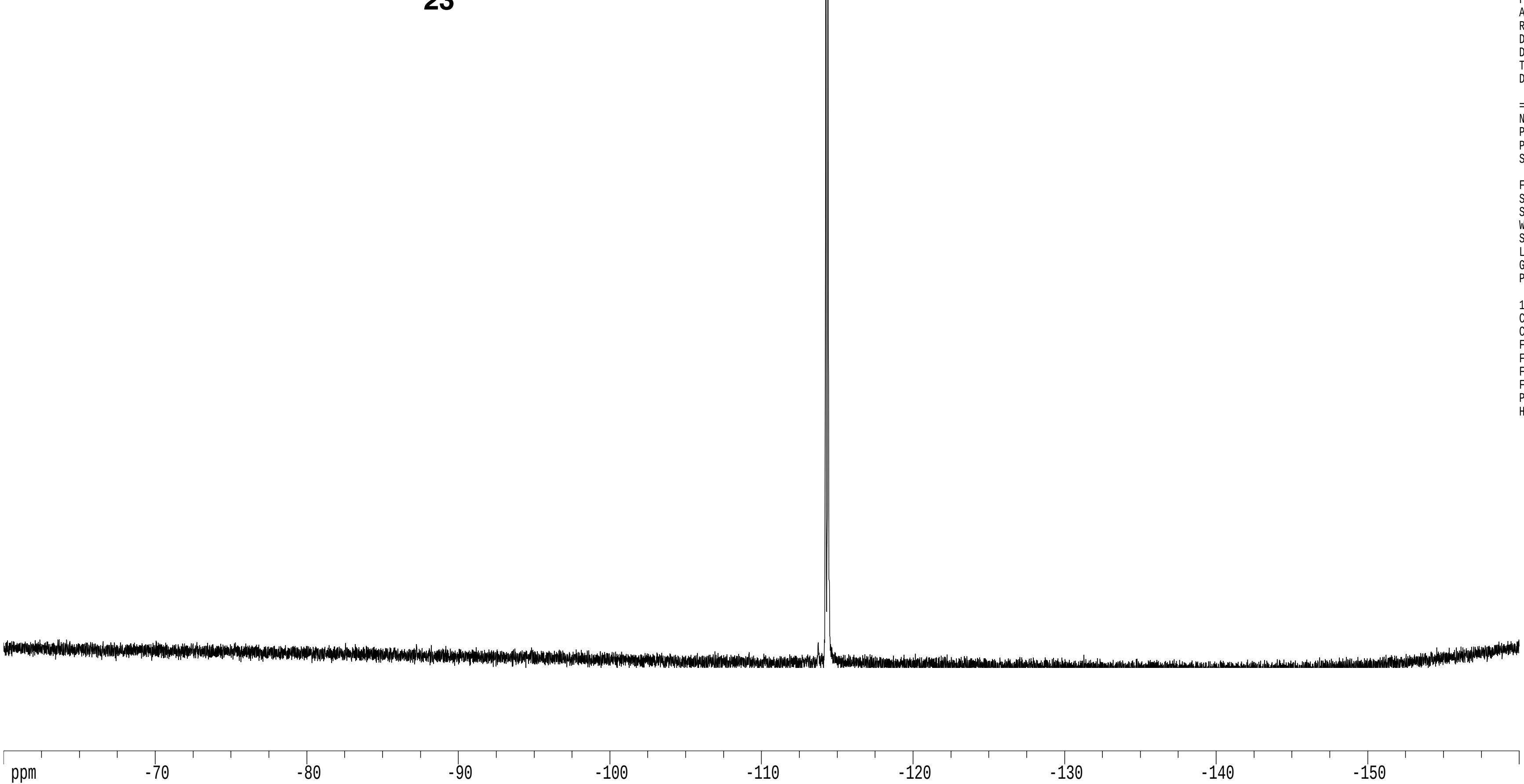

Current Data Parameters	
USER	mcginnit
NAME	tmm-4-095-char
EXPNO	3
PROCNO	1
F2 - Acquisition Parameters	
Date_	20220210
Time	11.11
INSTRUM	drx400
PROBHD	5 mm QNP H/F/P
PULPROG	zgflqn30
TD	65536
SOLVENT	CDCl3
NS	39
DS	2
SWH	75187.969 Hz
FIDRES	1.147277 Hz
AQ	0.4358644 sec
RG	1149.4
DW	6.650 usec
DE	9.46 usec
TE	298.0 K
D1	2.00000000 sec
======== CHANNEL f1 ========	
NUC1	19F
P1	21.75 usec
PL1	-6.00 dB
SFO1	376.4646491 MHz
F2 - Processing parameters	
SI	65536
SF	376.4984640 MHz
WDW	EM
SSB	0
LB	1.00 Hz
GB	0
PC	1.00
1D NMR plot parameters	
CX	22.80 cm
CY	15.00 cm
F1P	-60.000 ppm
F1	-22589.91 Hz
F2P	-160.000 ppm
F2	-60239.76 Hz
PPMCM	4.38596 ppm/cm
HZCM	1651.30908 Hz/cm

1H spectrum

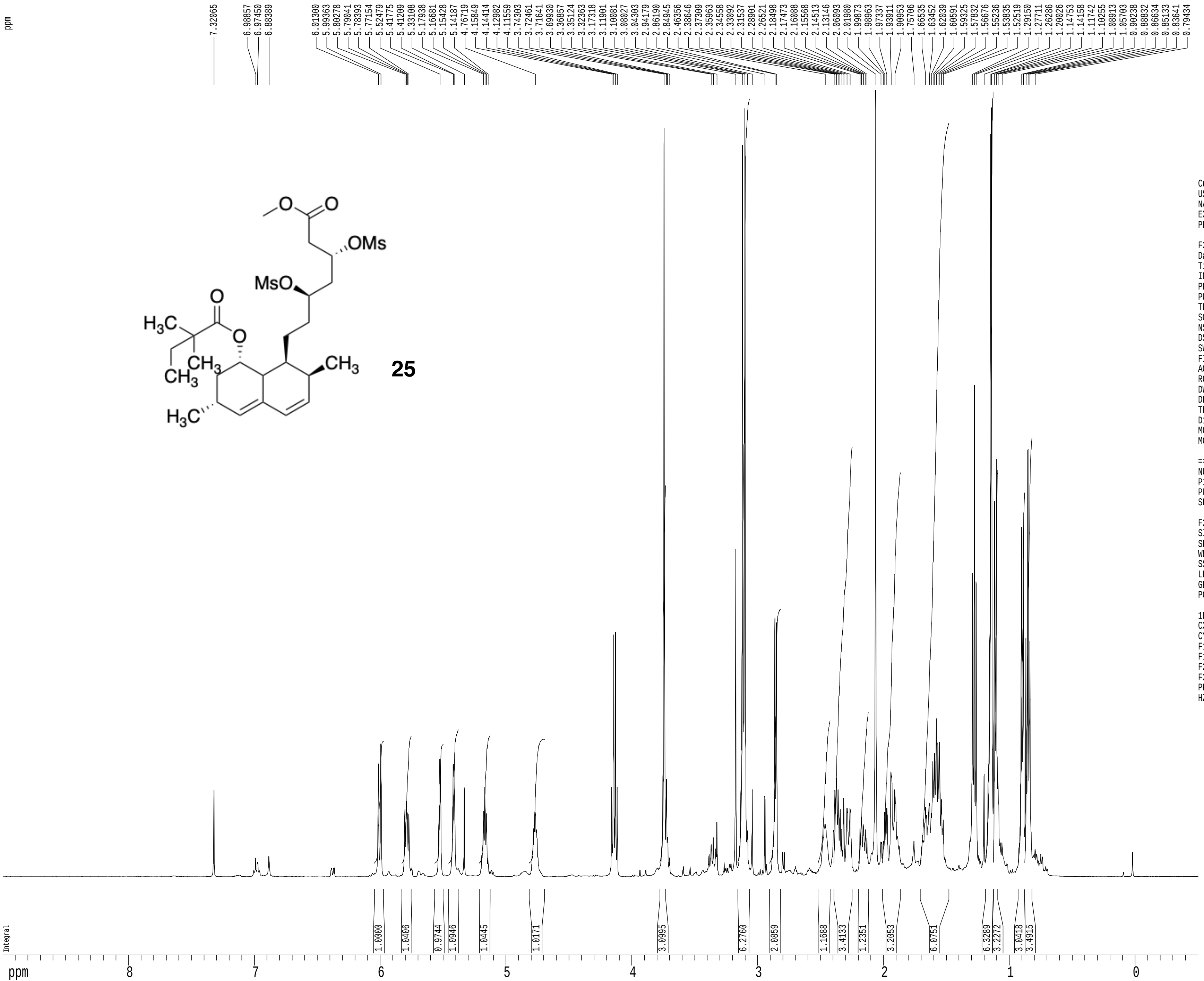

25

```
Current Data Parameters
USER            mcginnit
NAME        tmm-4-048-char
EXPNO                  1
PROCNO                 1

F2 - Acquisition Parameters
Date_          20220314
Time              11.05
INSTRUM         cryo500
PROBHD   5 mm CPTCI 1H-
PULPROG            zg30
TD                81728
SOLVENT           CDCl3
NS                    8
DS                    2
SWH            8012.820 Hz
FIDRES         0.098043 Hz
AQ            5.0998774 sec
RG                  2.8
DW               62.400 usec
DE                 6.00 usec
TE                298.0 K
D1           0.10000000 sec
MCREST       0.00000000 sec
MCWRK        0.01500000 sec

======== CHANNEL f1 ========
NUC1                 1H
P1                 9.75 usec
PL1                1.60 dB
SFO1        500.2235015 MHz

F2 - Processing parameters
SI                65536
SF          500.2200000 MHz
WDW                  EM
SSB                   0
LB                 0.30 Hz
GB                    0
PC                 1.00

1D NMR plot parameters
CX                22.80 cm
CY                15.00 cm
F1P               9.000 ppm
F1              4501.98 Hz
F2P              -0.500 ppm
F2              -250.11 Hz
PPMCM           0.41667 ppm/cm
HZCM          208.42500 Hz/cm
```

Z-restored spin-echo 13C spectrum with 1H decoupling

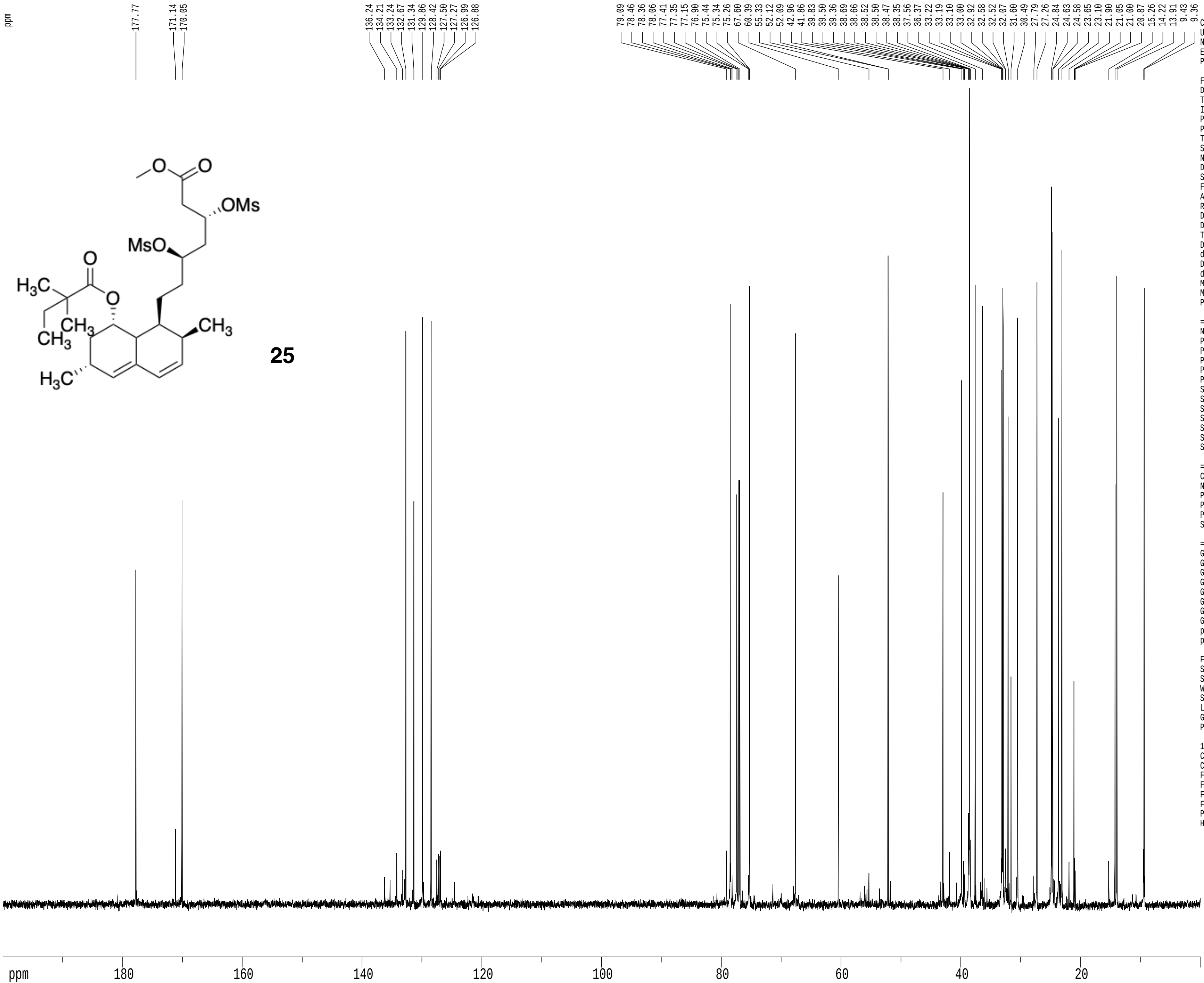


```
Current Data Parameters
USER          mcginnit
NAME     tmm-4-048-char
EXPNO                2
PROCNO               1

F2 - Acquisition Parameters
Date_         20220314
Time             11.12
INSTRUM        cryo500
PROBHD   5 mm CPTCI 1H-
PULPROG  SpinEchopg30gp2.prd
TD               65536
SOLVENT          CDCl3
NS                 309
DS                  16
SWH          30303.031 Hz
FIDRES        0.462388 Hz
AQ           1.0813940 sec
RG              3649.1
DW              16.500 usec
DE                6.00 usec
TE               298.0 K
D1          0.25000000 sec
d11         0.03000000 sec
D16         0.00020000 sec
d17         0.00019600 sec
MCREST      0.00000000 sec
MCWRK       0.01500000 sec
P2               37.70 usec

======== CHANNEL f1 ========
NUC1               13C
P1               18.85 usec
P12            2000.00 usec
P20             500.00 usec
PL0             120.00 dB
PL1              -1.00 dB
SFO1       125.7942548 MHz
SP2               1.55 dB
SP4               1.55 dB
SPNAM2     Crp60comp.4
SPNAM4   Crp60,0.5,20.1
SPOFF2            0.00 Hz
SPOFF4            0.00 Hz

======== CHANNEL f2 ========
CPDPRG2         waltz16
NUC2                1H
PCPD2           100.00 usec
PL2               1.60 dB
PL12             22.00 dB
SFO2       500.2225011 MHz

====== GRADIENT CHANNEL =====
GPNAM1        SINE.100
GPNAM2        SINE.100
GPX1              0.00 %
GPX2              0.00 %
GPY1              0.00 %
GPY2              0.00 %
GPZ1             30.00 %
GPZ2             50.00 %
p15             500.00 usec
p16            1000.00 usec

F2 - Processing parameters
SI               65536
SF         125.7804190 MHz
WDW                 EM
SSB                  0
LB                1.00 Hz
GB                   0
PC                2.00

1D NMR plot parameters
CX               22.80 cm
CY               15.65 cm
F1P            200.000 ppm
F1            25156.08 Hz
F2P              0.000 ppm
F2                0.00 Hz
PPMCM          8.77193 ppm/cm
HZCM        1103.33704 Hz/cm
```
